# Context-Specific Metabolic Model Extraction Based on Regularized Least Squares Optimization

**DOI:** 10.1371/journal.pone.0131875

**Published:** 2015-07-09

**Authors:** Semidán Robaina Estévez, Zoran Nikoloski

**Affiliations:** Systems Biology and Mathematical Modeling Group, Max Planck Institute of Molecular Plant Physiology (MPIMP), Potsdam-Golm, Germany; Virginia Commonwealth University, UNITED STATES

## Abstract

Genome-scale metabolic models have proven highly valuable in investigating cell physiology. Recent advances include the development of methods to extract context-specific models capable of describing metabolism under more specific scenarios (*e*.*g*., cell types). Yet, none of the existing computational approaches allows for a fully automated model extraction and determination of a flux distribution independent of user-defined parameters. Here we present RegrEx, a fully automated approach that relies solely on context-specific data and ℓ_1_-norm regularization to extract a context-specific model and to provide a flux distribution that maximizes its correlation to data. Moreover, the publically available implementation of RegrEx was used to extract 11 context-specific human models using publicly available RNAseq expression profiles, Recon1 and also Recon2, the most recent human metabolic model. The comparison of the performance of RegrEx and its contending alternatives demonstrates that the proposed method extracts models for which both the structure, *i*.*e*., reactions included, and the flux distributions are in concordance with the employed data. These findings are supported by validation and comparison of method performance on additional data not used in context-specific model extraction. Therefore, our study sets the ground for applications of other regularization techniques in large-scale metabolic modeling.

## Introduction

The investigation and understanding of cell metabolism has experienced a paradigm shift largely propelled by the development of high-throughput methods in the last two decades. As a result, the classical pathway-centered view have given way for a network-driven perspective, which considers the entire set of known interconnected biochemical reactions. This had led to the creation of genome-scale metabolic models (GEMs) for organisms from each of the three domains of life: archaea, bacteria and eukarya [1]. While a GEM constitutes an organized and comprehensive system of knowledge about an organism, it also allows *in silico* analyses based on constraint-based methods, relying on the corresponding stoichiometric matrix representation and assumptions about cellular metabolism. The findings from these analyses provide useful insights in metabolism, and may circumvent the drawbacks of estimating fluxes from labeling studies—still a computationally demanding undertaking [2–4]. Furthermore, several methods facilitate the integration of high-throughput data in GEMs. The benefits of these methods are twofold: improving the accuracy of flux prediction and providing a scaffold network for analysis of additional experimental data [5,6].

However, a metabolic network that includes all known biochemical reactions of an organism may not be realistic in a particular cellular scenario, since there is mounting evidence that cells adapt their metabolism to arising conditions, such as: external environment, developmental stage, cell type in multicellular organisms, or even during a pathological condition (e.g., cancer), to name only a few. In these different *contexts*, only a subset of reactions is typically active. Therefore, the shift towards reconstructing context-specific models of cell metabolism has become necessary to provide more accurate and biologically meaningful insights. This is of particular importance when tackling the physiology of multicellular organisms, not only to better understand tissue- or cell-specific metabolism, but as a first step to reconstruct metabolic networks of an entire organism/body, where multiple specialized models are mutually interconnected [7,8].

Several methods have been proposed to determine context-specific networks, already comprehensively reviewed in [9]. In general, the methods for extracting a context-specific model from a given GEM integrate high-throughput data from a particular context to select the set of respective active reactions. While these methods differ with respect to their underlying assumptions and mathematical formulation, they can be classified into three main groups [10], briefly discussed in the following.

GIMME [11] and GIM^3^E [12] form the first group, whereby first a metabolic functionality (*e*.*g*., biomass production) is optimized through Flux Balance Analysis [13] (a linear mathematical program), and then the obtained optimal value is employed to constrain a second linear program which aims at minimizing the discrepancies between fluxes and data. The latter is based on selecting a user-defined data-dependent threshold value and then penalizing reactions whose associated data is under the threshold.

The second group comprises iMAT [14] and INIT [15] which use a mixed integer linear program. The binary variables in this formulation select the reaction states (*i*.*e*., active or inactive) which are most concordant with the associate data state. While iMAT uses data to pre-classify reactions of the GEM into active or inactive groups, INIT integrates data as a weighting factor for the binary variable. Moreover, INIT includes a set of key metabolites which must exhibit a small positive deviation from the steady state condition. In an extended version, tINIT [16], a set of metabolic tasks (*i*.*e*., biochemical pathways) that must carry non-zero flux can be added as further constraints.

The third group, composed of MBA [17], mCADRE [18] and FastCORE [19], first defines a core set of reactions, classified as active in a given context according to experimental data, and then finds the minimum set of reactions outside the core required to satisfy the model consistency condition (*i*.*e*., all reactions in the model must be able to carry a non-zero flux in at least one of the allowed steady-state distributions). Unlike the methods in the previous two groups, these only extract a context-specific model and do not provide a respective flux distribution.

With respect to another classification criterion, the first group belongs to the so-called biased methods (within the constraint-based analysis) since the achieved solution depends on the definition of a metabolic function to be optimized. In contrast, the second and third group consist of unbiased methods since they are independent of a metabolic function [20]. However, in the case of iMAT, MBA and FastCORE, a group of preferential reactions (to the context of choice) must still be predefined. The choice of unbiased methods is of particular importance when the metabolic functions to be optimized under a given context may be difficult to obtain and justify; for instance, this is the case when dealing with multicellular organisms, where multiple cell types coexist (cooperate and/or compete) while performing a variety of specialized metabolic functions instead of optimizing a single general function.

However, none of these methods allow fully automated model extraction and flux prediction, without using *a priori* knowledge of a context-specific function and without any binarization or pre-classification of reactions in the process of data integration. This is of particular importance when a large number of context-specific models are to be extracted or, more relevant for not well-characterized organisms, for which no information regarding the context-specificity of reactions or metabolic function may be available in the existing databases or from detailed physiological studies. Here we present RegrEx, an approach based on regularized least squares optimization that fulfills these tasks of extracting context-specific models and providing a flux distribution in an automated and unbiased way.

## Methods

### Background

Regularization is commonly applied when modeling (*i*.*e*., learning) high-dimensional functions from observations, as a means to reduce model complexity (*i*.*e*., the number of variables included in the model) and prevent overfitting to background noise. The latter has been shown to considerably improve prediction performance and model robustness. Several regularization methods have already been proposed, including: the Dantzig [21], the Ridge [22] and the Elastic Net [23] selectors. However, a particular one, the Least Absolute Shrinkage and Selection Operator, abbreviated as LASSO [24], has become very popular in high-dimensional regression problems with *n* explanatory variables and *m* observations, where *n*≫*m*. This has been largely due to a better performance of the LASSO selector in feature selection (typically obtaining sparse models with a minimum number of explanatory variables) along with the simplicity of the operator, which facilitates its computation [25,26].

The LASSO optimization problem is given in Eq 1, below, whereby a weighted ℓ_1_-norm on the coefficients, β, as regularization to an ordinary least squares regression with response vector, y^*m*^, and variables, X^mxn^, is minimized:

$$\begin{array}{l} \underset{\beta }{\text{min}}\frac{1}{2}||y-X\beta |{|}_{2}^{2}+\lambda ||\beta |{|}_{1}\\ \beta \in {\mathbb{R}}^{n} \end{array}$$ (1)

The weighting parameter, λ, is usually determined by cross-validation, which offers an unbiased way (*i*.*e*., not user-defined and purely based on the data) to find a best suited value with respect to a measure of performance (*e*.*g*., mean squared error or coefficient of determination). Regularization by means of the ℓ_1_-norm, as generalizations of LASSO, has been already applied in metabolic modeling; for instance, it has been used to reconstruct biochemical networks from time series data [27], as an alternative to more computationally expensive methods, to study network adaptation to mutations [28] and, more recently, in FastCORE, one of the existing algorithms to reconstruct context-specific models [19].

### The RegrEx method

The Regularized Context-specific model Extraction method (RegrEx) aims at finding a feasible flux distribution, *v* (*i*.*e*., satisfying the mass-balance, thermodynamic and capacity constraints), which is as close as possible to the experimental data, *d* (*e*.*g*., gene expression or protein level profiles). At the same time, it excludes the reactions irrelevant for the given context by shrinking their fluxes to zero. This is obtained by minimizing the squared Euclidean distance (second norm) between *v* and *d*, and exploiting the ability of the ℓ_1_-norm regularization to perform feature selection. This leads to the optimization problem, Eq 2, which is analogous to the formulation of LASSO in Eq 1 augmented by the cellular constraints, and is given by:

$$\begin{array}{l} \underset{v}{\text{min}}\frac{1}{2}||d-v|{|}_{2}^{2}+\lambda ||v|{|}_{1}\\ s.t.\;\;\;\;\;\\ Sv=0\\ v_{i}\geq 0,\;\;\;\;\;i\in IrvRxn\\ v_{min}\leq v\leq v_{max}\\ v\in {\mathbb{R}}^{n} \end{array}$$ (2)

To implement RegrEx (Eq 2) in existing mathematical programming solvers, we cast it as a quadratic program, Eq 3, which minimizes the second norm of the error vector, *ϵ* = *d*−*v*, considering only the subset of reactions to which data can be associated (via the GPR associations [29]). We also need to introduce special constraints to deal with reversible reactions, which can take negative values (while the data vector is always non-negative). To this end, reversible reactions are split into forward and reverse; the net flux is then given by the difference of the respective fluxes. In addition, we included a binary variable, *x*, to select either the forward or the reverse sense for a particular reaction to avoid the drawback of bounding the two irreversible reactions to the same data value, which would cause the net flux to be zero.

$$\begin{array}{l} \underset{E,V}{\text{min}}\frac{1}{2}||\varepsilon |{|}_{2}^{2}+\lambda ||v|{|}_{1}\\ s.t.\;\;\;\;\;\\ Sv=0\\ v_{i}+\varepsilon_{i}=d_{i}\text{, }i\in Data\;\\ v_{i}\geq 0,\;\;\;\;i\in IrvRxn.\\ v_{min}\leq v\leq v_{max}\\ -\varepsilon_{\text{max}}\leq \varepsilon \leq \varepsilon_{max}\\ \varepsilon ,v\in {\mathbb{R}}^{n} \end{array}$$ (3)

Altogether, this resulted in a mixed integer quadratic program (MIQP) capturing the RegrEx method, Eq 4, in which the sign of the net flux for the reversible reactions (*i*.*e*., predominant direction of the two irreversible reactions) is part of the optimization problem and relies ultimately on maximizing similarity to data.

As pointed out above, cross-validation is the canonical method to determine an optimal λ-value for a regression problem. However, this is not an appropriate method for RegrEx due to its particular characteristics, since consecutive sampling of the observations, in this case, would imply using only a subset of reactions of the original metabolic model to be in a steady-state. For this reason, we optimized λ selection by running the algorithm for a sequence of λ-values and taking the one that renders the highest Pearson product-moment correlation between fluxes and data. RegrEx was applied first over a coarse sequence of λ-values (λ step size of 0.1) until all reactions shrunk to zero. Then a second optimization was performed over a finer λ-sequence (λ step size of 0.01) centered on the region of maximum correlation found in the first optimization.

$$\begin{array}{l} \underset{\varepsilon ,v,x}{\text{min}}\frac{1}{2}||\varepsilon |{|}_{2}^{2}+\lambda ||v|{|}_{1}\\ s.t.\;\;\;\\ Sv=0\\ v_{i}+{Ú}_{i}=d_{i},\;\;\;i\in IrvRxn\cap Data\;\\ \begin{array}{l} v_{for{\;}_{i}}+{Ú}_{for{\;}_{i}}-\;x_{i}d_{i}=d_{i}\\ v_{rev{\;}_{i}}+{Ú}_{rev{\;}_{i}}-x_{i}d_{i}\;=\;0 \end{array}\},\;\;i\in RevRxn\cap Data\\ \begin{array}{l} v_{for{\;}_{i}}+x_{i}v_{{\text{max}}_{i}}\leq v_{{\text{max}}_{i}}\\ v_{rev{\;}_{i}}-x_{i}v_{{\text{max}}_{i}}\leq 0\; \end{array}\},\;\;\;i\in RevRxn\\ \begin{array}{l} v_{for{\;}_{i}}+x_{i}v_{{\text{min}}_{i}}\geq v_{{\text{min}}_{i}}\\ v_{rev{\;}_{i}}-x_{i}v_{{\text{min}}_{i}}\geq 0\; \end{array}\},\;\;\;i\in RevRxn.\\ v_{min}\leq v\leq v_{max}\\ -\varepsilon_{max}\leq \varepsilon \leq \varepsilon_{max}\\ \varepsilon ,v\in {\mathbb{R}}^{n}\\ x\in {\left\{0,1\right\}}^{n} \end{array}$$ (4)

### RegrEx implementation

We solved the MIQP of RegrEx using the Gurobi solver [30]. To speed up the optimization, we restricted the computation time to 60 seconds per MIQP. Additional robustness analyses indicated that higher computation times implied a low increase in performance (tripling the time limit, *i*.*e*., 180 seconds, only caused a mean correlation increment between models of 0.0004, see Table 1).

**Table 1:** Results comparison for different time limits applied to the Gurobi solver. Four different time limits were evaluated to test the sensitivity of optimal solutions to the early termination criterion (60 s) imposed. In all cases, the λ-value was fixed to a reference optimum, the one obtained when the time limit was 60 s. Mean values for the 11 contexts (with the standard deviation within round brackets) are shown for the correlation between flux values and data, $\overset{-}{\rho }$_(V,D)_, the mean residual, $\overset{-}{R}$_(V,D)_, and the cardinality, *i*.*e*., number of reactions of the extracted models, $\overset{-}{Card.}$.

	30s	60s	90s	180s
_$\bar{\rho }$(V,D)_	0.4493(.068)	0.4493(.068)	0.4493(.068)	0.4497(.068)
_$\bar{R}$(V,D)_	0.2845(.081)	0.2845(.081)	0.2845(.081)	0.2844(.081)
$\bar{Card.}$	856.55(92.124)	856.27(91.153)	855.45(91.35)	856.55(93.17)

RegrEx was implemented in MATLAB, and the code is provided in S1 File. The implementation provides the final context-specific models in a COBRA toolbox compatible format [31], thus allowing facile subsequent analysis.

### Context-specific model extraction

To test RegrEx performance we selected, as a case study, the existent human metabolic network reconstructions, Recon 1 [32]which has been previously used with other algorithms [15,19,33] and Recon 2 [34] as a further test on a larger network,. This case study allows a direct comparison between the models extracted by different methods. As input data, we used available RNAseq human expression profiles for 11 different contexts (*i*.*e*., organs or tissues) published online in the RNAseq Atlas [35]. To avoid blocked reactions, we first extracted the consistent part of Recon 1 (cRecon 1) through a standard flux variability analysis, using the *reduceModel* function of the COBRA toolbox [31]. The expression profiles are provided as RPKM (reads per kilobase per million mapped reads) normalization in the RNAseq Atlas.

The range of expression values typically varies between genes, especially in RNAseq-derived expression data, where differences in mean values (*e*.*g*., across tissues) between genes can be of several orders of magnitude. This may likely cause RegrEx to favor reactions whose associated genes have higher mean values across contexts, thus, reconstructing context-specific models in a biased manner. To correct for this bias, we normalized the expression value, *t*, of each gene, *i*, in context, *j*, to its maximum value across all considered contexts:

$$d_{i,j\;}=\frac{t_{i,j}}{\text{max}(t_{i,\forall j})},\text{ i}\in genes,j\in contexts$$

### Performance analysis with competing methods

The existing iMAT implementation of the *reduceModel* function of the COBRA toolbox [31] was used to perform the iMAT model extraction. The 75^th^ percentile of the cumulative distribution was used as a threshold to binarize the gene expression data, *i*.*e*., to create the high- and low-expressed (reaction associated gene/s) groups. The implementation provided in [36] was used to analyze the model extraction approach, denoted as Lee2012. Since the RNAseq Atlas does not provide any variance measurement, the weighting factor to correct for experimental error was not used. In addition, the upper bound on flux values was set to 1, as in RegrEx, for fair comparison. Reactions with an absolute value above 10^−6^ were considered active for a given context. In the case of FastCORE, we used the implementation provided in [37] and obtained the core set of reaction by taking the reactions with an expression value for the associated gene(s) above the 75^th^ percentile; therefore, it uses the same set as the high-expressed group employed in iMAT.

**Fig 1:**
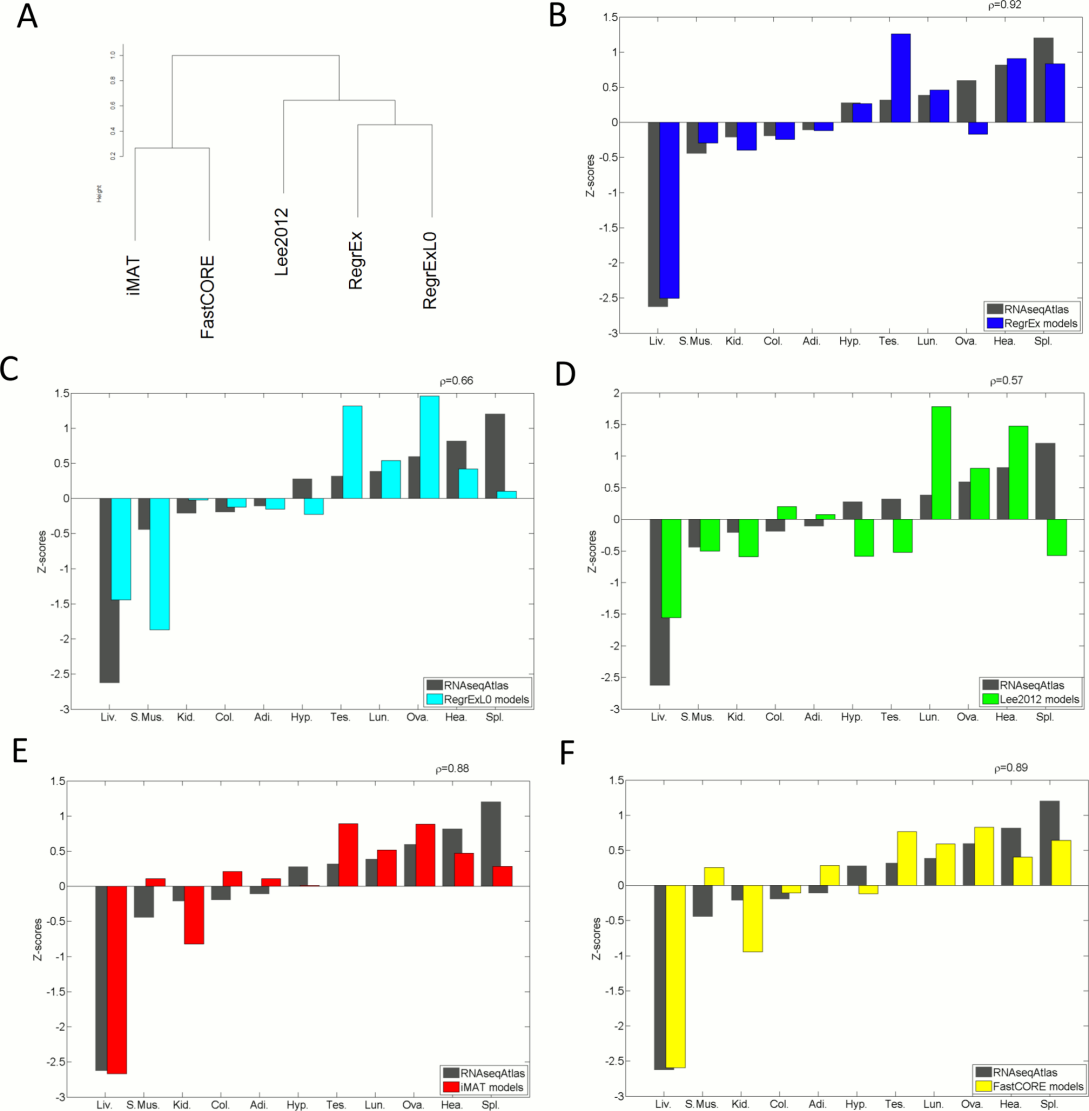
Dendrogram clustering the evaluated methods and comparison of data- and model-derived z-scores quantifying the differences between contexts. (A) The dendrogram is obtained from the the Jaccard similarity that models have across the different methods. Two main clusters are formed, iMAT and FastCORE on one side, and Lee2012, RegrEx-λ_0_ and RegrEx on the other. In the second cluster, RegrEx and RegrEx-λ_0_ form a subcluster. (B-F) data- and model-derived z-scores are compared for RegrEx, RegrEx-λ_0_, Lee2012, iMAT and FastCORE, respectively. Correlation values between the two series (data and model) are shown in the right upper corner in each case. *Adi*.:Adipose, *Col*.:Colon, *Hea*.:Heart, *Kid*.:Kidney, *Liv*.:Liver, *Lun*.:Lung, *Ova*.:Ovary, *S*.*Mus*.:Skeletal Muscle, *Spl*.:Spleen, *Tes*.:Testes.

The Jaccard index was used to generate the similarity matrices comparing models extracted for different contexts, as well as models of the same context extracted by different methods. In this last case, the clustering dendrogram in Fig 1 was generated with the *hclust* function of the package *stats* in the R environment, and by using the average linkage criterion

We z-normalized the sum of Jaccard similarities of each context to the remaining (*i*.*e*., the sum of each column of the distance matrix). Therefore, the respective z-score quantifies the extent to which a given context differs from the remaining.

### Model agreement with human protein expression data

The protein expression profiles were taken from the Human Protein Atlas [38] where 10 out of the 11 contexts are represented (note that the hypothalamus is missing, so we did not include it in the evaluation; moreover, for the adipose tissue, we took the data from the cell type adipocyte). In the Human Protein atlas, protein expression levels are classified into *high*, *medium* or *low* and are derived by inmunohystochemical staining. Recon 1 uses Entrez gene identifiers, while the protein coding genes in the Human Protein Atlas are identified following the Ensembl convention. Hence, we mapped the Ensembl identifiers onto Recon 1 using the BioMart data mining tool from Ensembl [39].

## Results and Discussion

### Evaluation of RegrEx performance

As a case study, we applied RegrEx to extract 11 context-specific human models, namely, adipose tissue, colon, heart, hypothalamus, kidney, liver, lung, ovary, skeletal muscle, spleen and testes, and to obtain the corresponding flux distributions. The starting GEM was the Recon 1 reconstruction [32], which was reduced to its consistent part (*i*.*e*., blocked reactions, incapable of carrying flux in any feasible steady-state distributions, were removed). This pre-processing improves RegrEx performance since the existence of blocked reactions in the GEM would lead to stoichiometric inconsistencies. We used RNAseq expression profiles for 11 human tissues as context-specific data [35]. Although it has been shown that gene expression does not always represent a good proxy of the metabolic flux state [40,41], it still constitutes the best data source regarding coverage and quality (typically providing quantitative data for the great majority of genes in the GEM).

We compared RegrEx performance with other existing methods for context-specific model extraction and flux prediction. Namely, iMAT [14], the method proposed in Lee et al., 2012 (here called Lee2012) as well as a non-regularized version of RegrEx (RegrEx-λ_0_), as a special case of the RegrEx method with λ = 0. We also included FastCORE in our comparison; however, since FastCORE only provides a context-specific model (*i*.*e*. set of active reactions), we only included it in comparisons at the structural level of the extracted models. In each case, we used the consistent part of Recon 1 and the same gene expression data from the RNAseq Atlas to provide unbiased and fair comparison.

We would like to point out that Lee2012 was not originally developed to extract context-specific models. However, RegrEx has a form similar to that of Lee2012, which aims at improving flux prediction through minimizing the absolute distance between data (*e*.*g*., RNAseq expression profiles) and flux values. For this reason, we also included Lee2012 in the comparative analysis. Nevertheless, RegrEx differs from Lee2012 in the inclusion of regularization and also in the treatment of reversible reactions: Lee2012 applies an iterative approach, where the optimization problem starts first with the subset of irreversible reactions, and reversible reactions are then added sequentially by solving additional optimization problems. This last step is time consuming because it involves two optimization problems per reversible reaction. In contrast, RegrEx selects direction of reversible reactions at once through the use of a binary variable, as explained in the Methods section, thus reducing the computational time. Moreover, RegrEx is unbiased with respect to the order in which the reversible reactions are added, which is a shortcoming not resolved in Lee2012.

The performance analysis was divided into two parts: First, the similarity with the expression data used to extract the models was evaluated. This evaluation included two measures—the correlation between predicted fluxes and data values (except for FastCORE) and the level of agreement between the correlation matrix of the expression data for each context and the Jaccard distance matrix of the extracted models (to quantify the distance between two models in terms of the set of active reactions). Second, we performed an independent validation of the extracted models by measuring their level of agreement with protein expression data taken from the Human Protein Atlas [38].

### Main characteristics of extracted models by the evaluated methods

The general characteristics of the extracted models by each method are summarized in Table 2, and fully detailed in S1 Table. In terms of cardinality (*i*.*e*., the number of reactions included in an extracted model), Lee2012 generates models with the lowest mean cardinality (on average approximately 785 reactions per model) followed by RegrEx and RegrEx-λ_0_ (with, on average, approximately 843 and 1030 reactions per model, respectively). In contrast, FastCORE and iMAT result in markedly bigger models for the corresponding contexts (with, on average, approximately 1358 and 1411 reactions per model, respectively). Each set of context-specific models extracted by a particular method has a core set of reactions shared by all contexts. In addition, each context has an exclusive set of reactions (*i*.*e*., reactions that are only present in the examined context). In this sense, RegrEx extracted models have the smallest set of shared reactions, with 299 reactions, and the biggest set of total exclusive reactions (*i*.*e*., exclusive reactions over all context), with 332 reactions. These two properties demonstrate that the models extracted by RegrEx are in fact more context-specific than the ones extracted by the other methods, which is confirmed by the mean Jaccard similarity between models. The Jaccard similarity is lowest in the case of RegrEx ($\bar{\text{I}}$_JRegrEx_ = 0.56, with a standard deviation, σ_IJRegrEx_ = 0.04) in support of the previous claim. On the contrary, Lee2012 generates the greatest core set among the methods evaluated, with 509 shared reactions, and the smallest set of total exclusive reactions, amounting to 140 reactions, This, in turn, makes the Lee2012 models to be the least context-specific ($\bar{\text{I}}$_JLee2012_ = 0.77, with a standard deviation, σ_ILee2012_ = 0.01).

**Table 2:** Comparison of models extracted by the four evaluated contending methods: Mean values across contexts. Global characteristics of the models are derived by applying RegrEx (with automated determination of λ), RegrEx-λ_0_ (*i*.*e*., RegrEx without regularization), Lee2012, iMAT and FastCORE. The abbreviations stand for the following: *$\overset{-}{Card.}$* denotes mean cardinality, $\overset{-}{O}$_*R*_, mean data-orphan ratio,$\;\overset{-}{\rho }$_*(V*,*D)*_, mean correlation between data and predicted flux values, $\overset{-}{R}$_*(V*,*D)*_, mean residual value between fluxes and data $,\;\overset{-}{I}$_*J*_, mean Jaccard index to any other context, *Shared*, number of shared reactions across all contexts, and *Total Exclusive* represents total number of exclusively context-specific reactions across all contexts. Values in round brackets correspond to the standard deviation.

	$\bar{Card.}$	_$\bar{O}$ R_	_$\bar{\rho }$(V,D)_	_$\bar{R}$(V,D)_	_$\bar{I}$ J_	Shared	Total Exclusive	Total Card
RegrEx	842,91(55.14)	0,34(0.05)	0,42(0.07)	0,29(0.08)	0,56(0.04)	299	332	1618
RegrExλ_0_	1030,30(76.32)	0,47(0.04)	0,38(0.08)	0,28(0.08)	0,65(0.03)	490	239	1711
Lee2012	784,6 (26.53)	0,28(0.03)	0,13 (0.05)	-	0,77 (0.01)	509	140	1092
FastCORE	1357,9(39.03)	0,50(0.04)	-	-	0,61(0.05)	503	230	2232
iMAT	1411(41.62)	0,42(0.04)	-0.17(0.03)	-	0,65(0.04)	611	210	2205

When extracting a context-specific model, there exists, in general, a subset of reactions of the original (unspecific) GEM whose fluxes are unbounded by data. This can be due to the absence of GPR rules (either because the reaction is not enzyme catalyzed or because the gene-protein association has not been annotated), or simply because experimental data is missing for that reaction. In any case, it is of interest to minimize the number of included reactions without associated data, here called *data-orphan* reactions, since their inclusion results in uncertainty (given the available data). On this line, we evaluated the aforementioned property by computing the *data-orphan* ratio (*i*.*e*., the ratio between the number of incorporated reactions with non-associated and that with associated experimental data) of each of the extracted models. RegrEx extracted models show the second lowest mean *data-orphan* ratio across all methods (O_RRegrEx_ = 0.34, with a standard deviation, σ_RegrEx_ = 0.05), only surpassed by Lee2012 (O_RLee2012_ = 0.28, with a standard deviation, σ_Lee2012_ = 0.03), Notably, this is only valid when a regularized extraction is used, since in the case of RegrEx-λ_0_, the *data-orphan* ratio ranks to the second worst position, only surpassed by FastCORE, with mean orphan ratios of 0.47 and 0.50, respectively. The reduced *data-orphan* ratio indicates that RegrEx, although surpassed by Lee2012, is still capable of extracting compact models in which the number of data-orphan (uncertain) reactions is minimized.

Regarding the set of represented reactions of Recon 1 across all contexts, here called total cardinality, RegrEx models collect 1618 unique reactions out of the total of 2469 reactions in Recon 1. Therefore, RegrEx models rank in an intermediate position between Lee2012 models (with 1092 total unique reactions) and iMAT and FastCORE (with 2205 and 2232 total reactions, respectively). The differences in total cardinality as well as mean cardinality per model can be explained by the two main objectives that the evaluated methods take; namely, minimizing the distance between data and flux values, like in the case of Lee2012 and RegrEx(-λ_0_), or including the entirety or a majority of a predefined core set for a particular context, like in FastCORE and iMAT, respectively, which here was the same for both methods (see the Methods section).

Indeed, this last grouping was reflected when we compared the similarity between models of the same context extracted by the different methods. Results were similar across all contexts, iMAT and FastCORE-derived models share many reactions, as indicated by the high Jaccard similarity index (mean value across contexts,$\bar{\text{I}}$_JiiMAT/FastCORE_ = 0.81, with σ_IiiMAT/FastCORE_ = 0.02) and are thus grouped together in the corresponding dendrogram of Fig 1. On the other hand, RegrEx, RegrEx-λ_0_ and Lee2012 form another cluster, where models extracted by RegrEx and RegrEx-λ_0_ are grouped together, and the ones extracted by Lee2012 are closer to RegrEx-λ_0_.

### Similarity to data evaluation

When inspecting the correlation between data values and predicted fluxes, RegrEx obtained the first position in the ranking (mean correlation, $\bar{\rho }$_RegrEx_ = 0.42, σ_ρRegrEx_ = 0.07), followed by RegrEx-λ_0_ and Lee2012 (with a mean correlation of 0.38 and 0.13, respectively). Moreover, iMAT results in the worst mean correlation value of -0.17 (FastCORE does not provide flux values, as commented before, so it is not evaluated with respect to this criterion). However, this difference in correlation can be explained by the different approach followed by iMAT, since in this case the method does not aim at minimizing the distance between data and flux values.

To include FastCORE in the comparative analysis, we next inspected the similarity to data in a different manner: instead of considering the flux values, we now compare the set of active reactions per context. This criterion captures an aspect of the structure of the extracted metabolic networks. We used the sets of active reactions across different contexts and per method to compute the similarity matrix, using the Jaccard index. We then compared this similarity matrix with the corresponding correlation matrix of the gene expression values for all contexts by using the Rv coefficient. The level of agreement between these two matrices was indeed high for RegrEx, iMAT and FastCORE, as supported by Rv coefficient values of 0.9, 0.92 and 0.93, respectively; the level of agreement was markedly lower for RegrEx-λ_0_ (Rv = 0.76) and Lee2012 (Rv = 0.69). This result support two claims: Firstly, although FastCORE does not provide fluxes and iMAT results in very poor correlation between flux and data values, they are still capable of capturing the general differences between contexts collected in the expression data. Secondly, RegrEx is also capable of capturing this differences but with a lower mean cardinality per extracted model. In other words, even with fewer reactions on average, the models extracted by RegrEx are still able to capture the main differences in active reactions contained in the gene expression data. This fact summarizes very well the purpose of regularization when learning models from experimental observations: remove variables (*i*.*e*. reactions) that are not important to explain the observations.

The previously reported values of the Rv coefficient quantify the overall agreement between both the similarity and correlation matrices. We next decomposed this result for each different context. To this end, we computed the z-scores (as detailed in Methods) for both matrices. In this sense, RegrEx performed better than iMAT and FastCORE in capturing the pattern showed by gene expression, as quantified by a correlation value of 0.92, between the z-score values of the extracted models and the ones of data, against a value of 0.88 and 0.89 for iMAT and FastCORE, respectively, see Fig 1. The better agreement in the case of RegrEx can be observed in the number of mismatches between the sign of the data z-score value and the one of the extracted model. More specifically, RegrEx only fails in the case of the ovary model, which lies under the mean similarity between any two of the extracted models, thus having a negative z-score, while the corresponding expression data lies over the mean. However, iMAT and FastCORE commit three mismatches (skeletal muscle, kidney and colon in FastCORE and skeletal muscle, adipose tissue and hypothalamus in FastCORE). As expected, the performance of RegrEx-λ_0_ and Lee2012 is again worse than the other methods, with a correlation value of 0.66 and 0.57, respectively. Interestingly, liver appears to be the most different context in terms of active reactions, and this is captured by all methods except for RegrEx-λ_0_. This is not surprising, since liver is well known to be the organ with greater metabolic capabilities.

To conclude this section, it is noteworthy to highlight the comparison of RegrEx performance with the one of its non-regularized version, RegrEx-λ_0_, since the only difference between the two approaches is the application of regularization and the inclusion of an optimization step to determine the optimal λ-value. Precisely the consideration of regularization allows RegrEx not only to extract more compact models, as commented before, but also to increase the correlation between data and flux values, reduce the data-orphan ratio, and greatly improve the general similarities and differences in the metabolic state of the different contexts, as showed by the RV coefficient and z-score values.

### Models evaluation by human protein profiles

We performed and independent test on the biological reliability of the extracted models by all evaluated methods. To this end, we compared the level of agreement of each model with protein expression profiles taken from the Human Protein Atlas [38] (we had to exclude hypothalamus from the comparison since it is missing in this database, see Methods). The protein expression data is semi-quantitative, namely, it only provides the categorical levels *high*, *medium* and *low*. To account for this, we evaluated whether models contained an enriched group of genes coding for proteins within the category of *high* expressed for the corresponding organ/tissue in comparison with the other two categories, as well as and an enrichment in genes from the *medium* expression value group in comparison to those from the *low* expression.

To tests these hypotheses, we determined the number of genes of each group in each context, *i*.*e*., the number of genes in *high*, *medium* and *low* across all organs/tissues, and applied the Mann-Whitmann-Wilconxon test on the obtained distributions to determine the statistical significance of their difference. This test was applied for each evaluated method. As observed in Table 3, RegrEx extracted models are indeed significantly enriched in *high* and *medium* expressed genes since the p-values for all three comparisons, number of genes in the *high* group greater than in the *medium*, H>M, *medium* greater than *low*, M>L, and *high* greater than *low*, H>L, are below the significance threshold of 0.05. In the case of RegrEx-λ_0_ and iMAT, only two of the comparisons are significant, H>M and H>L, and M>L and H>L, respectively. Models in FastCORE are only enriched in *high* expressed genes in comparison to *low* expressed and none of the comparisons are significant in the case of Lee2012. These results add and additional experimental support to the extracted models by RegrEx and in a lesser extend the ones by RegrEx-λ_0_, iMAT and FastCORE, for two main reasons: the additional experimental data comes from a different (independent) database than the one used during the extraction, and relies on a lower hierarchical level in the causal chain controlling metabolic fluxes, namely, is based on protein expression rather than gene expression.

**Table 3:** Comparison on the level of agreement of each extracted model with the Human Protein Database. P-values for each Mann-Whitman-Wilconxon test (with alternative hypothesis H>M, M>L and H>L) are collected here. A significance threshold of 0.05 was used to reject the null hypothesis (p-values<0.05 in bold).

Method	H>M	M>L	H>L
RegrEx	**0.0445**	**0.0262**	**0.0034**
RegrExL0	**0.0045**	0.1763	**0.0006**
iMAT	0.1399	**0.0444**	**0.0319**
Lee2012	0.3421	0.4559	0.2179
FastCORE	0.0525	0.1575	**0.0216**

### Functional analysis of RegrEx extracted models

The next step in evaluating RegrEx extracted models was to perform a functional analysis by determining which metabolic functions were important in each context. To this end, we calculated the flux capacity of every reaction in the models, given by the difference between the maximum and minimum corresponding flux value obtained per Flux Variability Analysis [42] (in this case the only constraints where arbitrary upper and lower bounds for flux values to prevent optimizing on an unbounded flux cone and respect the thermodynamic constraints). We stress that the flux capacity value herein defined only quantifies the theoretical range of flux values that a reaction can take in a given network; hence the actual flux range of a reaction in a particular metabolic scenario does not have to coincide with the theoretical. However, the flux capacity of a reaction does depend on the topology of an extracted metabolic network, thus it is a reliable proxy to evaluate which reactions are favored in a certain context. In an alternative way, it also allows evaluating which reactions are not influenced by the differences in network topology across contexts, *i*.*e*., which are robust in flux capacity irrespective of the different contexts (note that this set of robust reactions must belong to the core set of shared reactions across contexts).

To facilitate the analysis, we grouped the subsystems into 8 broader metabolic categories: Central metabolism, Amino-acid metabolism, Carbohydrate metabolism, Cofactor and vitamin metabolism, Lipid metabolism, Nucleotide metabolism, Transport and Others, see S2 Table. In addition, we averaged flux capacity values of the reactions in each metabolic subsystem of Recon 1 and in each previously defined metabolic category, to obtain the mean flux capacity (MFC) per subsystem or category. We also counted the total number of reactions (TNR) in each category as an alternative way of quantifying their metabolic importance in the extracted networks.

**Fig 2:**
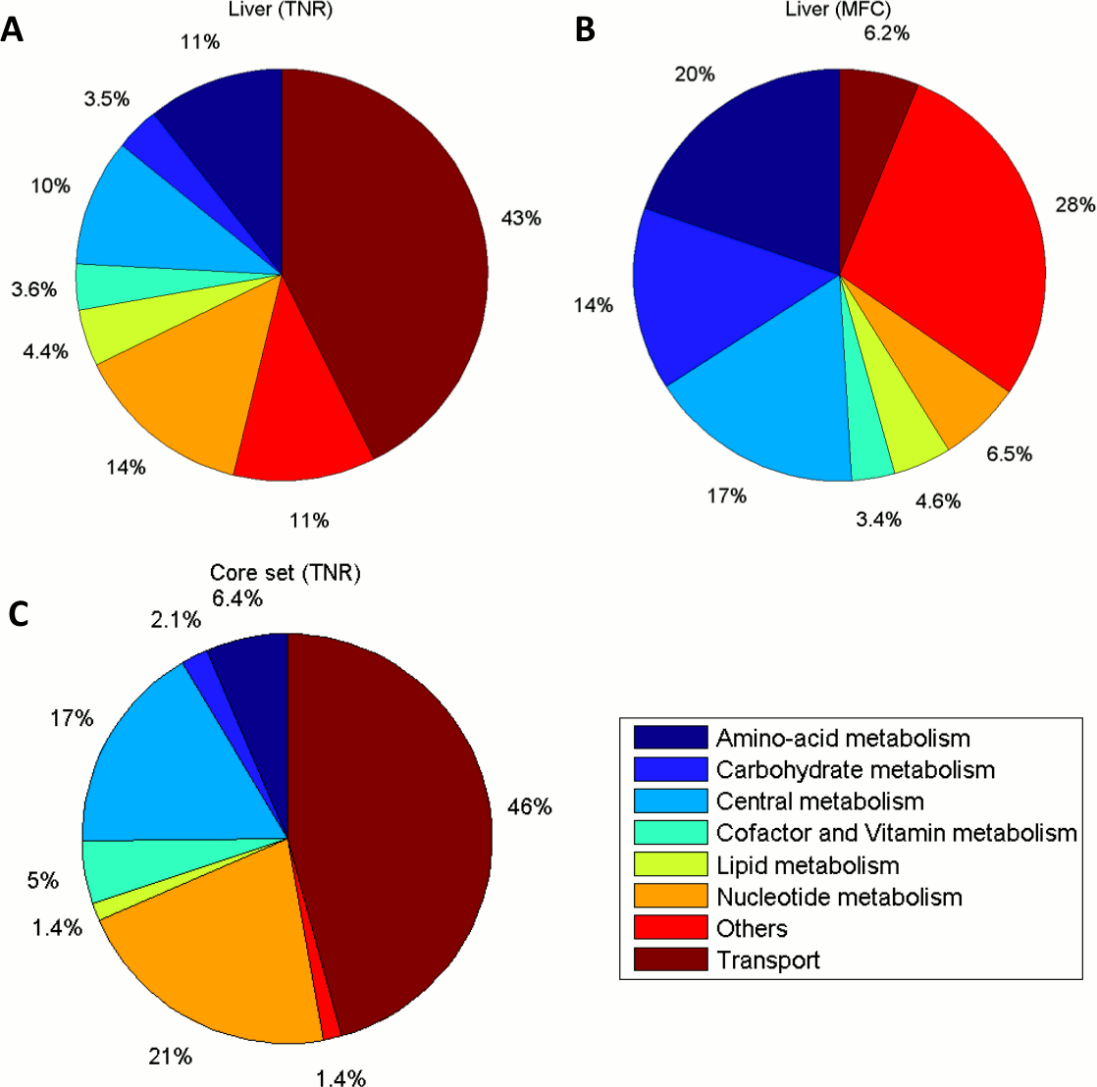
Pie charts displaying the core set of shared reactions for the 11 models extracted by RegrEx and a selected comparison of the metabolic categories presented in the liver model. (A) Distribution of the total number of reactions (TNR) per metabolic category for the liver model (containing a total of 821 reactions across all categories). (B) Distribution of the mean flux capacity (MFC) values of each metabolic category in the liver model, as explained in the main text, noticeable differences arise with respect to the distribution depict in A. (C) Reaction content (TNR) for the core set of shared reactions divided per metabolic category, as explained in the main text, the three dominant categories are Transport, Central and Nucleotide metabolism. Metabolic category names are displayed in the color bar legend.

Marked differences arose when we compared the results obtained by counting the number of reactions per metabolic category with the ones obtained by averaging the flux capacity. For instance, the biggest one in terms of the number of reactions in all extracted models (using any of the methods) is Transport, similar to the findings in [34]. However, if we look at the MFC, Transport, in general, takes a modest position, often surpassed by Central and Carbohydrate metabolism (the miscellaneous group “Others” get the highest MFC in all extracted models). More specifically, approximately 43% of the reactions in Liver are assigned to Transport, whereas Transport only contributes with 6.2% to the total MFC of the extracted model. Similarly, Nucleotide metabolism is associated 14% of the total number of reactions while only contributes with 6.5% to the total MFC of Liver. On the contrary, systems with a smaller number of reactions, such as Amino acid and Carbohydrate metabolism (11% and 3.5%, respectively), get a higher contribution to the total MFC (20% and 14%, respectively), see Fig 2 and S1 Fig for a complete comparison for each context.

As commented before, all context-specific models share a core set of 299 reactions in the case of RegrEx extracted models. If we consider the TNR in each metabolic category we obtain a distribution like the one displayed in Fig 2. The core is dominated by Transport reactions (46%), followed by Nucleotide metabolism (21%) and Central metabolism (17%), being the rest of the categories represented to a smaller extent. Moreover, when computing the MFC for each individual reaction in the core, we see that the majority of them (80%) are, in fact, robust reactions, which means that the flux capacity is maintained across contexts. However, a non-negligible part of the core (the remaining 20%) is constituted by non-robust reactions, those that, although being shared by all contexts, present a variable flux capacity. In this group we encounter reactions like the superoxide dismutase (ROS detoxification), with a coefficient of variation (CV) value of 0.49, one of the highest in the core. Interestingly, we also find all the reactions belonging to the pentose phosphate pathway that are present in the core, see S3 Table. These observations show that not all reactions in the core behave in a similar way. On the contrary, we can partition it into a subset of reactions that are independent of (context-specific) modifications of the network topology, and a subset that depends on the context and therefore can be more or less prominent in certain tissues or organs.

**Fig 3:**
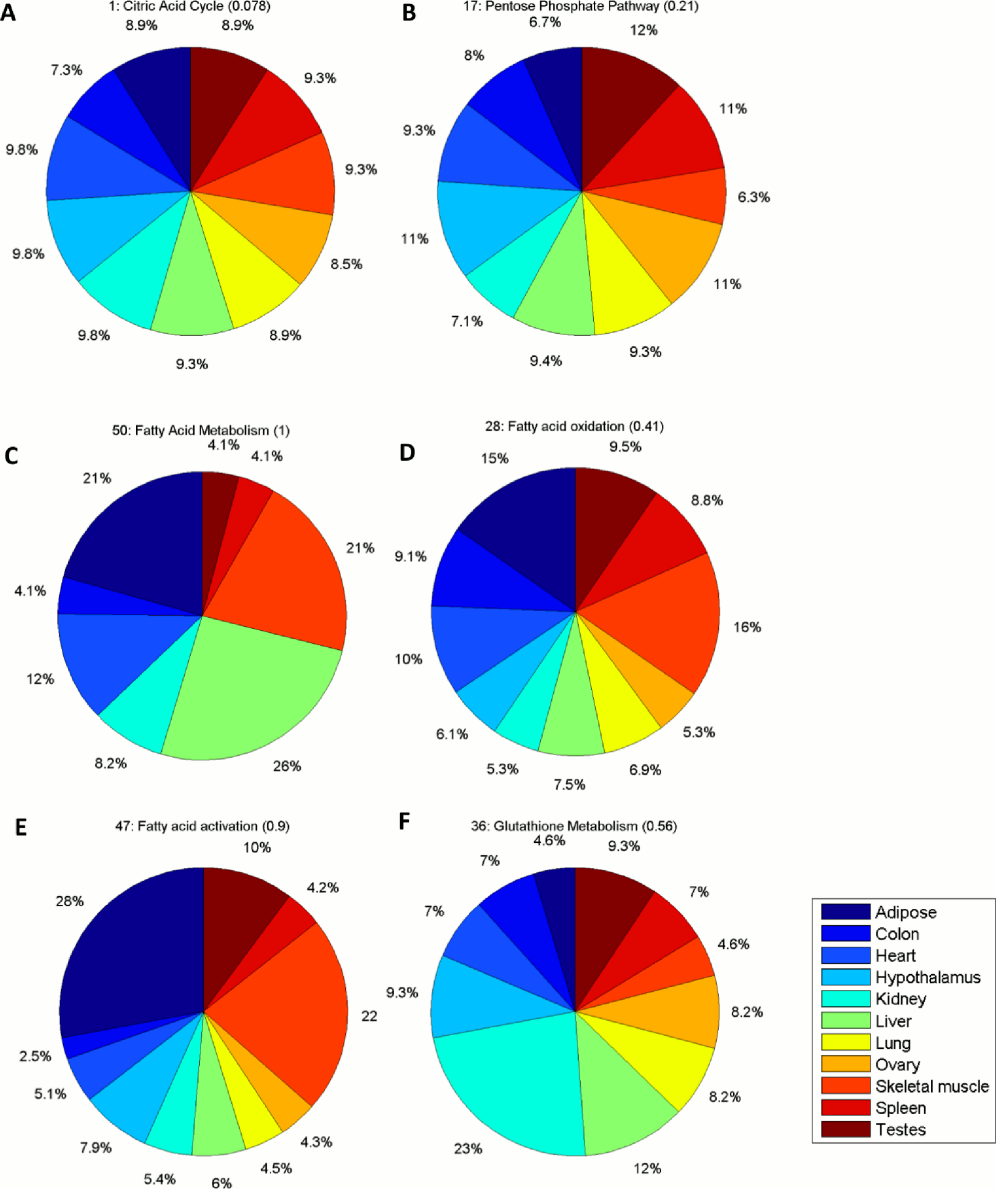
Illustration of selected Recon 1 subsystems displayed in a Pie chart form, depicting the distribution of mean flux capacity (MFC) values across contexts. **Panels** A-B correspond to the two extreme metabolic subsystems, in terms of CV, in Central metabolism. The citric acid cycle (A) shows the lowest CV value (both in Central metabolism and within the entirety of Recon 1 subsystems). The pentose phosphate pathway (B) shows the greatest CV value in Central metabolism. (C-E) the distribution of MFC values is shown for fatty acid metabolism (C) which is predominantly represented in liver, adipose tissue and skeletal muscle, fatty acid oxidation (D) and fatty acid activation (E) both subsystems predominant in adipose tissue and skeletal muscle. (F) The MFC distribution across contexts is depicted for gluthatione metabolism. Kidney is the context where this subsystem gets a highest MFC value, constituting a 23% of the total MFC value across contexts. See main text for details. In all cases, the first number preceding the name of the subsystem corresponds to its position in the ranking generated by the CV values, which are shown in round brackets here. Context names are displayed in the color bar legend.

Alternatively, to further evaluate the functional validity of the RegrEx extracted models, we used the previously calculated MFC to investigate the importance that a given subsystem had in each context. Furthermore, we ranked the subsystems according to the CV of the MFC value distribution of each subsystem across contexts. This implies that subsystems with a low CV are evenly represented among the different contexts, while with increasing CV, these subsystems tend to be more specific for certain contexts. For instance, all subsystems belonging to Central metabolism occupy top positions in the ranking, which is expected due to the fundamental role that these subsystems play in all cell types. The citric acid cycle is the first subsystem in the ranking with a CV value of 0.078. On the contrary, the pentose phosphate pathway is the subsystem in Central metabolism with a highest CV value of 0.21. However it can be considered low in the context of the entire ranking, and may be explained by the fact that, unlike the rest of subsystems in Central metabolism, the totality of its reactions in the core are non-robust, as mentioned before, see Fig 3 and S4 Table. In addition to these subsystems, we also find in top positions others equally fundamental pathways, including: NAD, folate and vitamin A metabolism (all in the category of Cofactor and Vitamin metabolism), extracellular and mitochondrial transport or nucleotides metabolism. Interestingly, the last three subsystems are also the ones containing the greatest number of the previously defined robust reactions (S4 Table).

Lipid metabolism presents a middle level of specialization across the different contexts occupying middle positions in the ranking. Therefore, this finding implies that there are some tissues in which lipid metabolism is predominant. For instance, fatty acid metabolism is predominant in adipose tissue, liver and skeletal muscle. In addition, fatty acid oxidation and fatty acid activation are predominant in adipose tissue and skeletal muscle. This is consistent with known functions of these contexts, since the adipose tissue and liver are primary locations for fatty acid metabolism and the fatty acid oxidation provides the required energy supply for oxidative muscle contraction [43]. We also find gluthatione metabolism (assigned to the category “Others”) in a middle position, with a CV of 0.6, and it is highlighted in kidney. This last feature also serves as validation of the extracted model since it is well known that glutathione metabolism is essential in the kidney for an adequate functioning [44]. Finally, the lasts positions are mainly populated by subsystems in Cofactor and Vitamin metabolism and the miscellaneous category “Others”, such as keratin sulfate degradation, heme biosynthesis and degradation or bile acid biosynthesis, see S4 Table and Fig 3

**Fig 4:**
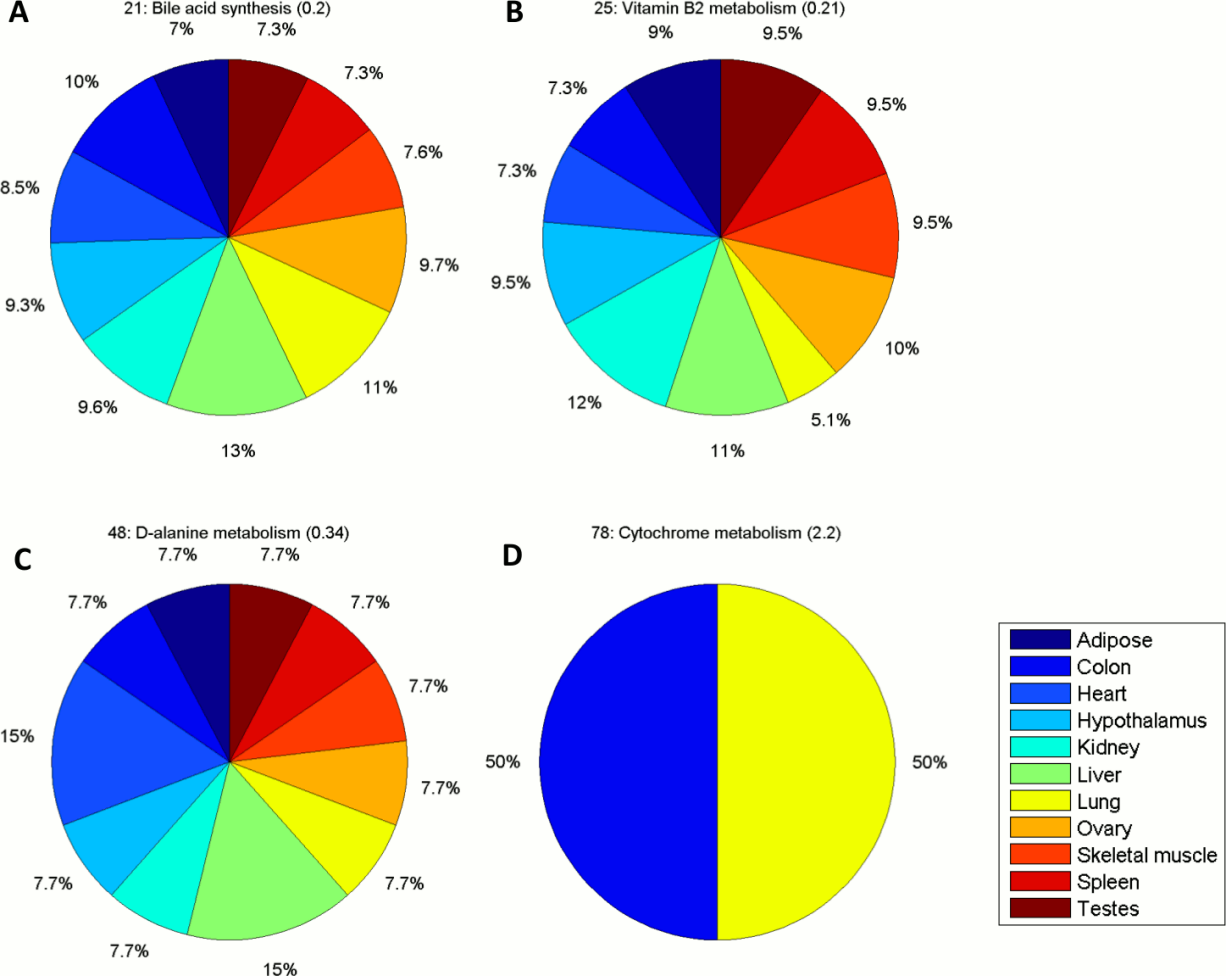
MFC value distribution across contexts for selected subsystems in Recon 2. Bile acid (bio)synthesis (A), vitamin B2 metabolism (B, equivalent to riboflavin metabolism in Recon 1) and D-alanine metabolism (C) are represented in all contexts in Recon 2. Cytochrome metabolism (D, equivalent to CYP in Recon 1) is represented only in two contexts, Lung and Colon, in Recon 2. In all cases, the first number preceding the name of the subsystem corresponds to its position in the ranking generated by the CV values, which are shown in round brackets here. Context names are displayed in the color bar aside. See main text for details.

The subsystems with largest CV value include those with extreme behavior, *i*.*e*., these subsystems are only predicted to be active in a single context. This category consists of bile acid biosynthesis, biotin, riboflavin, vitamin B6, vitamin D, CYP, methionine and D-alanine metabolism (S4 Table). We can explain this behavior as a reflection of the original gene expression values associated to the reactions in each of these subsystems. For instance, in the case of bile acid biosynthesis, if we look at the distribution of the total expression values in the subsystem across contexts, the liver presents an extreme value (z-score = 2.9, S5 Table). We wanted to know if this characteristic of the data used was sufficient to explain the artifact or if the network topology of Recon 1 was also contributing to this observation. The latter may happen if Recon 1 lacks some reactions that are crucial to satisfying the steady-state conditions while including reactions of the bile acid biosynthesis in other contexts. To test this hypothesis, we applied RegrEx on Recon 2, a recent extended version of the Recon 1 model of increased size (*i*.*e*., 5317 in Recon 2 *versus* 2469 reactions in Recon 1, both after eliminating the blocked reactions) [34]. After extracting the context-specific models, and ranking the subsystems by the CV value of the MFC, we found that the majority of these subsystems were now represented in more than one context. For instance, bile acid biosynthesis (named bile acid synthesis in Recon 2) is present in all contexts, but has a greatest MFC value in Liver, see Fig 4 and S6 Table.

### Computation time comparison

RegrEx computational performance was also evaluated, and compared to the other methods. In all cases, with the exception of FastCORE, the Gurobi [30] solver was used. CPLEX [45] was used in FastCORE instead of Gurobi for two reasons: CPLEX is the default solver in the code provided in [37], and we believe that the differences in computation time between Gurobi and CPLEX are negligible when solving LP problems, as is the case in FastCORE.

Table 4 summarizes the differences in computational time and problem formulation for the evaluated methods. Markedly, iMAT shows a very good computation time, with a mean of 0.16 seconds per model extracted (this result changes dramatically when using GLPK as solver [46], where mean network extraction time is typically above one minute). This computation time is comparable to the one obtained by FastCORE. Lee2012 presents a computation time of around 10 minutes per extraction (this result again changes dramatically when using GLPK as solver, with computation times of several hours per context extracted). When RegrEx is evaluated for a fixed λ-value (that is, RegrEx-λ_0_), the computation is fixed to around 60 seconds per model extracted, this is due to the time limitation constraint imposed to the Gurobi solver, as discussed in Methods. When no time limit is imposed RegrEx gets the worst position among the evaluated methods, this may be due to the complexity inherent of solving an MIQP for a big network like the one of Recon 1. However, the sensitivity to time limit analysis suggests that the improvement obtained by increasing the time limit does not worth the extra time expended, even could be reduced to 30 seconds with a similar outcome (see Table 1 in Methods).

**Table 4:** Computation time of the evaluated methods. Mean computation times per model extraction, type of mathematical program solved and the used commercial solver are displayed for each evaluated method.

Method	Formulation	Solver	Mean Time ± SEM *
**iMAT**	MILP	Gurobi	0.1652 ± 0.0038
**FastCORE**	LP ⟳	CPLEX	0.2976 ± 0.0101
**RegrEx-λ0**	MIQP	Gurobi	60.0785 ± 0.0025
**Lee2012**	LP ⟳	Gurobi	571.2108 ± 24.8921
**RegrEx**	MIQP ⟳	Gurobi	928.5313 ± 2.3079

SEM stands for Standard Error of the Mean.

^⟳^ stands for iteratively repeated.

*Time is shown in seconds.

Finally, in the case of RegrEx, the total computation time depends on the number of λ-values evaluated during the optimization step. For instance, in this case the mean computation time per model extraction stays around 15 minutes, since a sequence of 15 λ-values is used in the optimization. This greater computational time required by RegrEx is explained by the necessity of finding an optimal λ-value to control the regularization during the extraction, which is specific to any particular data set and GEM. However, the total computational time spent by RegrEx still remains within a reasonable range, and, as seen in Results, including regularization is fundamental to increase the overall performance.

## Conclusion

We have presented RegrEx, a method to extract context-specific metabolic models and provide a flux distribution most in accordance with experimental data. RegrEx have generated context-specific flux distributions with the highest correlation values among the competing methods evaluated, as well as extracted compact models, enriched in reactions with high associated data values. Importantly, RegrEx performance is severely impaired when performing a non-regularized extraction (*i*.*e*., when λ = 0, here called RegrEx-λ_0_). More specifically, the models obtained *without* employing regularization are less specific to each particular context, share a greater amount of reactions and contain less exclusive reactions in comparison to models for other contexts. This is supported by the higher mean Jaccard index over all pairs of compared context-specific models. In addition, the mean orphan ratio is higher if regularization is not used, implying that a greater number of reactions with non-associated experimental data is included and causing these models to be less compact. Finally the mean correlation values between predicted fluxes and data are also smaller in the non-regularized extraction. Altogether, these observations support the importance of including regularization to obtain a better performance in context-specific model extraction.

RegrEx have also proven to be a suitable method among the alternatives evaluated here, to provide a larger correlation between predicted fluxes and experimental data, as well as models that capture the general pattern of differences and similarities in reaction activity across contexts expressed by data. The models extracted by RegrEx are also in agreement with an independent data source, based on protein expression, and include preferentially genes that are associated to highly expressed proteins, outperforming the competing methods with respect to this criterion.

In the case study presented here, we have used gene expression profiles as experimental data. However, RegrEx can support other data sources; protein profiles can be easily integrated, (*e*.*g*. generated through mass-spectrometry based approaches), and the problem of lower coverage typically presented by protein profiles can be alleviated by jointly integrating gene expression data to fill the gaps with respect to data support. In addition, when there exist strong experimental evidence supporting the presence of a certain reaction in a given context, its lower bound can be set to an arbitrary positive value (*i*.*e*., V_min_>ϵ, when splitting reversible reactions) thus forcing it to be included in the context-specific model. In a similar manner, when the evidence is for the presence of a metabolite, the sum of the reactions producing such metabolite could be constrained to ensure its inclusion, thus allowing integrating metabolomics data in a qualitative way.

RegrEx can be easily used in MATLAB through the provided files. Moreover, no parameters need to be chosen by the user, since the only parameter, λ, is determined by RegrEx in an automated fashion. In this manner, the user only needs to provide a relevant (context-specific) data source(s) and the GEM from where the context-specific model is to be extracted, and the rest of the operating process is fully automated. Finally, RegrEx does not require any *a priori* knowledge on metabolic functionality in a given context. The property of being an unbiased method along with the fully automation of the process may be a prominent quality when dealing with complex, multicellular organisms, where multiple cell types or tissues coexist and specialized in certain functions that are not yet very well understood and in the case where similarity to data (*e*.*g*., in the sense of correlations) are deemed appropriate by the experimentalists.

## Supporting Information

S1 Fig Comparison of metabolic category distributions across contexts. The importance of each metabolic category in each contexts is quantified using the total number of reactions (TNR) belonging to it and their mean flux capacity (MFC).
(PDF)

S1 File RegrEx source code, context-specific models and Data. The RegrEx code is provided for MATLAB. The 11 context-specific models are provided in the COBRA format (MATLAB structure), both for Recon 1 and Recon 2. The data set used in both cases is also provided.
(ZIP)

S2 File Pie charts displaying the MFC distribution for each metabolic category in Recon 1 and Recon 2 for the 11 context-specific models extracted by RegrEx. (ZIP)

S1 Table Comparison of models extracted by the four evaluated contending methods: Detailed values for each context. Global characteristics of the models derived by RegrEx (with automated determination of λ), RegrEx-λ0 (*i*.*e*., RegrEx without regularization), Lee2012, FastCORE and iMAT are collected in tables A, B, C, D and E, respectively. The abbreviations stand for the following: Card, denotes cardinality, #ExRxns., number of exclusive reactions, #Genes, number of genes, OrphRatio, data-orphan ratio, Cor(V,D), correlation between data and flux values, Res, mean residual value between fluxes and data, IJaccard, mean Jaccard index to any other context (values in brackets correspond to the standard deviation), and Lambda, represents the obtained optimum λ-value.
(XLSX)

S2 Table Metabolic categories of Recon 1. All metabolic subsystems from Recon1 present in the 11 RegrEx models were grouped in 8 broader categories, which are depicted in this table. This classification is based on the KEGG orthology for *Homo sapiens*. The miscellaneous group “Others” contains subsystems of several different categories in the original KEGG classification. Metabolic categories are highlighted in bold.
(XLSX)

S3 Table Core set of share reactions across contexts. The 299 reactions of the shared core set are displayed along with the subsystem and metabolic category to which they belong, the mean flux capacity (MFC) of each reaction across contexts and the CV value of the previous MFC. The core set is partitioned into the robust subset, composed of reactions with the same flux capacity in all contexts, *i*.*e*., with CV = 0, and the non-robust subset, which is composed by reactions with CV≠0. In this latter case, the reactions are ranked in descending CV value. Reactions highlighted in red in the non-robust subset belong to the pentose phosphate pathway.
(XLSX)

S4 Table Ranked list of Recon 1 metabolic subsystems represented in the 11 RegrEx derived models. In this table, the subsystems are ranked in ascending order in terms of their CV value. The Recon 1 subsystem name is displayed along with the metabolic category to which each subsystem belongs, the mean flux capacity (MFC), the number of robust reactions (#RobustRxns), *i*.*e*., reactions whose MFC value does not change across contexts, and the total number of reactions (#Rxns) are displayed for each subsystem. Subsystems presented in Fig 3 are highlighted in purple (A-B), orange (C-E) and green (F); subsytems that are represented in a single context are highlighted in red; subsystems with the highest number of robust reactions are highlighted in brown.
(XLSX)

S5 Table Associated gene expression data for subsystems present only in one context. Z-score values of the associated expression data are displayed for the 8 subsystems with unique representation across contexts. The z-scores represent the deviations from the mean of the total sum of data values in each subsystem across contexts. The values highlighted in red correspond to the contexts where RegrEx uniquely assigns each subsystem. In all cases, these contexts present an extreme total sum of data values, as reflected in the highest z-score value as compared with the rest of the contexts.
(XLSX)

S6 Table Ranked subsystems of Recon 2. All metabolic subsystems of Recon 2 represented in the 11 contexts are ranked according to their (increasing) CV value of the MFC across contexts. Subsystems highlighted in red correspond to subsystems of Recon 1 that were assigned by RegrEx to a single context.
(XLSX)
