# Supplementary figures and images for: Context-Specific Metabolic Model Extraction Based on Regularized Least Squares Optimization (part 1 of 2)
Source: PLoS One. 2015 Jul 9;10(7):e0131875. doi: 10.1371/journal.pone.0131875 (PMC4497637; doi:10.1371/journal.pone.0131875)

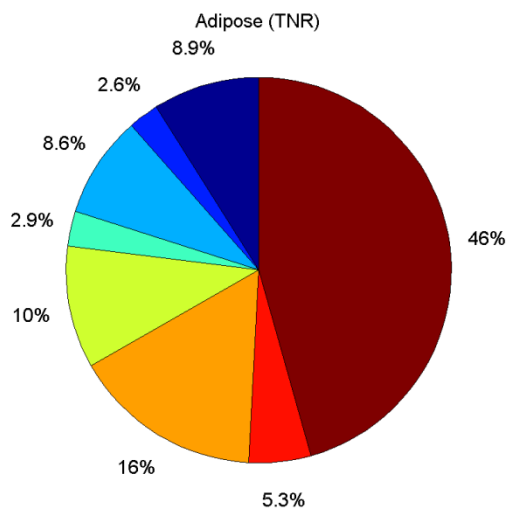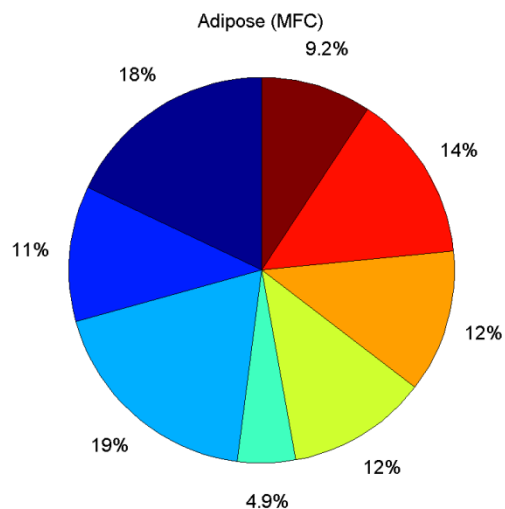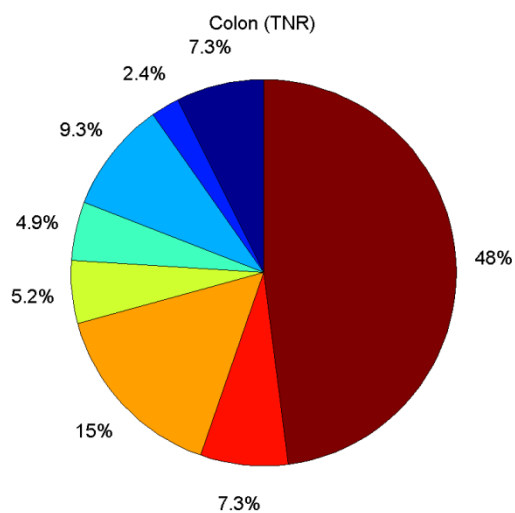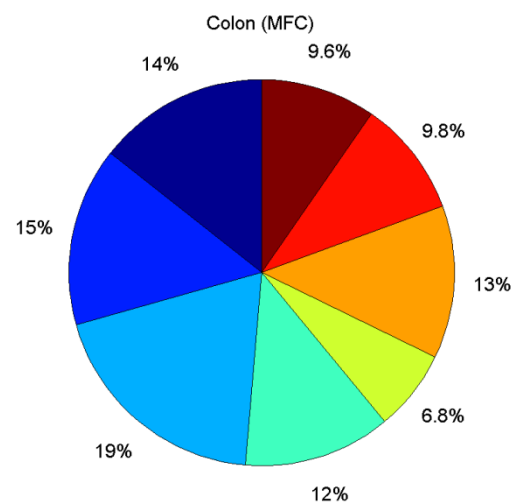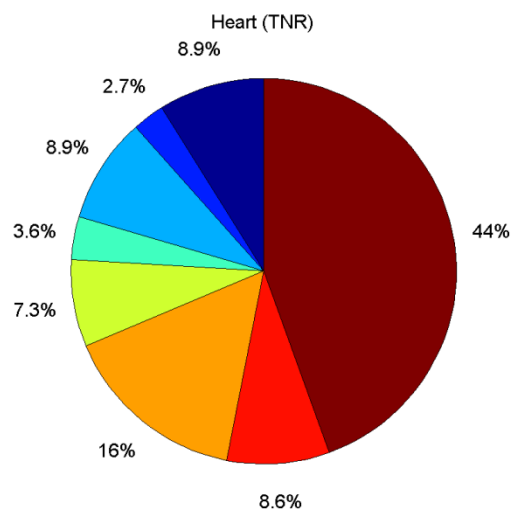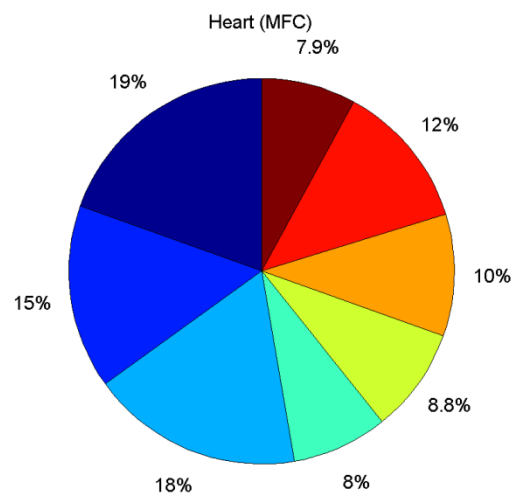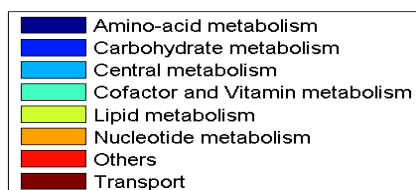

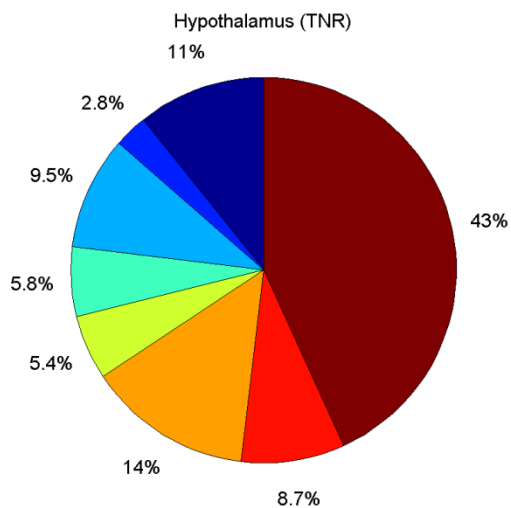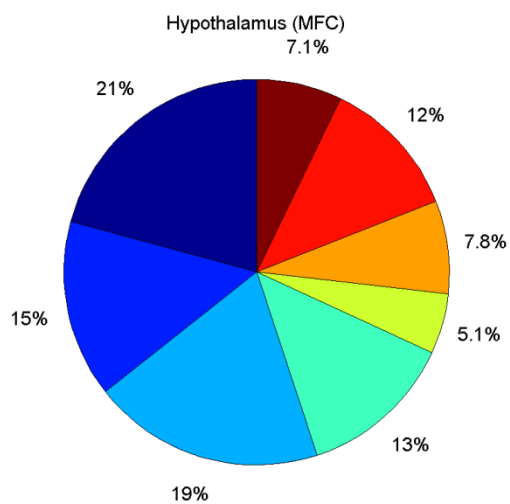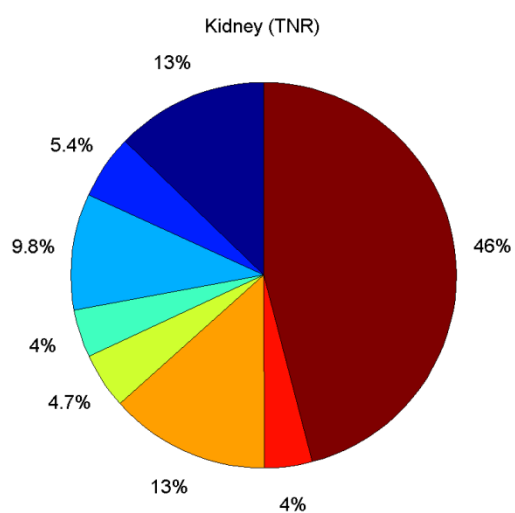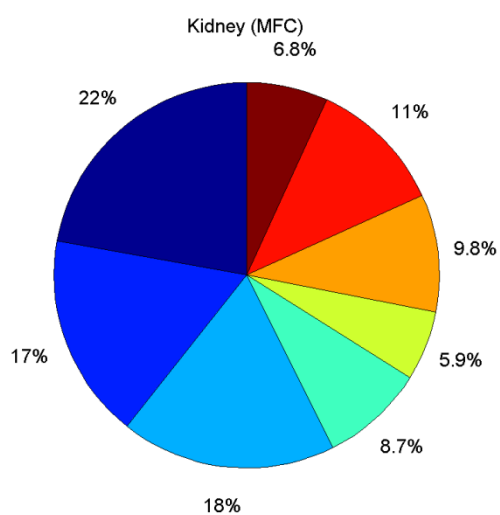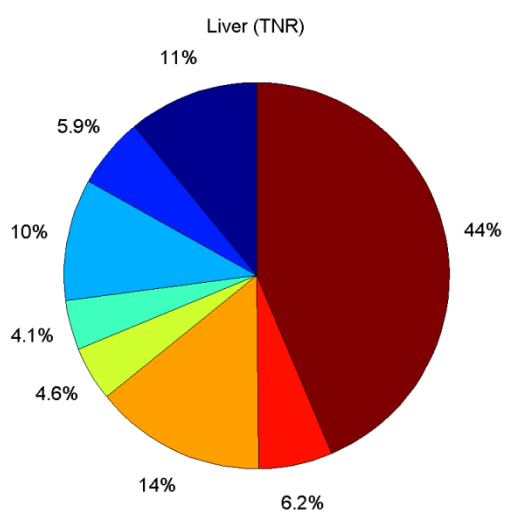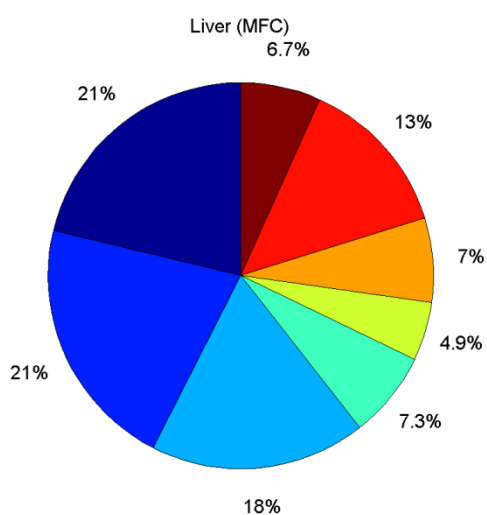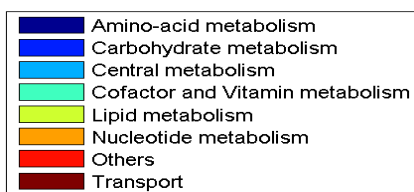

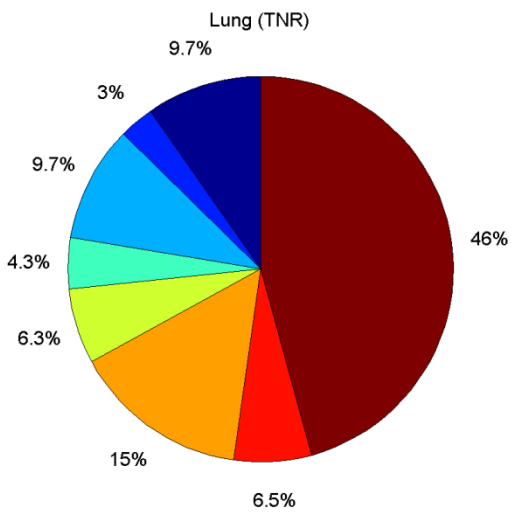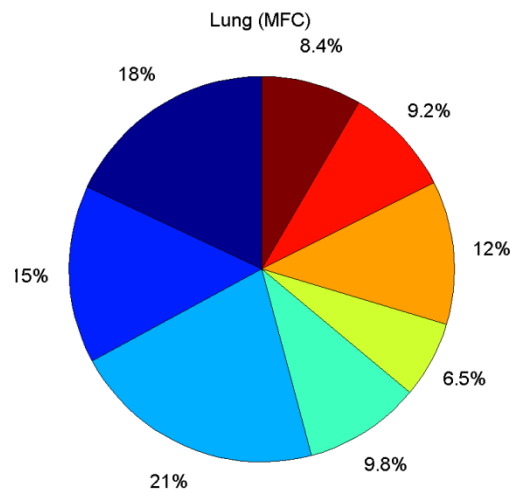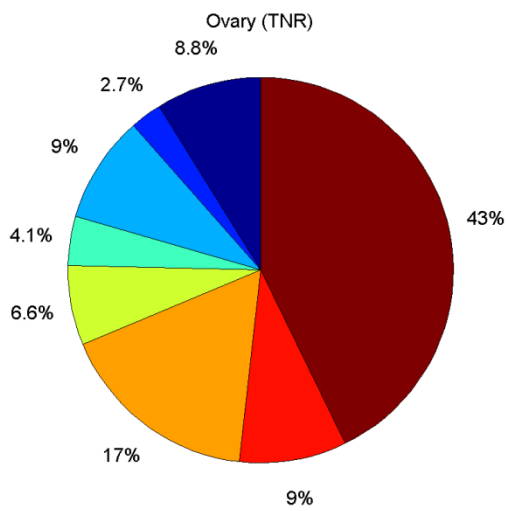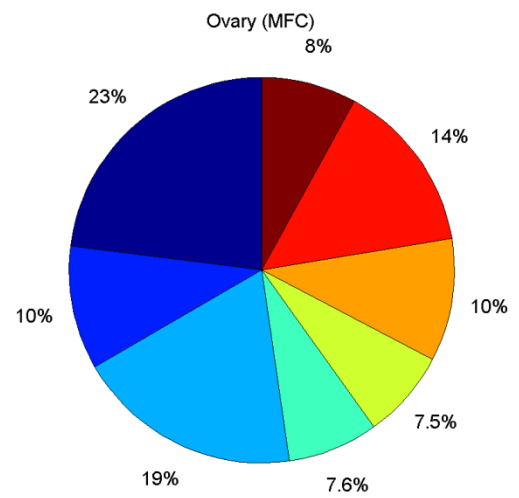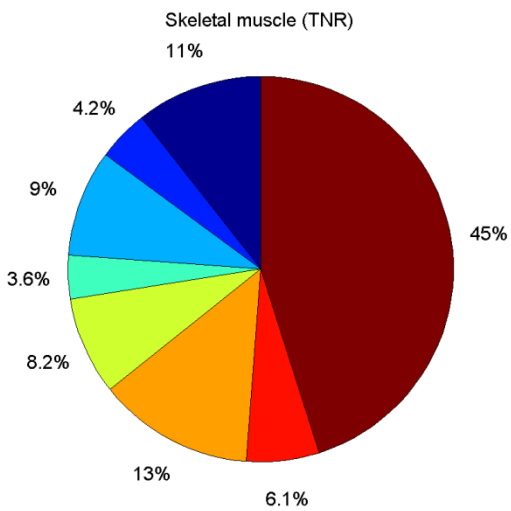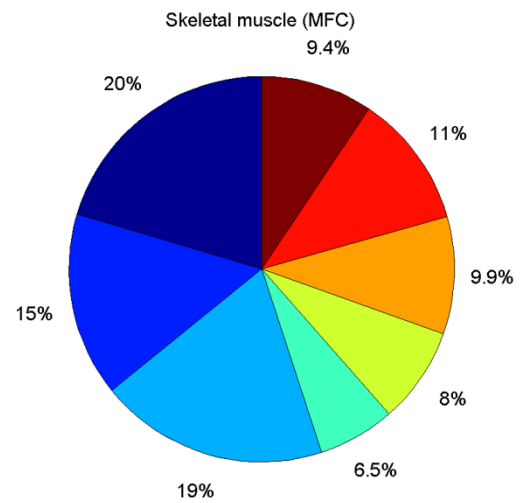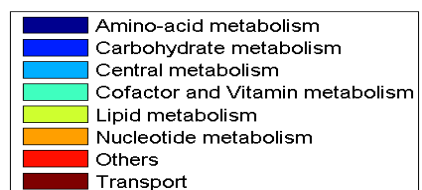

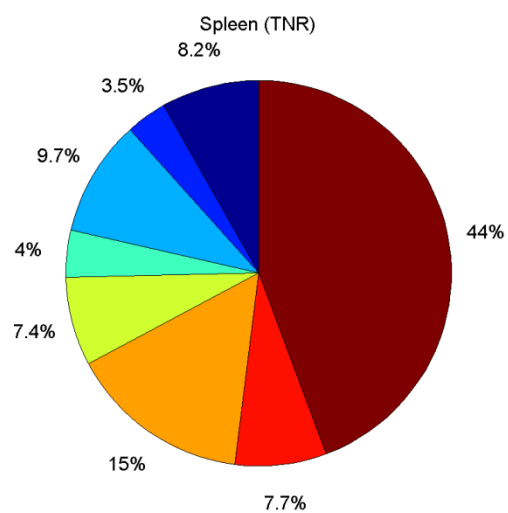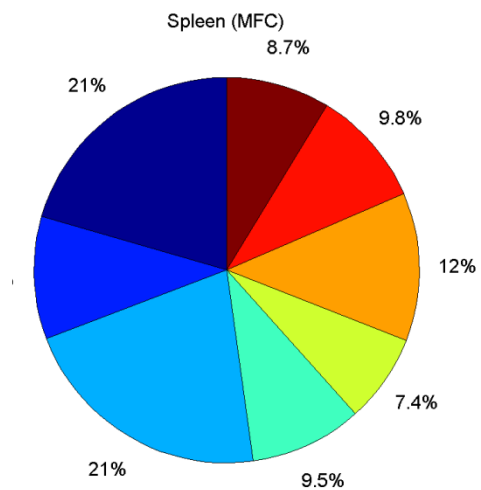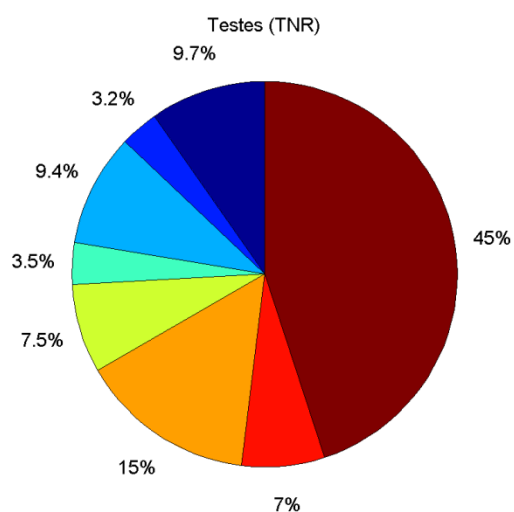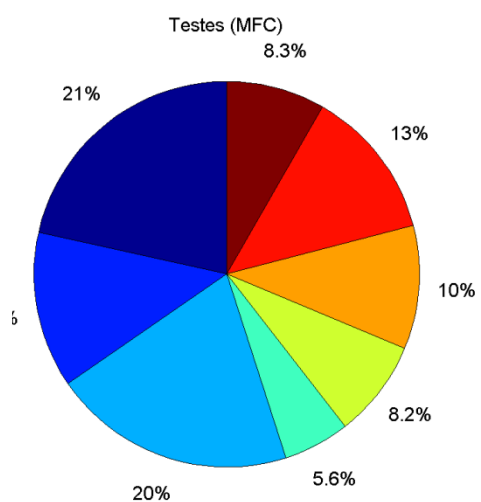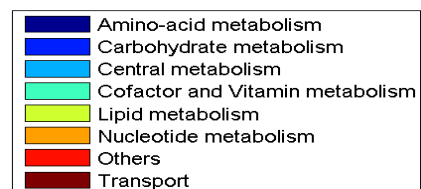

Supplement: S1 Fig — The importance of each metabolic category in each contexts is quantified using the total number of reactions (TNR) belonging to it and their mean flux capacity (MFC). (PDF) [file pone.0131875.s001.pdf]

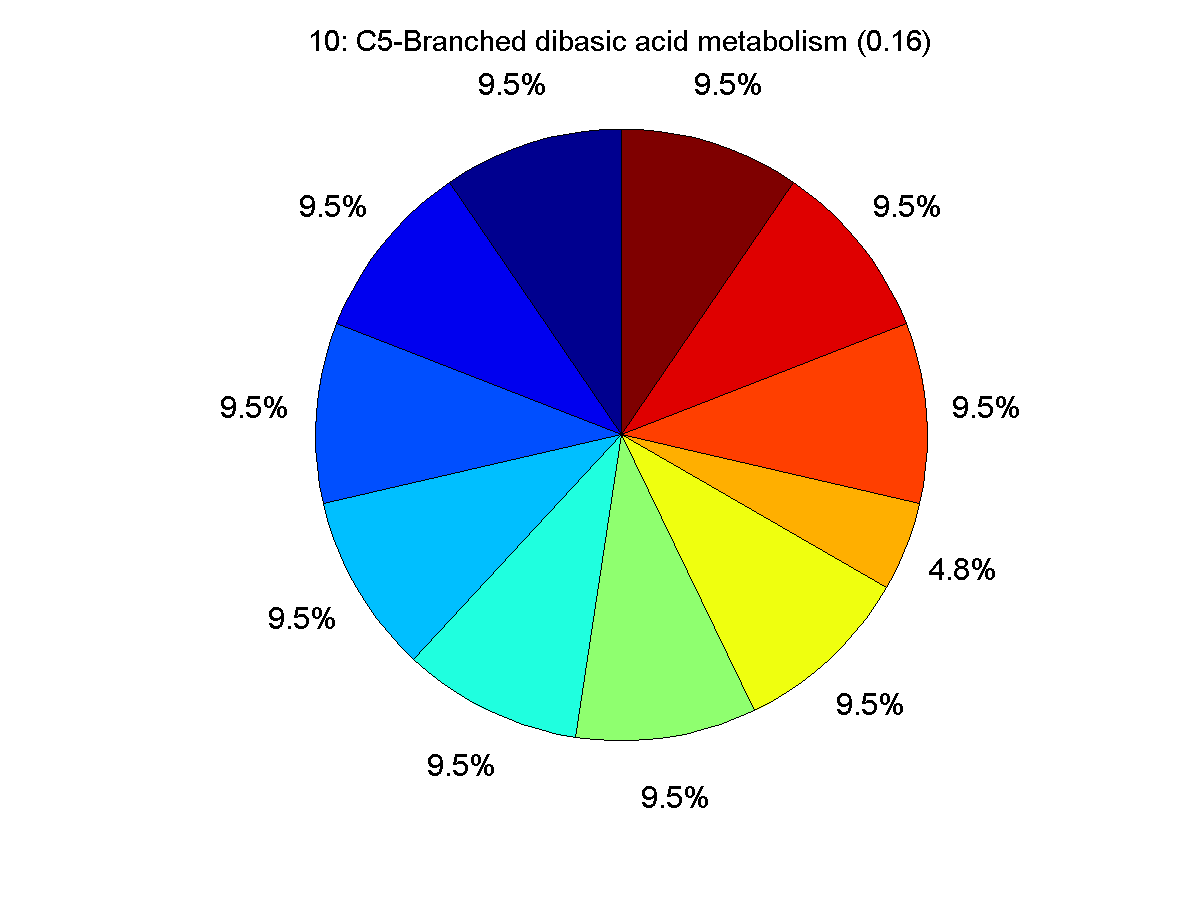

Supplement: S2 File — (ZIP) [file pone.0131875.s003.zip › MFC PieCharts/RegrEx1MFC/10C5-Brancheddibasicacidmetabolism.tif]

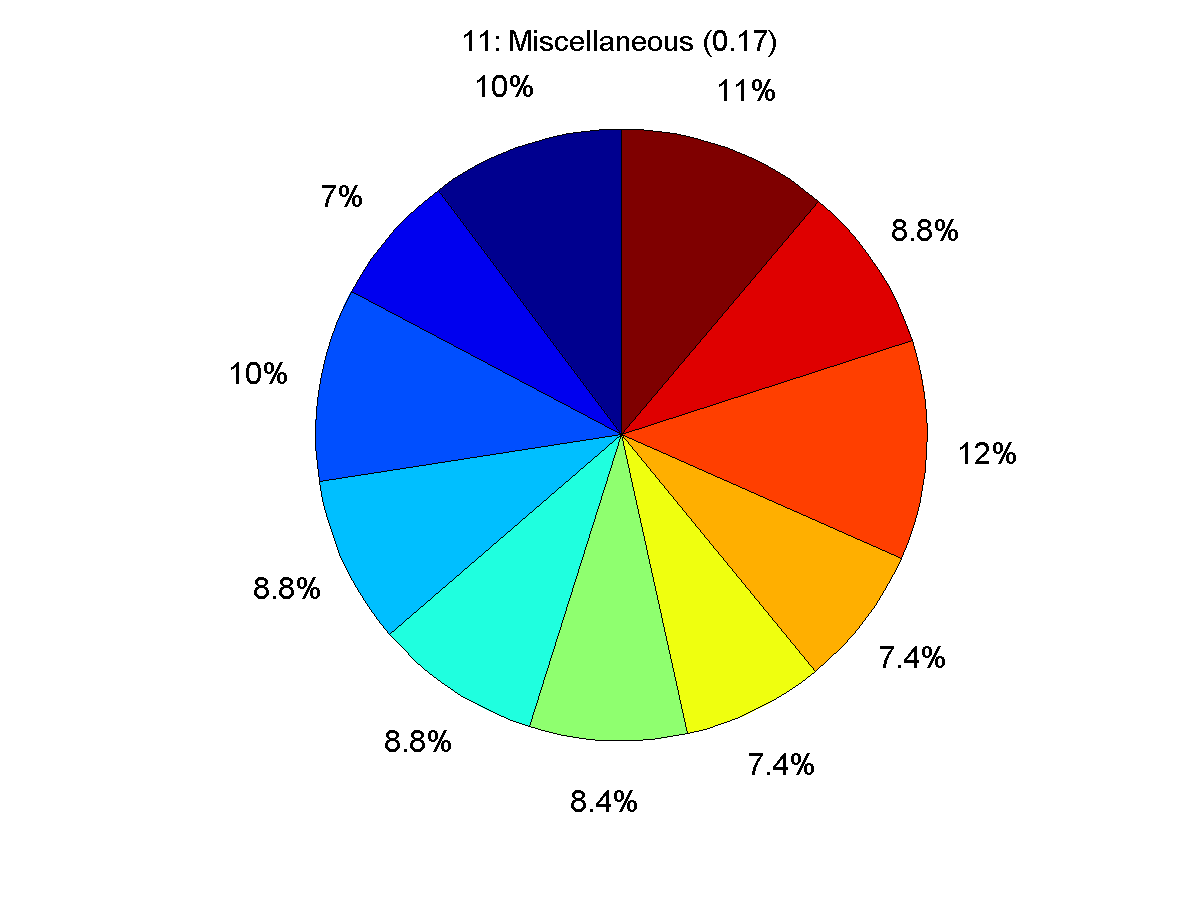

Supplement: S2 File — (ZIP) [file pone.0131875.s003.zip › MFC PieCharts/RegrEx1MFC/11Miscellaneous.tif]

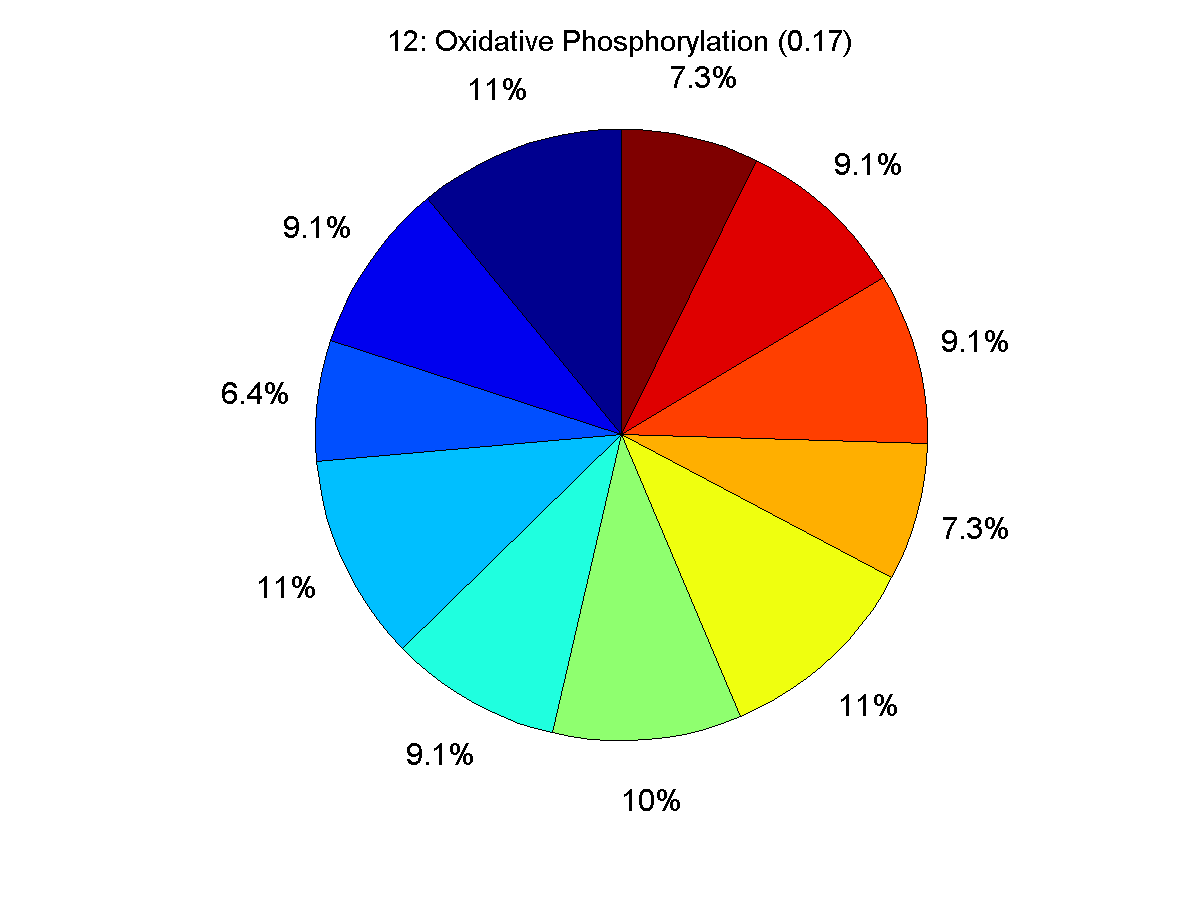

Supplement: S2 File — (ZIP) [file pone.0131875.s003.zip › MFC PieCharts/RegrEx1MFC/12OxidativePhosphorylation.tif]

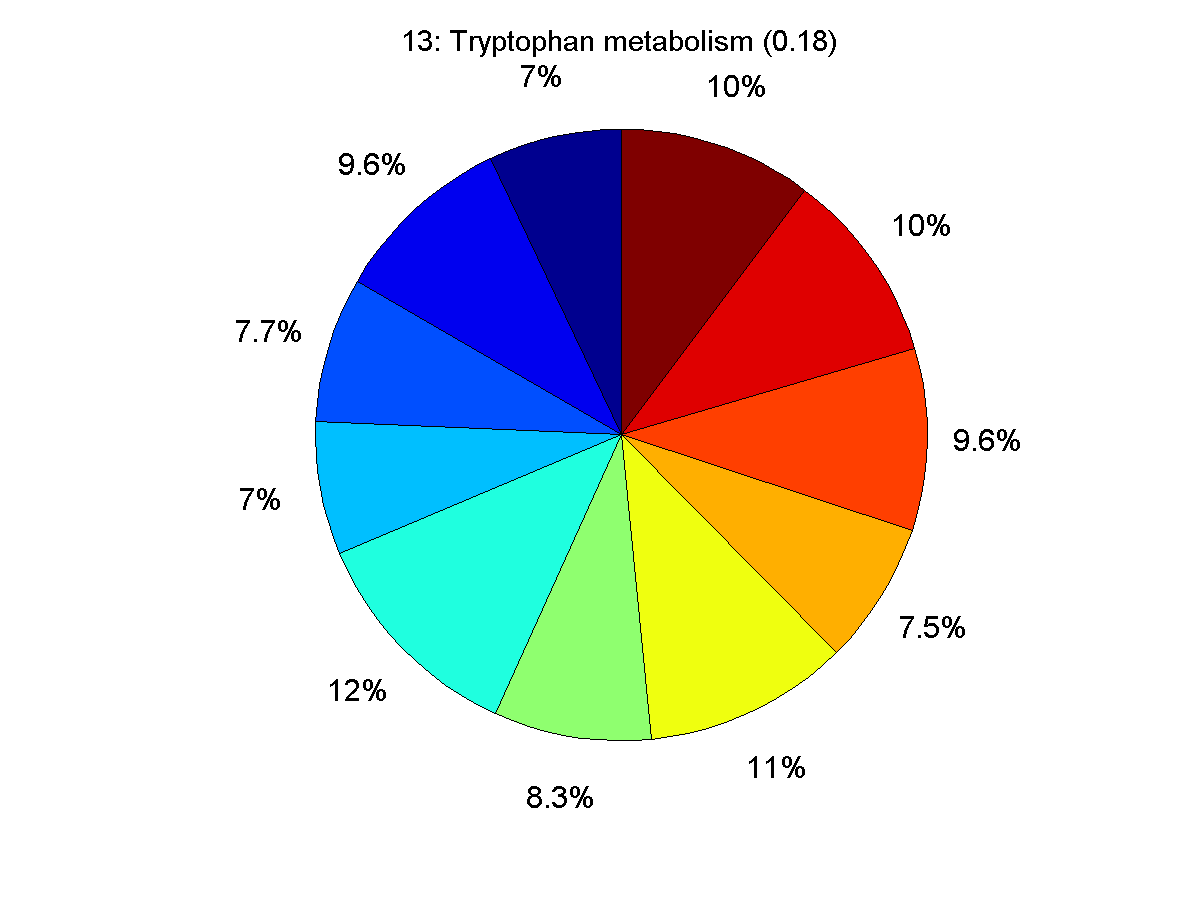

Supplement: S2 File — (ZIP) [file pone.0131875.s003.zip › MFC PieCharts/RegrEx1MFC/13Tryptophanmetabolism.tif]

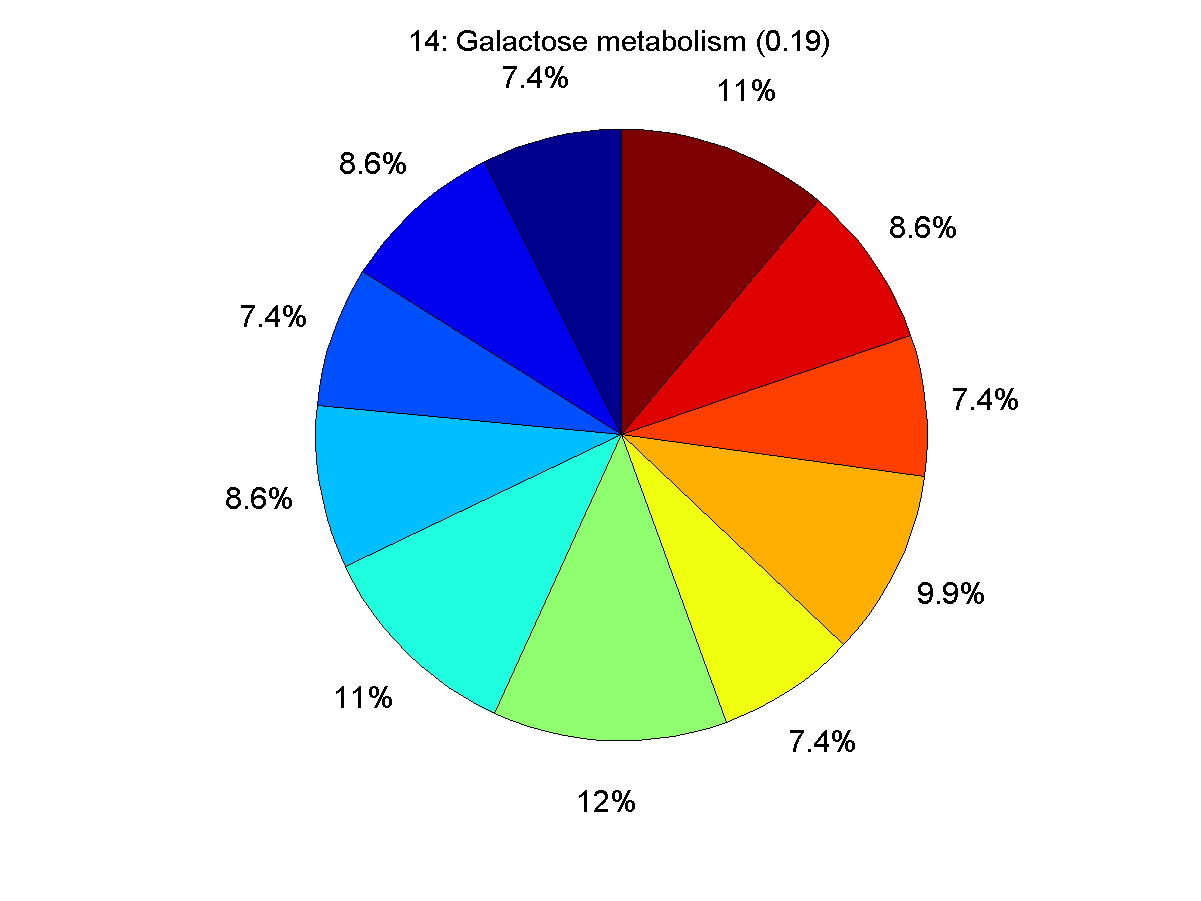

Supplement: S2 File — (ZIP) [file pone.0131875.s003.zip › MFC PieCharts/RegrEx1MFC/14Galactosemetabolism.tif]

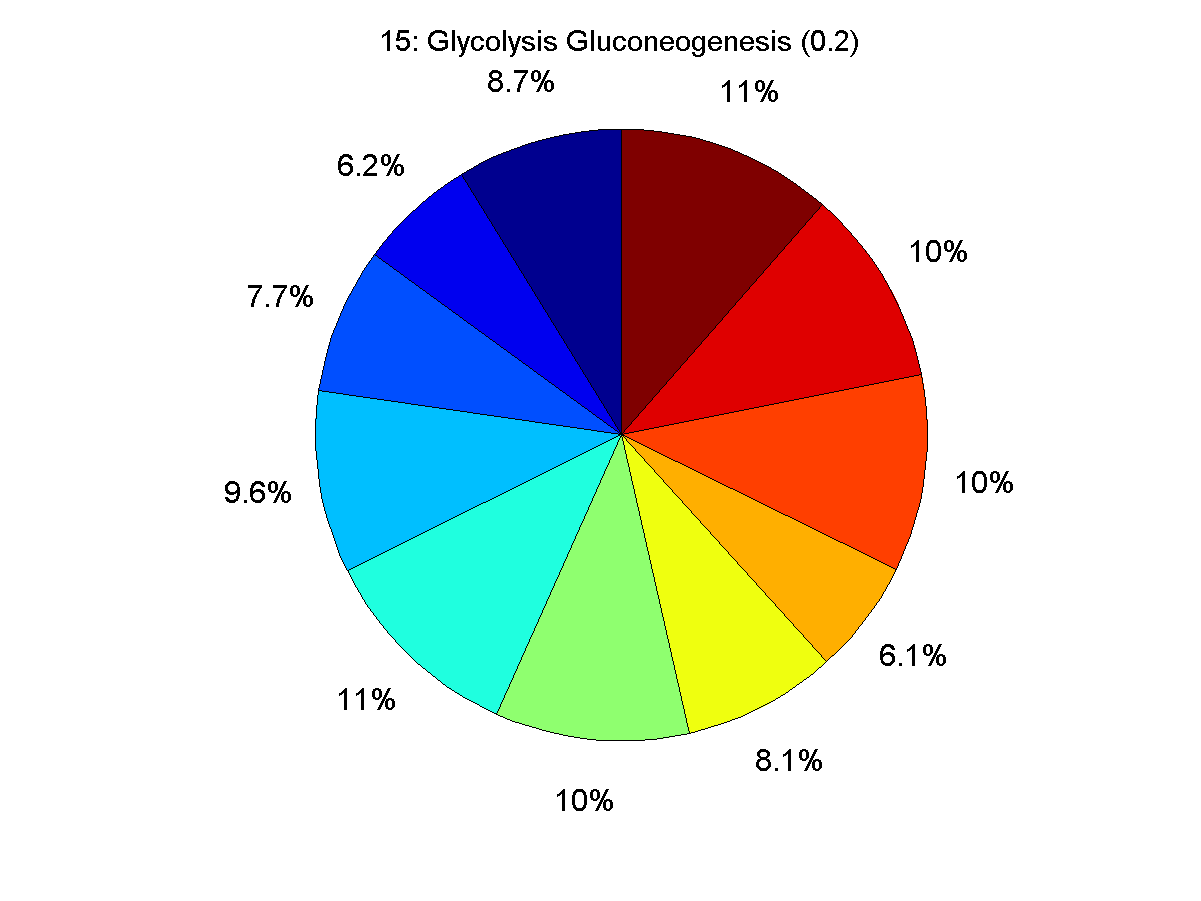

Supplement: S2 File — (ZIP) [file pone.0131875.s003.zip › MFC PieCharts/RegrEx1MFC/15GlycolysisGluconeogenesis.tif]

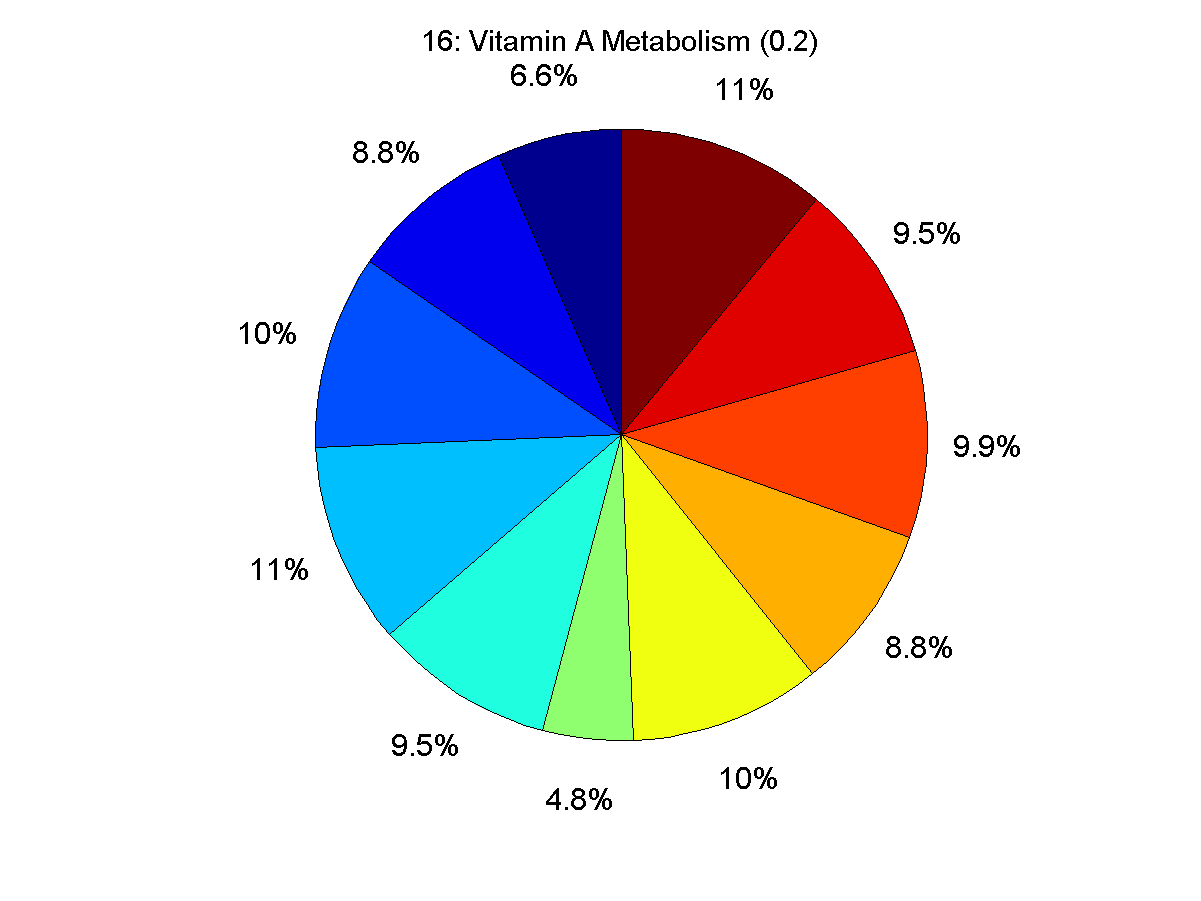

Supplement: S2 File — (ZIP) [file pone.0131875.s003.zip › MFC PieCharts/RegrEx1MFC/16VitaminAMetabolism.tif]

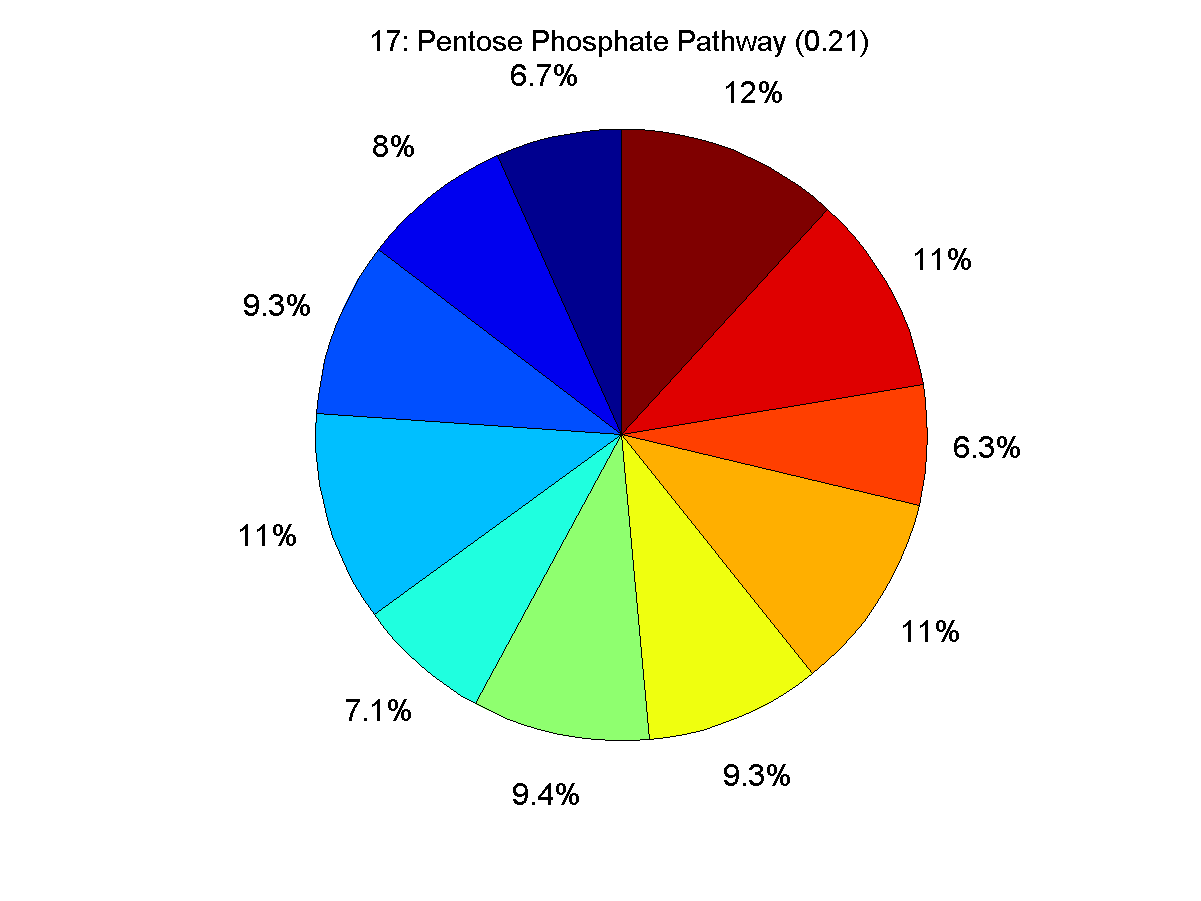

Supplement: S2 File — (ZIP) [file pone.0131875.s003.zip › MFC PieCharts/RegrEx1MFC/17PentosePhosphatePathway.tif]

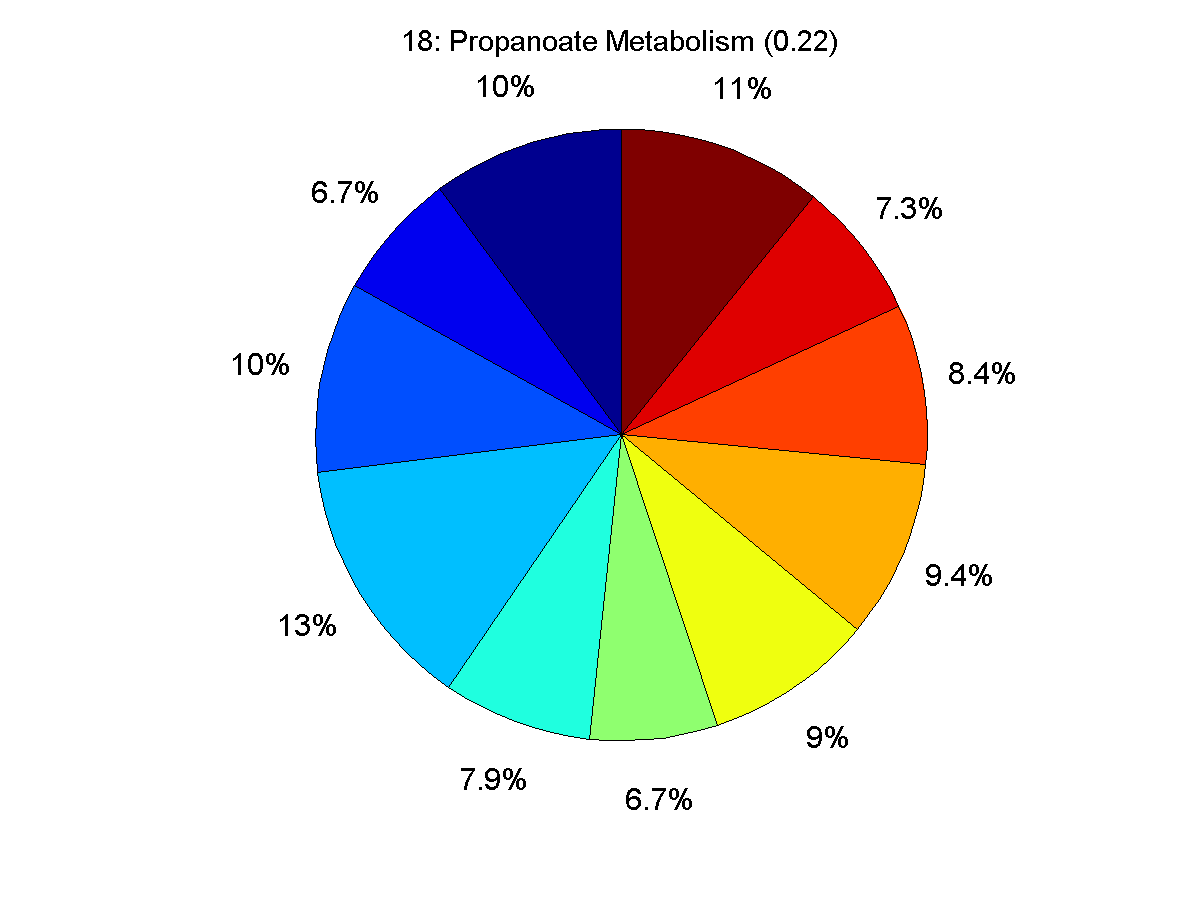

Supplement: S2 File — (ZIP) [file pone.0131875.s003.zip › MFC PieCharts/RegrEx1MFC/18PropanoateMetabolism.tif]

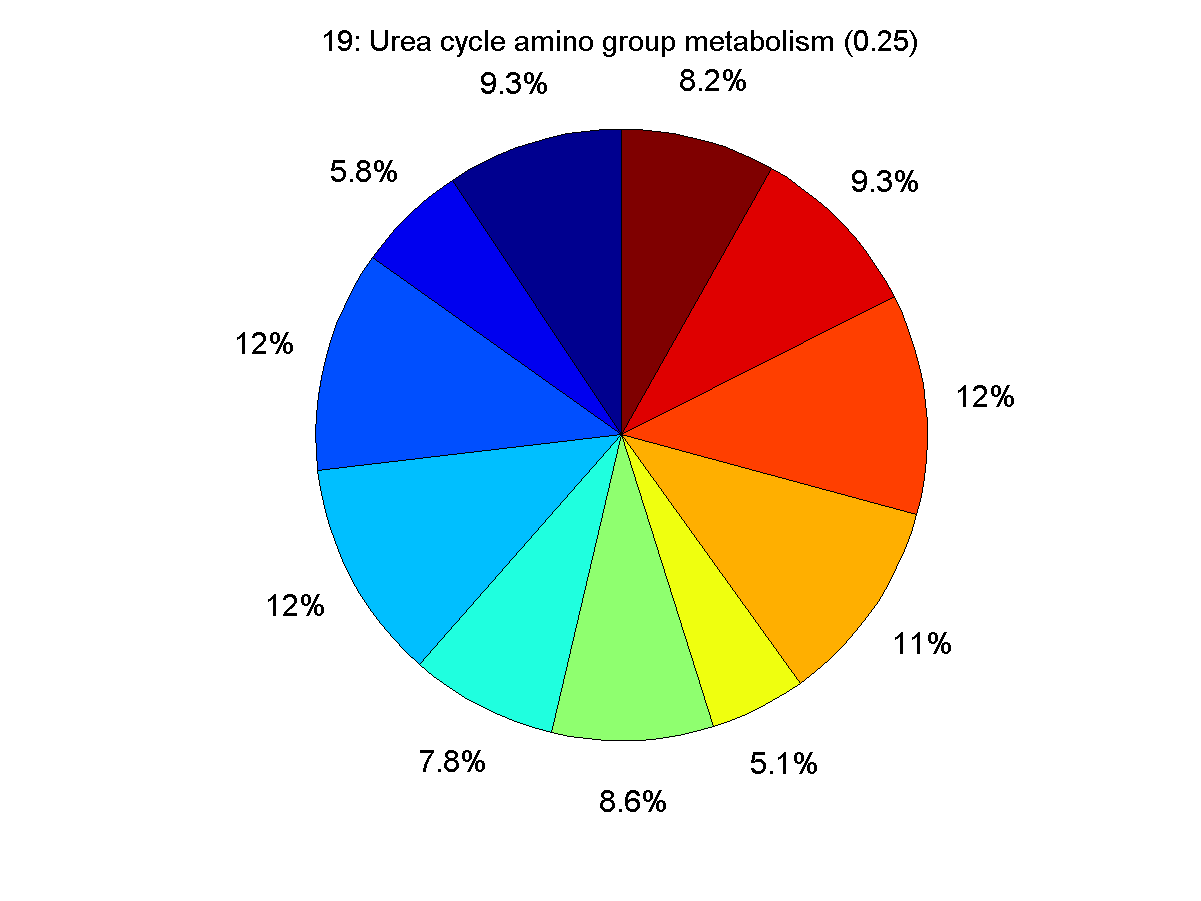

Supplement: S2 File — (ZIP) [file pone.0131875.s003.zip › MFC PieCharts/RegrEx1MFC/19Ureacycleaminogroupmetabolism.tif]

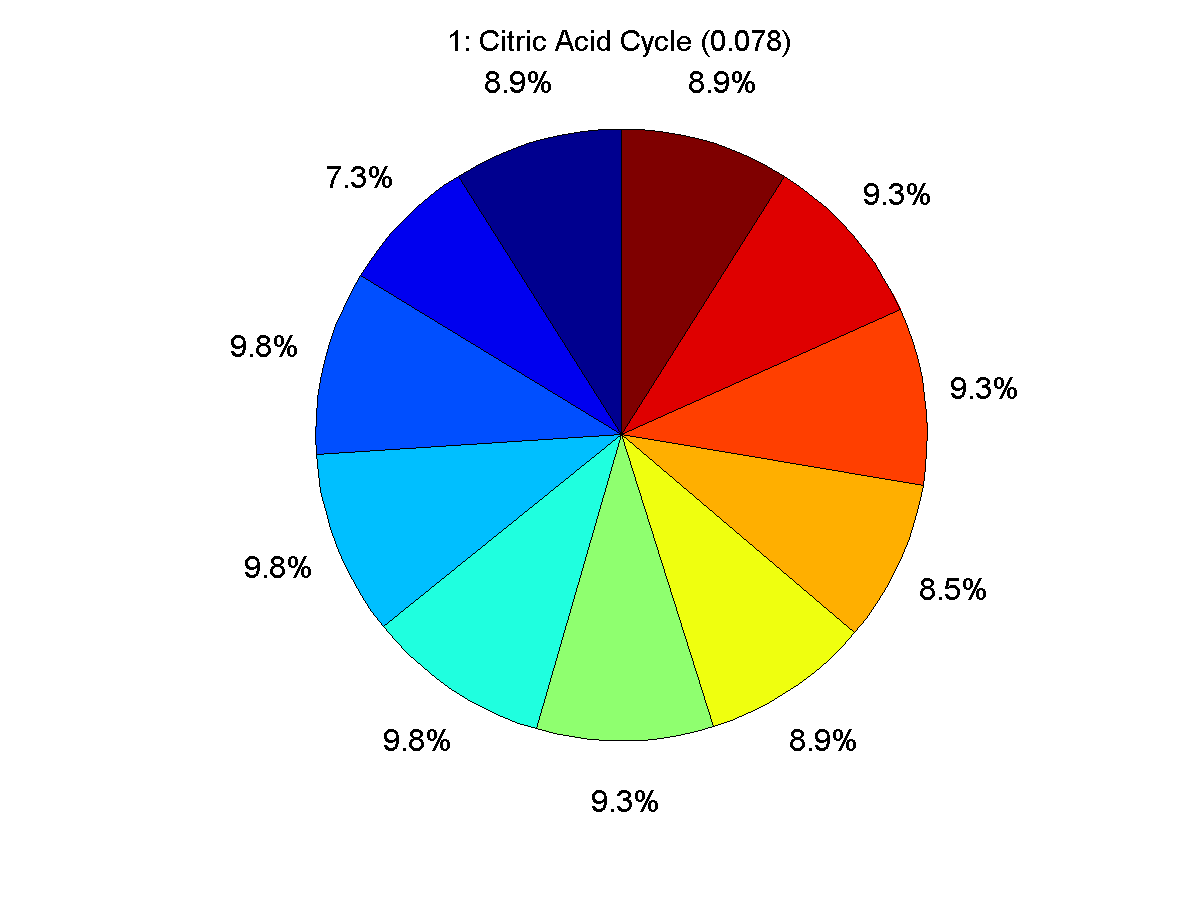

Supplement: S2 File — (ZIP) [file pone.0131875.s003.zip › MFC PieCharts/RegrEx1MFC/1CitricAcidCycle.tif]

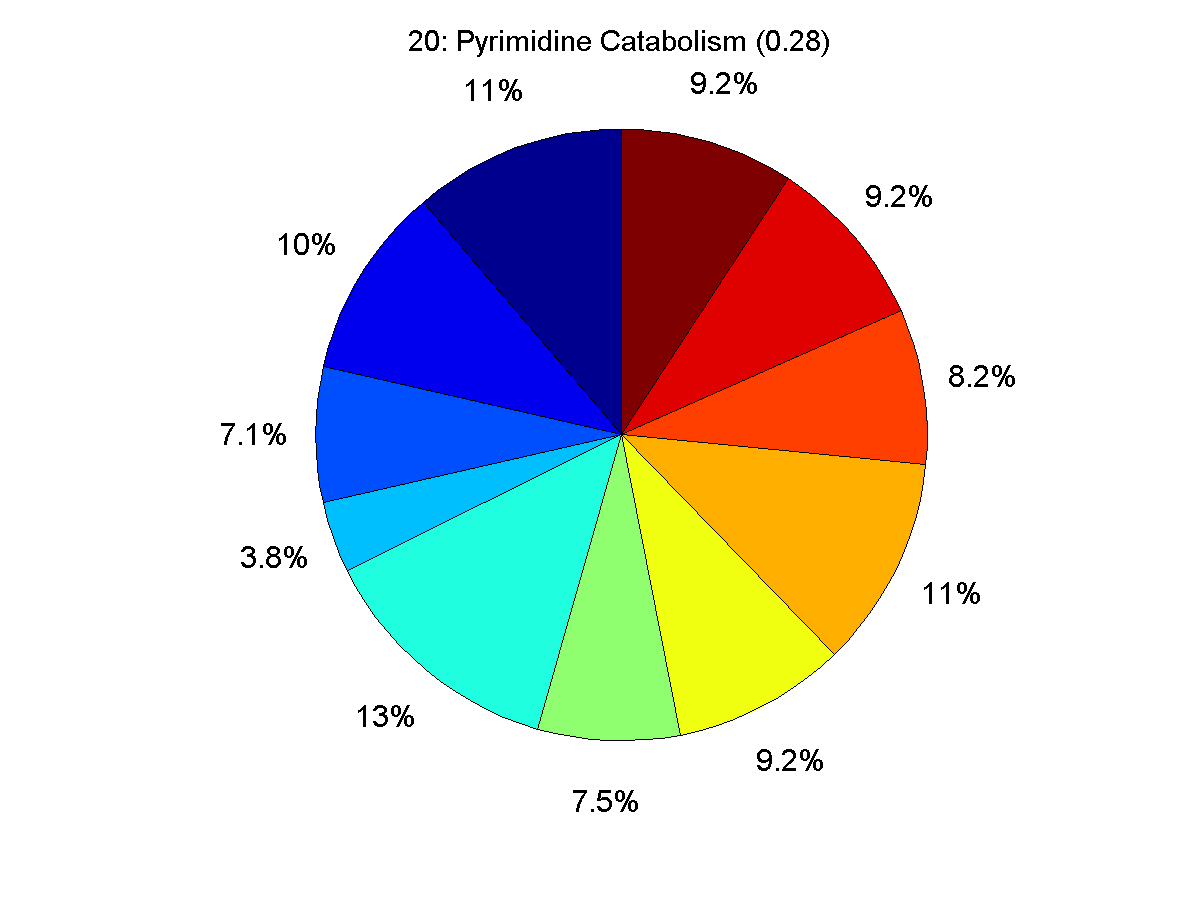

Supplement: S2 File — (ZIP) [file pone.0131875.s003.zip › MFC PieCharts/RegrEx1MFC/20PyrimidineCatabolism.tif]

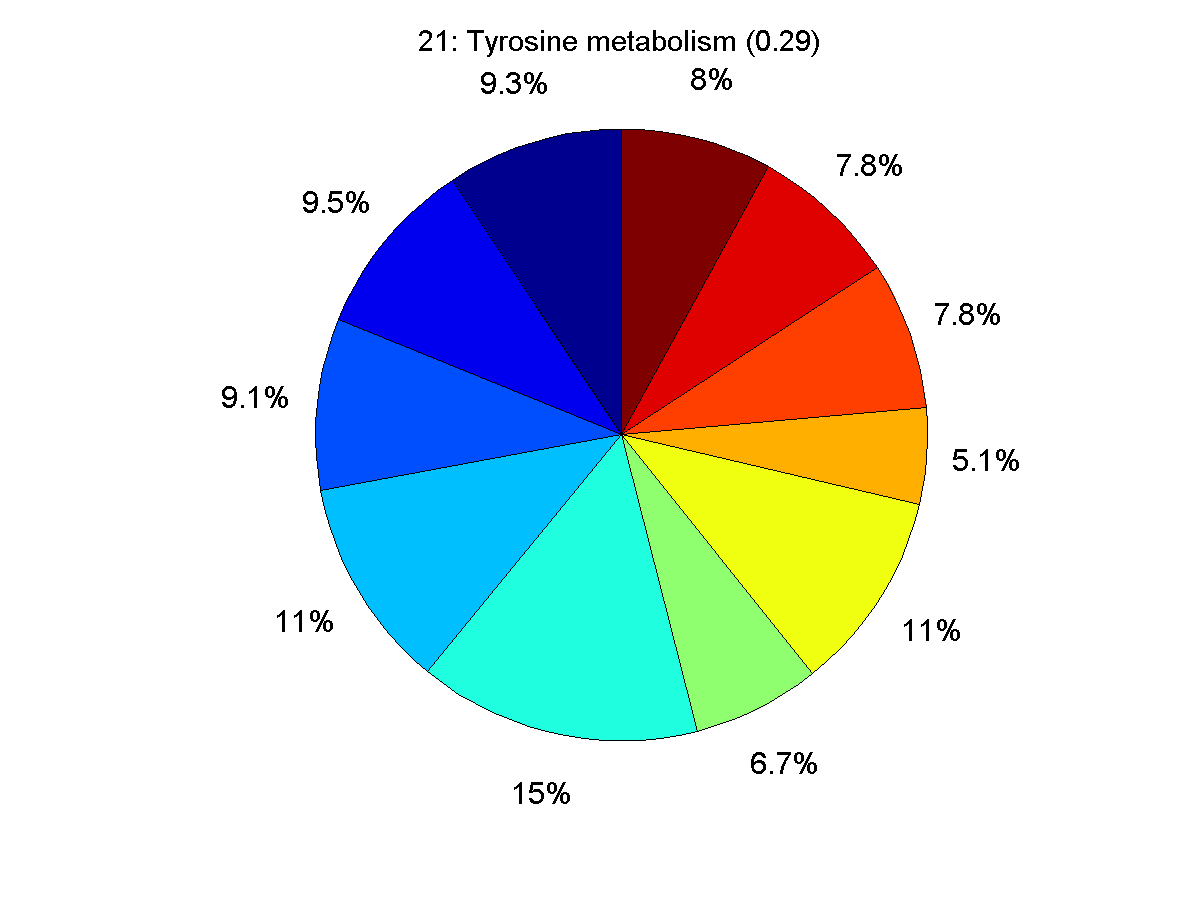

Supplement: S2 File — (ZIP) [file pone.0131875.s003.zip › MFC PieCharts/RegrEx1MFC/21Tyrosinemetabolism.tif]

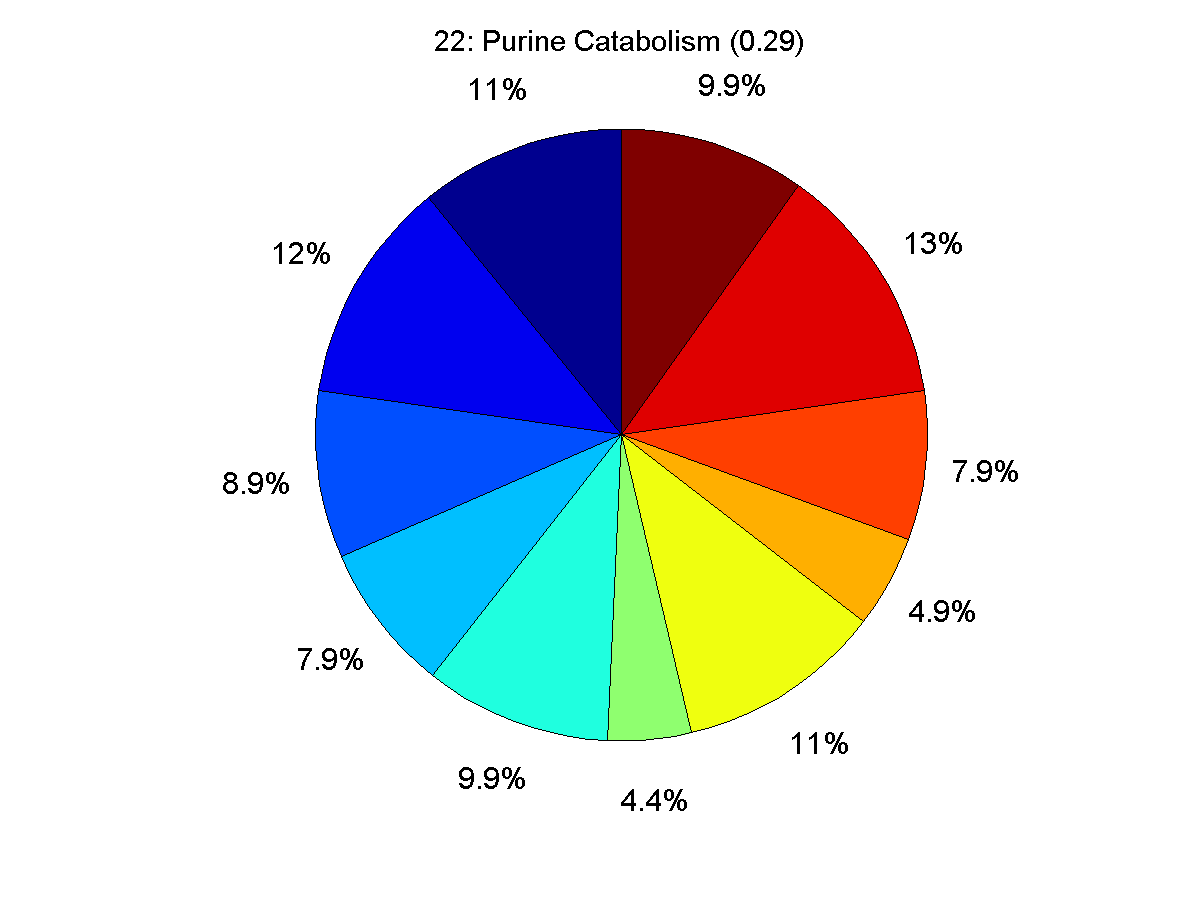

Supplement: S2 File — (ZIP) [file pone.0131875.s003.zip › MFC PieCharts/RegrEx1MFC/22PurineCatabolism.tif]

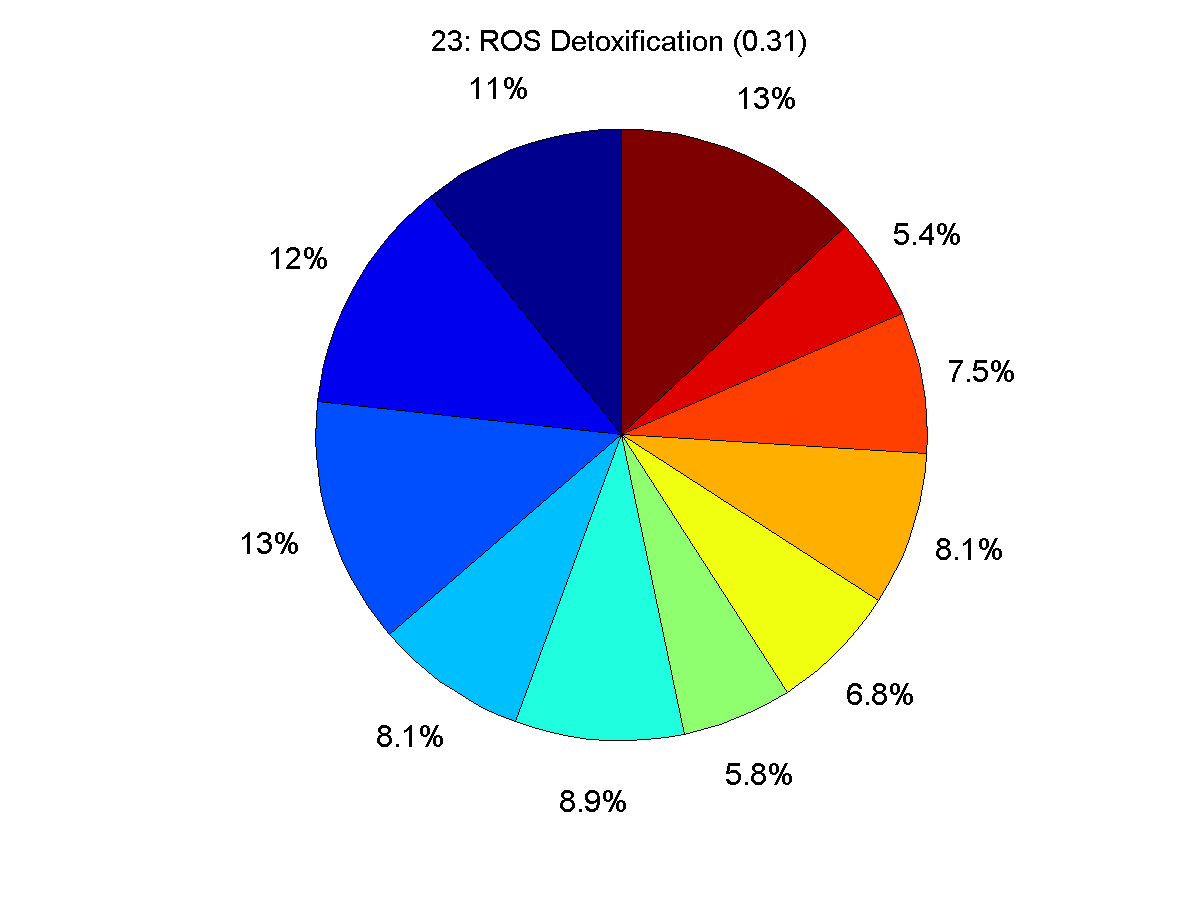

Supplement: S2 File — (ZIP) [file pone.0131875.s003.zip › MFC PieCharts/RegrEx1MFC/23ROSDetoxification.tif]

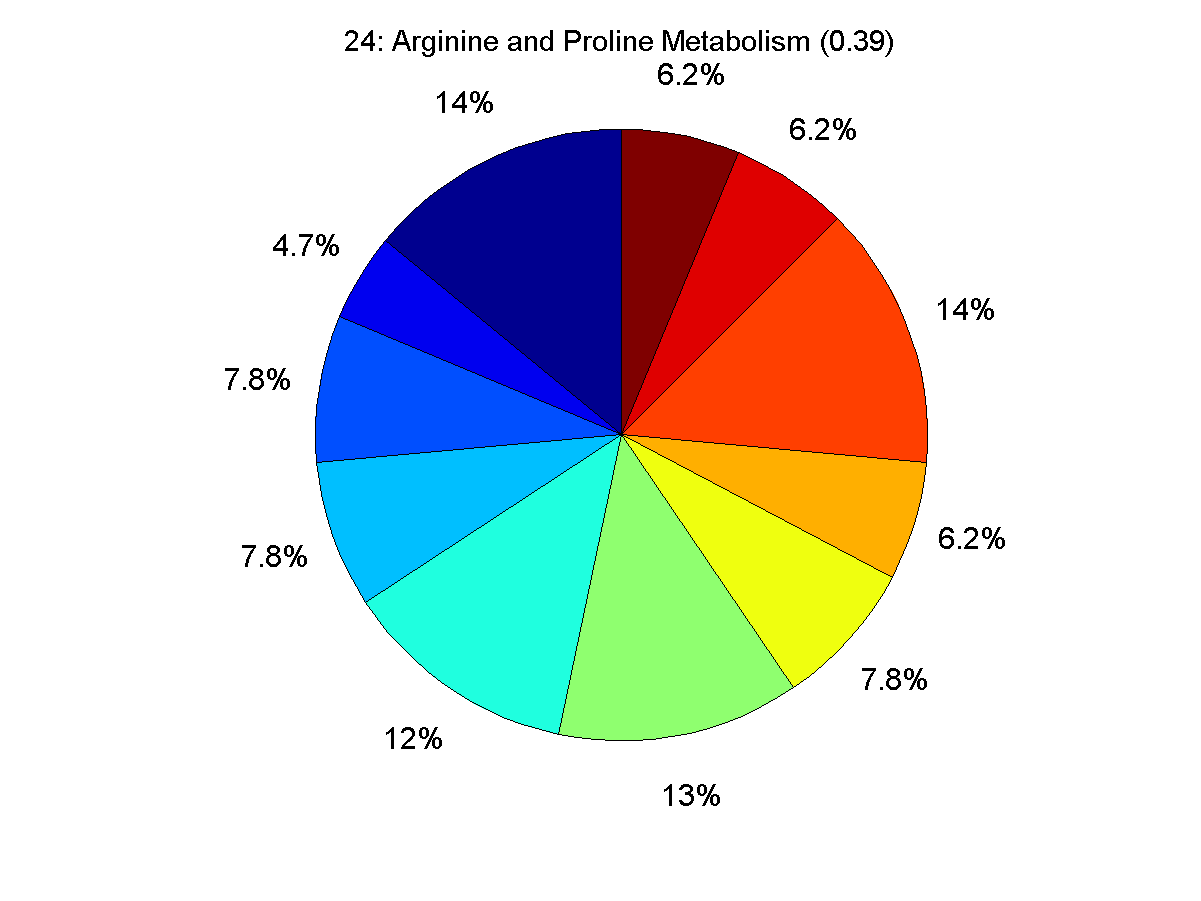

Supplement: S2 File — (ZIP) [file pone.0131875.s003.zip › MFC PieCharts/RegrEx1MFC/24ArginineandProlineMetabolism.tif]

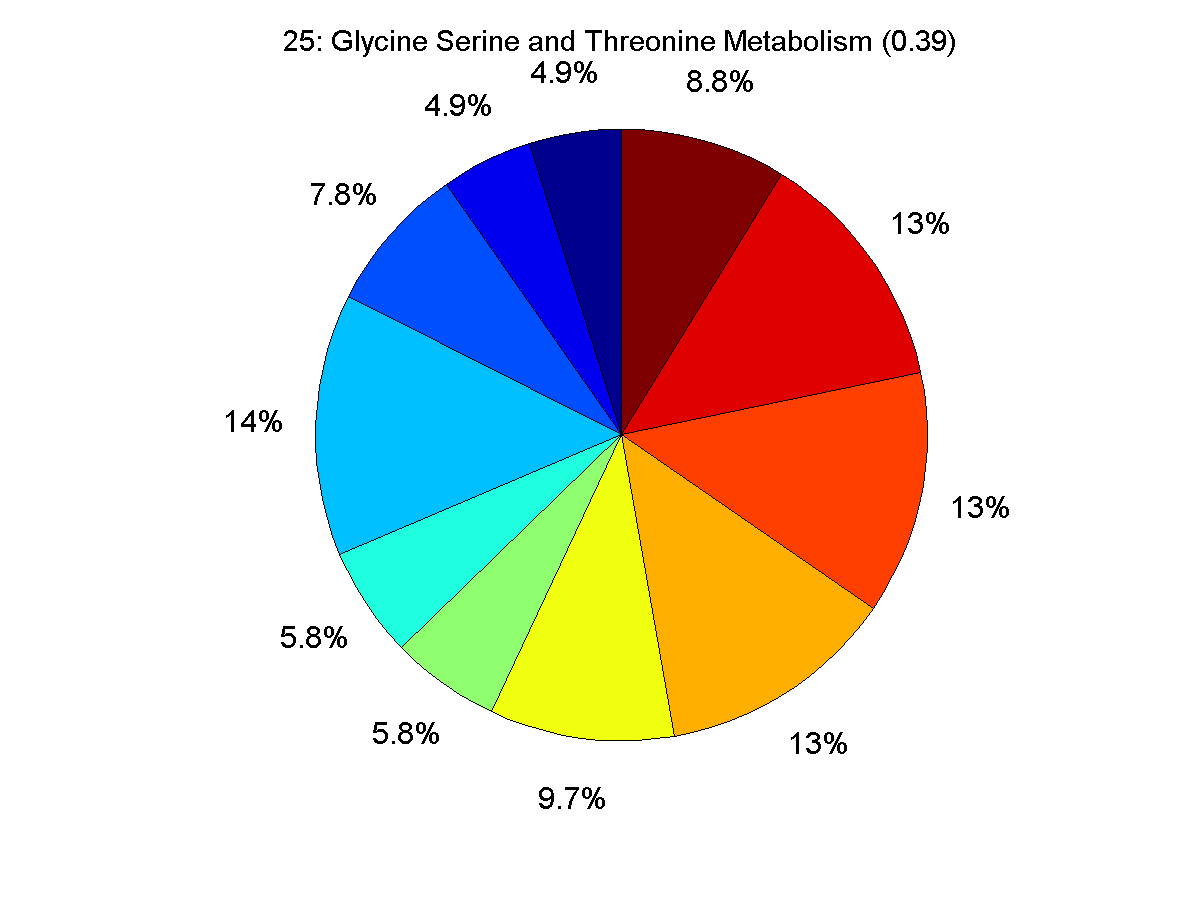

Supplement: S2 File — (ZIP) [file pone.0131875.s003.zip › MFC PieCharts/RegrEx1MFC/25GlycineSerineandThreonineMetabolism.tif]

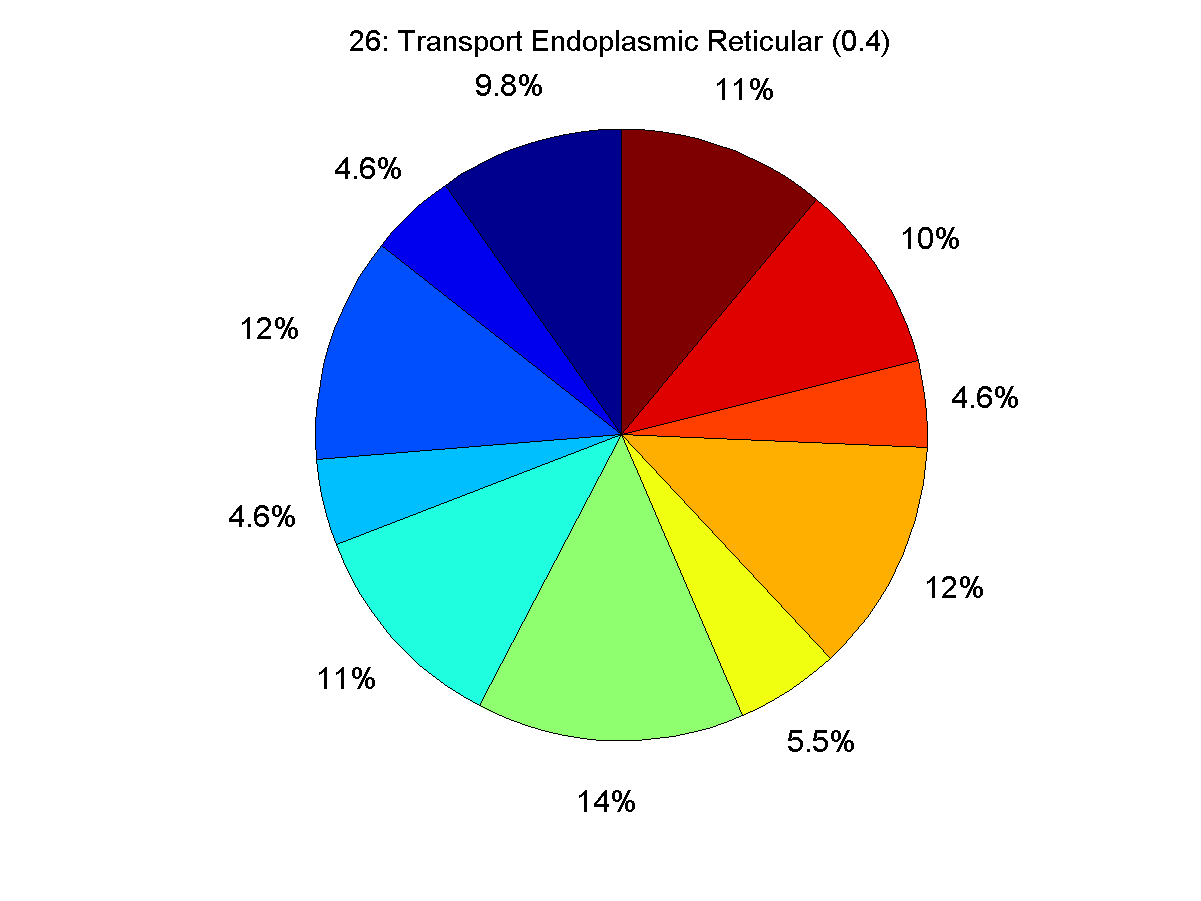

Supplement: S2 File — (ZIP) [file pone.0131875.s003.zip › MFC PieCharts/RegrEx1MFC/26TransportEndoplasmicReticular.tif]

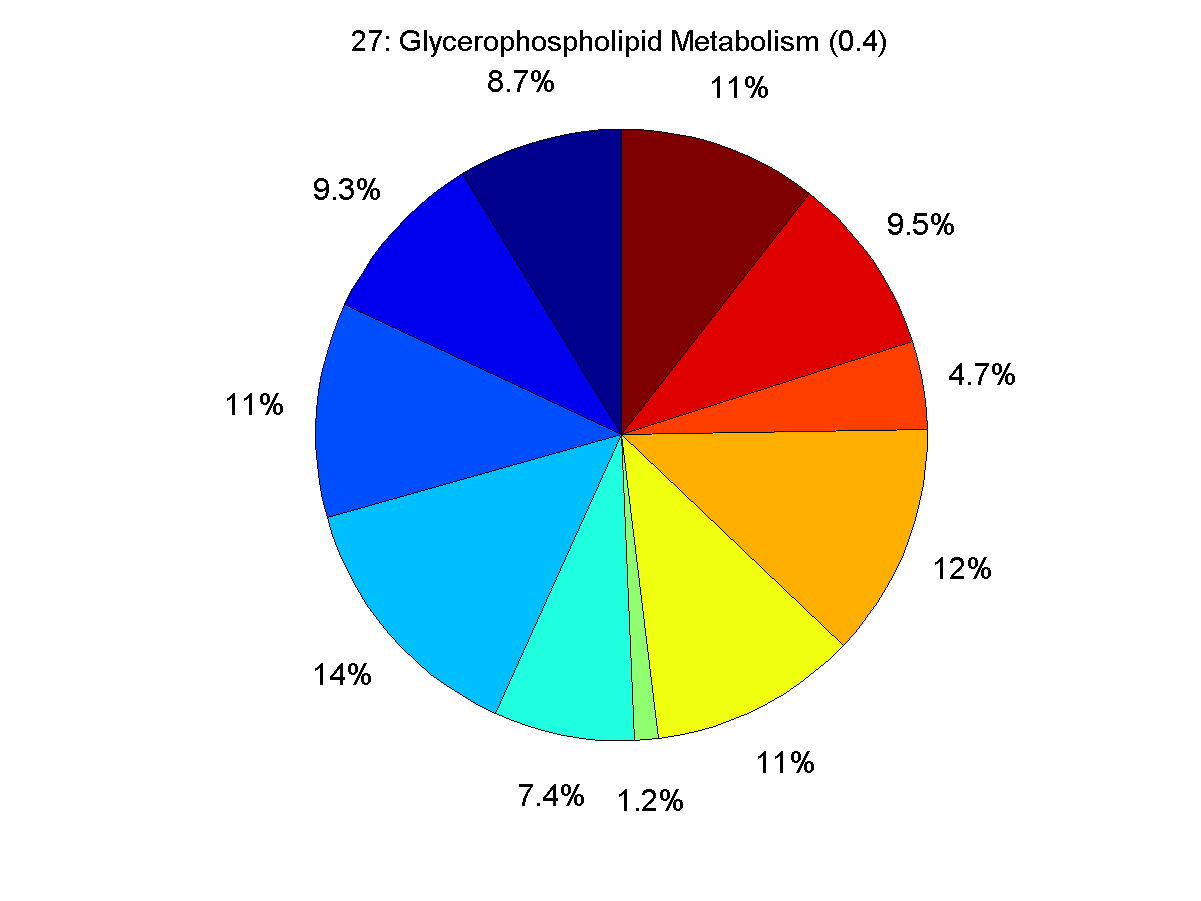

Supplement: S2 File — (ZIP) [file pone.0131875.s003.zip › MFC PieCharts/RegrEx1MFC/27GlycerophospholipidMetabolism.tif]

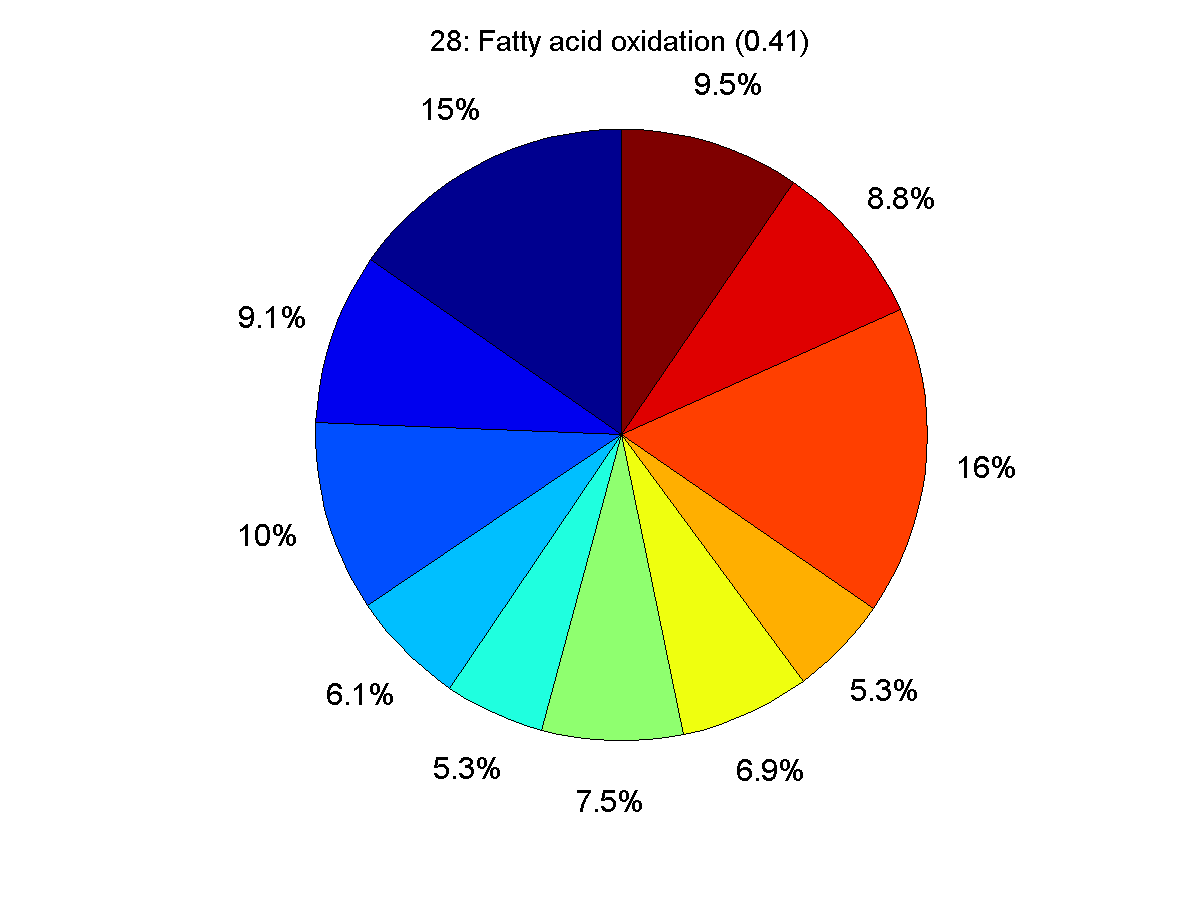

Supplement: S2 File — (ZIP) [file pone.0131875.s003.zip › MFC PieCharts/RegrEx1MFC/28Fattyacidoxidation.tif]

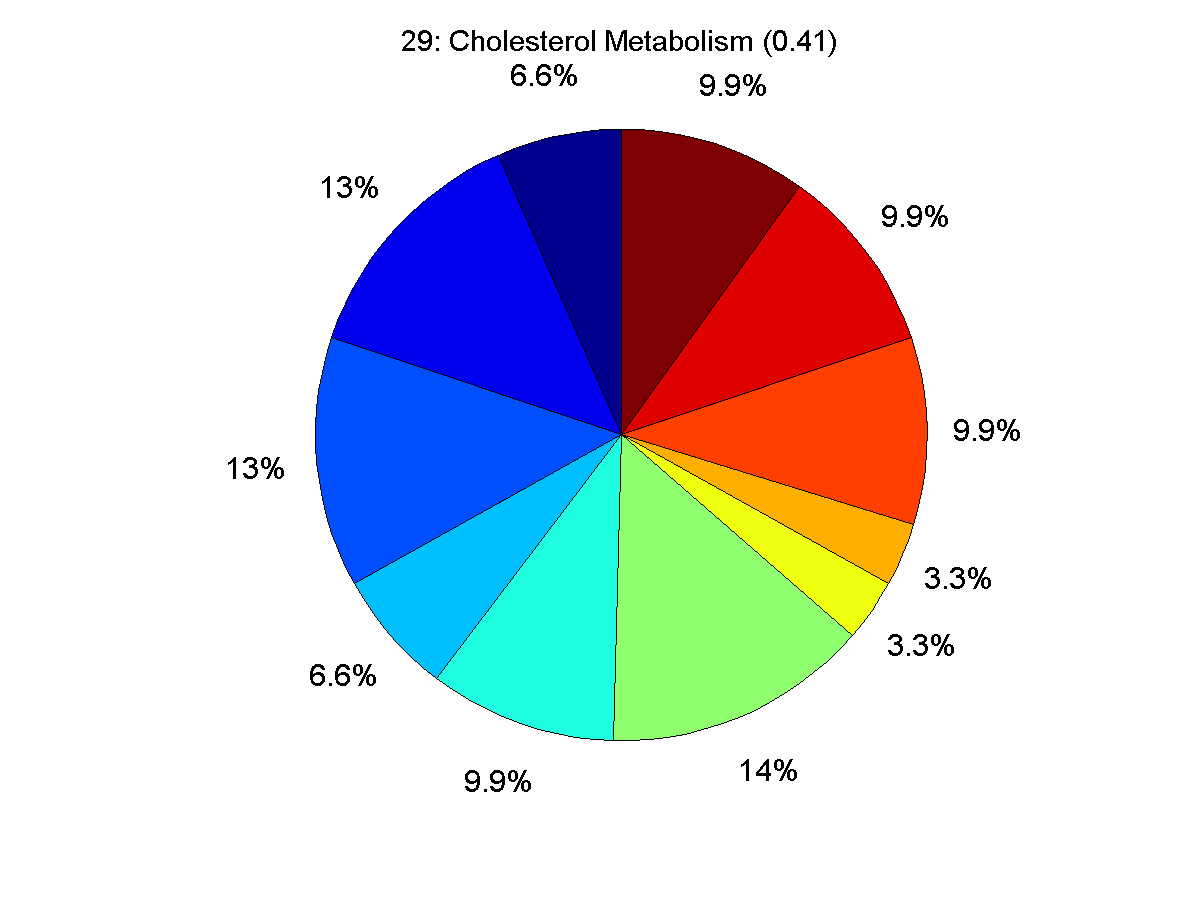

Supplement: S2 File — (ZIP) [file pone.0131875.s003.zip › MFC PieCharts/RegrEx1MFC/29CholesterolMetabolism.tif]

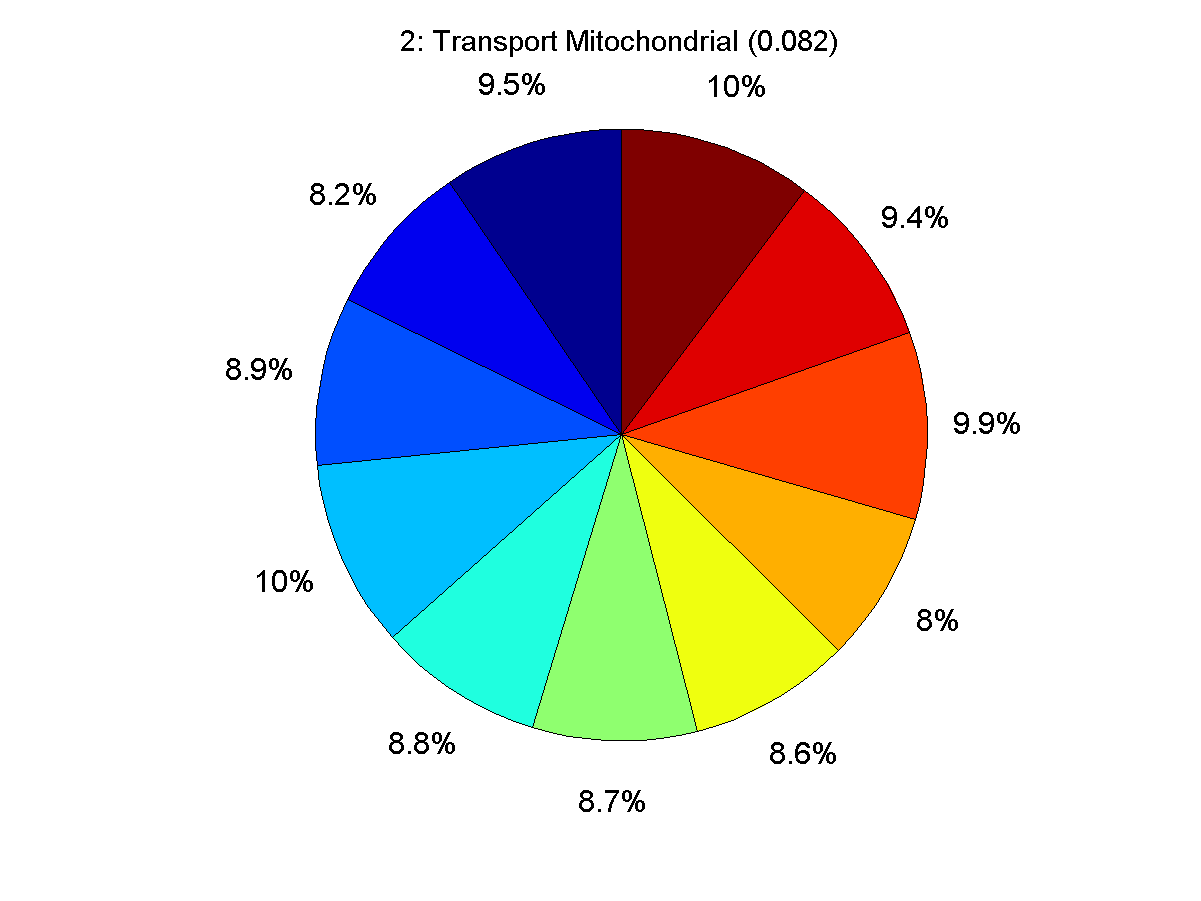

Supplement: S2 File — (ZIP) [file pone.0131875.s003.zip › MFC PieCharts/RegrEx1MFC/2TransportMitochondrial.tif]

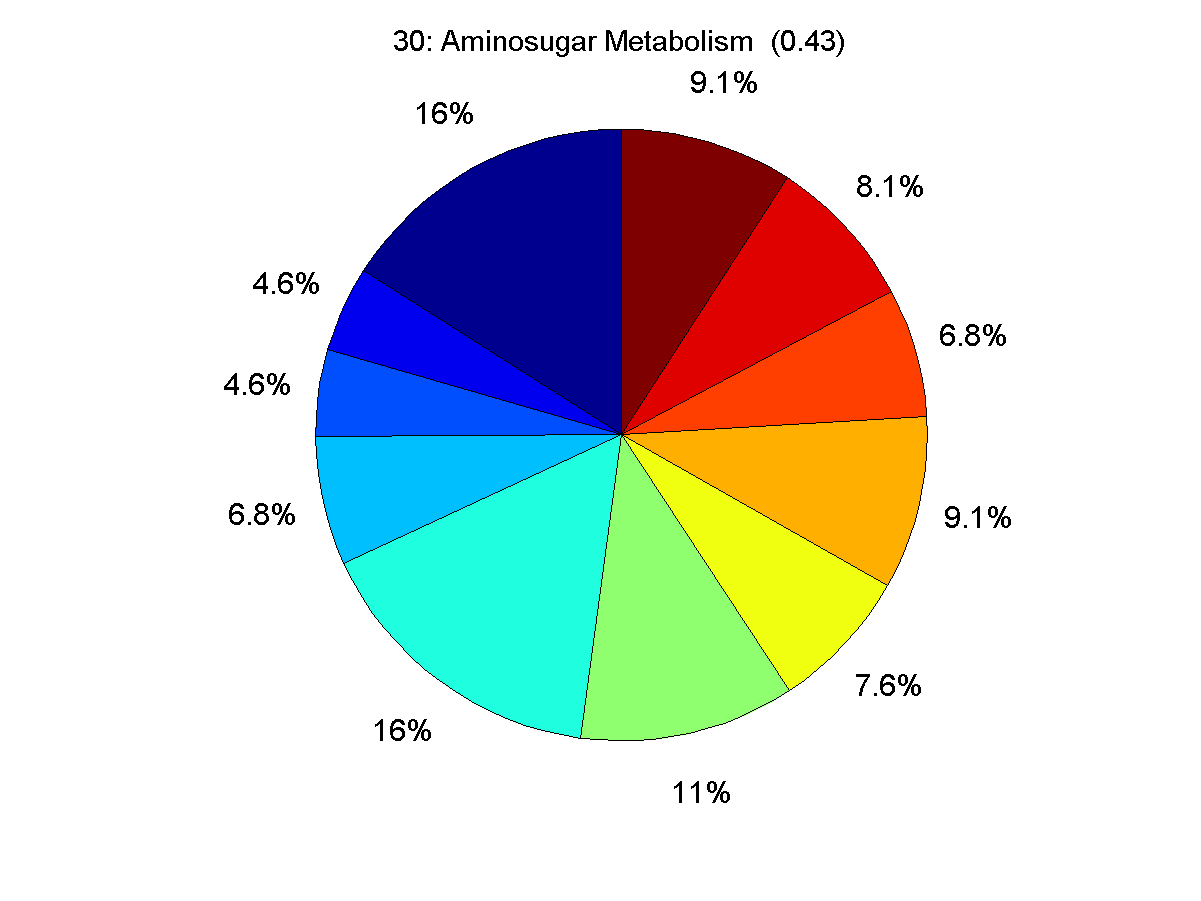

Supplement: S2 File — (ZIP) [file pone.0131875.s003.zip › MFC PieCharts/RegrEx1MFC/30AminosugarMetabolism.tif]

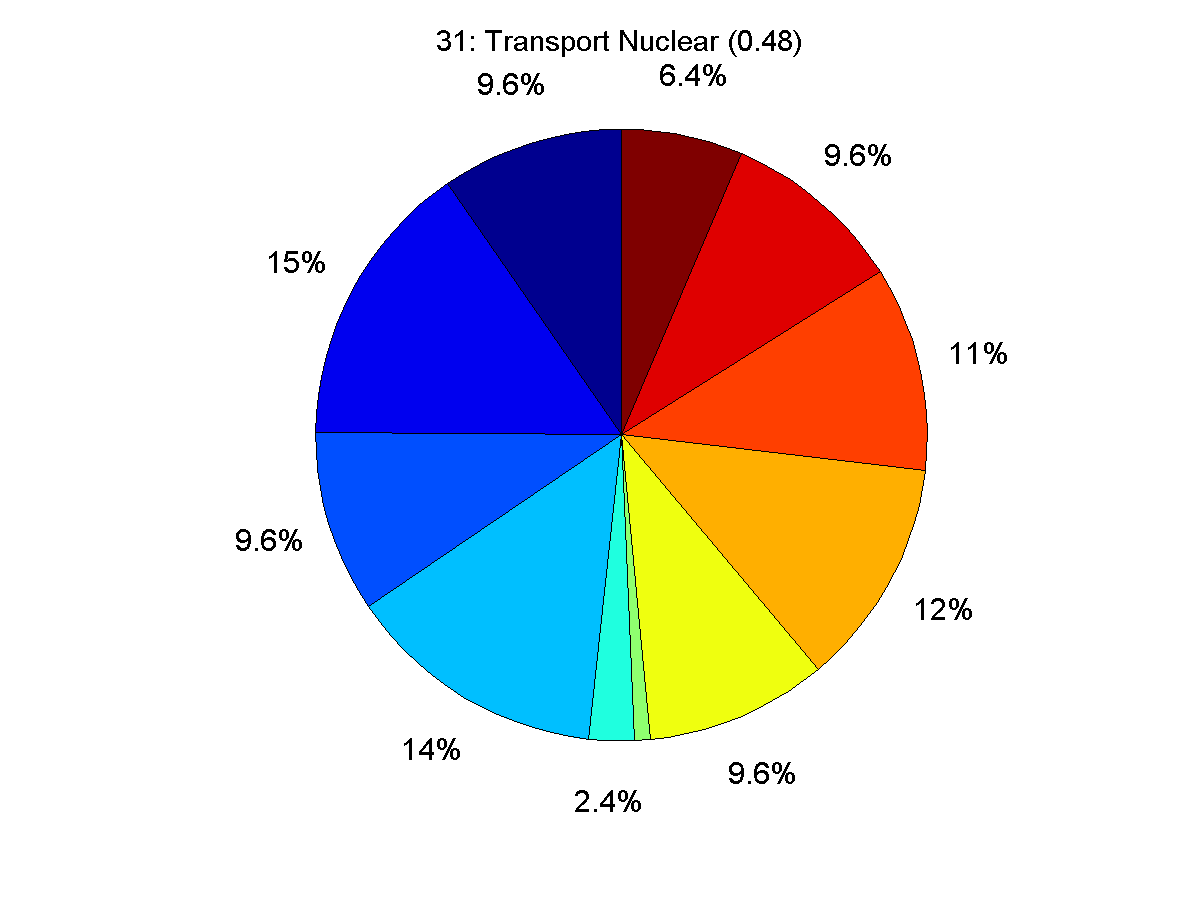

Supplement: S2 File — (ZIP) [file pone.0131875.s003.zip › MFC PieCharts/RegrEx1MFC/31TransportNuclear.tif]

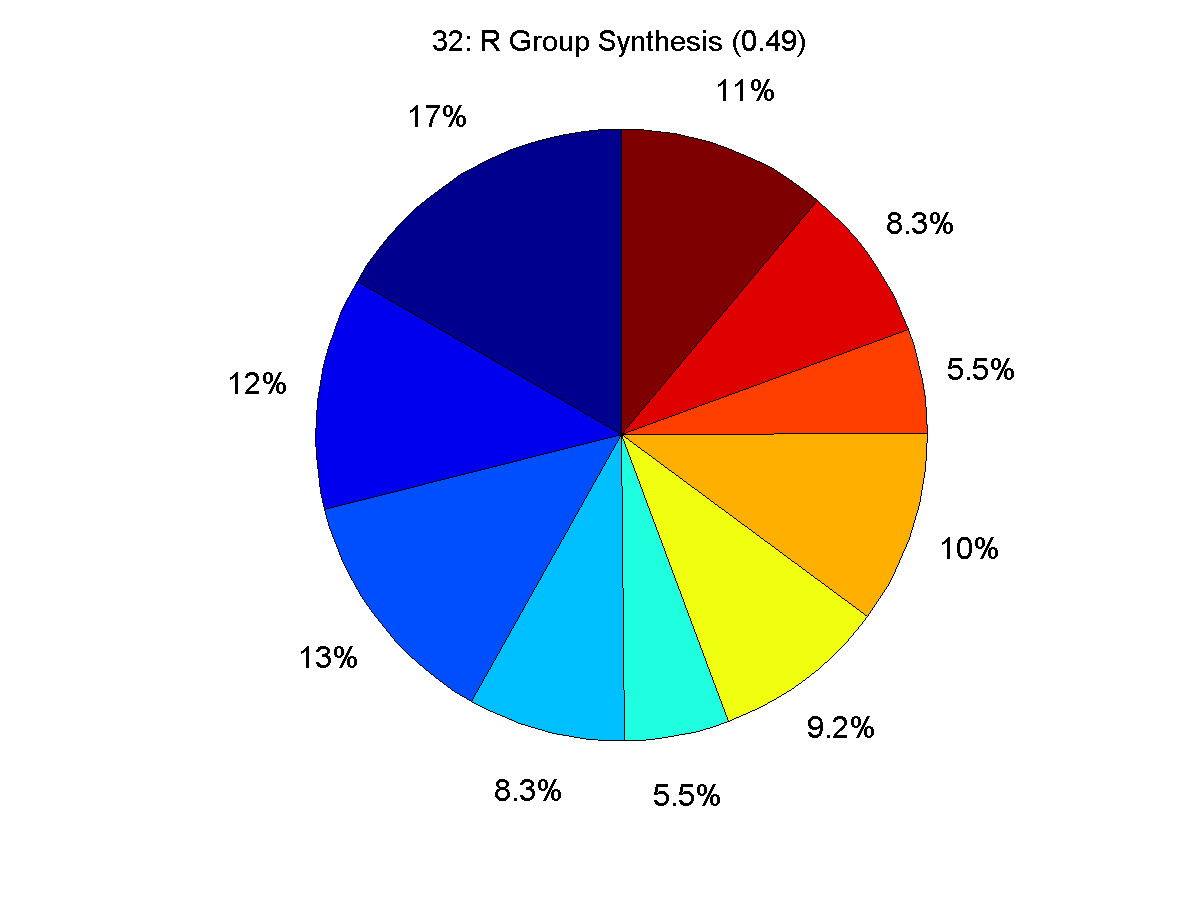

Supplement: S2 File — (ZIP) [file pone.0131875.s003.zip › MFC PieCharts/RegrEx1MFC/32RGroupSynthesis.tif]

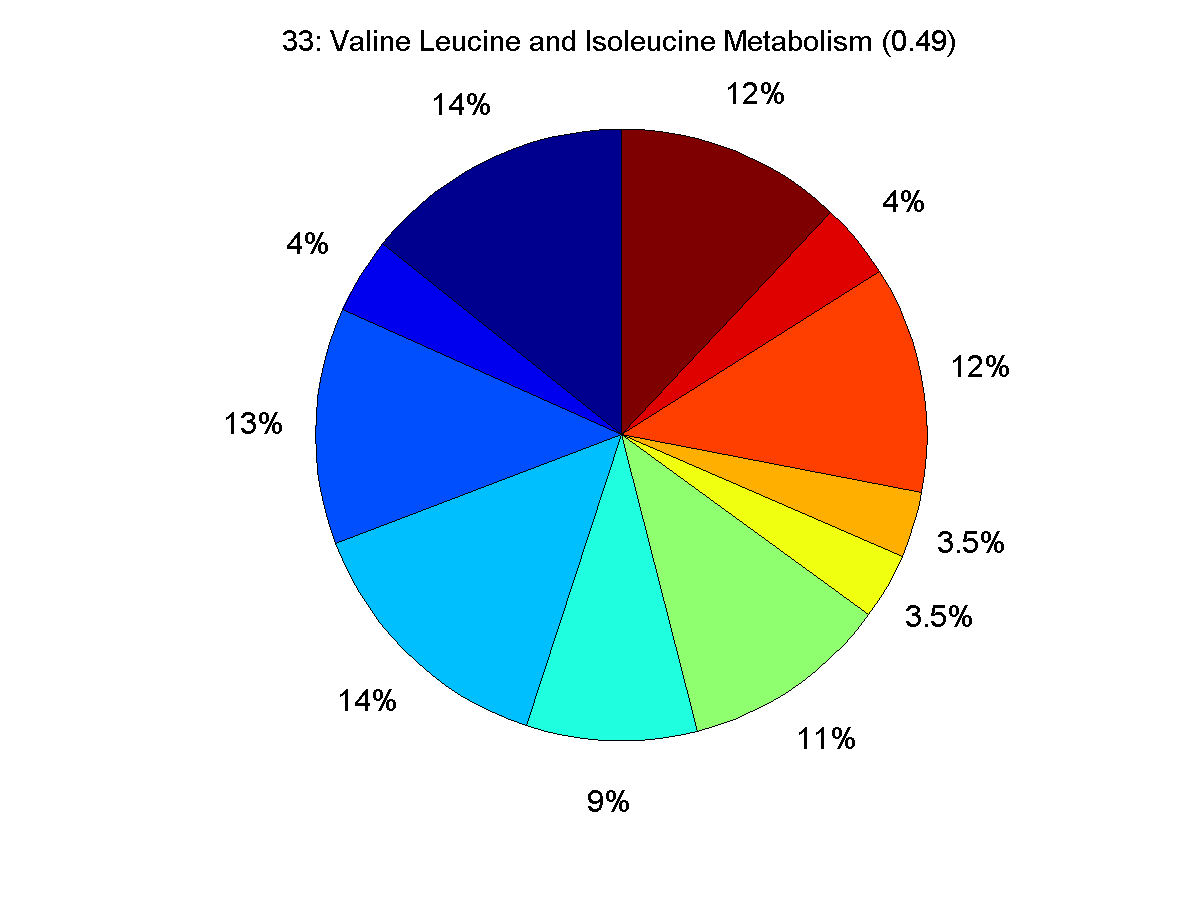

Supplement: S2 File — (ZIP) [file pone.0131875.s003.zip › MFC PieCharts/RegrEx1MFC/33ValineLeucineandIsoleucineMetabolism.tif]

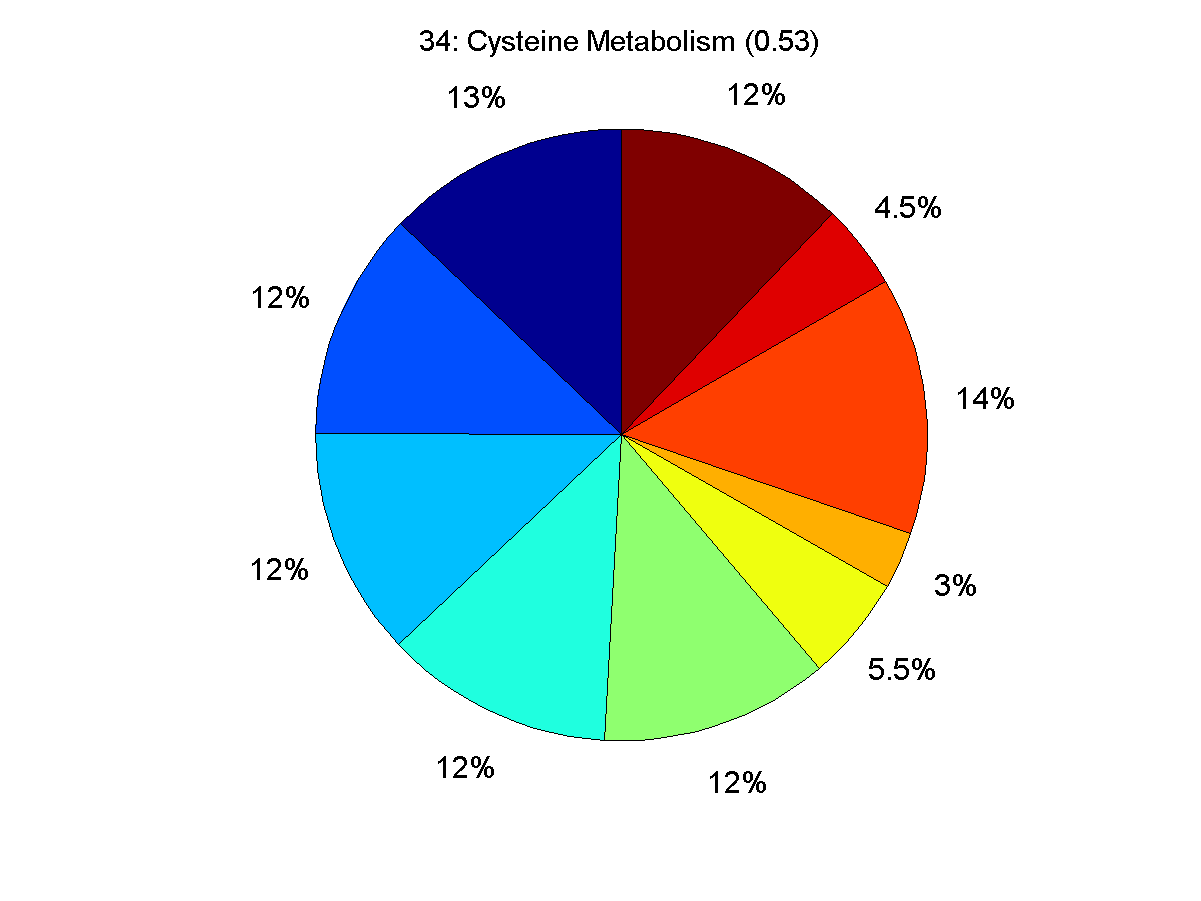

Supplement: S2 File — (ZIP) [file pone.0131875.s003.zip › MFC PieCharts/RegrEx1MFC/34CysteineMetabolism.tif]

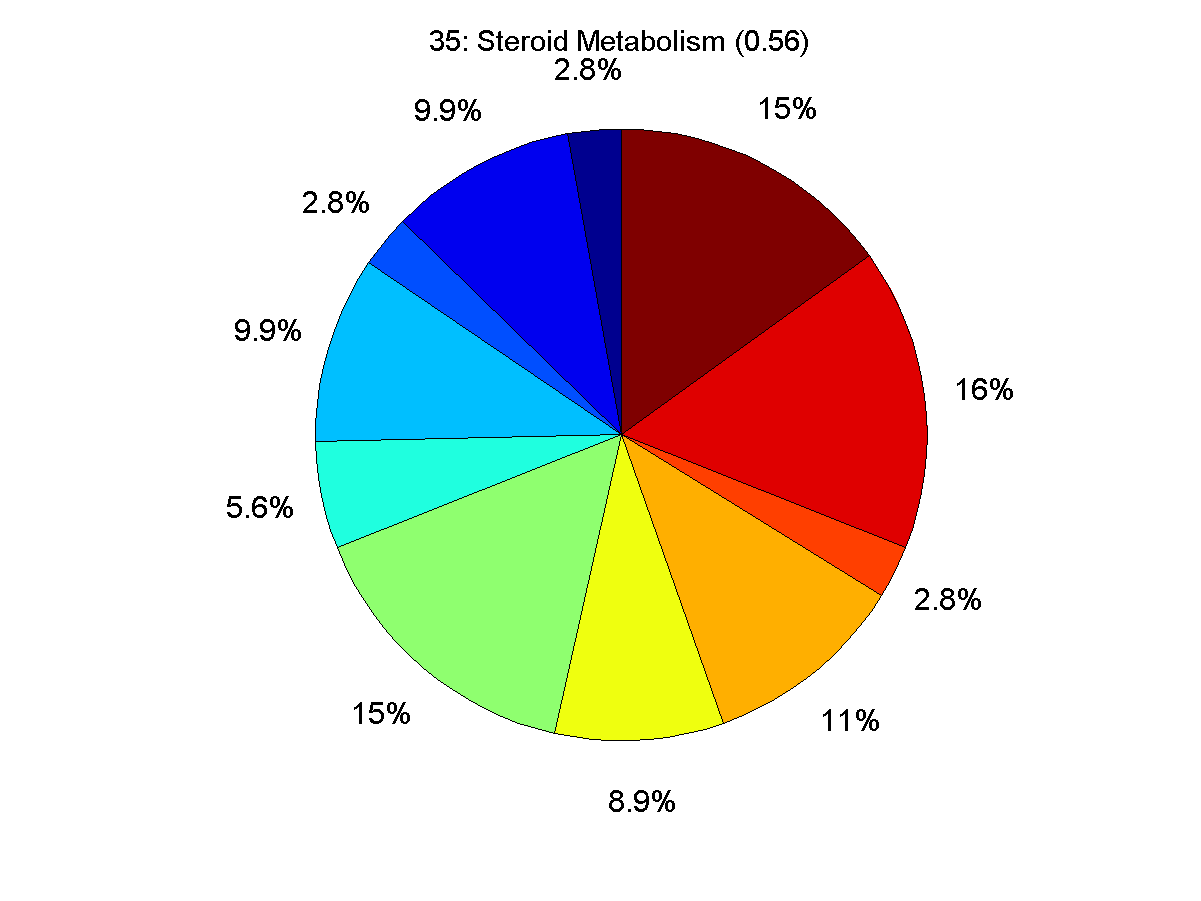

Supplement: S2 File — (ZIP) [file pone.0131875.s003.zip › MFC PieCharts/RegrEx1MFC/35SteroidMetabolism.tif]

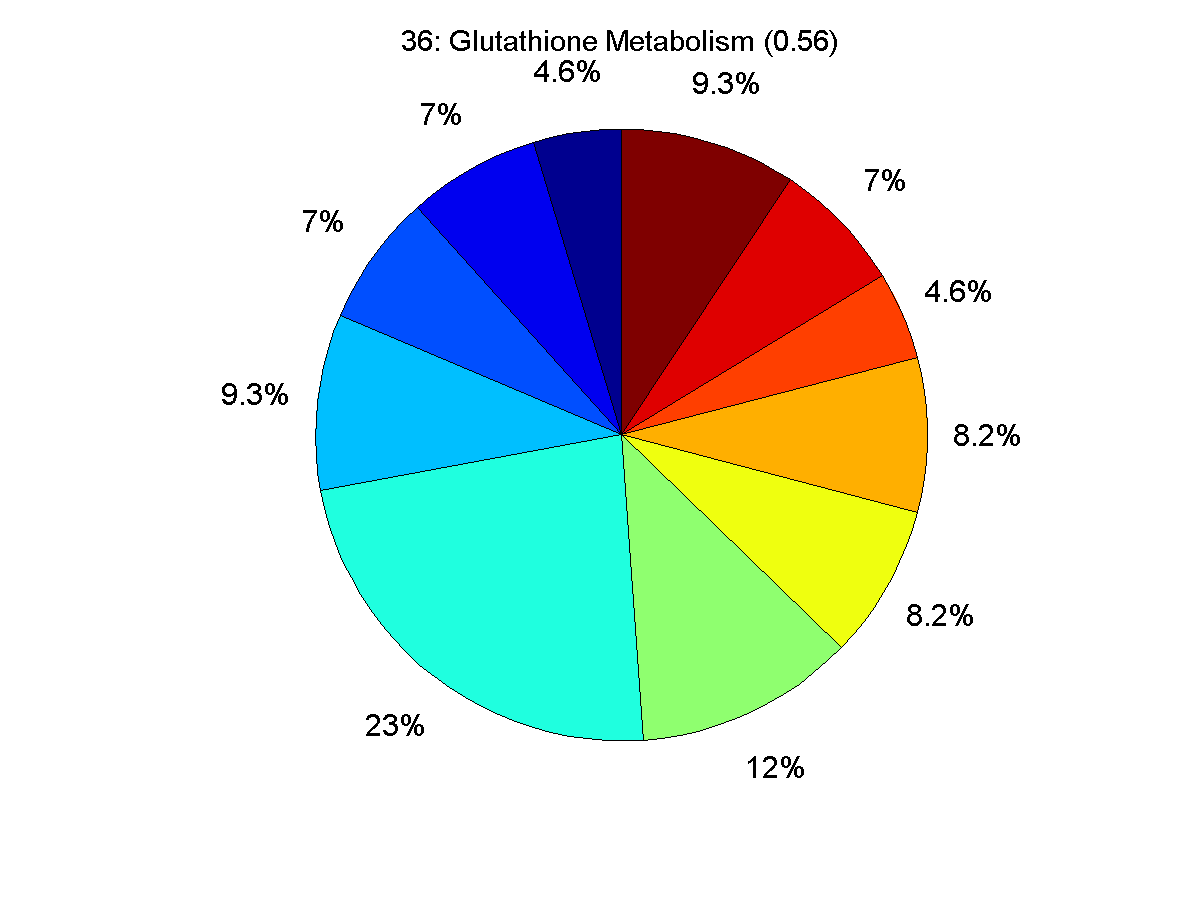

Supplement: S2 File — (ZIP) [file pone.0131875.s003.zip › MFC PieCharts/RegrEx1MFC/36GlutathioneMetabolism.tif]

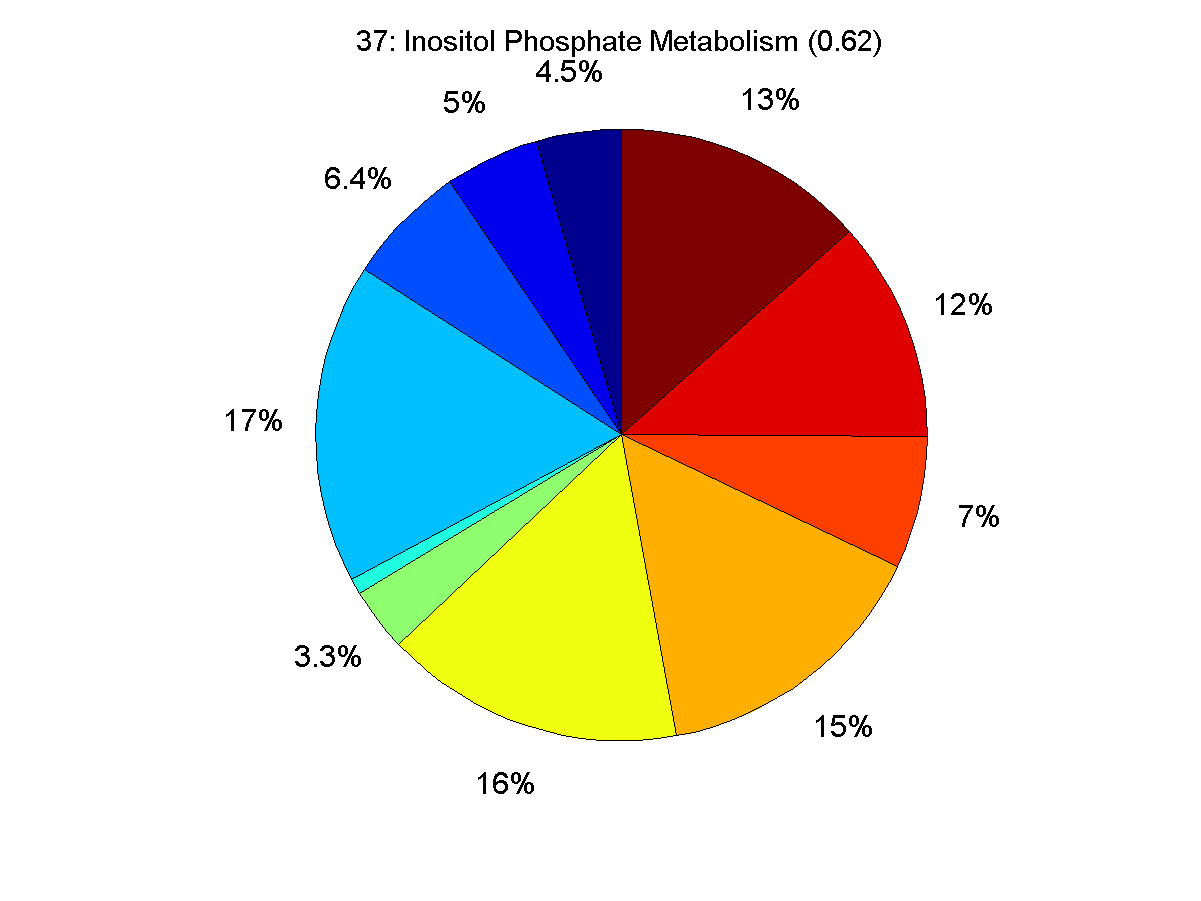

Supplement: S2 File — (ZIP) [file pone.0131875.s003.zip › MFC PieCharts/RegrEx1MFC/37InositolPhosphateMetabolism.tif]

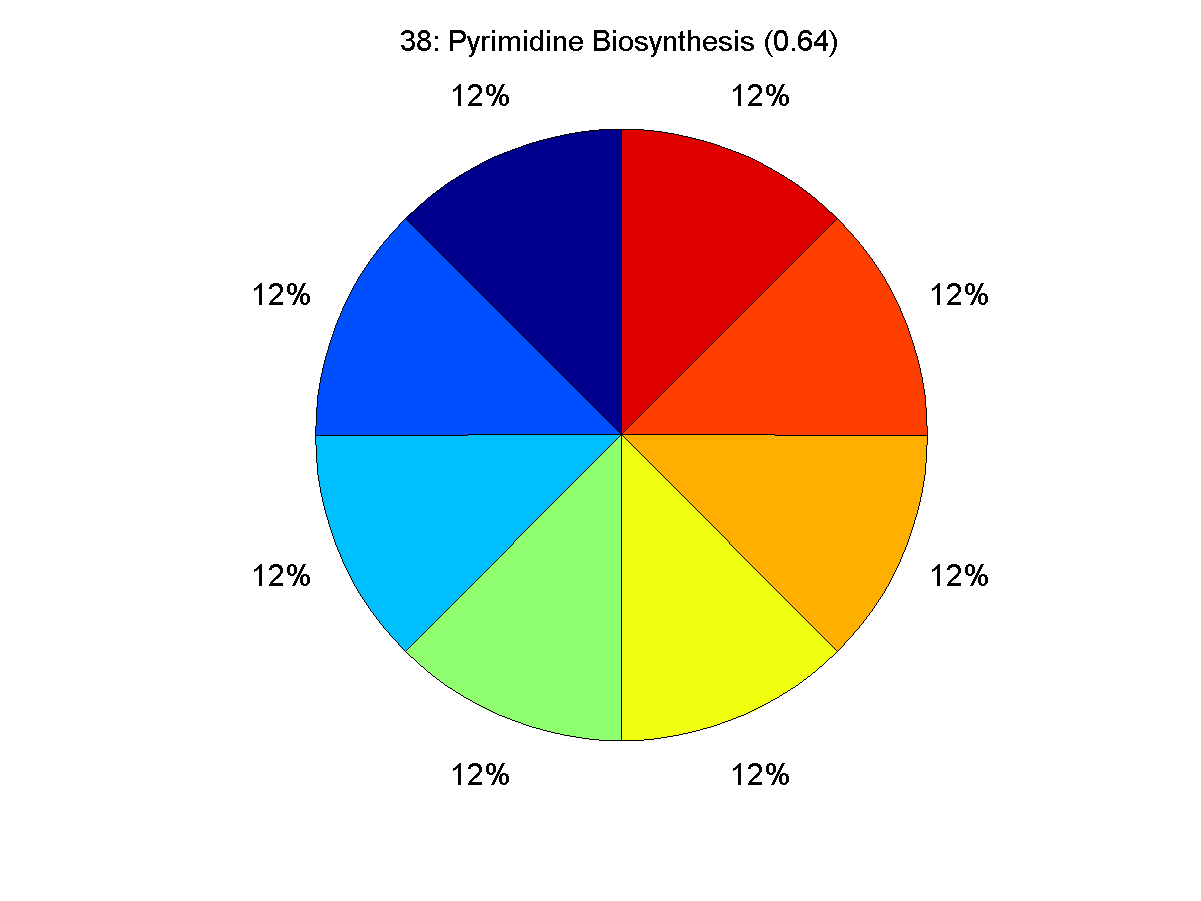

Supplement: S2 File — (ZIP) [file pone.0131875.s003.zip › MFC PieCharts/RegrEx1MFC/38PyrimidineBiosynthesis.tif]

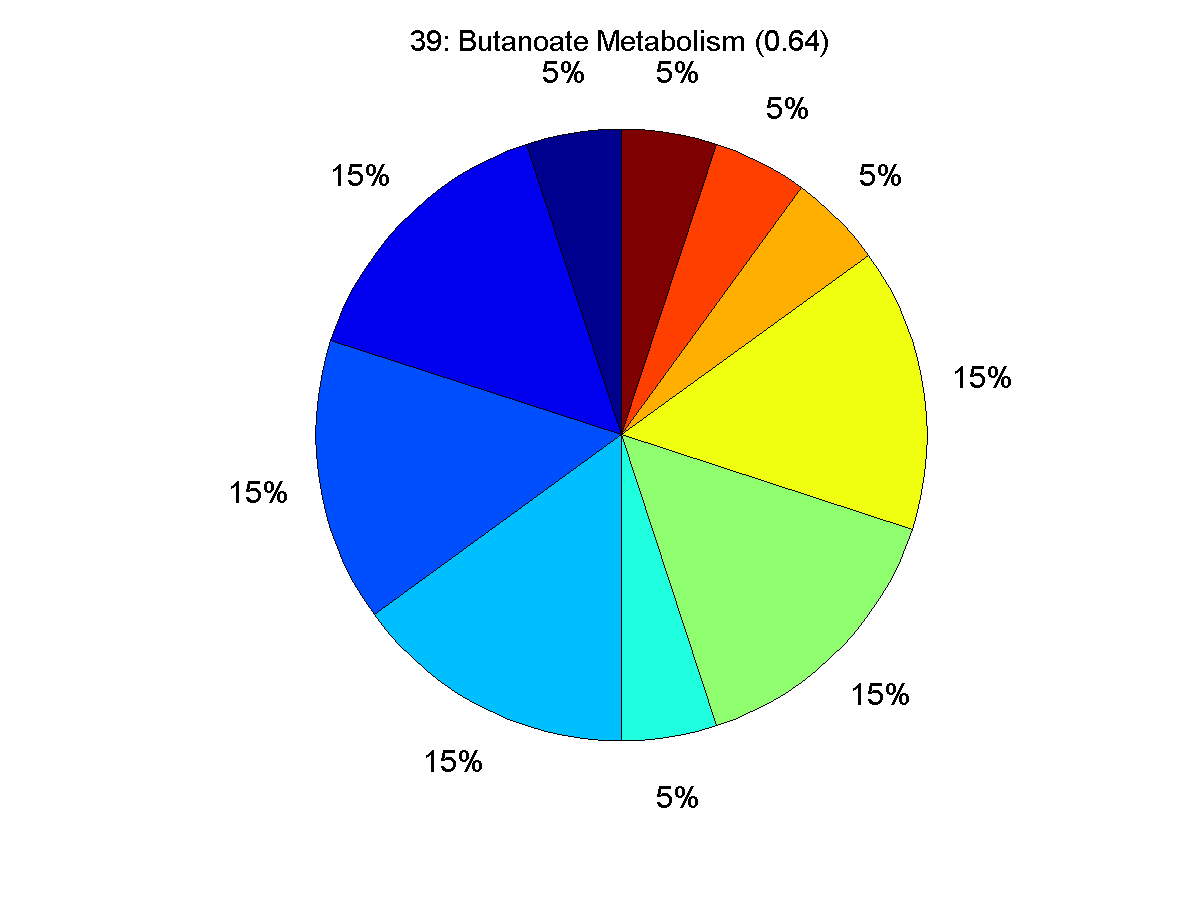

Supplement: S2 File — (ZIP) [file pone.0131875.s003.zip › MFC PieCharts/RegrEx1MFC/39ButanoateMetabolism.tif]

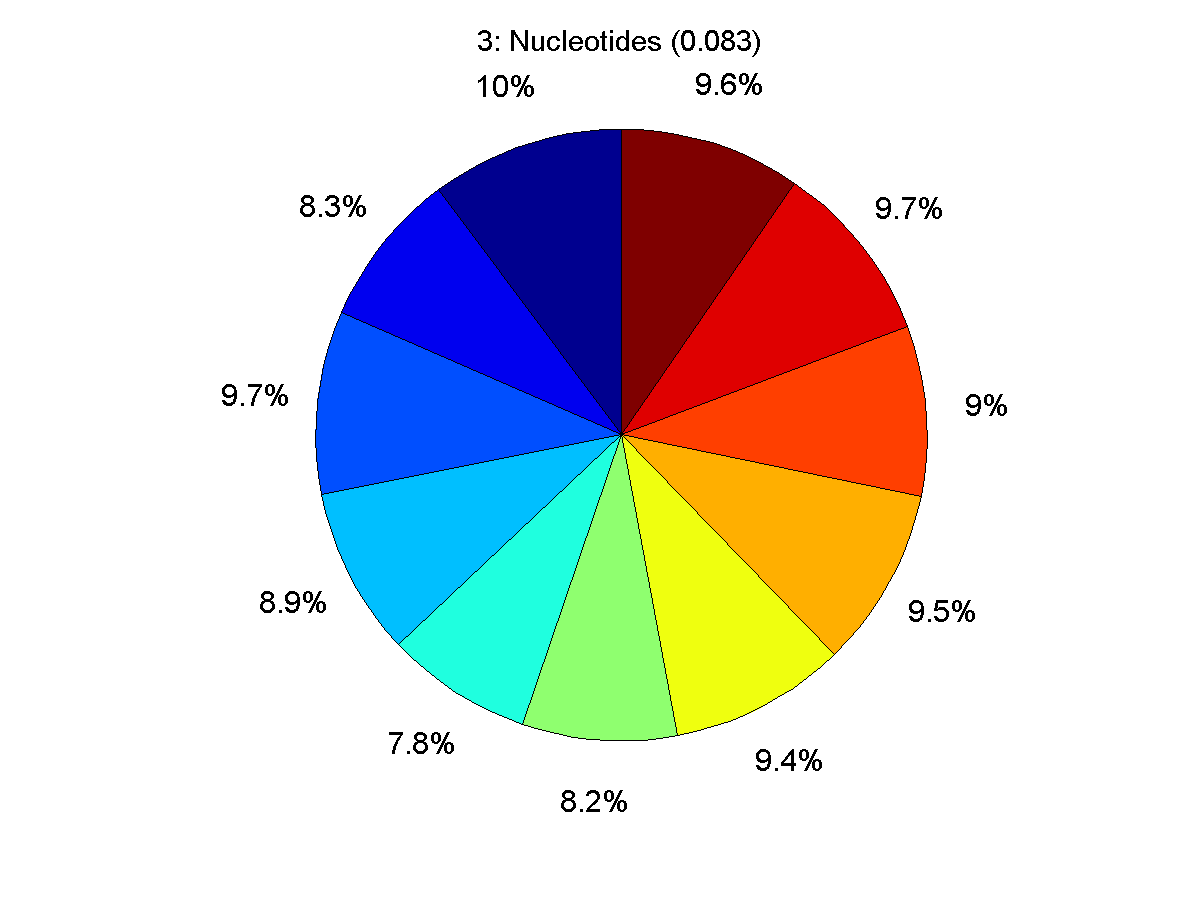

Supplement: S2 File — (ZIP) [file pone.0131875.s003.zip › MFC PieCharts/RegrEx1MFC/3Nucleotides.tif]

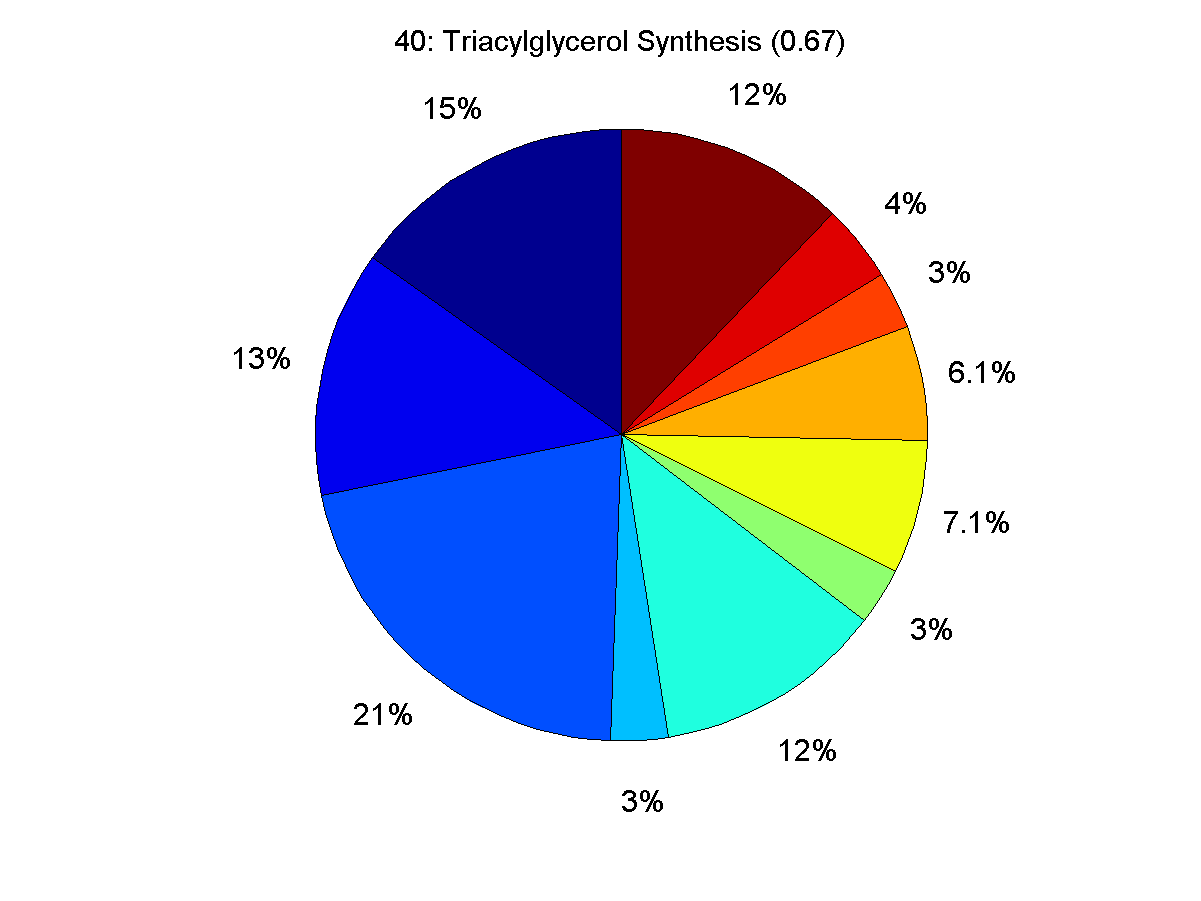

Supplement: S2 File — (ZIP) [file pone.0131875.s003.zip › MFC PieCharts/RegrEx1MFC/40TriacylglycerolSynthesis.tif]

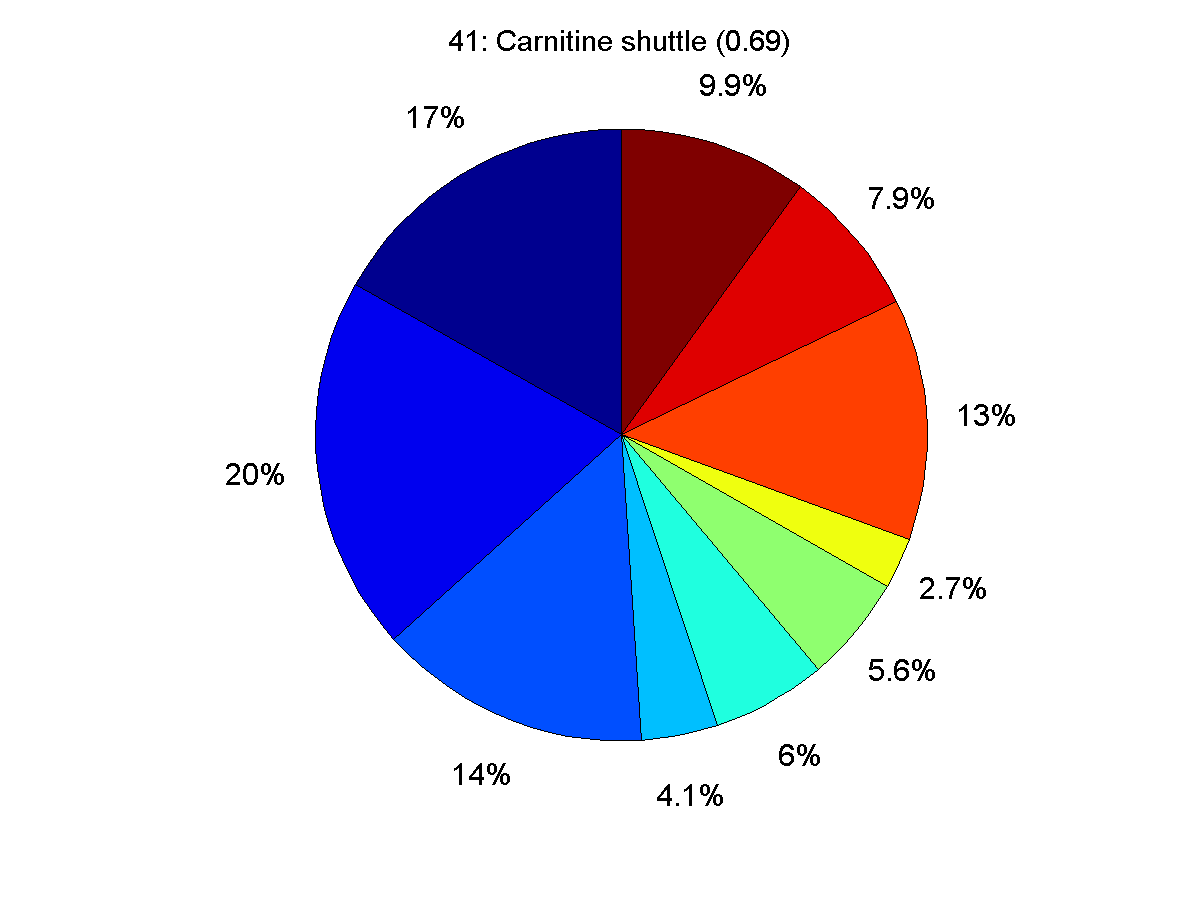

Supplement: S2 File — (ZIP) [file pone.0131875.s003.zip › MFC PieCharts/RegrEx1MFC/41Carnitineshuttle.tif]

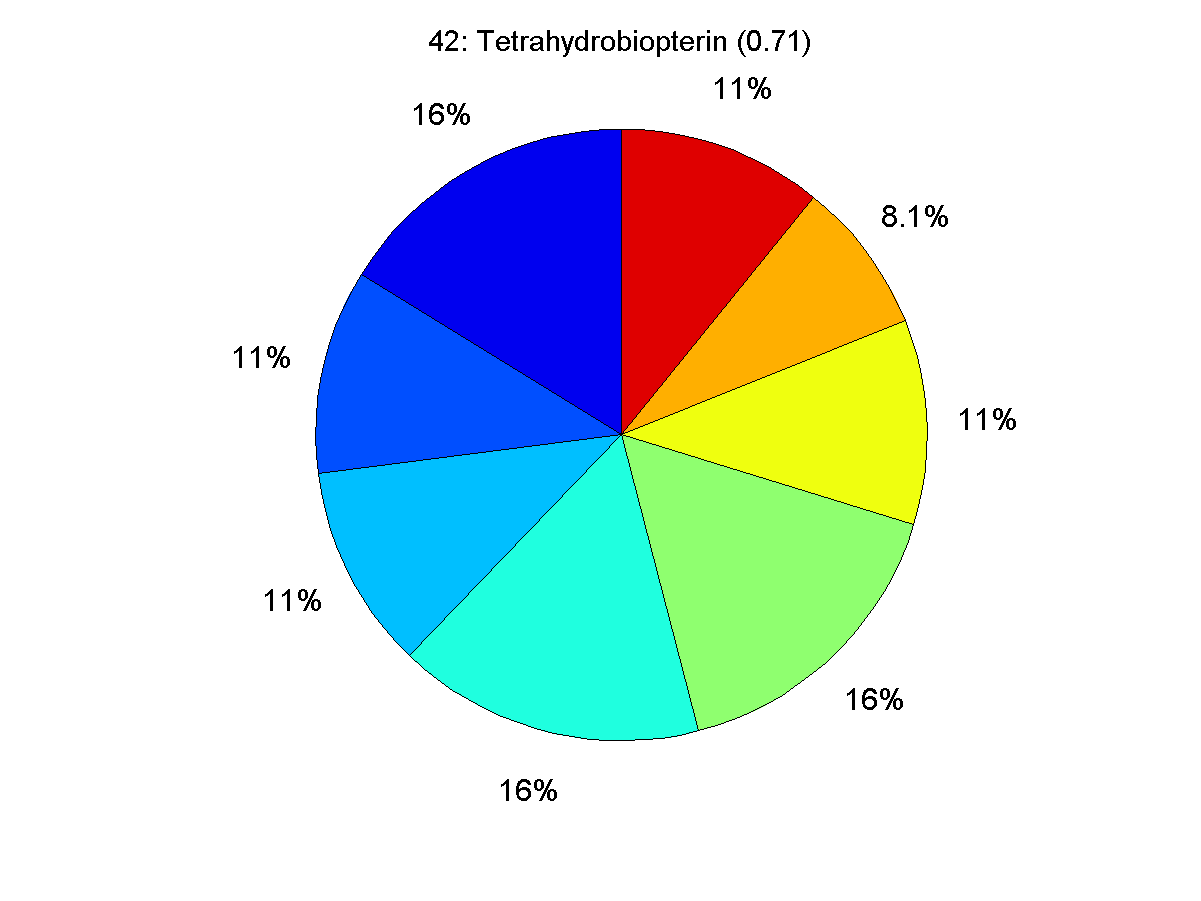

Supplement: S2 File — (ZIP) [file pone.0131875.s003.zip › MFC PieCharts/RegrEx1MFC/42Tetrahydrobiopterin.tif]

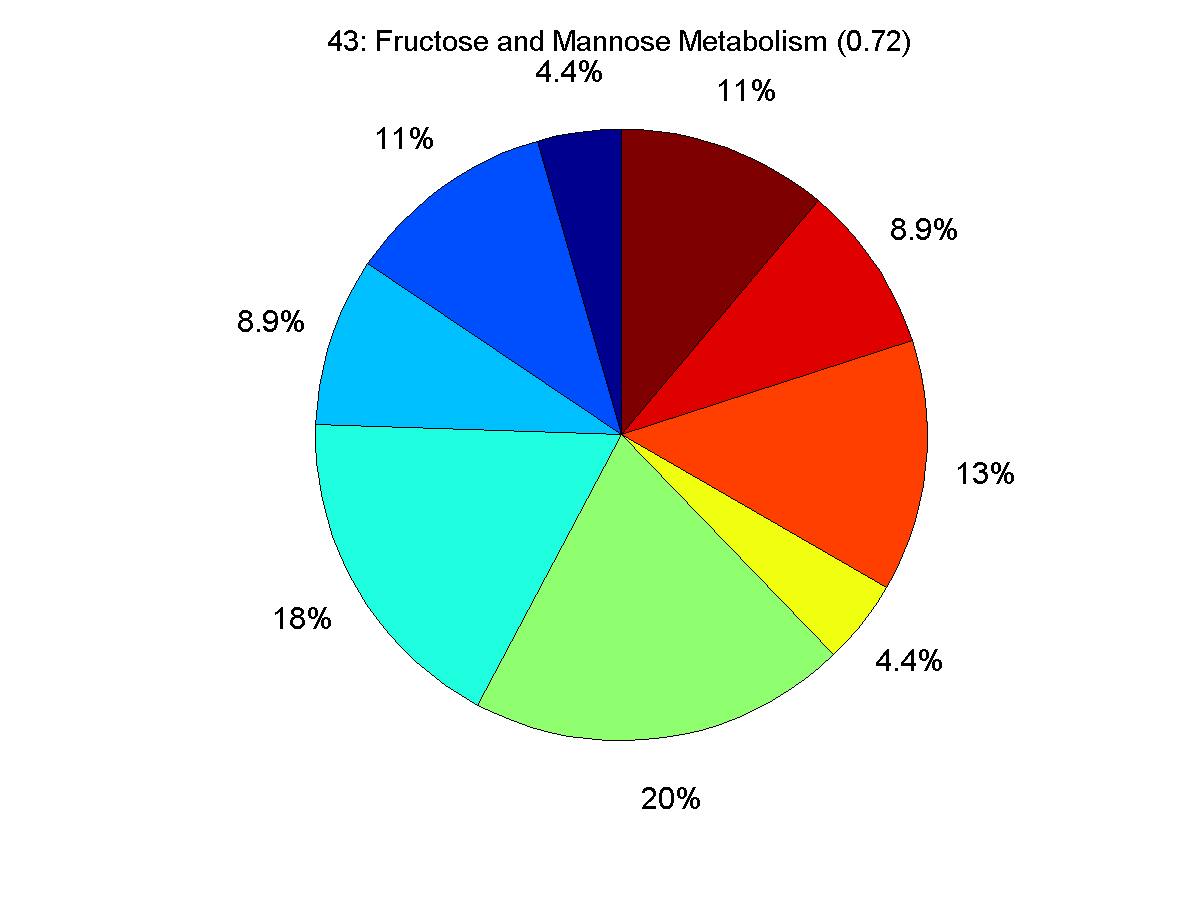

Supplement: S2 File — (ZIP) [file pone.0131875.s003.zip › MFC PieCharts/RegrEx1MFC/43FructoseandMannoseMetabolism.tif]

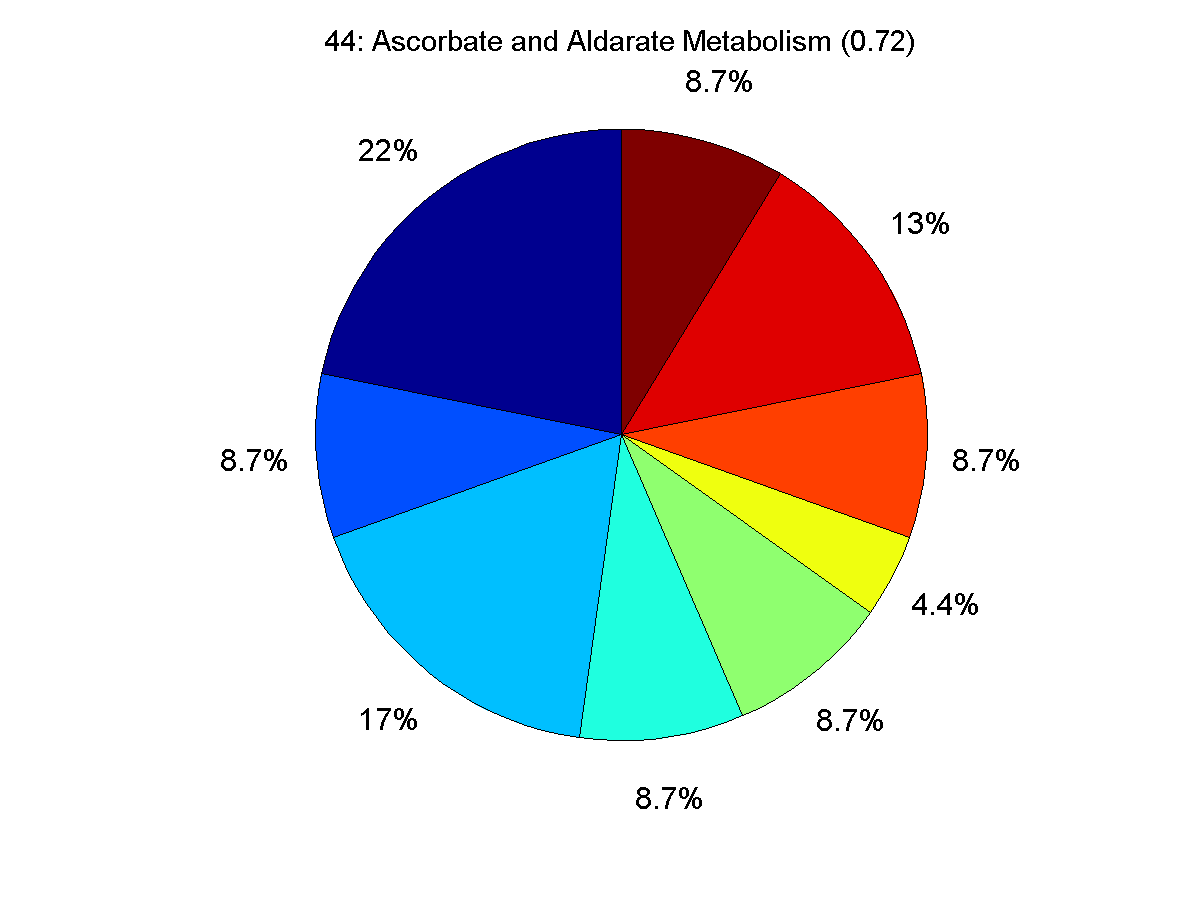

Supplement: S2 File — (ZIP) [file pone.0131875.s003.zip › MFC PieCharts/RegrEx1MFC/44AscorbateandAldarateMetabolism.tif]

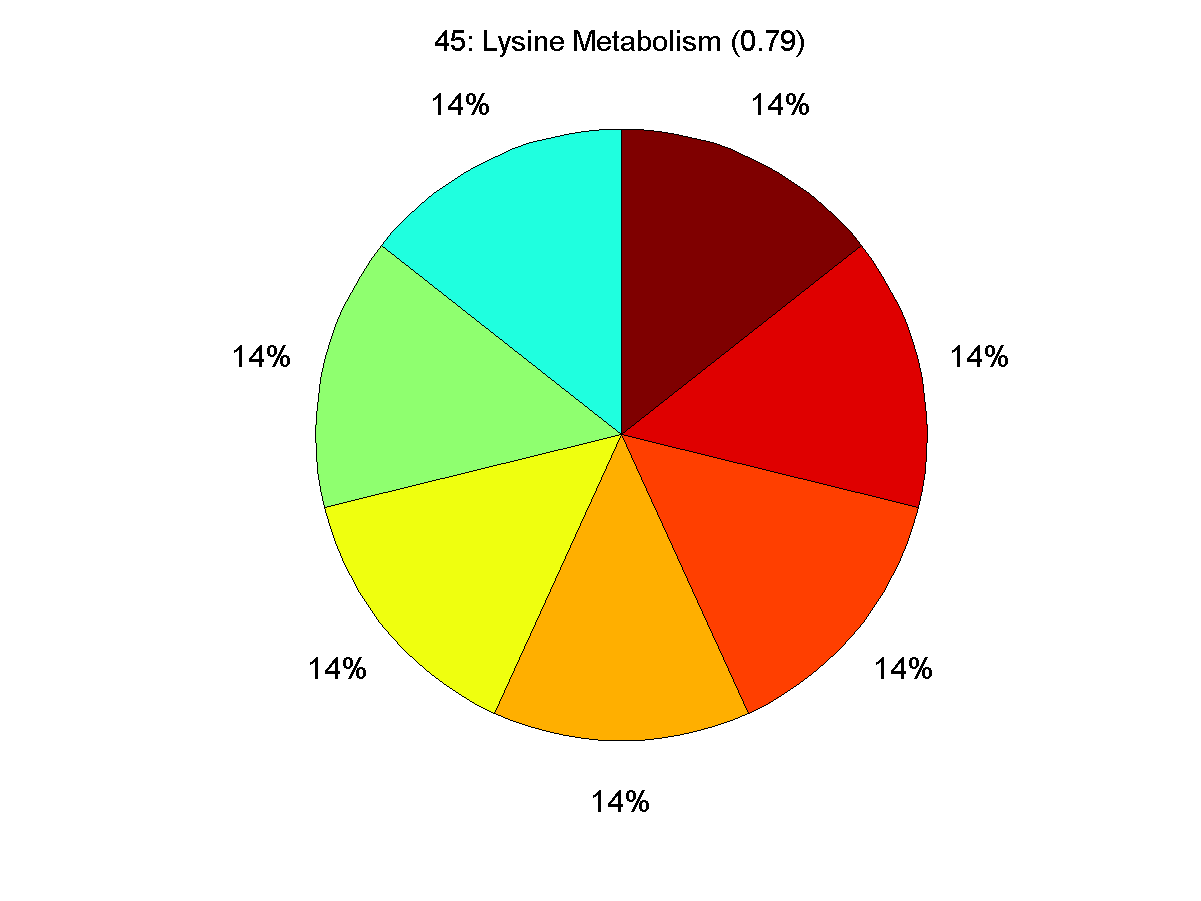

Supplement: S2 File — (ZIP) [file pone.0131875.s003.zip › MFC PieCharts/RegrEx1MFC/45LysineMetabolism.tif]

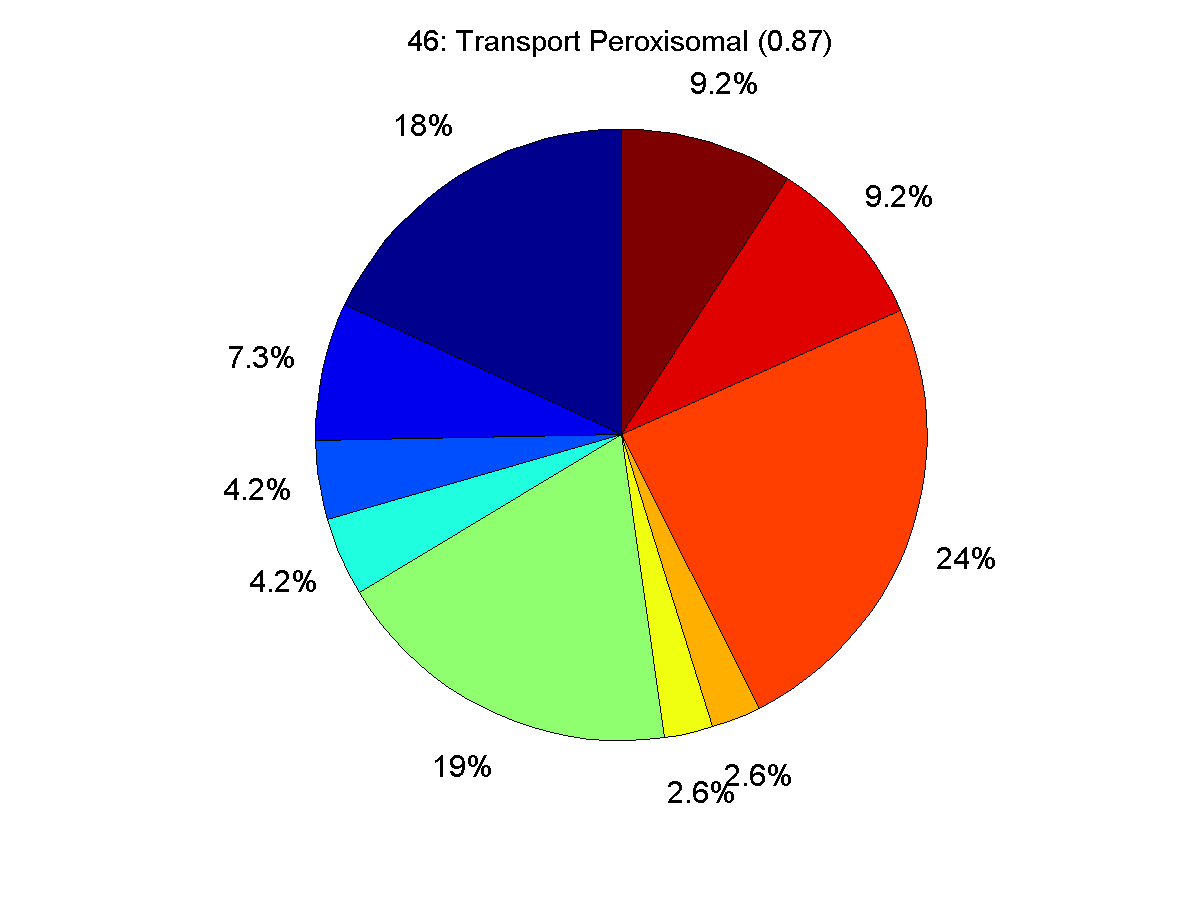

Supplement: S2 File — (ZIP) [file pone.0131875.s003.zip › MFC PieCharts/RegrEx1MFC/46TransportPeroxisomal.tif]

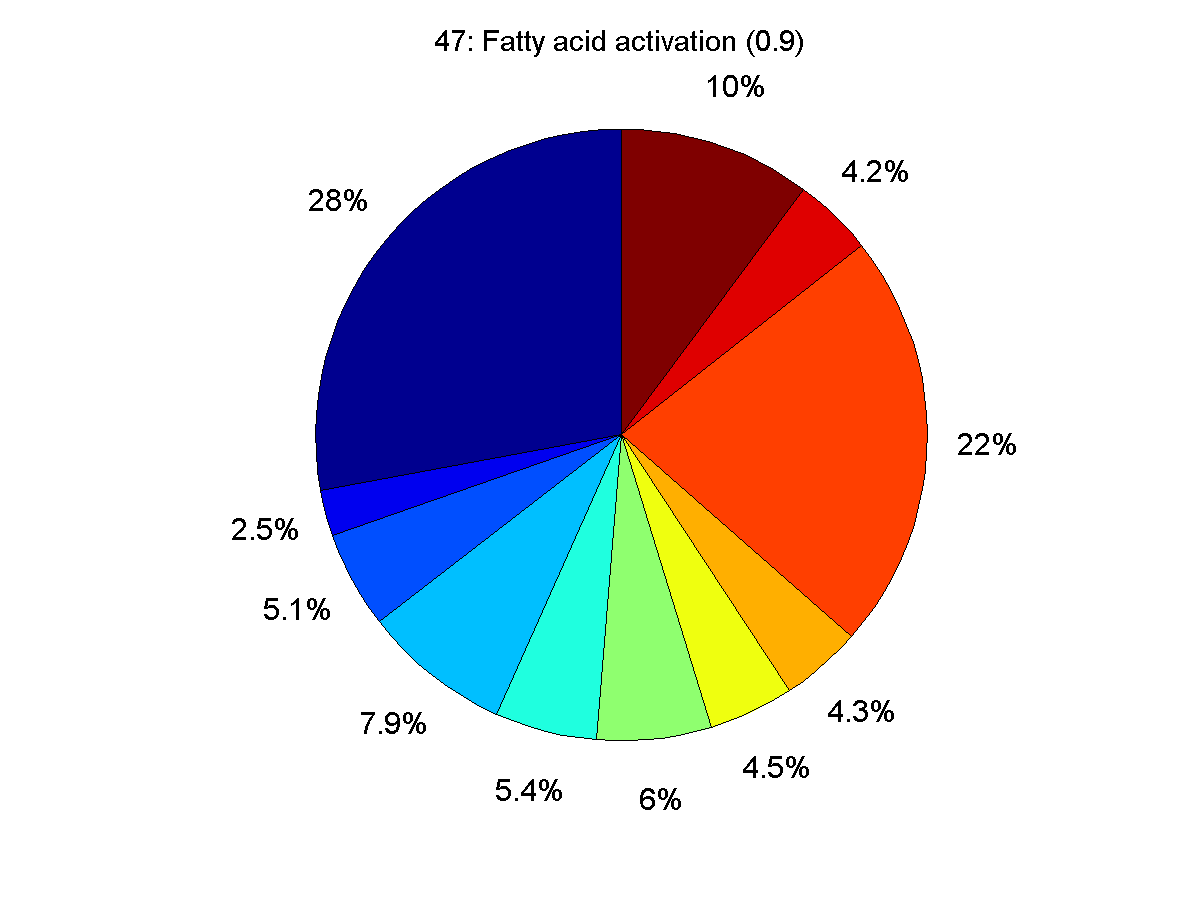

Supplement: S2 File — (ZIP) [file pone.0131875.s003.zip › MFC PieCharts/RegrEx1MFC/47Fattyacidactivation.tif]

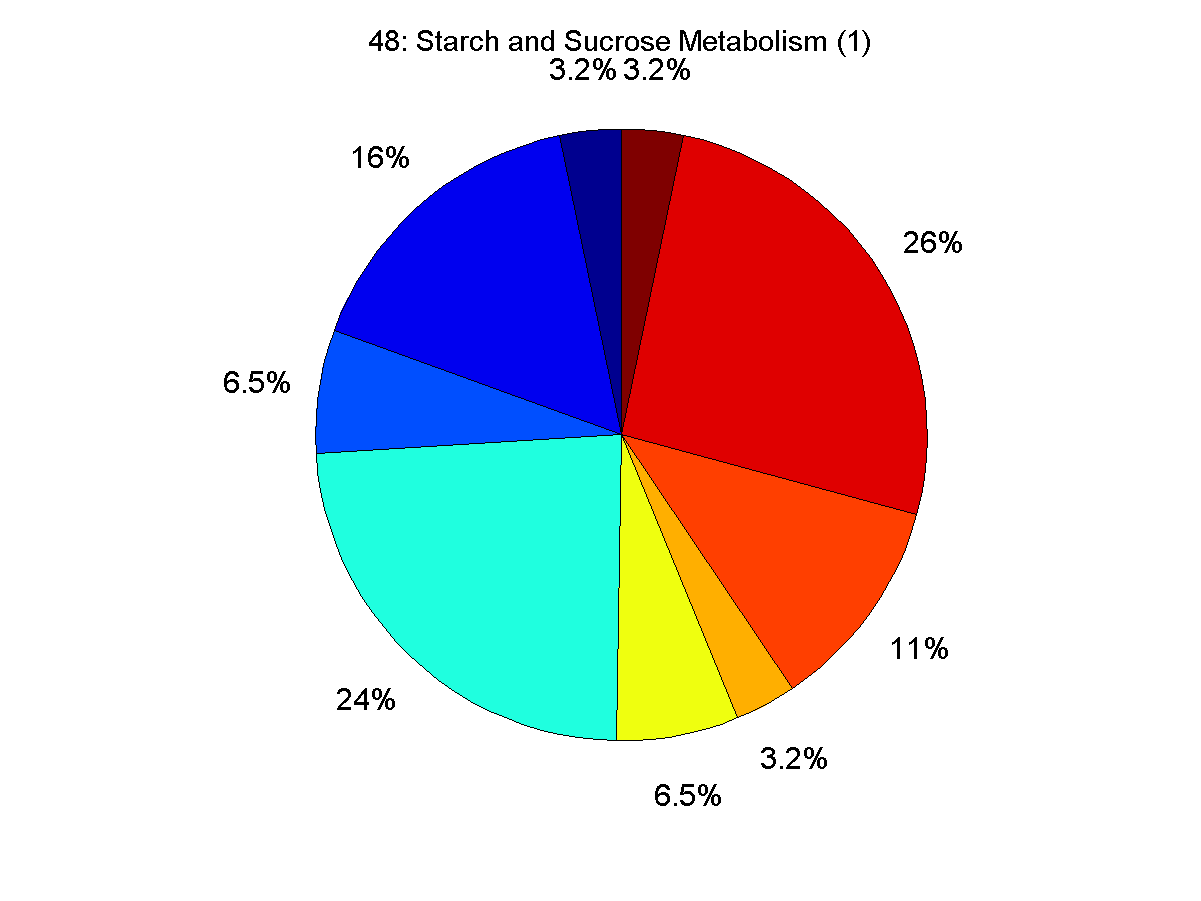

Supplement: S2 File — (ZIP) [file pone.0131875.s003.zip › MFC PieCharts/RegrEx1MFC/48StarchandSucroseMetabolism.tif]

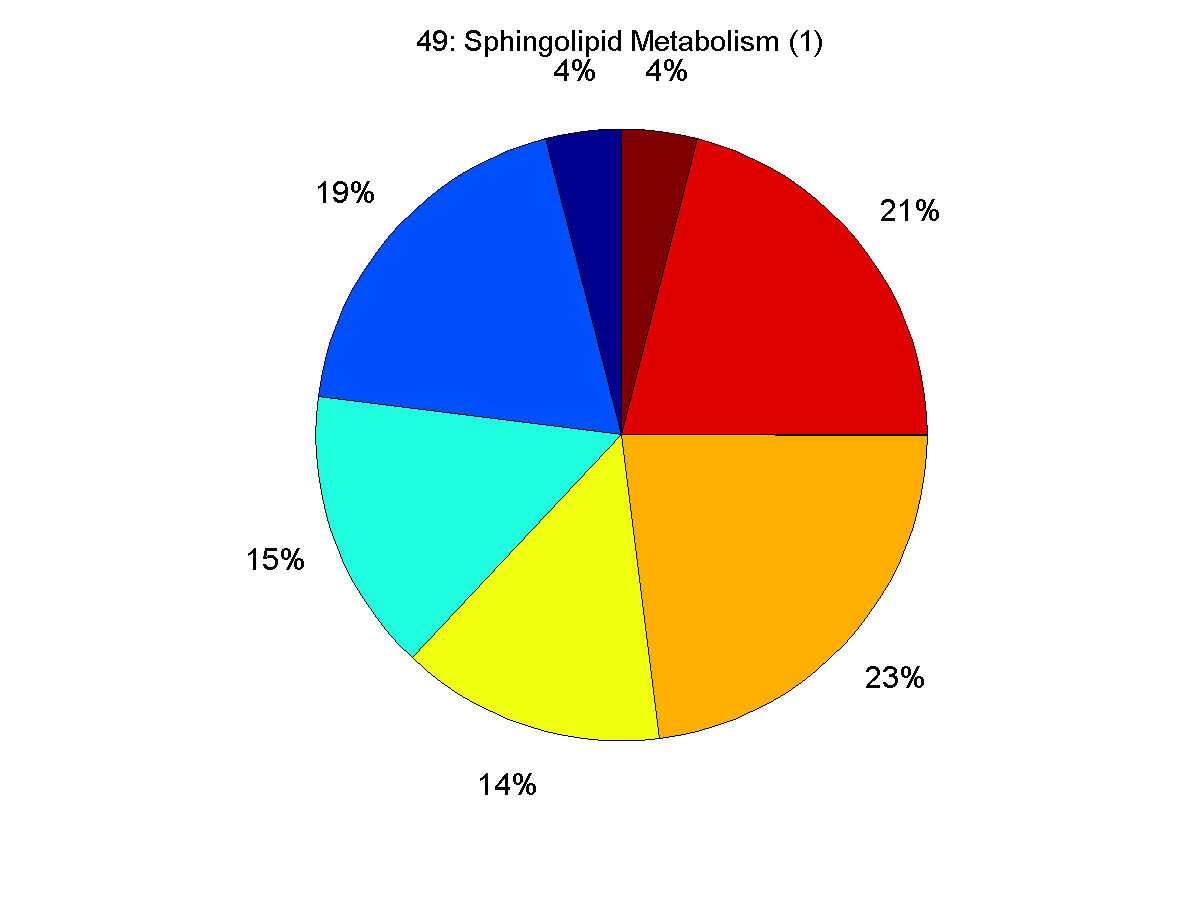

Supplement: S2 File — (ZIP) [file pone.0131875.s003.zip › MFC PieCharts/RegrEx1MFC/49SphingolipidMetabolism.tif]

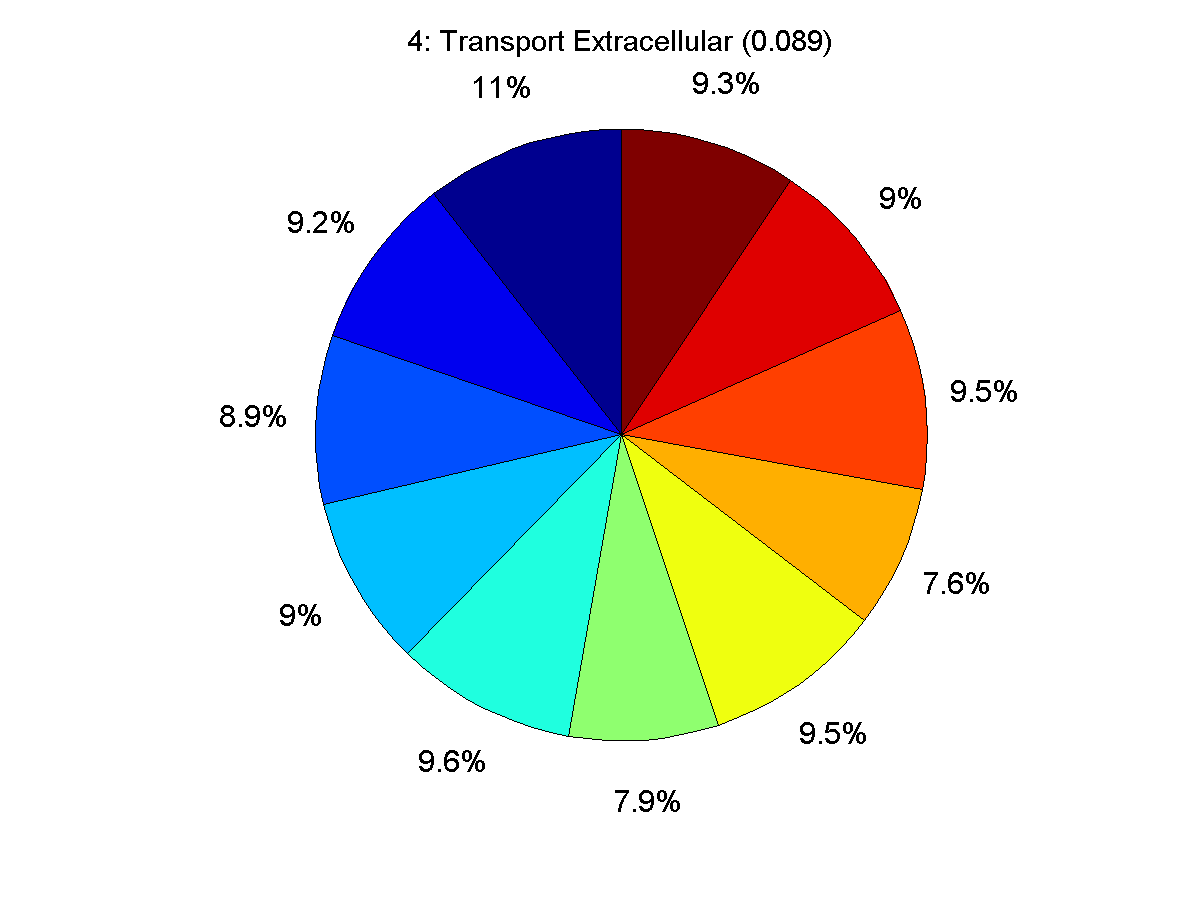

Supplement: S2 File — (ZIP) [file pone.0131875.s003.zip › MFC PieCharts/RegrEx1MFC/4TransportExtracellular.tif]

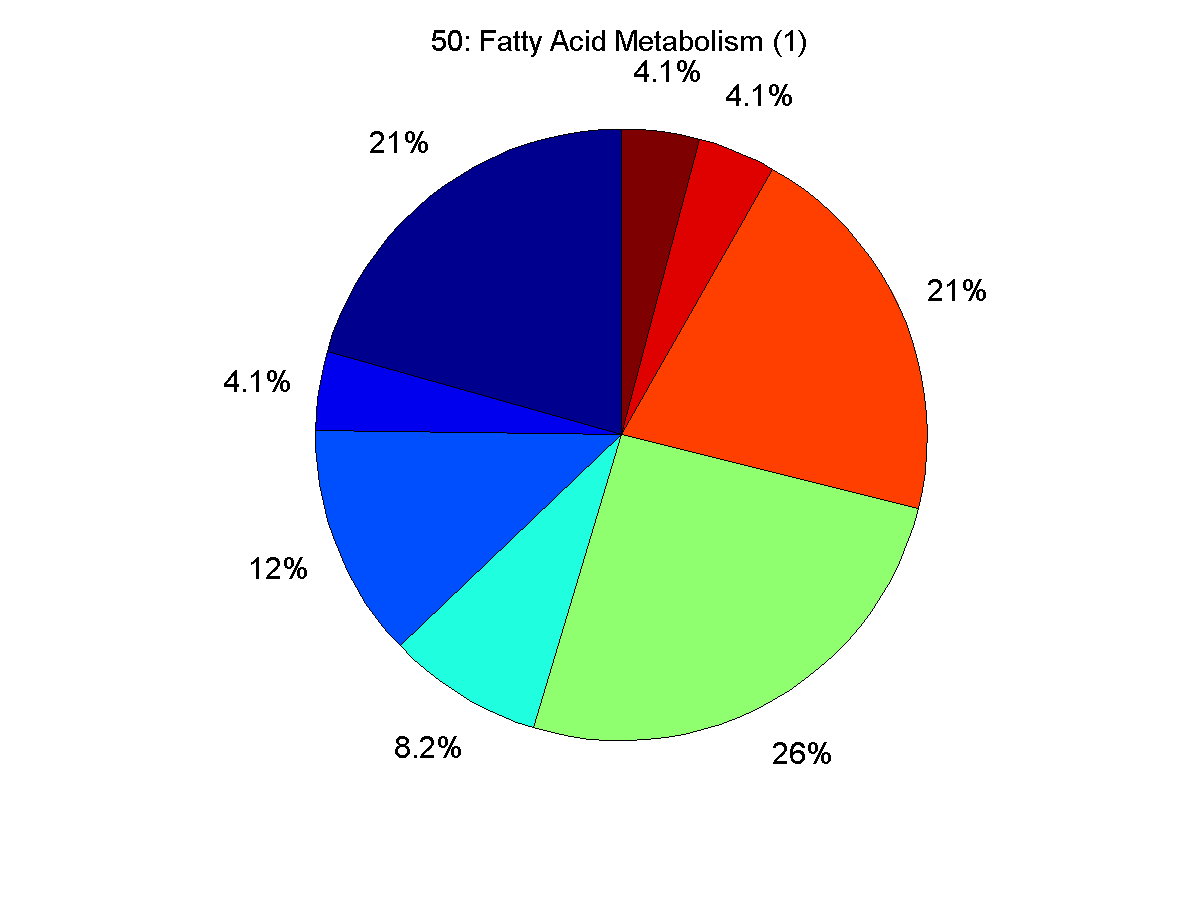

Supplement: S2 File — (ZIP) [file pone.0131875.s003.zip › MFC PieCharts/RegrEx1MFC/50FattyAcidMetabolism.tif]

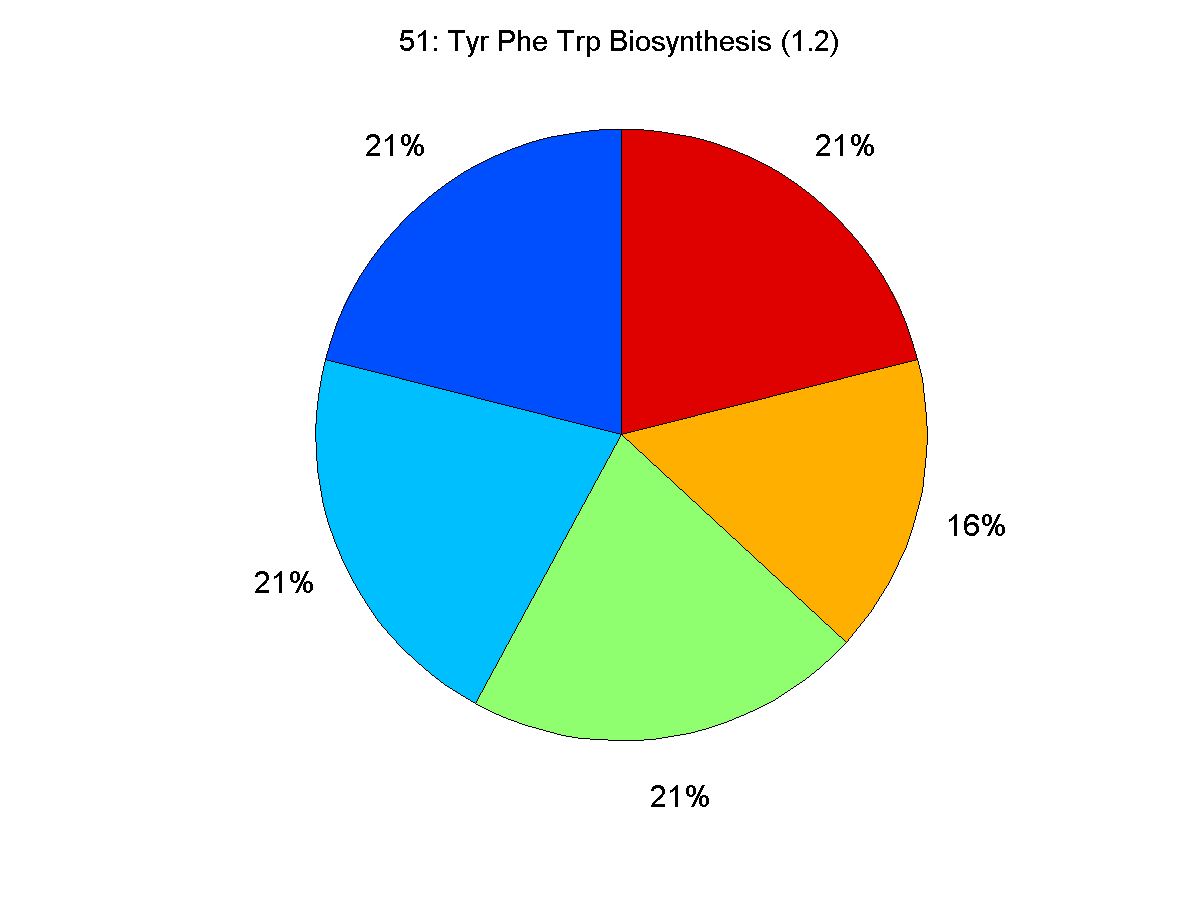

Supplement: S2 File — (ZIP) [file pone.0131875.s003.zip › MFC PieCharts/RegrEx1MFC/51TyrPheTrpBiosynthesis.tif]

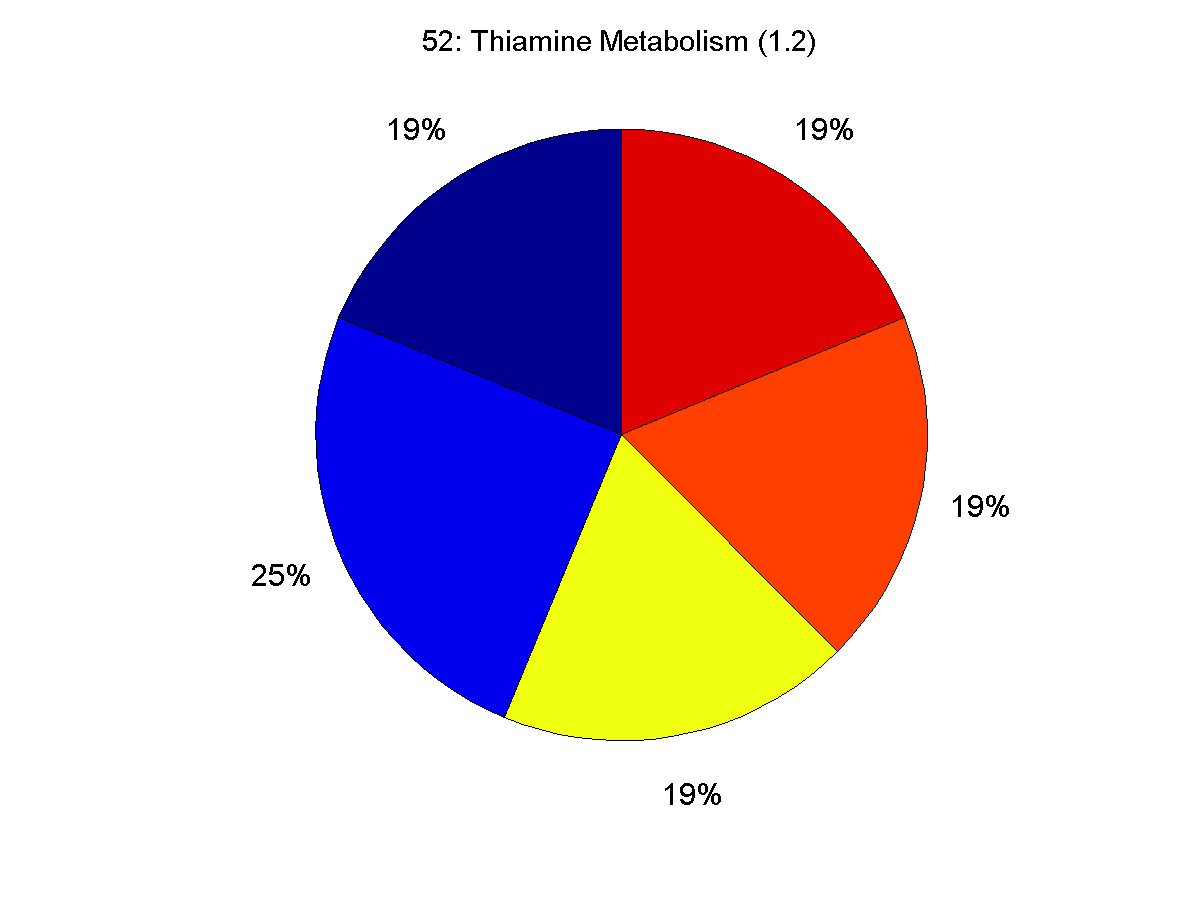

Supplement: S2 File — (ZIP) [file pone.0131875.s003.zip › MFC PieCharts/RegrEx1MFC/52ThiamineMetabolism.tif]

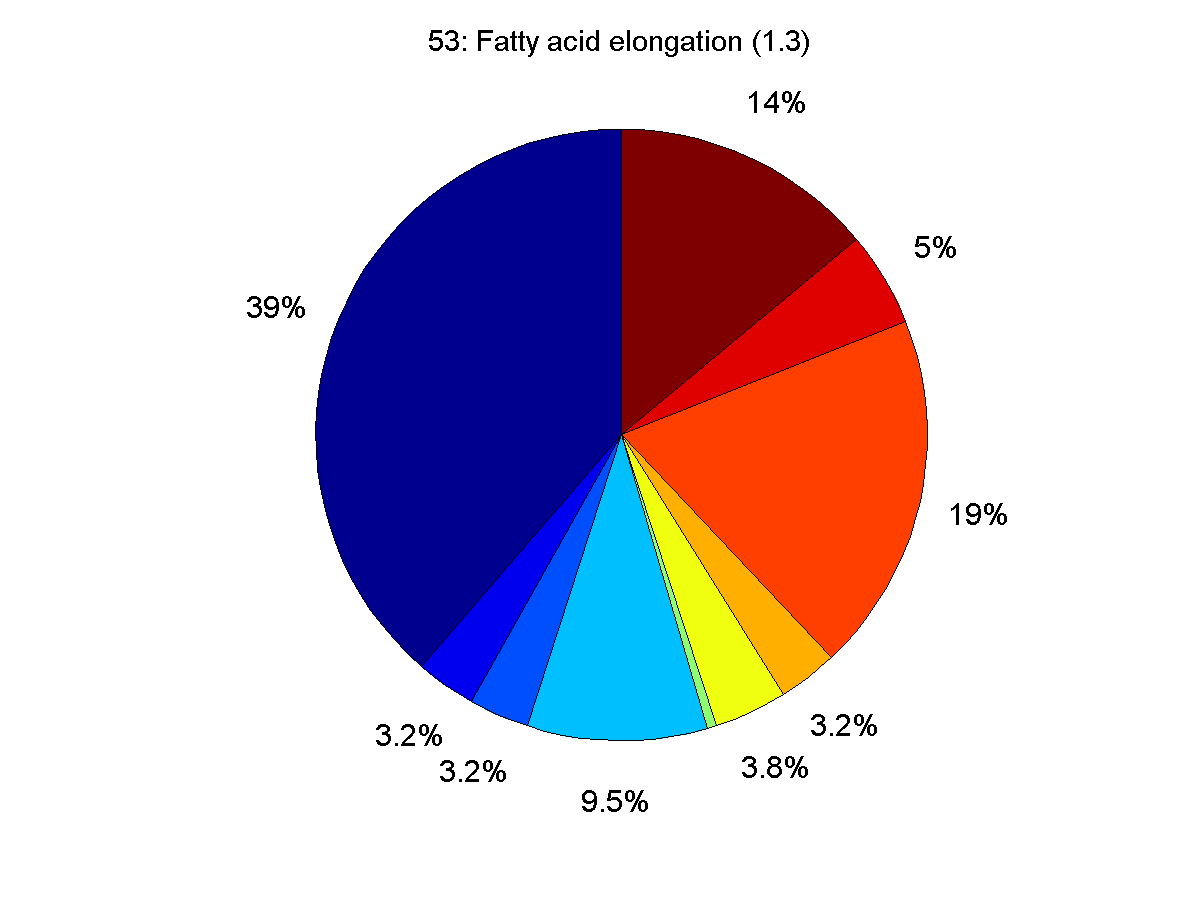

Supplement: S2 File — (ZIP) [file pone.0131875.s003.zip › MFC PieCharts/RegrEx1MFC/53Fattyacidelongation.tif]

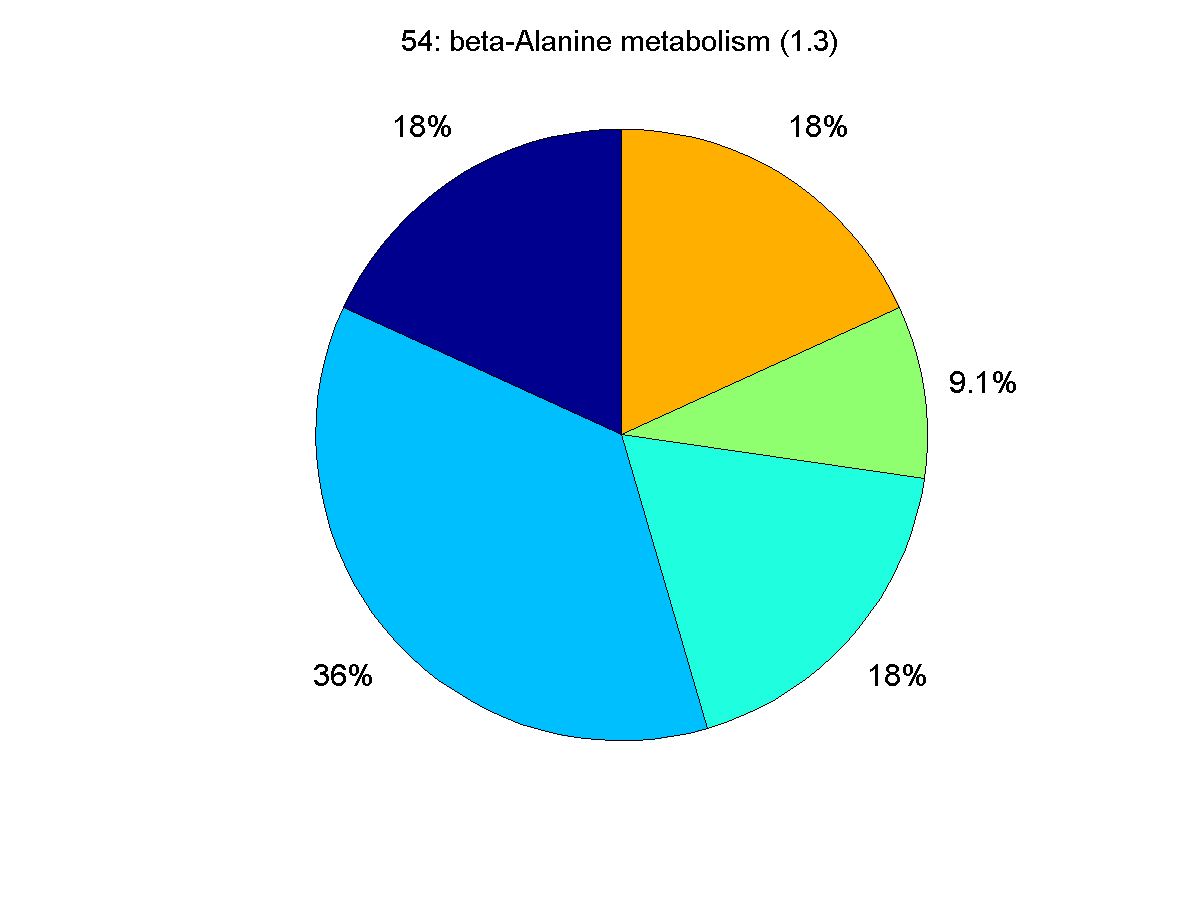

Supplement: S2 File — (ZIP) [file pone.0131875.s003.zip › MFC PieCharts/RegrEx1MFC/54beta-Alaninemetabolism.tif]

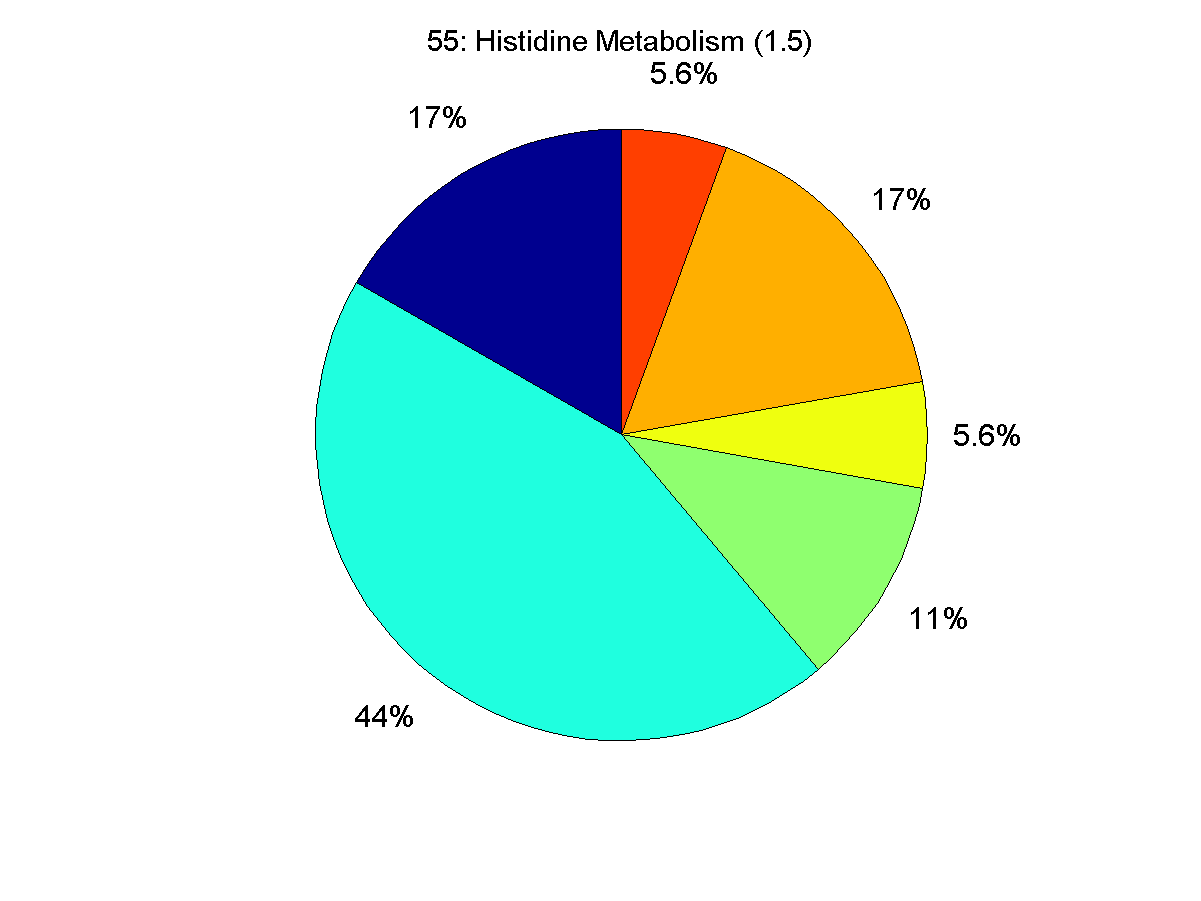

Supplement: S2 File — (ZIP) [file pone.0131875.s003.zip › MFC PieCharts/RegrEx1MFC/55HistidineMetabolism.tif]

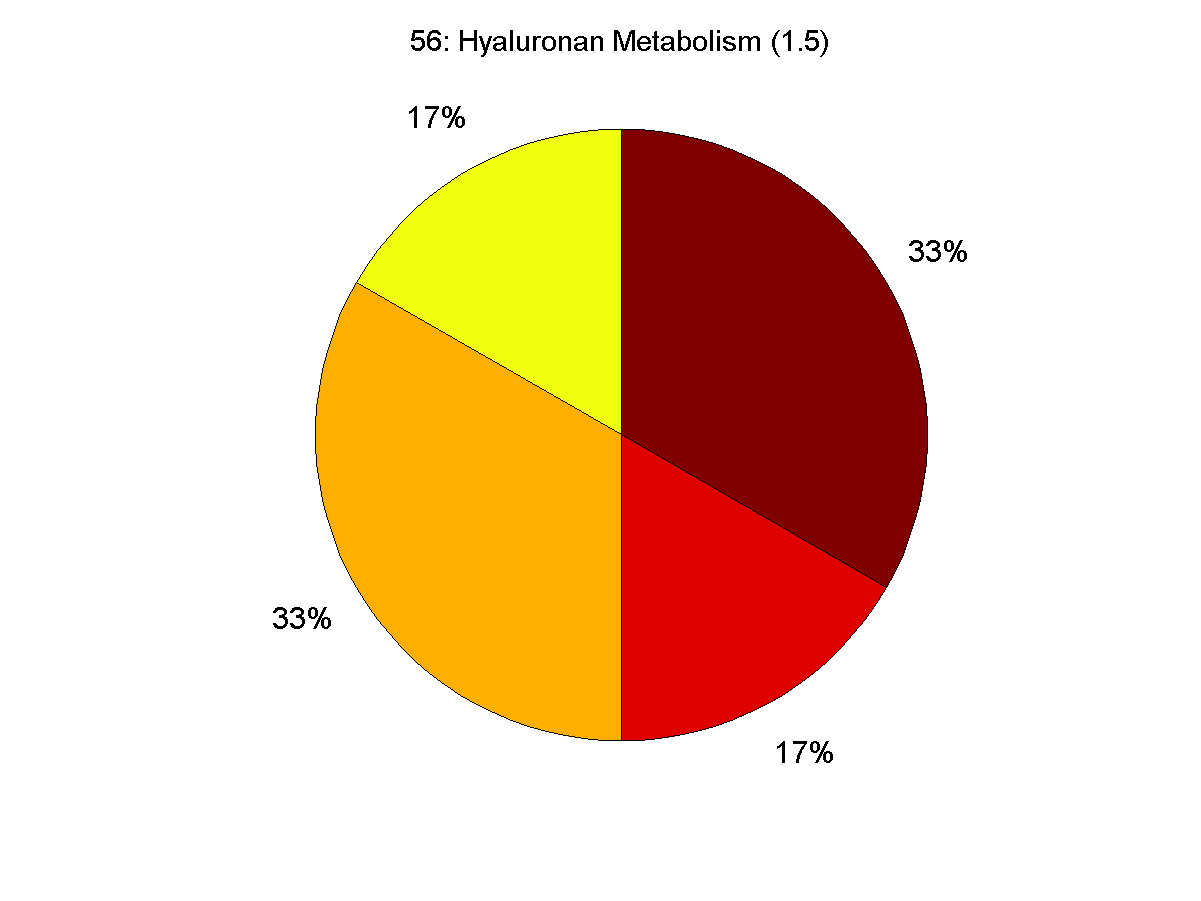

Supplement: S2 File — (ZIP) [file pone.0131875.s003.zip › MFC PieCharts/RegrEx1MFC/56HyaluronanMetabolism.tif]

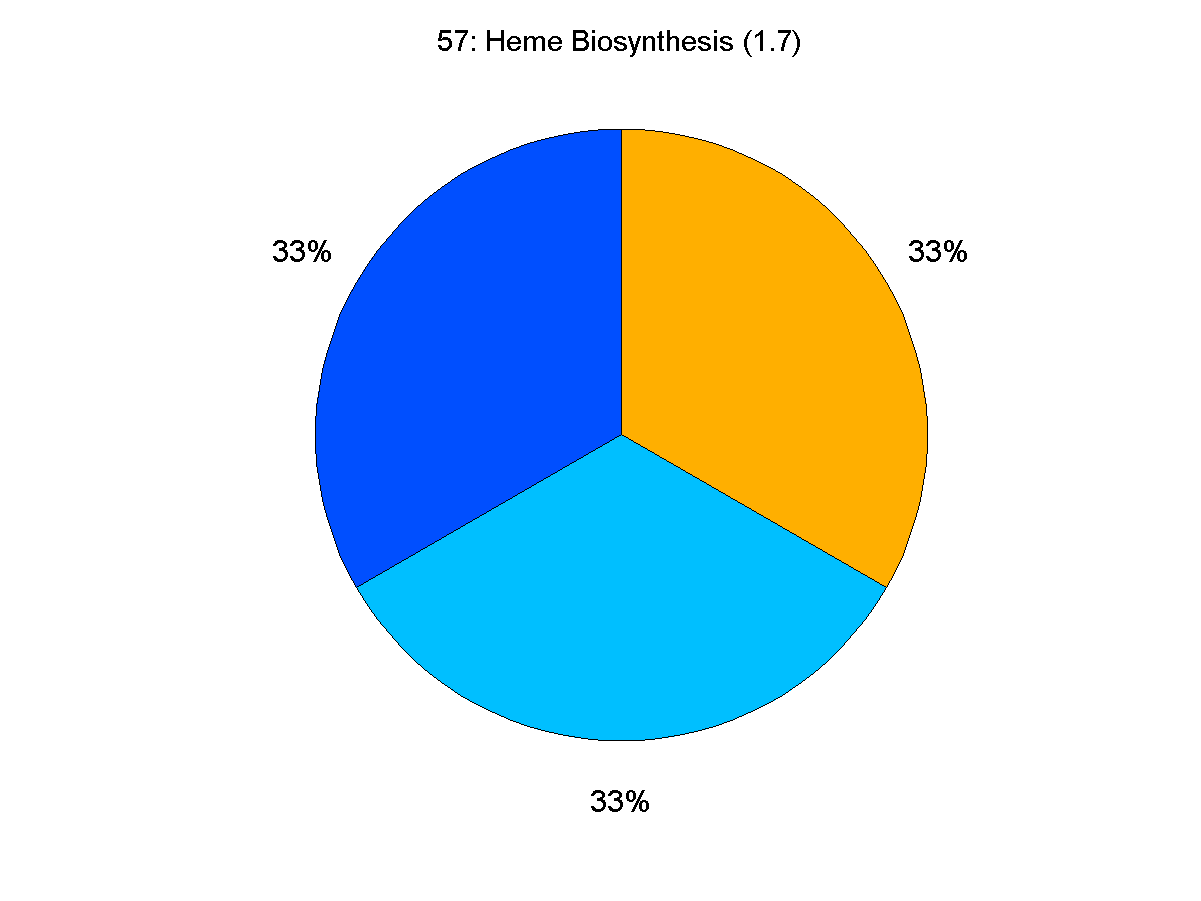

Supplement: S2 File — (ZIP) [file pone.0131875.s003.zip › MFC PieCharts/RegrEx1MFC/57HemeBiosynthesis.tif]

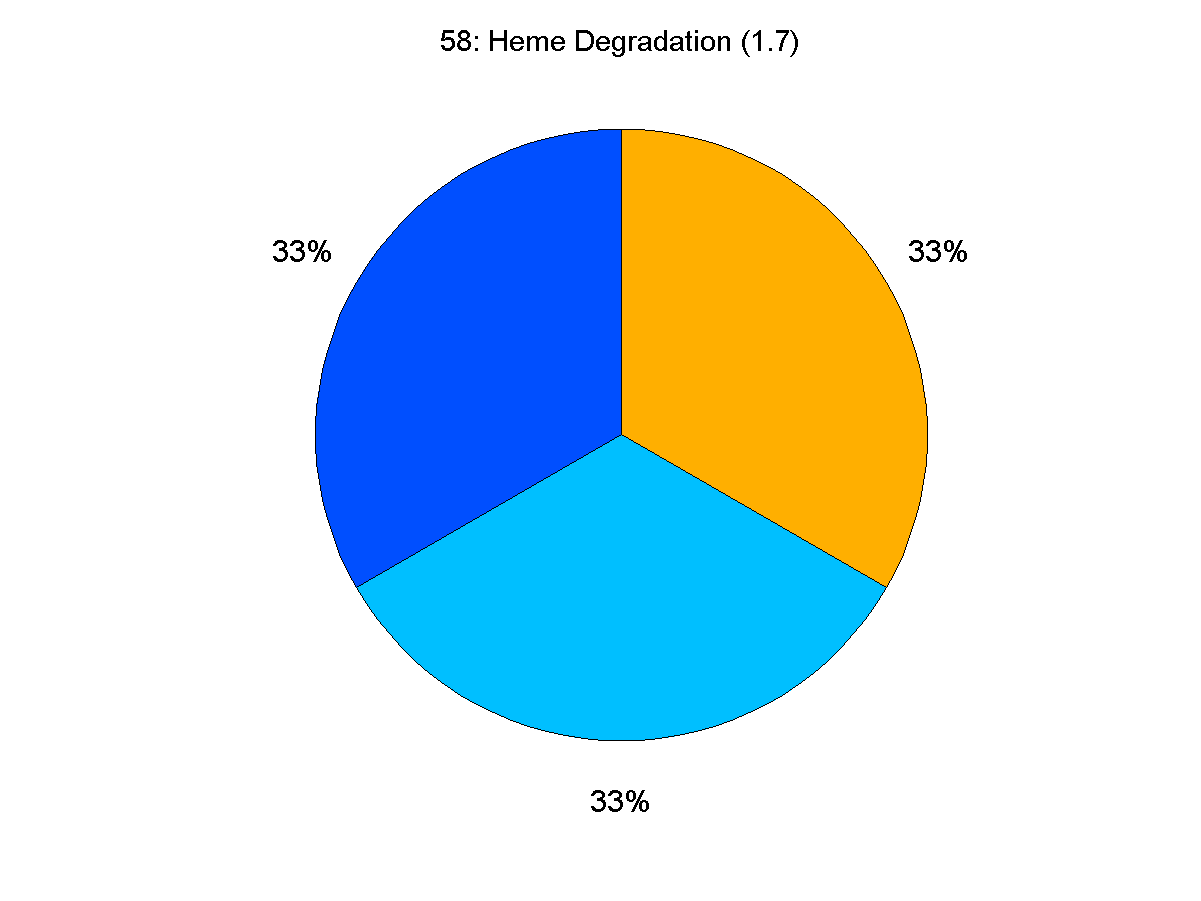

Supplement: S2 File — (ZIP) [file pone.0131875.s003.zip › MFC PieCharts/RegrEx1MFC/58HemeDegradation.tif]

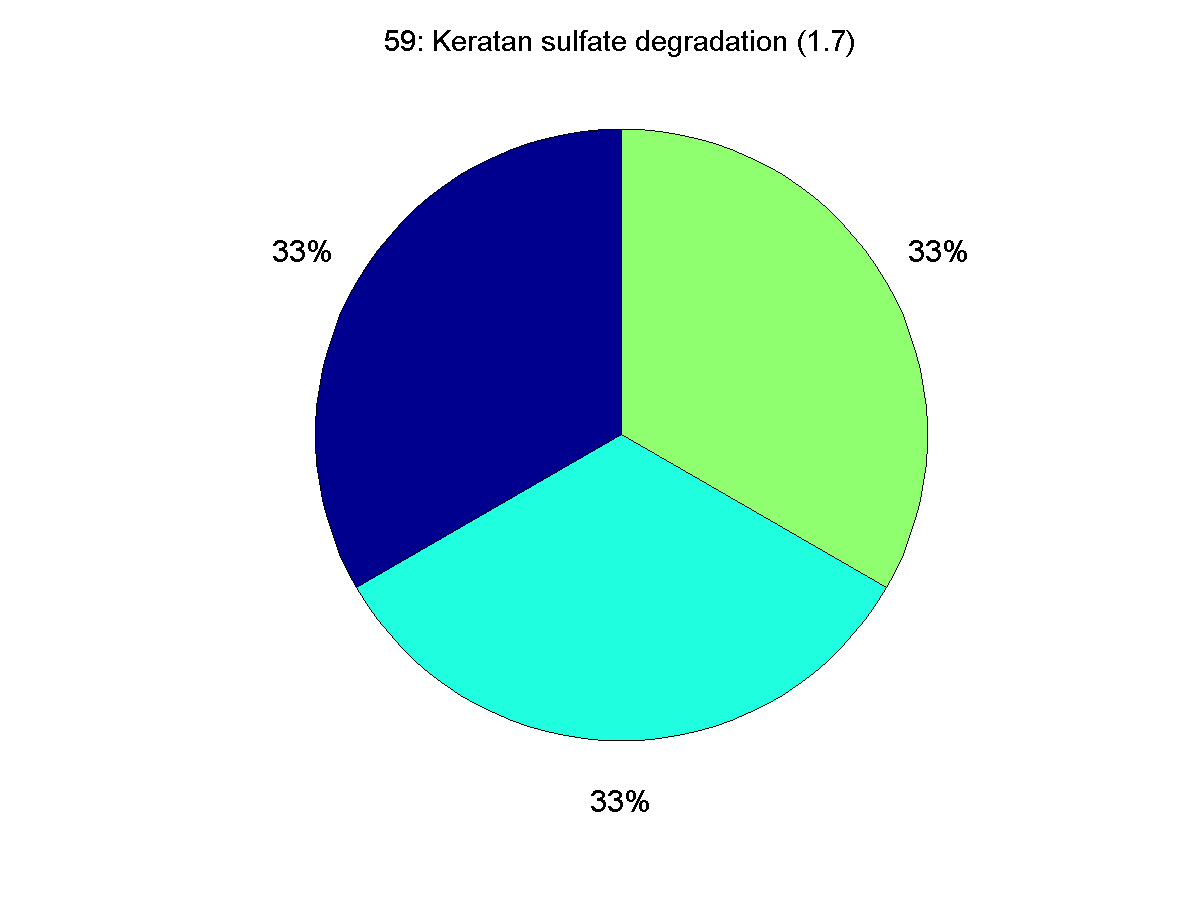

Supplement: S2 File — (ZIP) [file pone.0131875.s003.zip › MFC PieCharts/RegrEx1MFC/59Keratansulfatedegradation.tif]

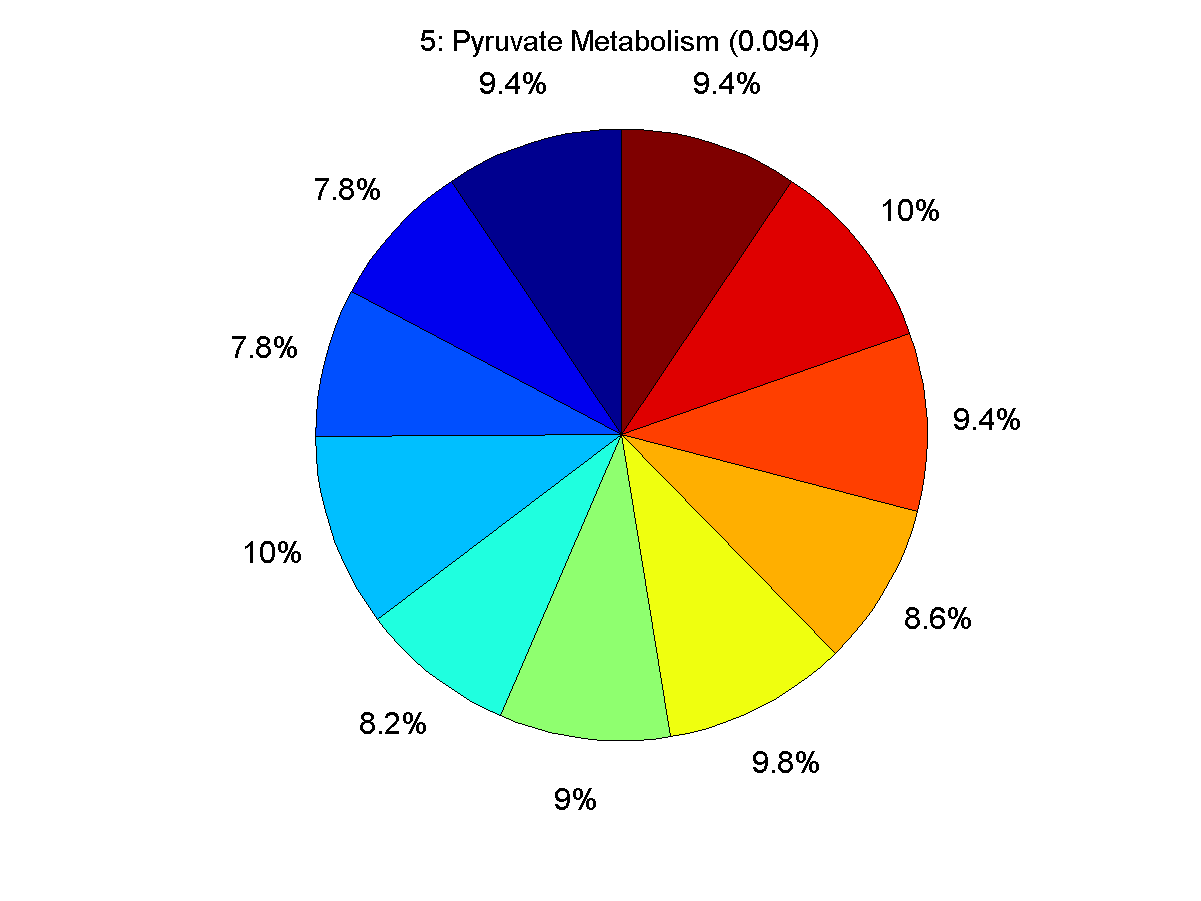

Supplement: S2 File — (ZIP) [file pone.0131875.s003.zip › MFC PieCharts/RegrEx1MFC/5PyruvateMetabolism.tif]

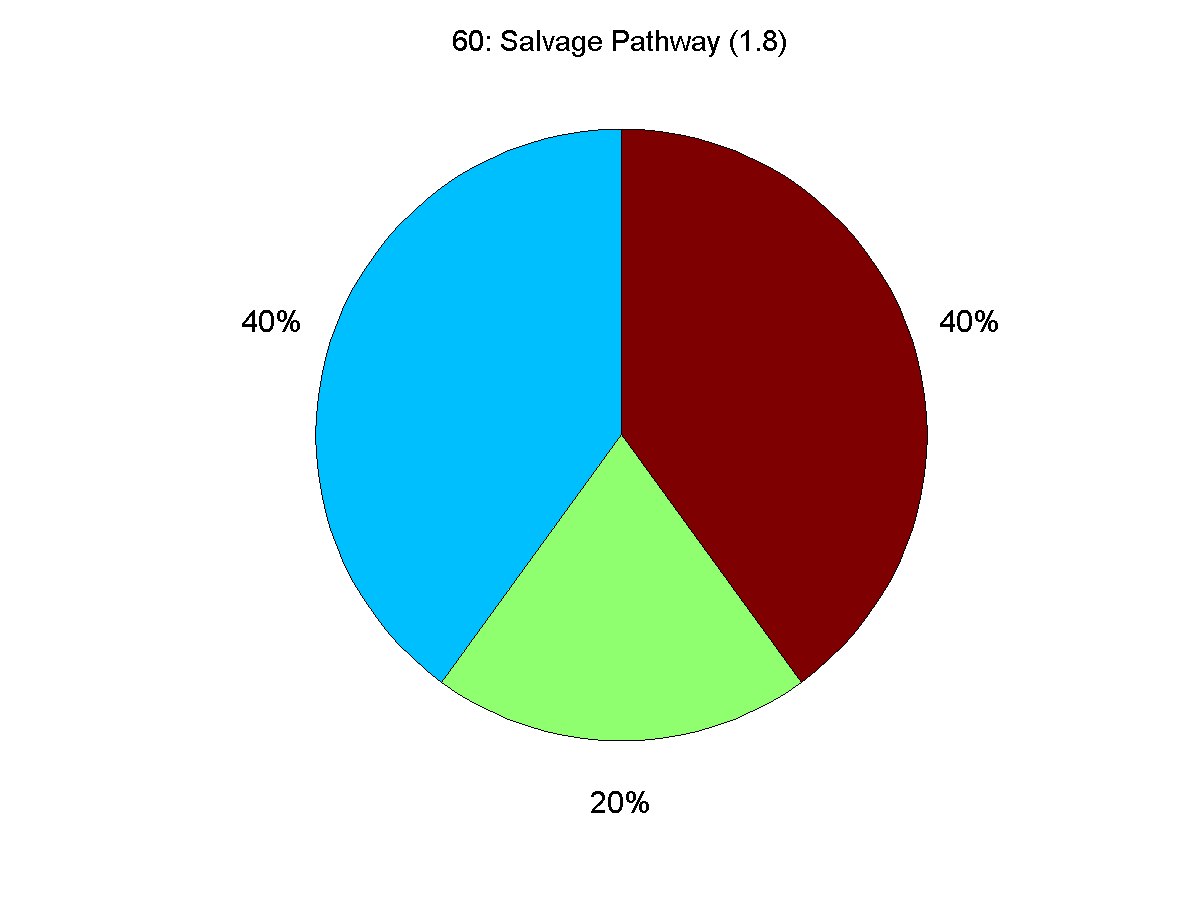

Supplement: S2 File — (ZIP) [file pone.0131875.s003.zip › MFC PieCharts/RegrEx1MFC/60SalvagePathway.tif]

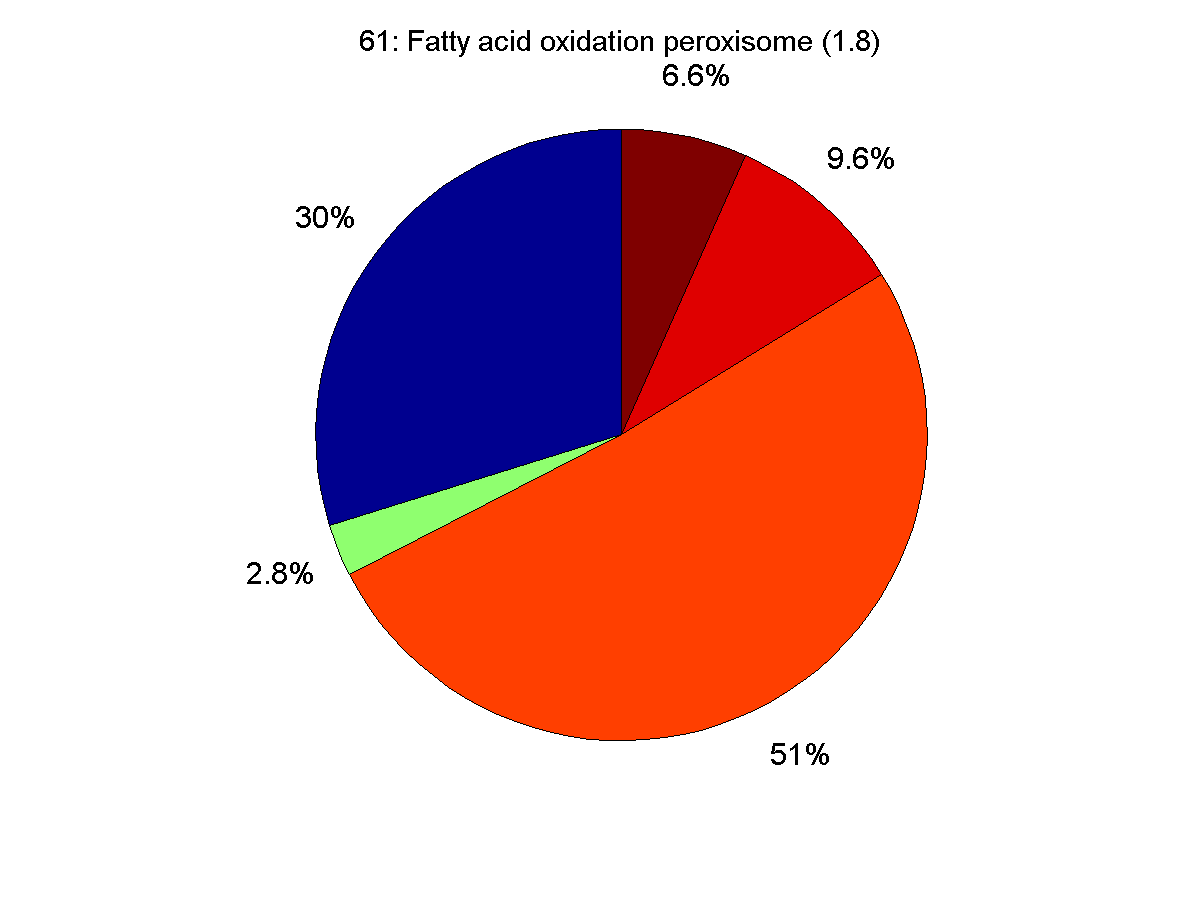

Supplement: S2 File — (ZIP) [file pone.0131875.s003.zip › MFC PieCharts/RegrEx1MFC/61Fattyacidoxidationperoxisome.tif]

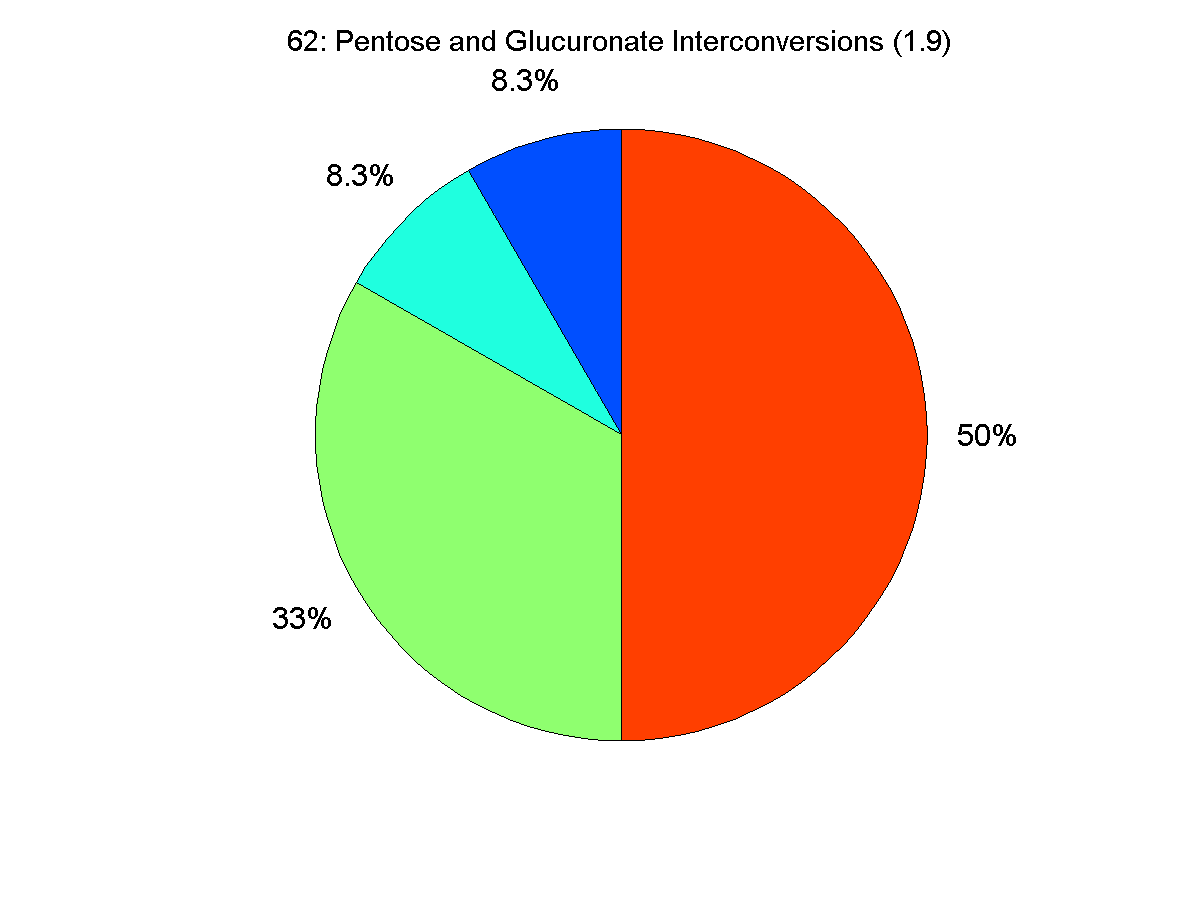

Supplement: S2 File — (ZIP) [file pone.0131875.s003.zip › MFC PieCharts/RegrEx1MFC/62PentoseandGlucuronateInterconversions.tif]

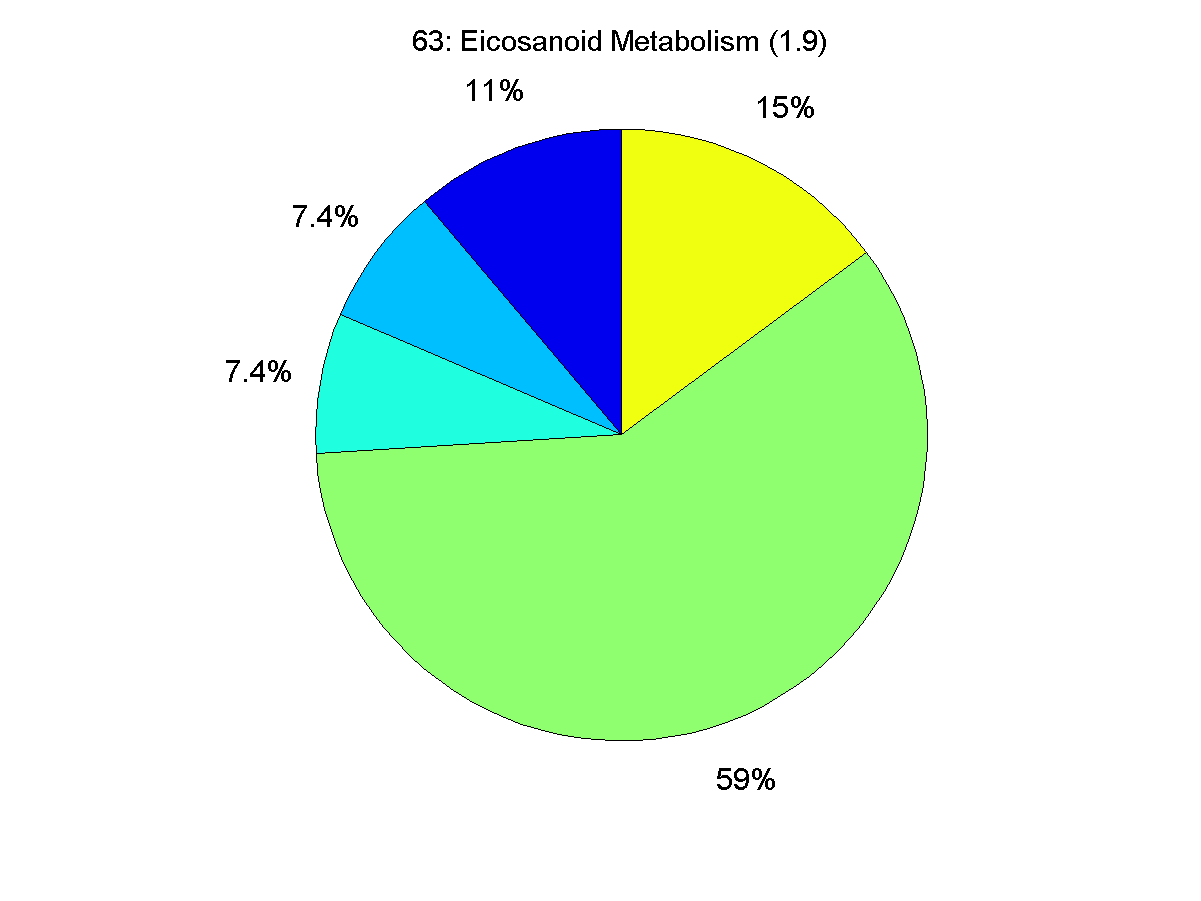

Supplement: S2 File — (ZIP) [file pone.0131875.s003.zip › MFC PieCharts/RegrEx1MFC/63EicosanoidMetabolism.tif]

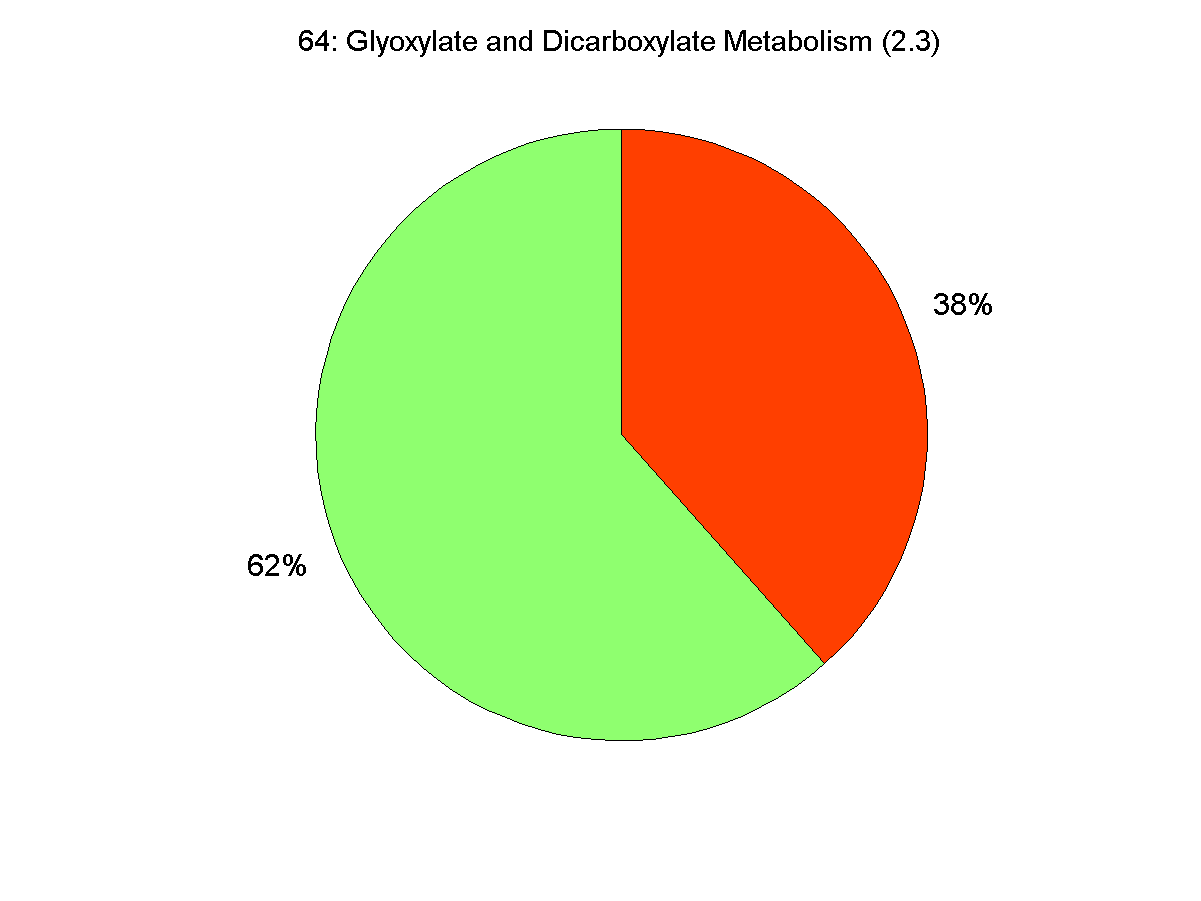

Supplement: S2 File — (ZIP) [file pone.0131875.s003.zip › MFC PieCharts/RegrEx1MFC/64GlyoxylateandDicarboxylateMetabolism.tif]

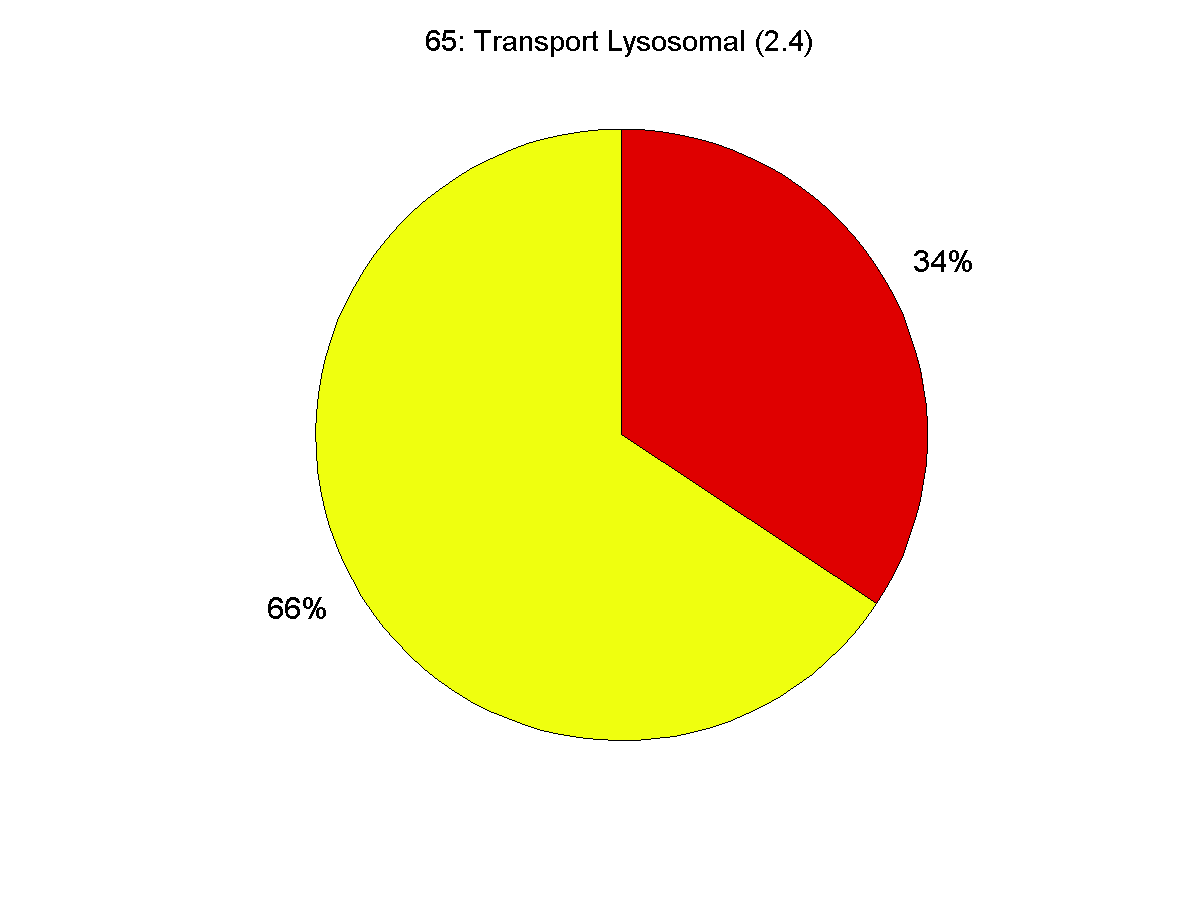

Supplement: S2 File — (ZIP) [file pone.0131875.s003.zip › MFC PieCharts/RegrEx1MFC/65TransportLysosomal.tif]

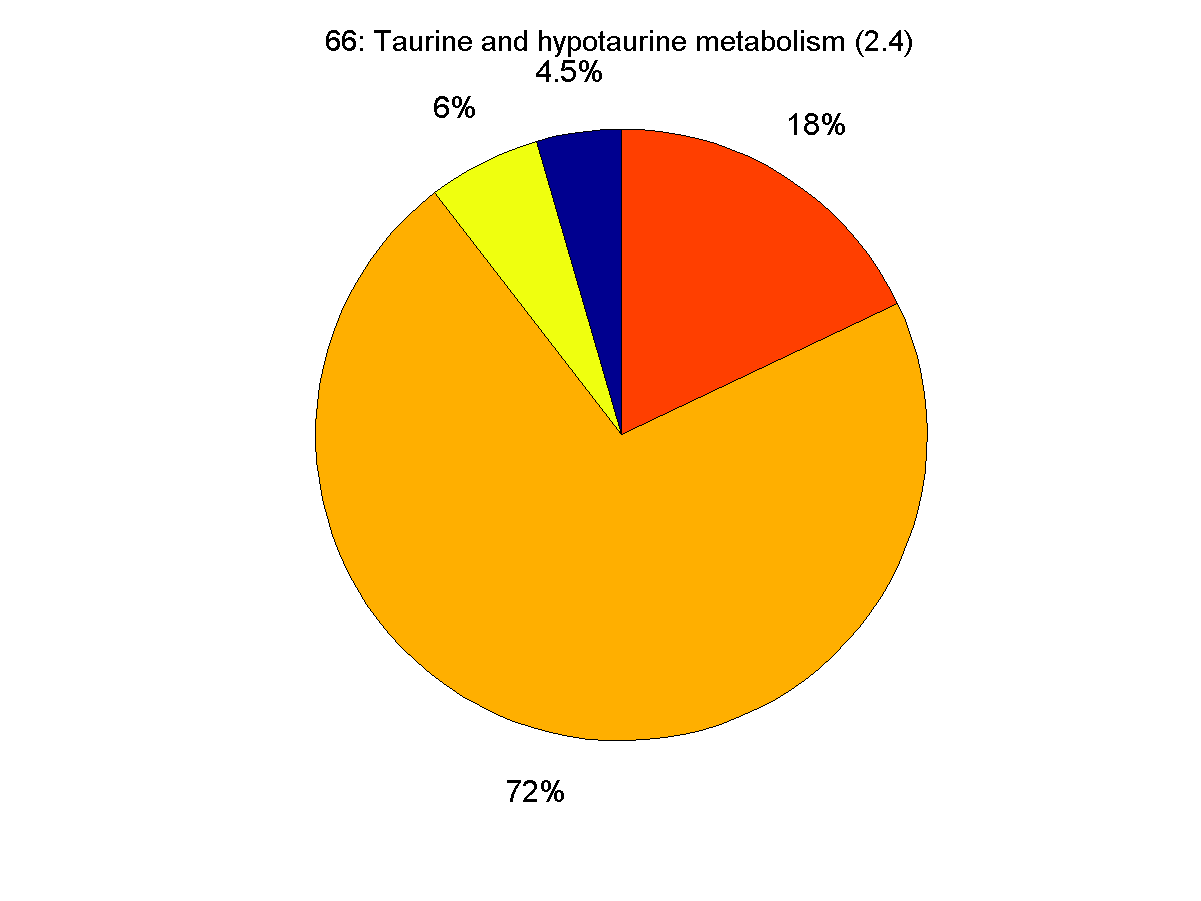

Supplement: S2 File — (ZIP) [file pone.0131875.s003.zip › MFC PieCharts/RegrEx1MFC/66Taurineandhypotaurinemetabolism.tif]

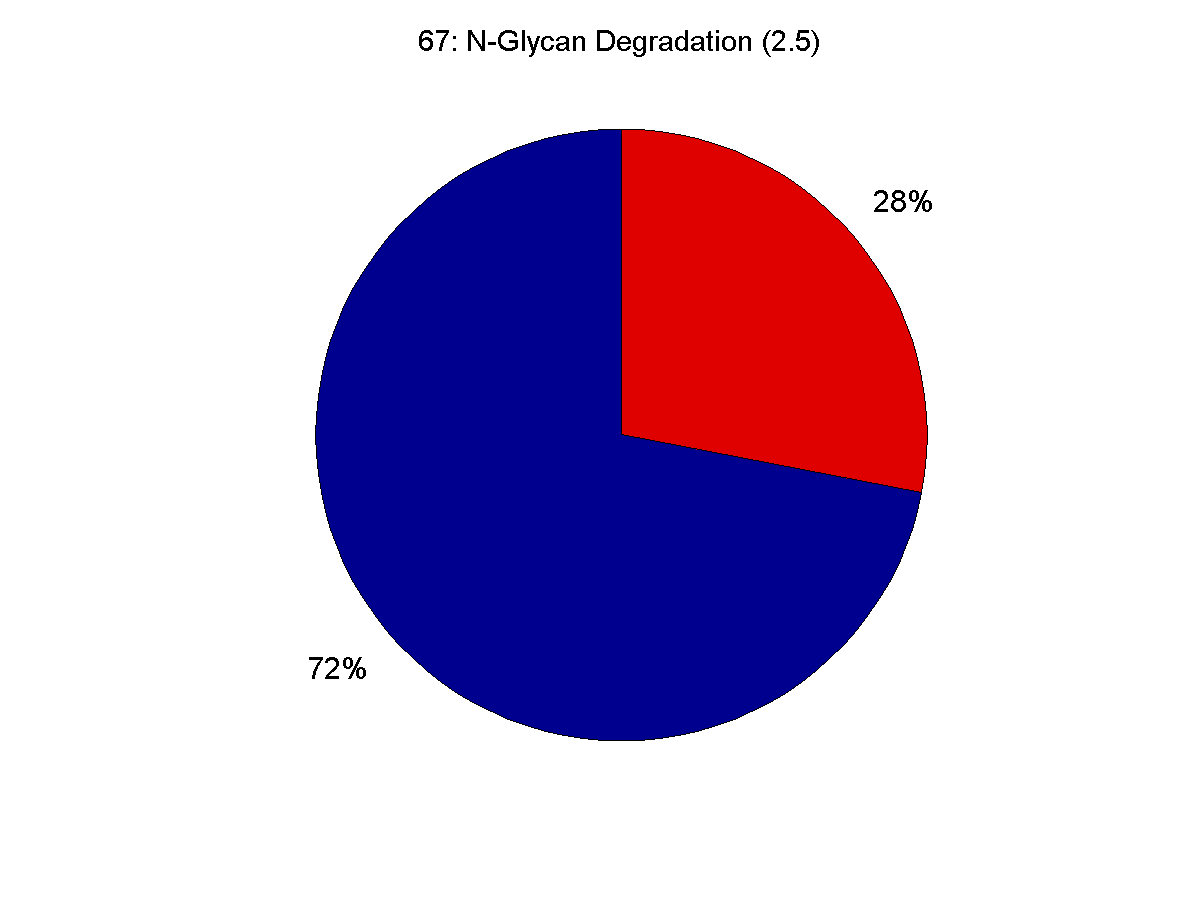

Supplement: S2 File — (ZIP) [file pone.0131875.s003.zip › MFC PieCharts/RegrEx1MFC/67N-GlycanDegradation.tif]

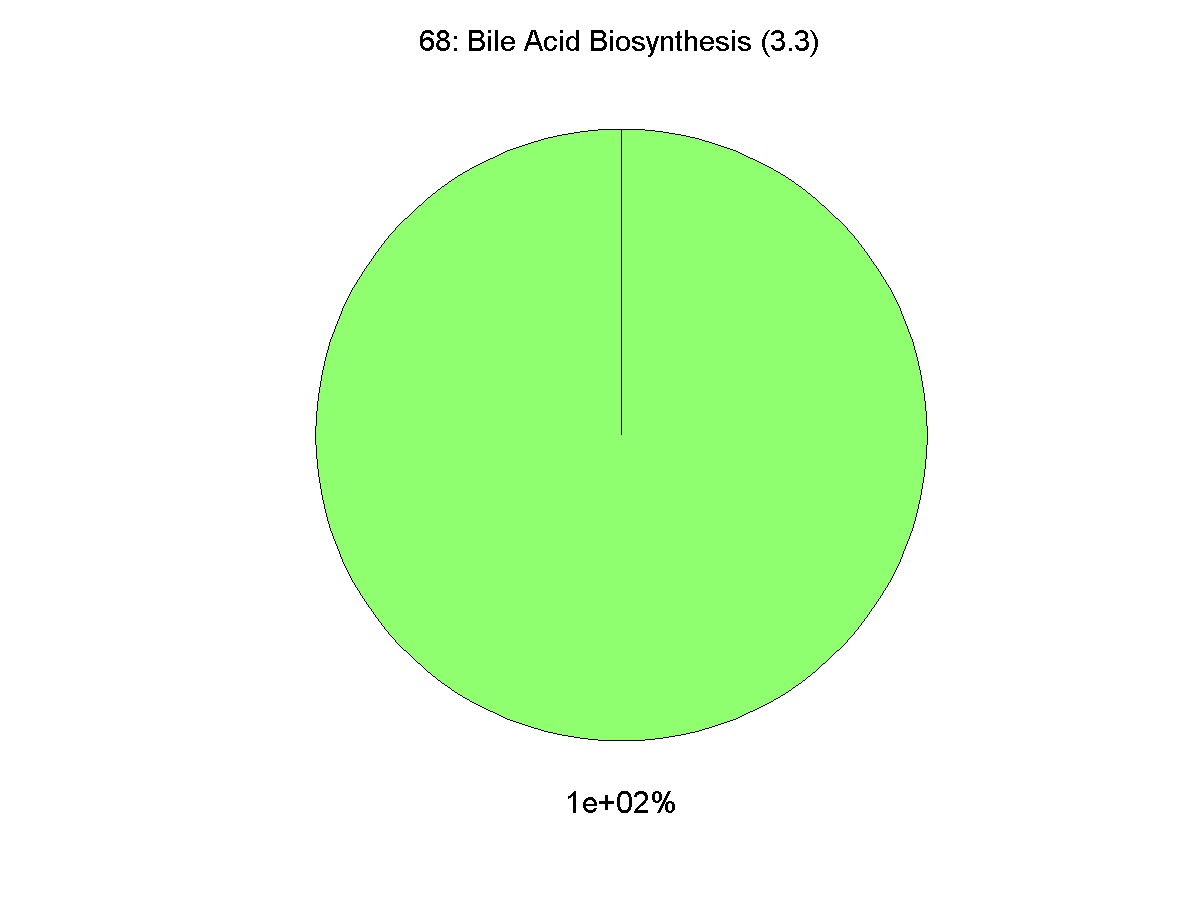

Supplement: S2 File — (ZIP) [file pone.0131875.s003.zip › MFC PieCharts/RegrEx1MFC/68BileAcidBiosynthesis.tif]

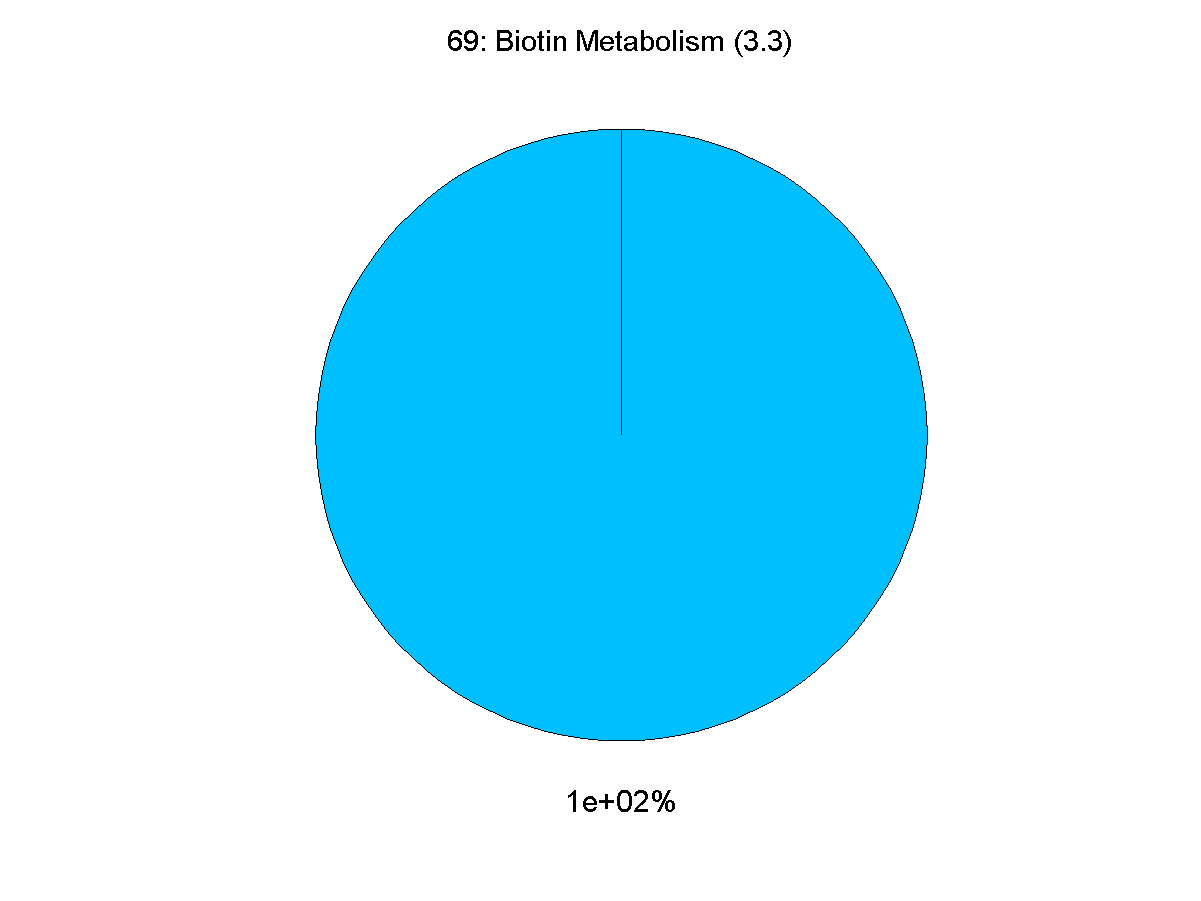

Supplement: S2 File — (ZIP) [file pone.0131875.s003.zip › MFC PieCharts/RegrEx1MFC/69BiotinMetabolism.tif]

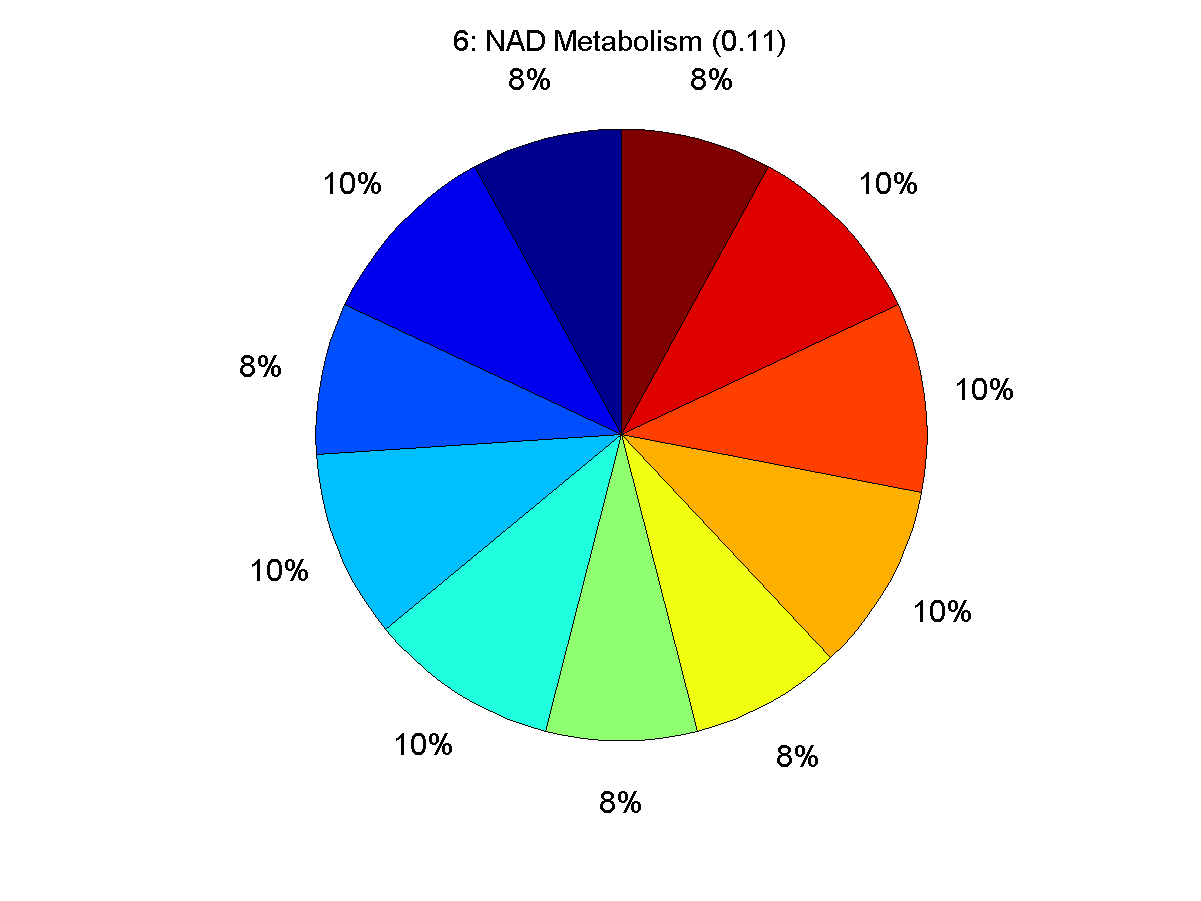

Supplement: S2 File — (ZIP) [file pone.0131875.s003.zip › MFC PieCharts/RegrEx1MFC/6NADMetabolism.tif]

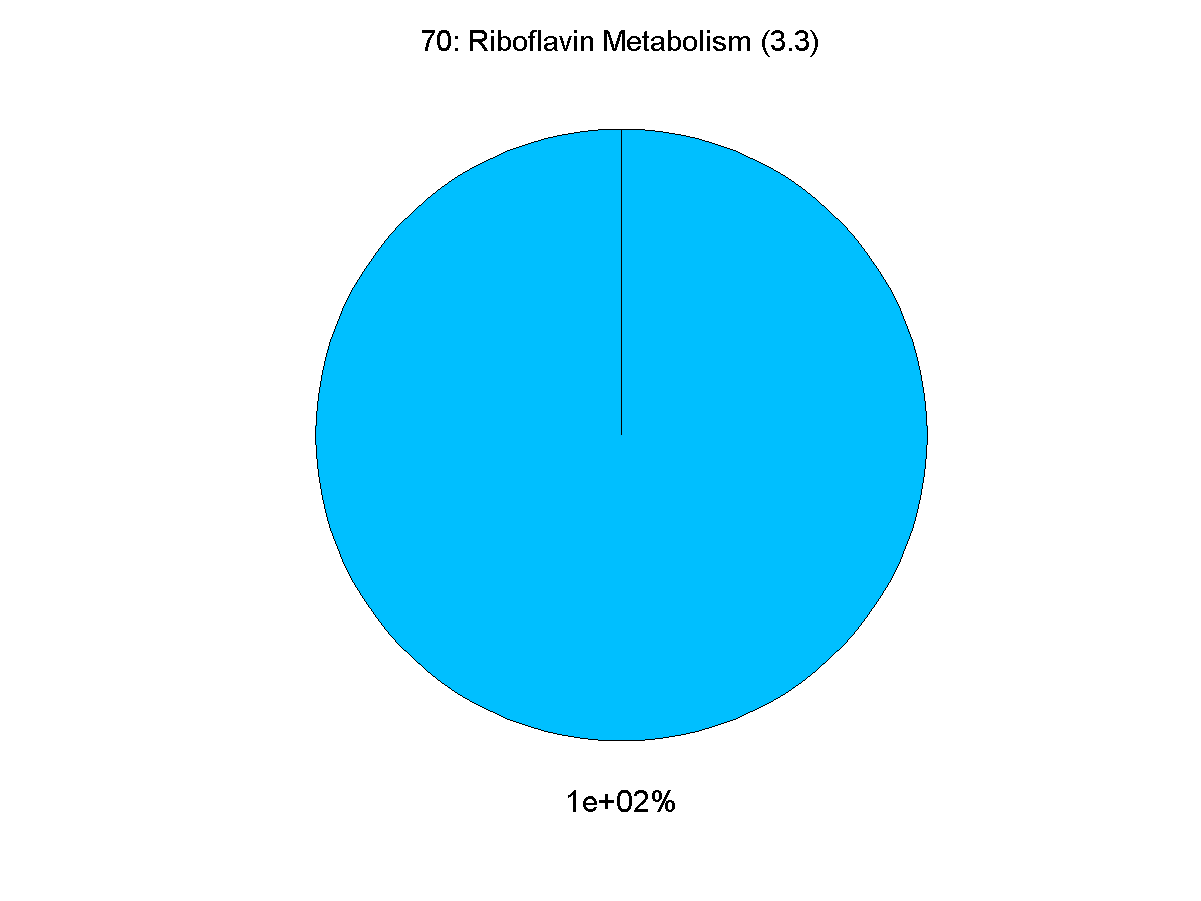

Supplement: S2 File — (ZIP) [file pone.0131875.s003.zip › MFC PieCharts/RegrEx1MFC/70RiboflavinMetabolism.tif]

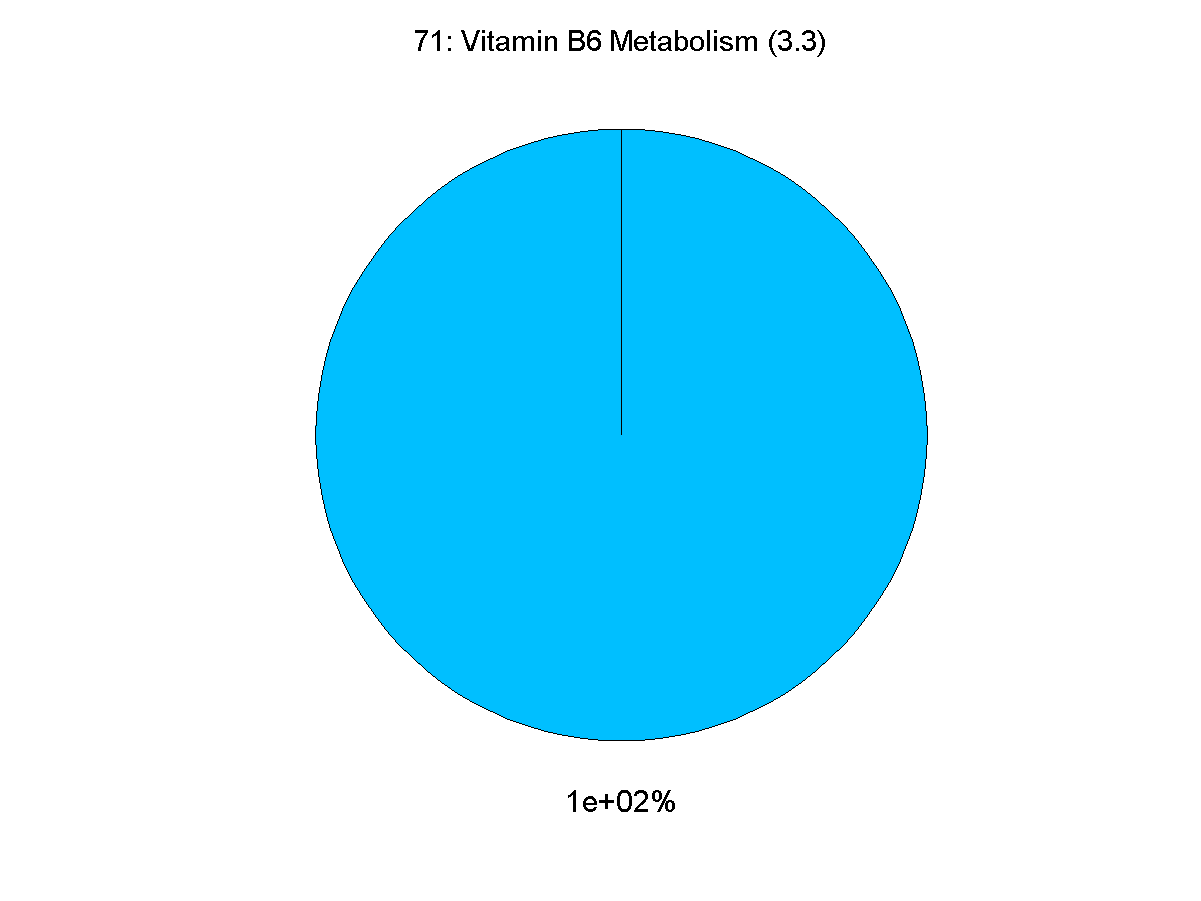

Supplement: S2 File — (ZIP) [file pone.0131875.s003.zip › MFC PieCharts/RegrEx1MFC/71VitaminB6Metabolism.tif]

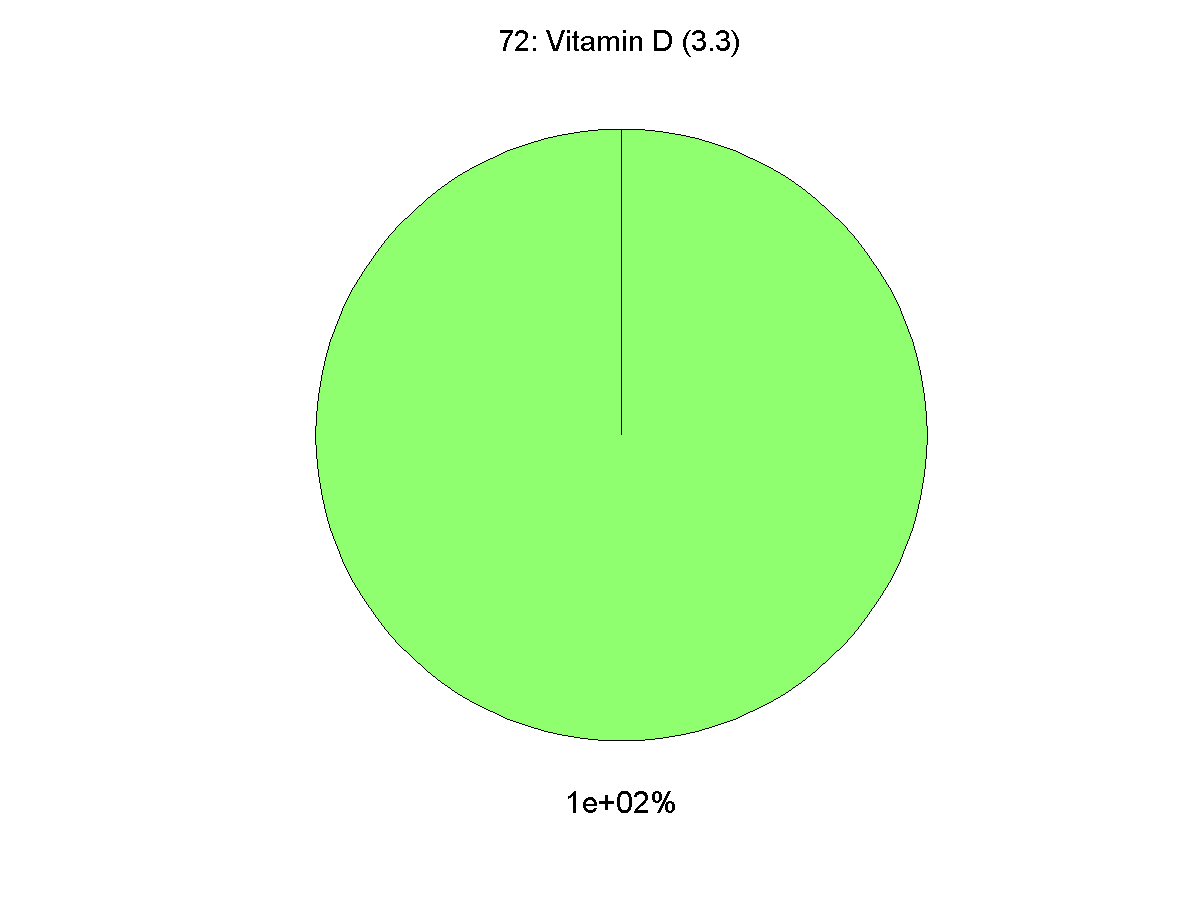

Supplement: S2 File — (ZIP) [file pone.0131875.s003.zip › MFC PieCharts/RegrEx1MFC/72VitaminD.tif]

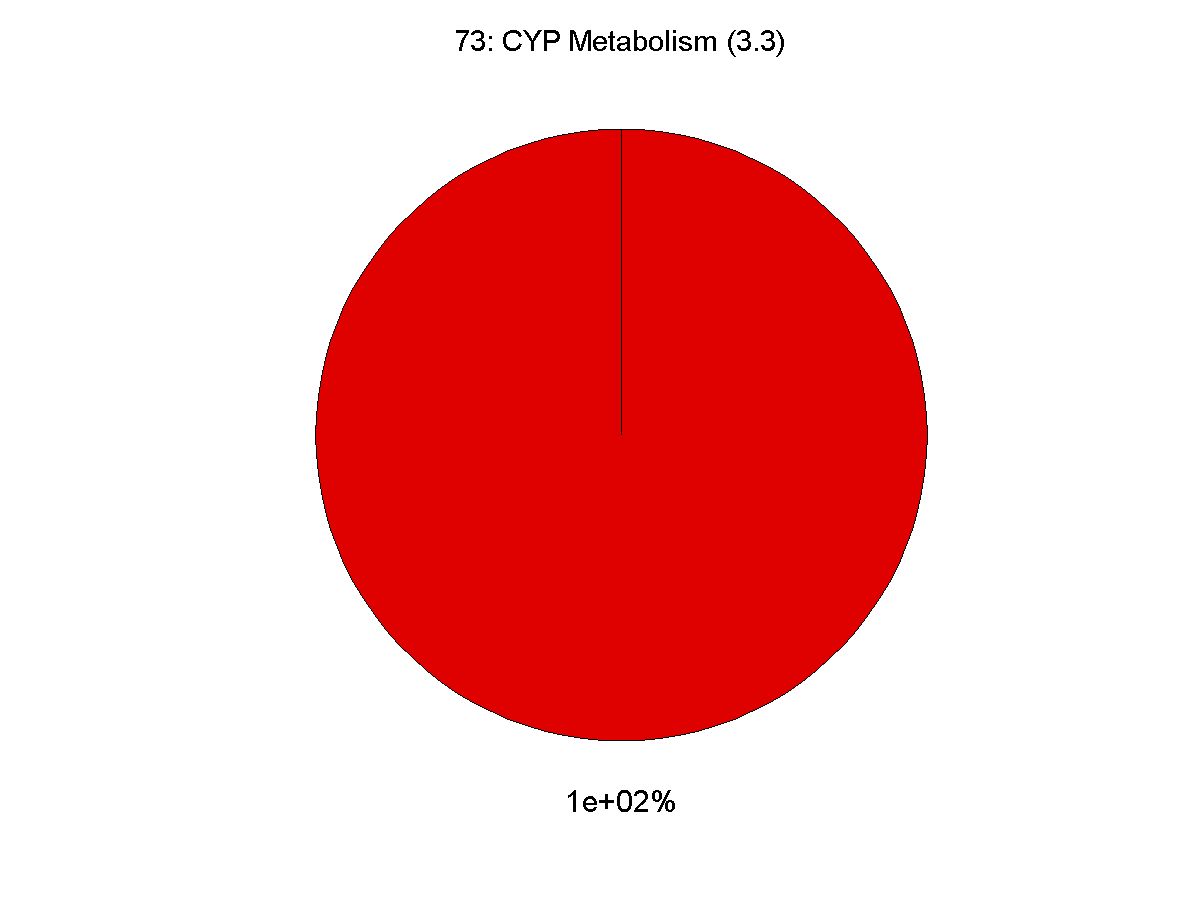

Supplement: S2 File — (ZIP) [file pone.0131875.s003.zip › MFC PieCharts/RegrEx1MFC/73CYPMetabolism.tif]

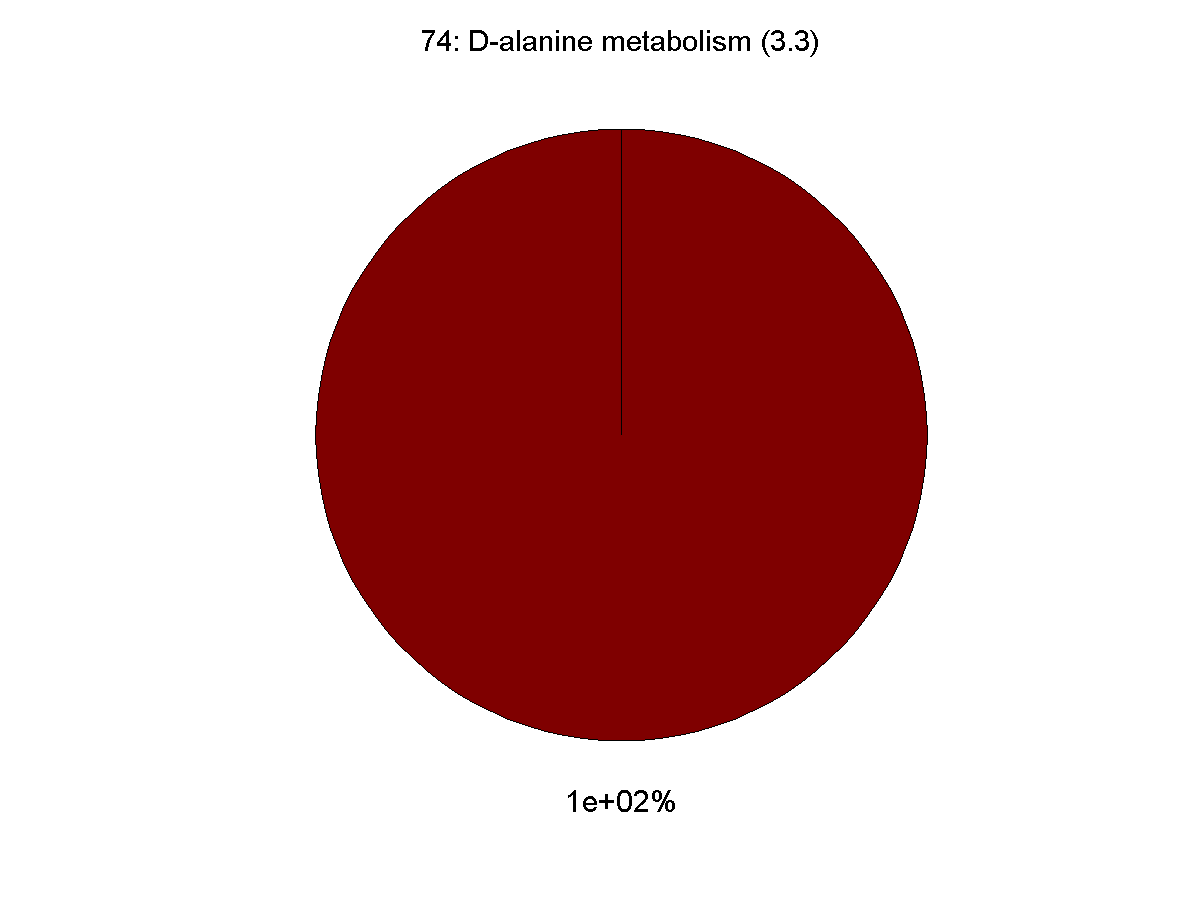

Supplement: S2 File — (ZIP) [file pone.0131875.s003.zip › MFC PieCharts/RegrEx1MFC/74D-alaninemetabolism.tif]

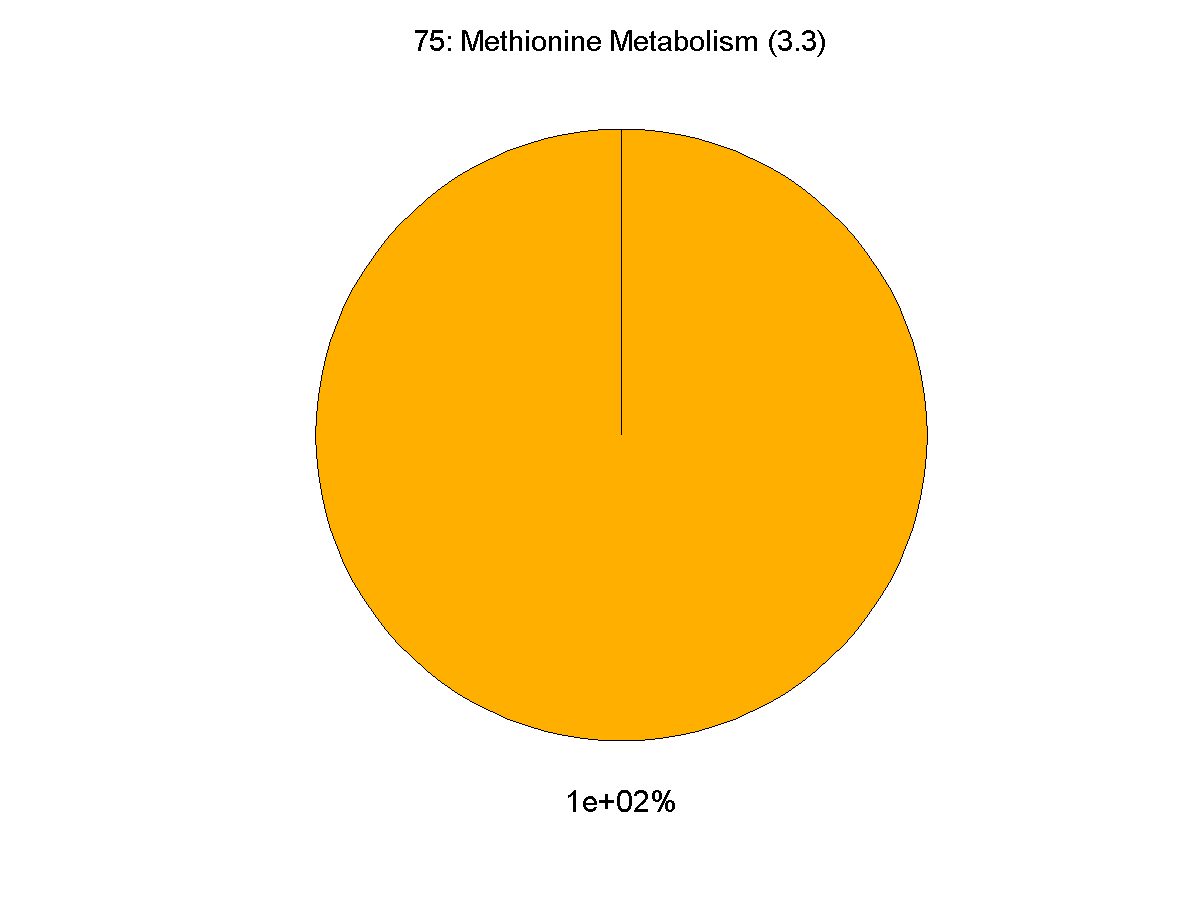

Supplement: S2 File — (ZIP) [file pone.0131875.s003.zip › MFC PieCharts/RegrEx1MFC/75MethionineMetabolism.tif]

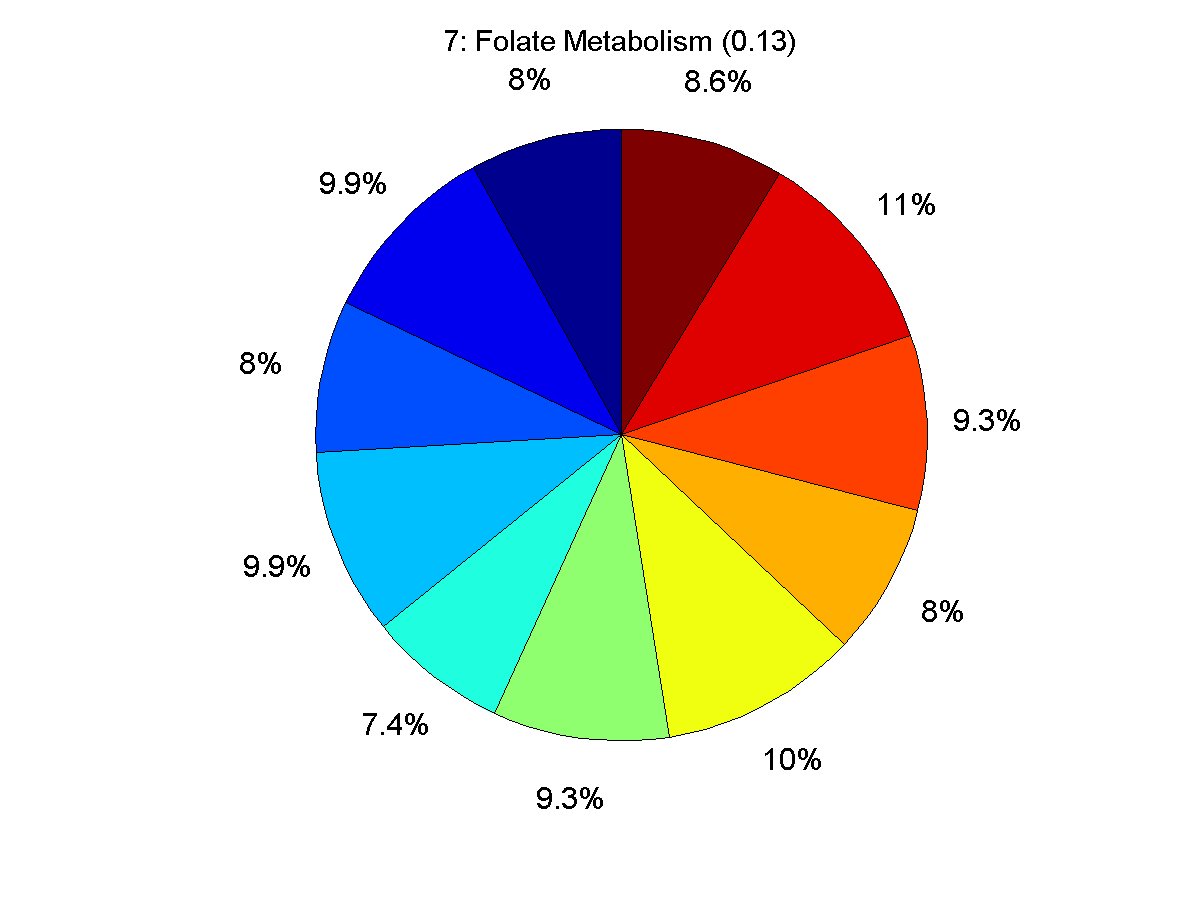

Supplement: S2 File — (ZIP) [file pone.0131875.s003.zip › MFC PieCharts/RegrEx1MFC/7FolateMetabolism.tif]

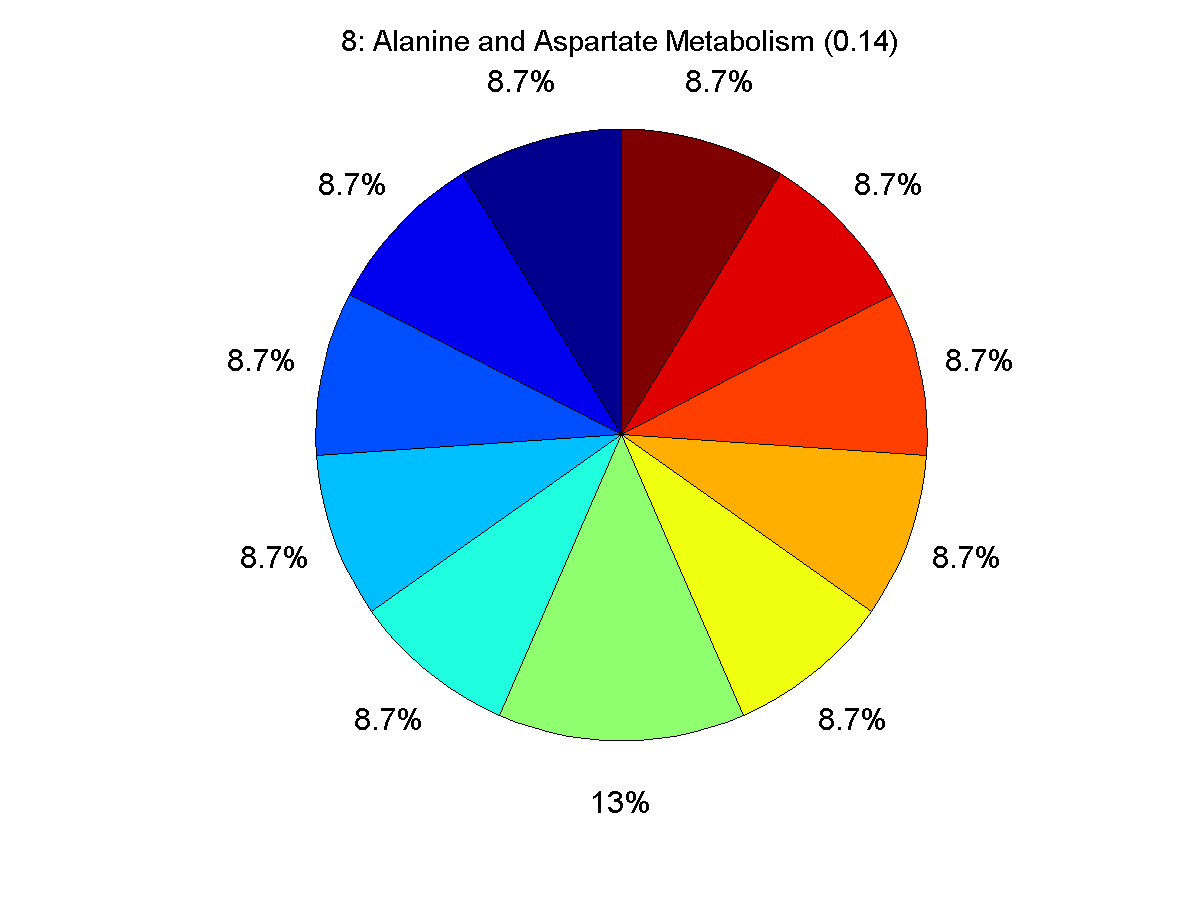

Supplement: S2 File — (ZIP) [file pone.0131875.s003.zip › MFC PieCharts/RegrEx1MFC/8AlanineandAspartateMetabolism.tif]

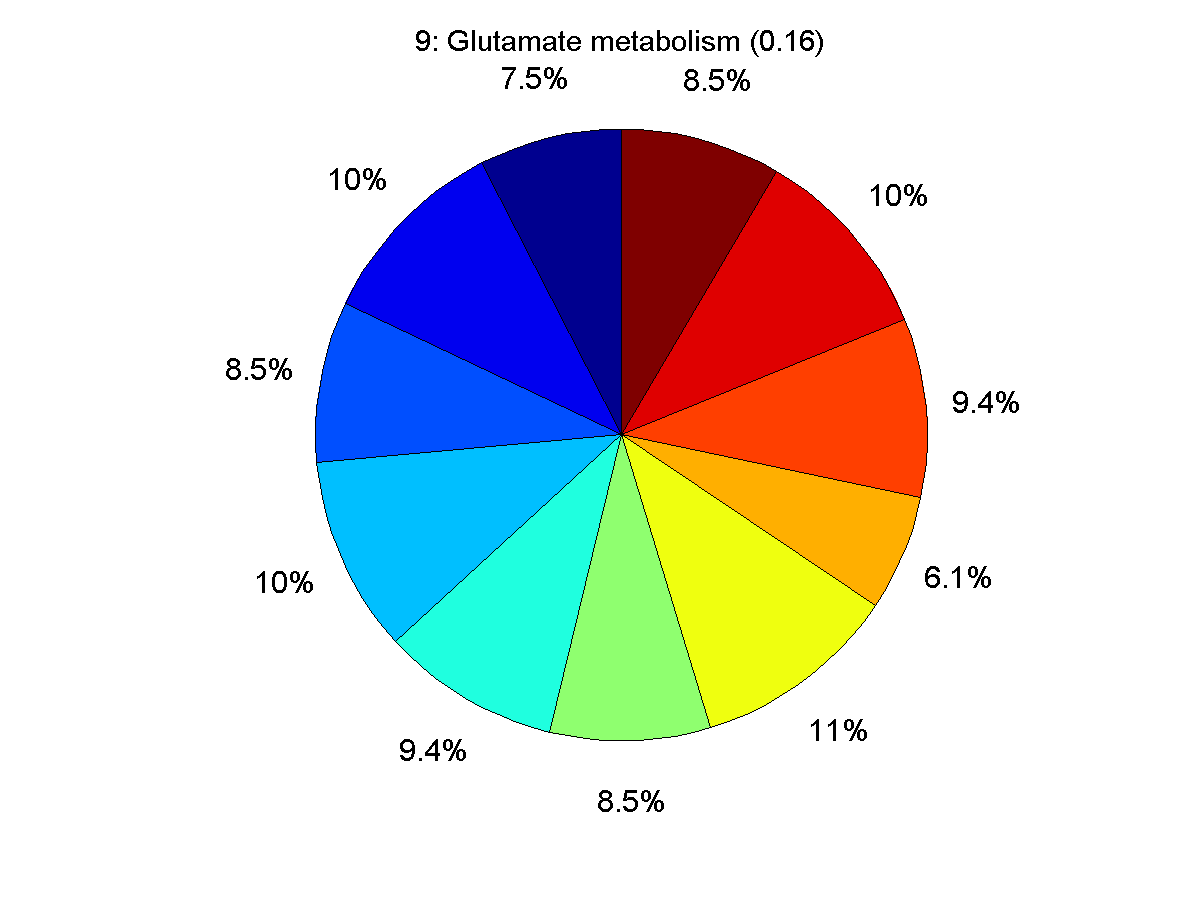

Supplement: S2 File — (ZIP) [file pone.0131875.s003.zip › MFC PieCharts/RegrEx1MFC/9Glutamatemetabolism.tif]

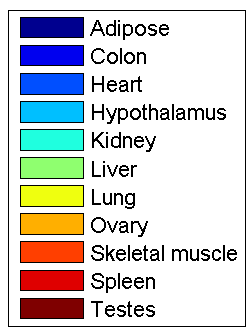

Supplement: S2 File — (ZIP) [file pone.0131875.s003.zip › MFC PieCharts/RegrEx1MFC/Legend.tif]

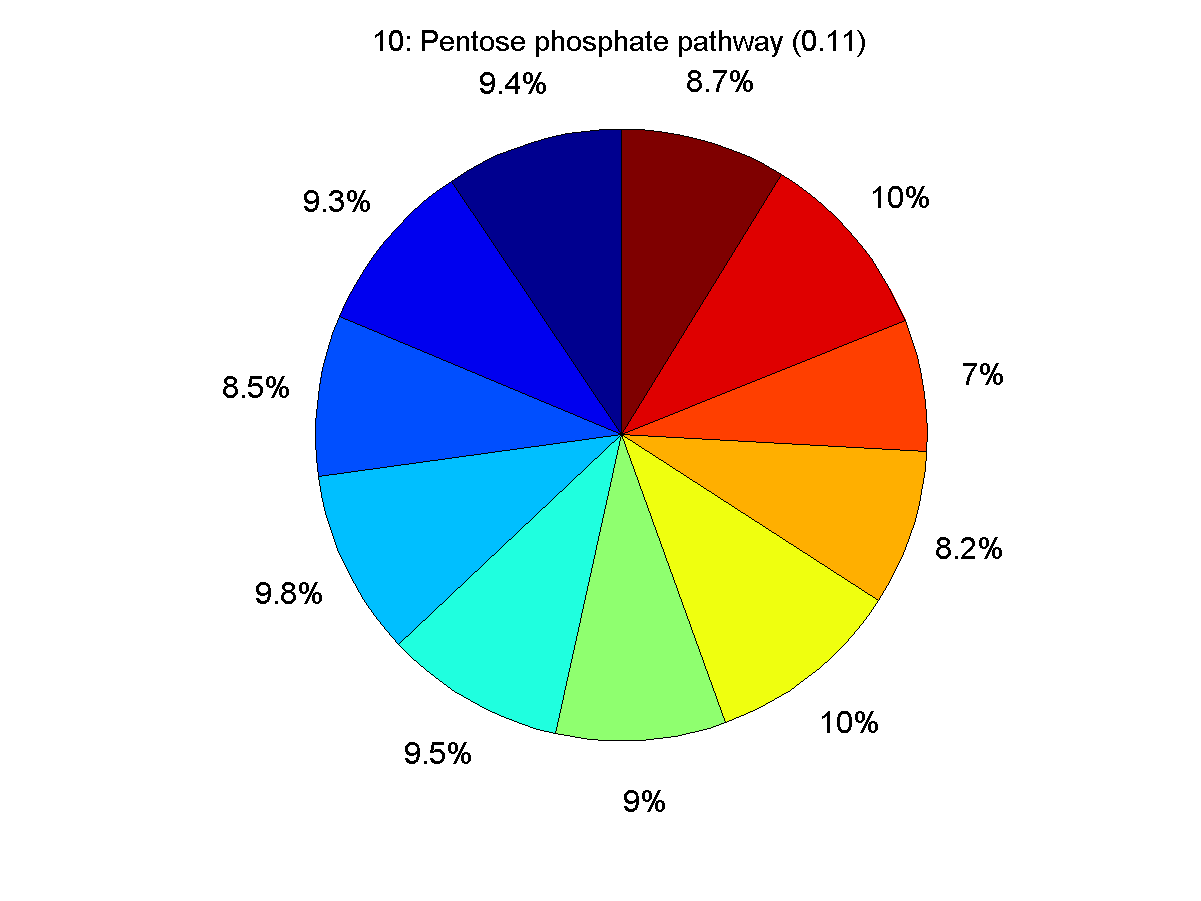

Supplement: S2 File — (ZIP) [file pone.0131875.s003.zip › MFC PieCharts/RegrEx2MFC/10Pentosephosphatepathway.tif]

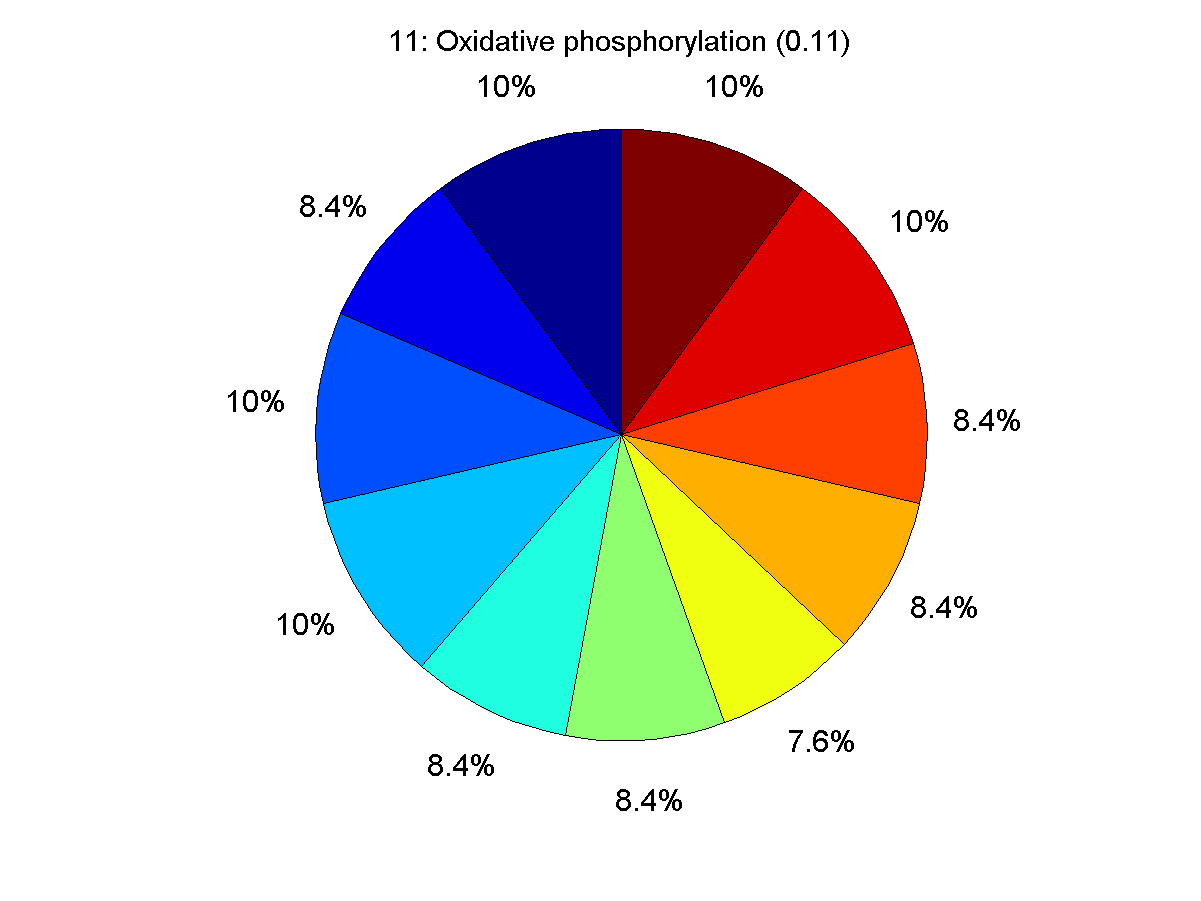

Supplement: S2 File — (ZIP) [file pone.0131875.s003.zip › MFC PieCharts/RegrEx2MFC/11Oxidativephosphorylation.tif]

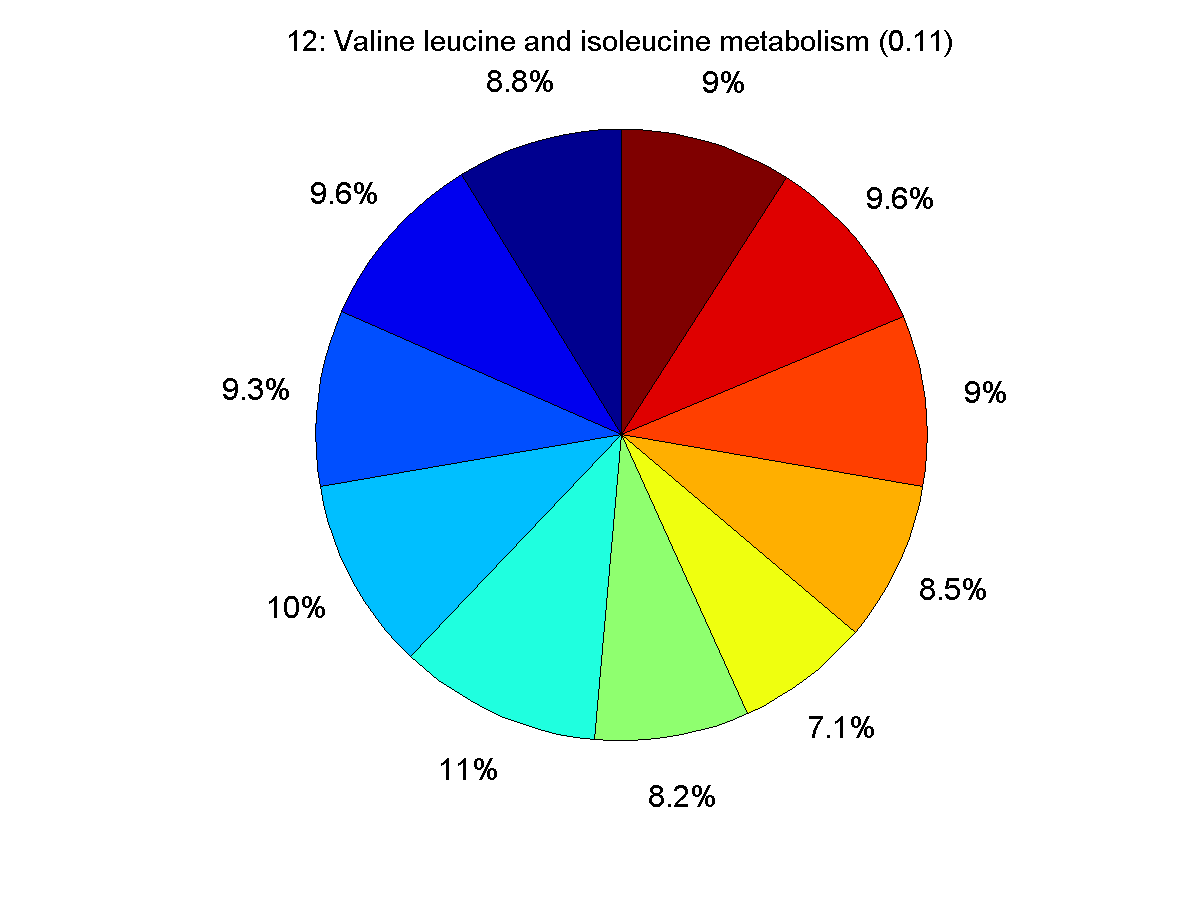

Supplement: S2 File — (ZIP) [file pone.0131875.s003.zip › MFC PieCharts/RegrEx2MFC/12Valineleucineandisoleucinemetabolism.tif]

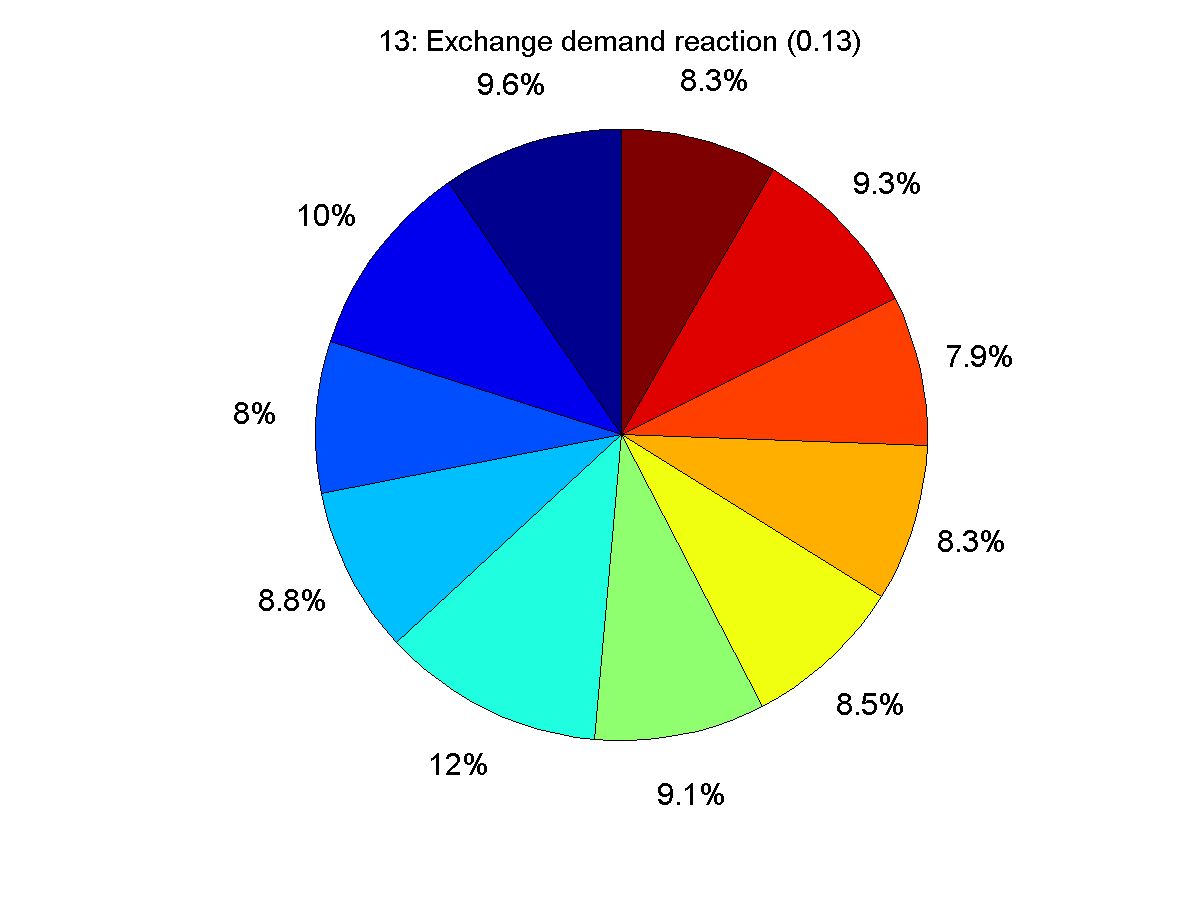

Supplement: S2 File — (ZIP) [file pone.0131875.s003.zip › MFC PieCharts/RegrEx2MFC/13Exchangedemandreaction.tif]

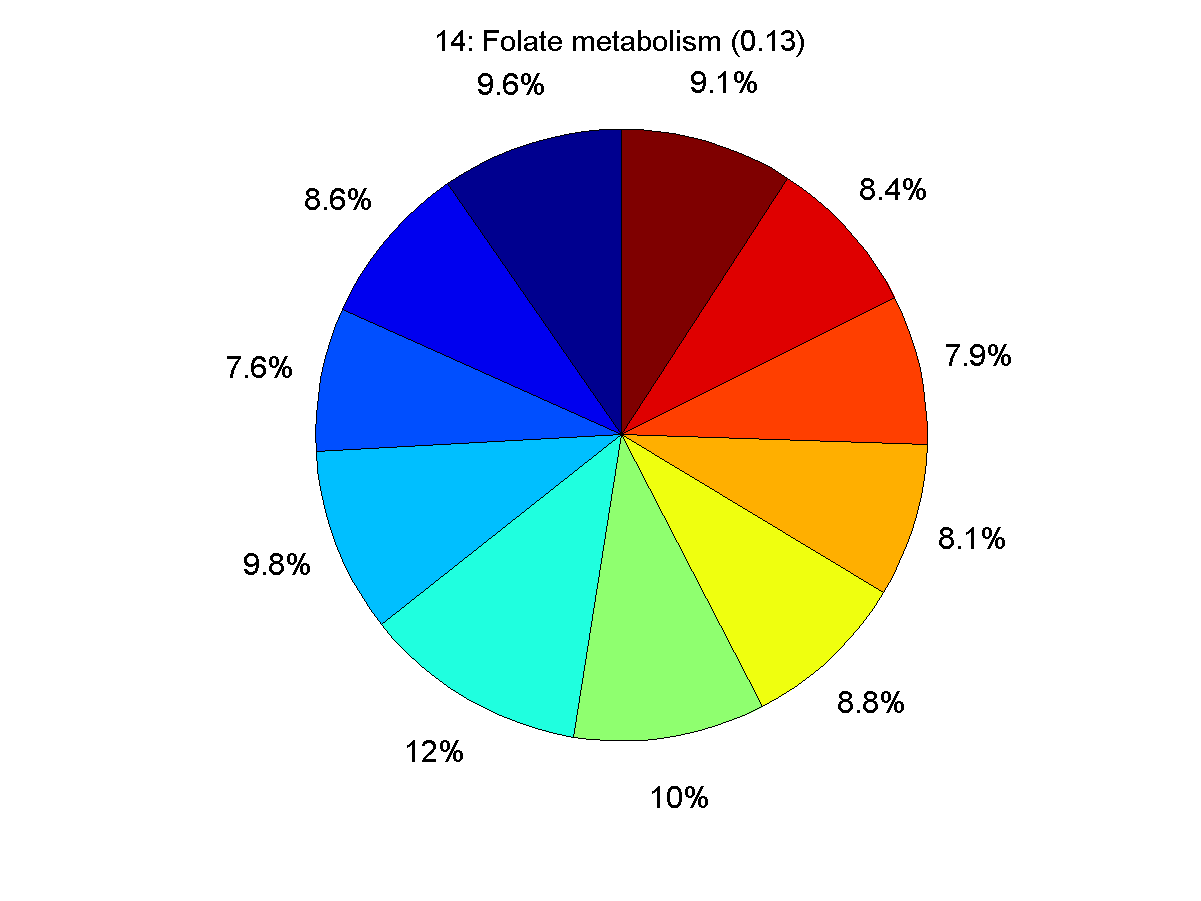

Supplement: S2 File — (ZIP) [file pone.0131875.s003.zip › MFC PieCharts/RegrEx2MFC/14Folatemetabolism.tif]

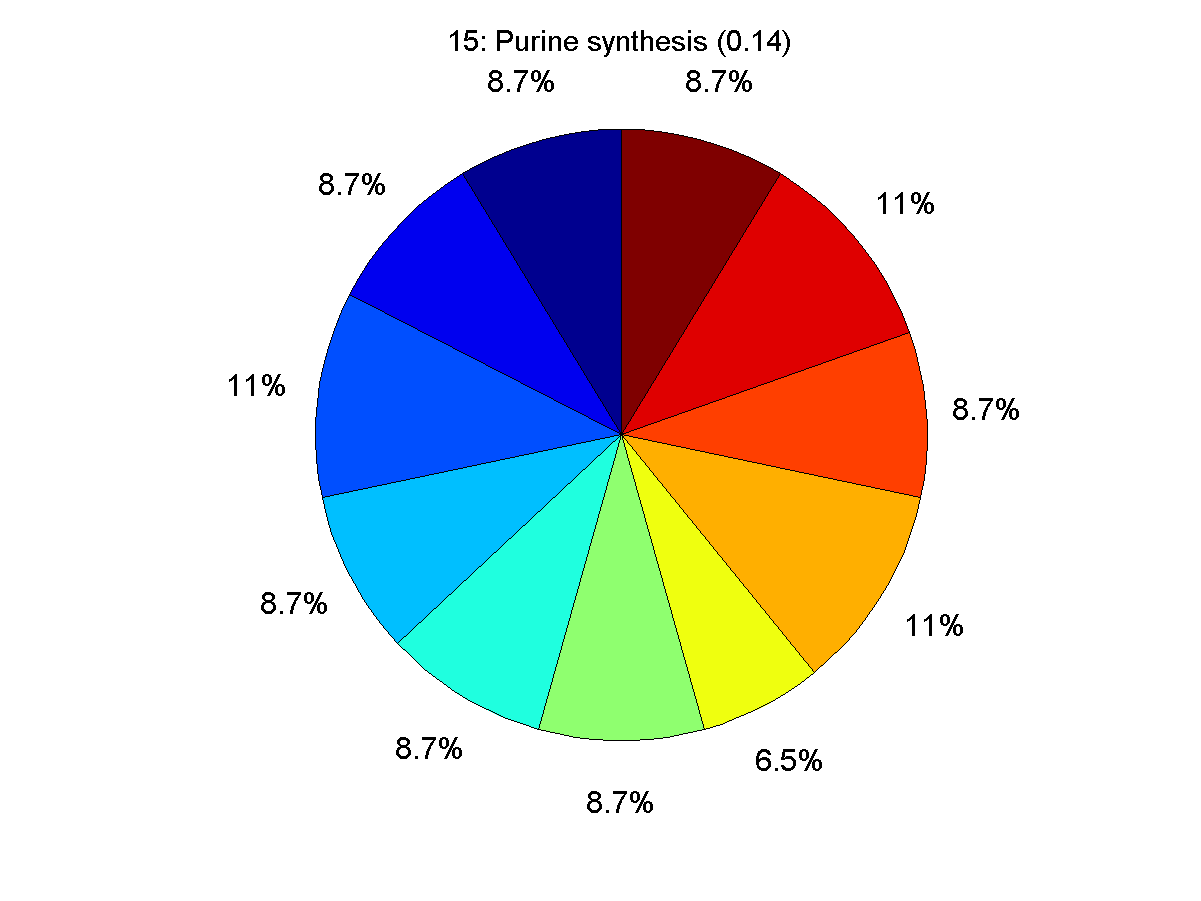

Supplement: S2 File — (ZIP) [file pone.0131875.s003.zip › MFC PieCharts/RegrEx2MFC/15Purinesynthesis.tif]

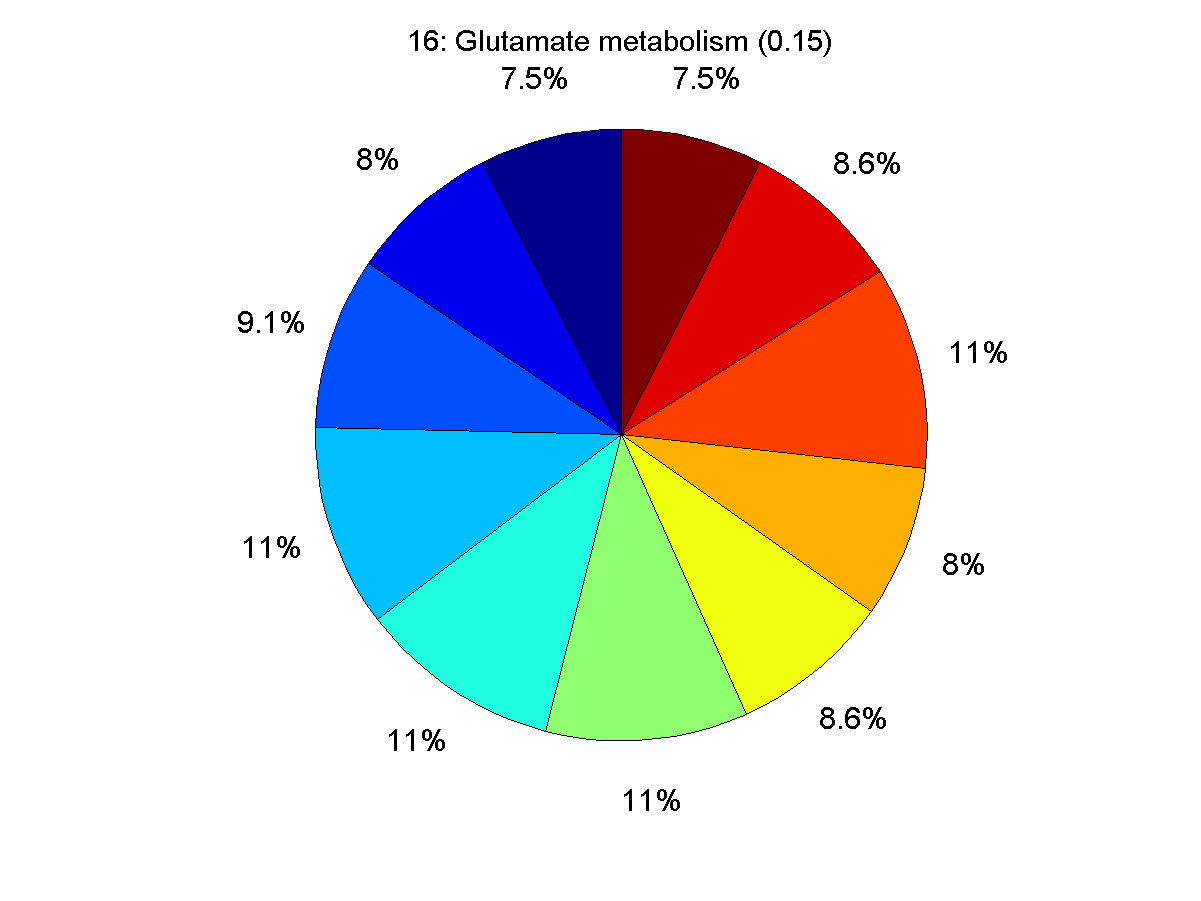

Supplement: S2 File — (ZIP) [file pone.0131875.s003.zip › MFC PieCharts/RegrEx2MFC/16Glutamatemetabolism.tif]

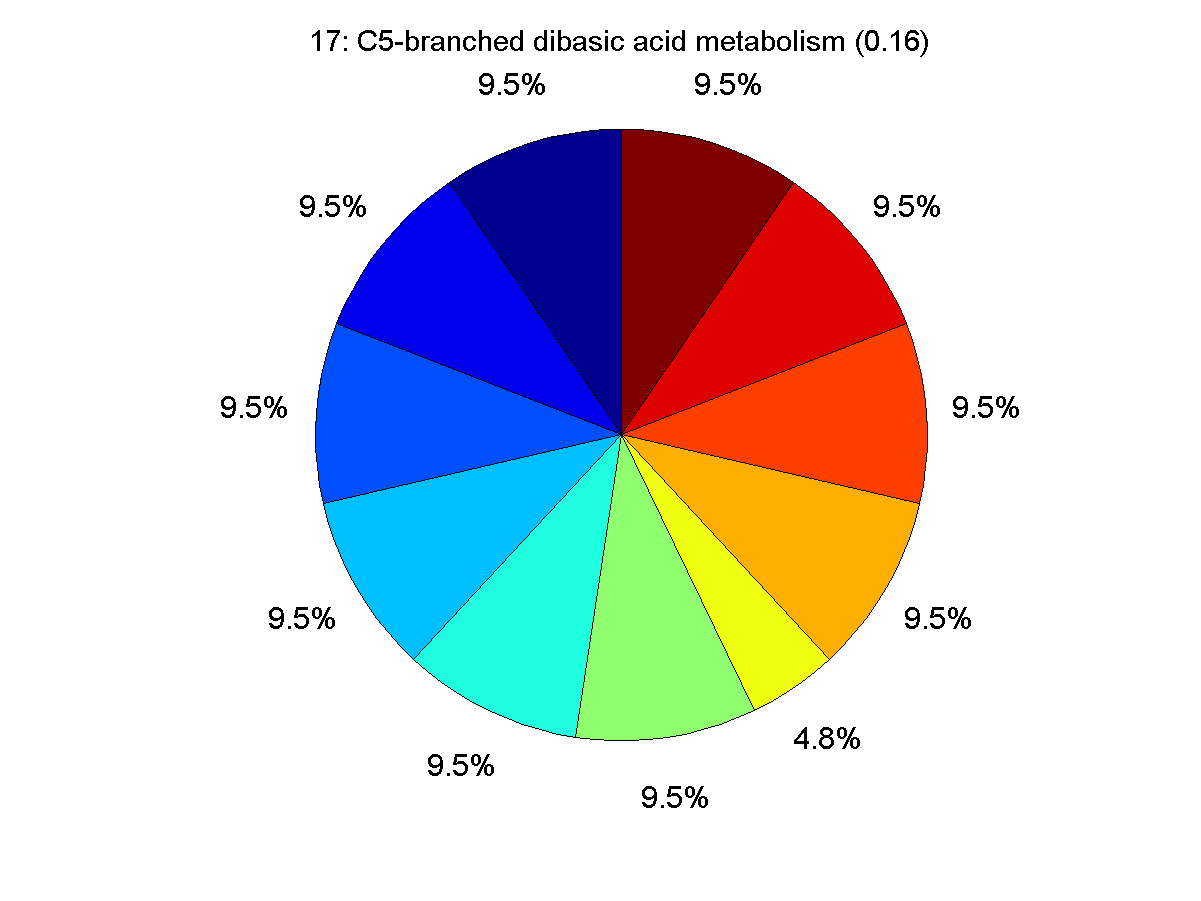

Supplement: S2 File — (ZIP) [file pone.0131875.s003.zip › MFC PieCharts/RegrEx2MFC/17C5-brancheddibasicacidmetabolism.tif]

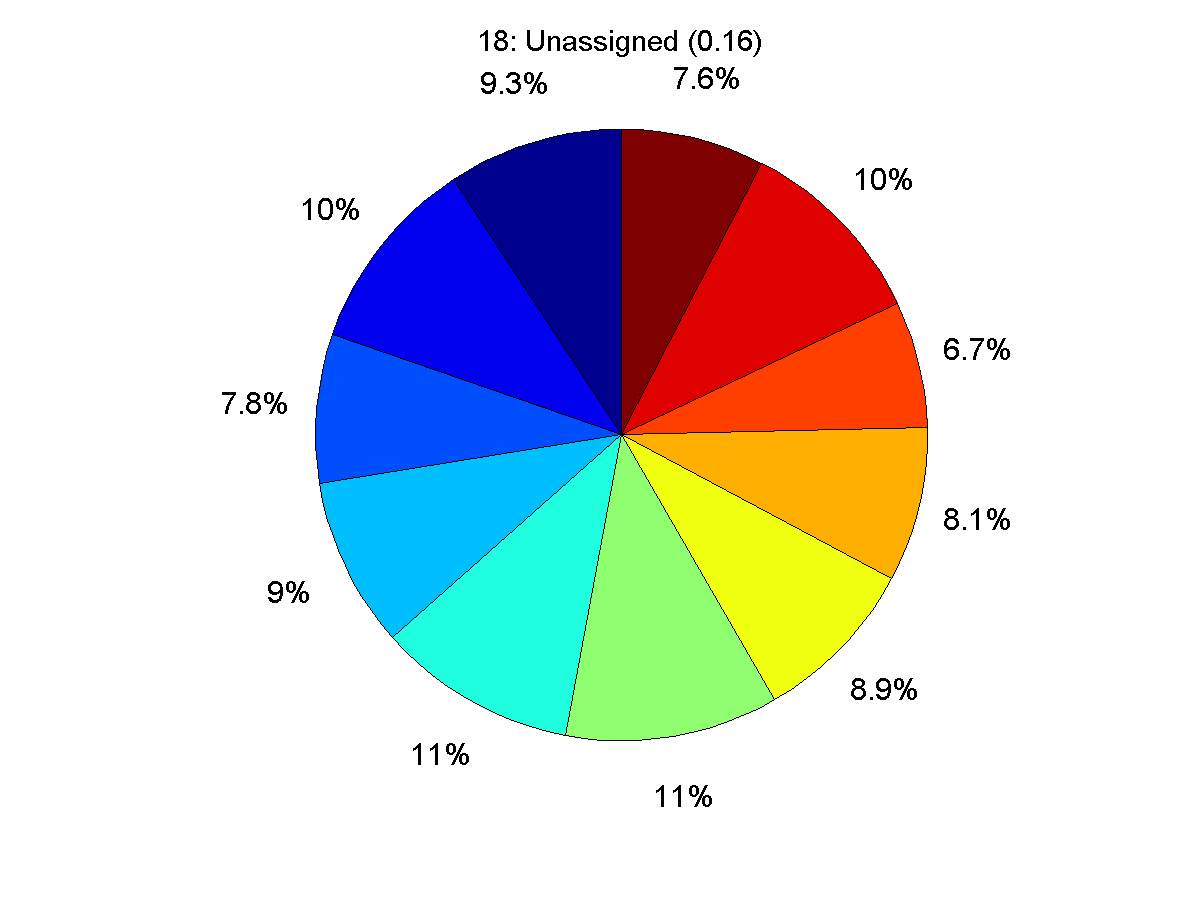

Supplement: S2 File — (ZIP) [file pone.0131875.s003.zip › MFC PieCharts/RegrEx2MFC/18Unassigned.tif]

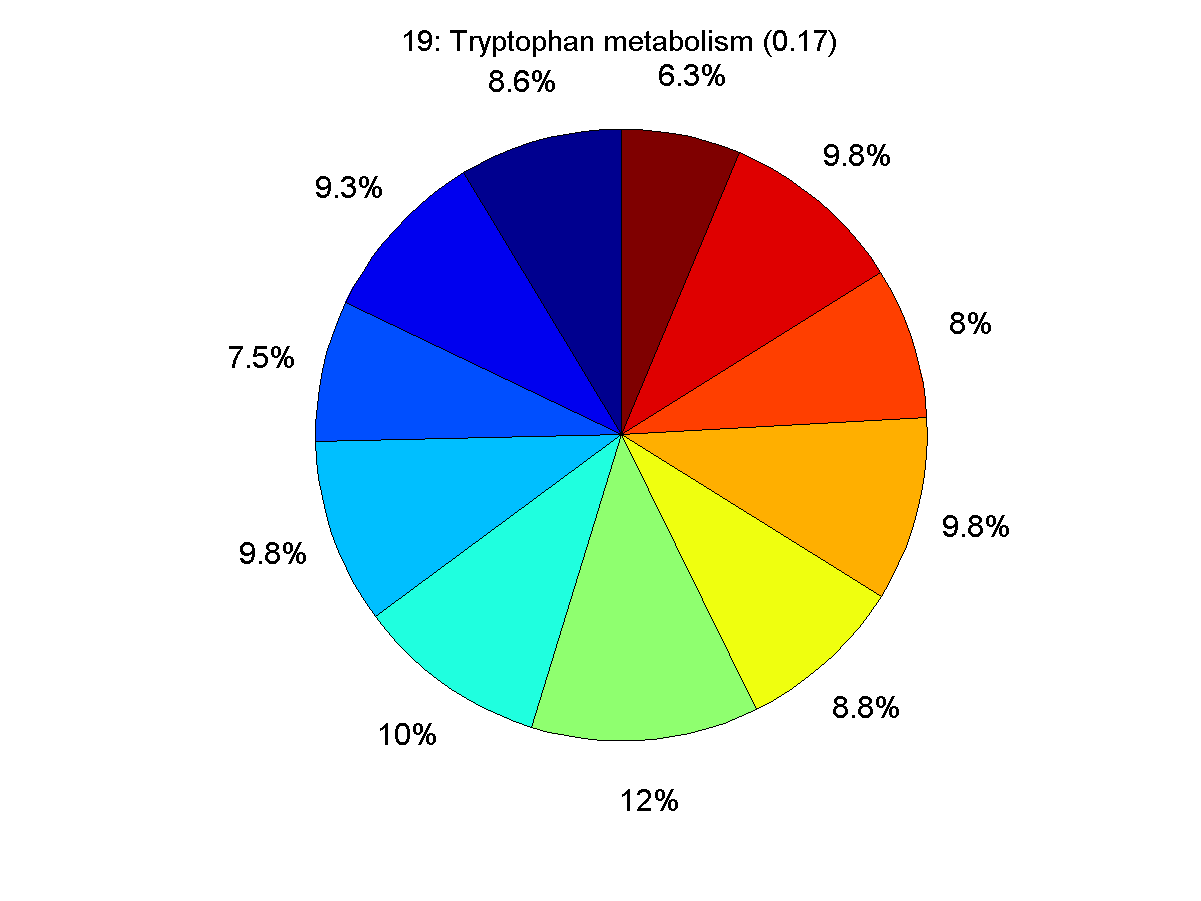

Supplement: S2 File — (ZIP) [file pone.0131875.s003.zip › MFC PieCharts/RegrEx2MFC/19Tryptophanmetabolism.tif]

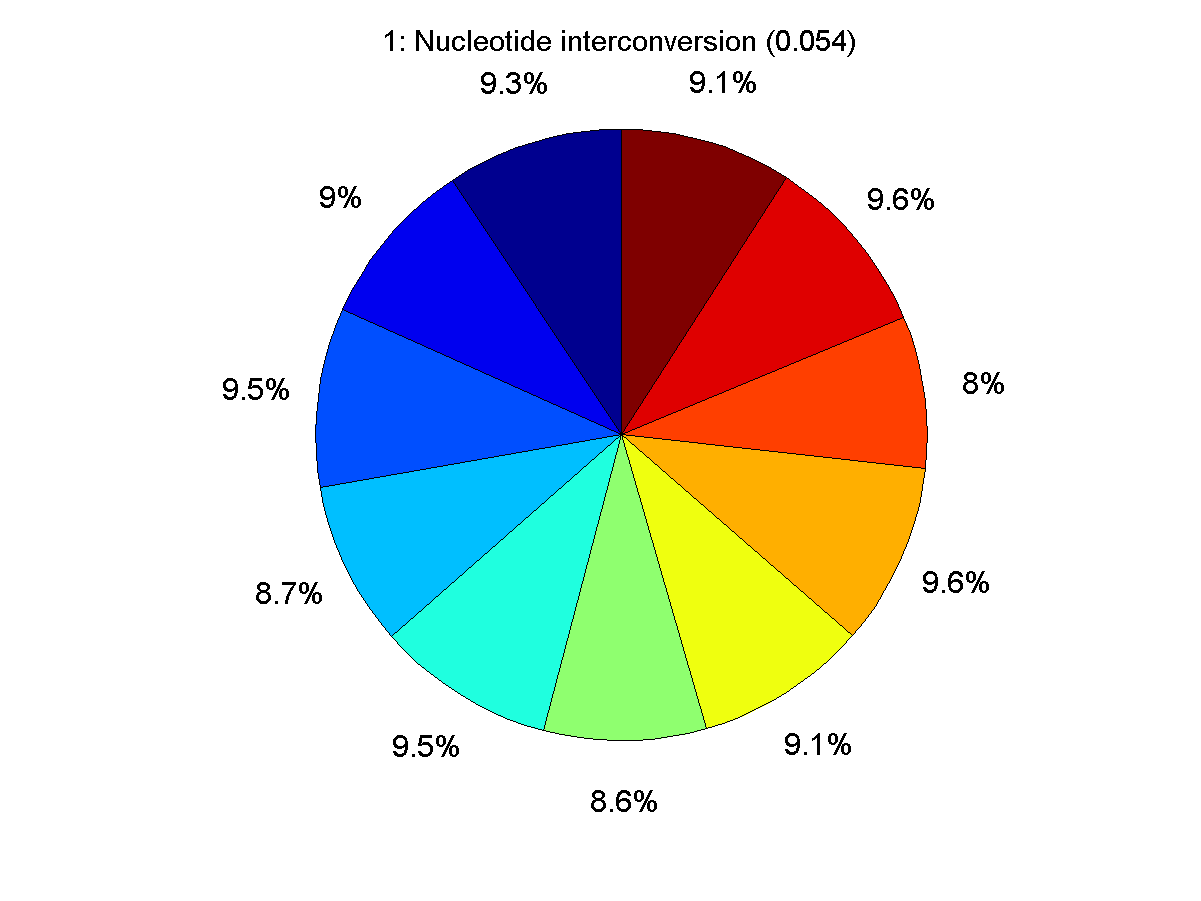

Supplement: S2 File — (ZIP) [file pone.0131875.s003.zip › MFC PieCharts/RegrEx2MFC/1Nucleotideinterconversion.tif]

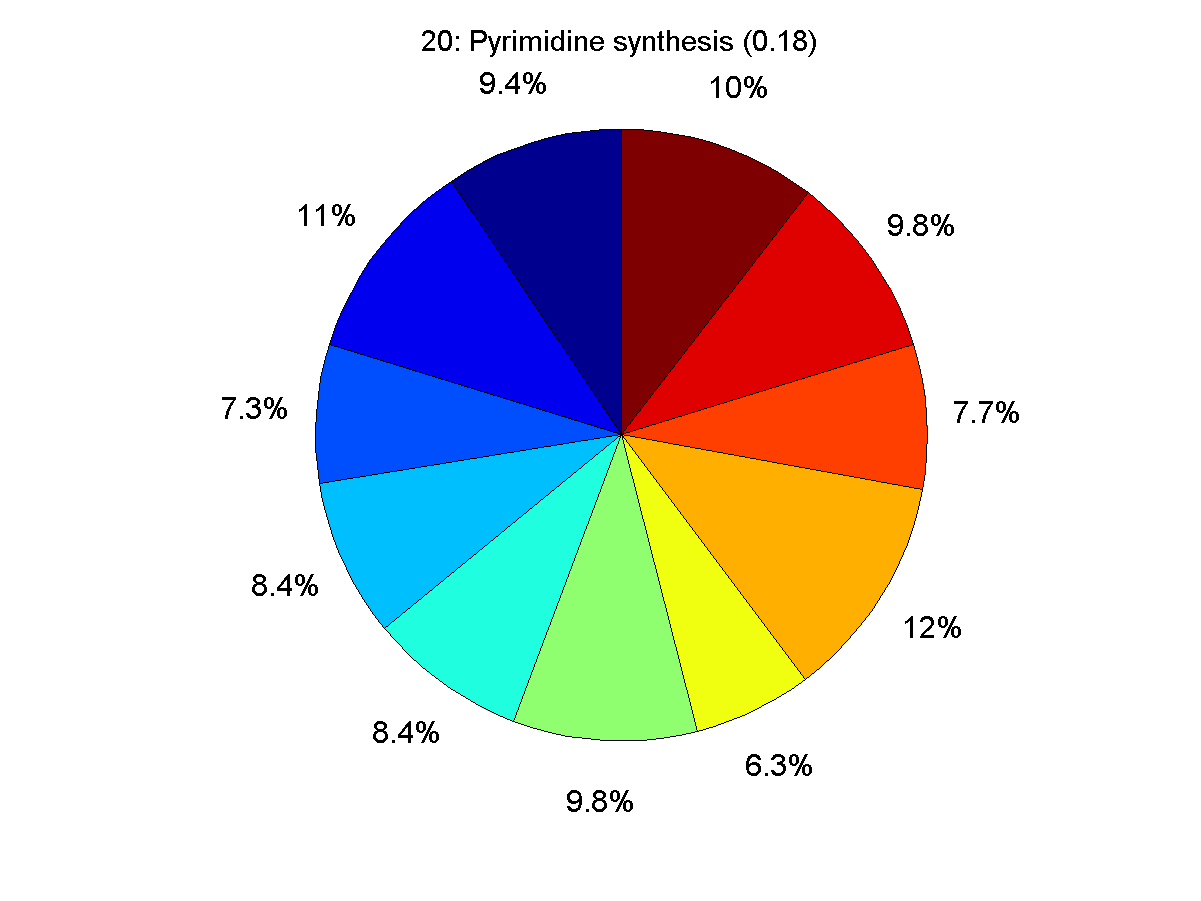

Supplement: S2 File — (ZIP) [file pone.0131875.s003.zip › MFC PieCharts/RegrEx2MFC/20Pyrimidinesynthesis.tif]

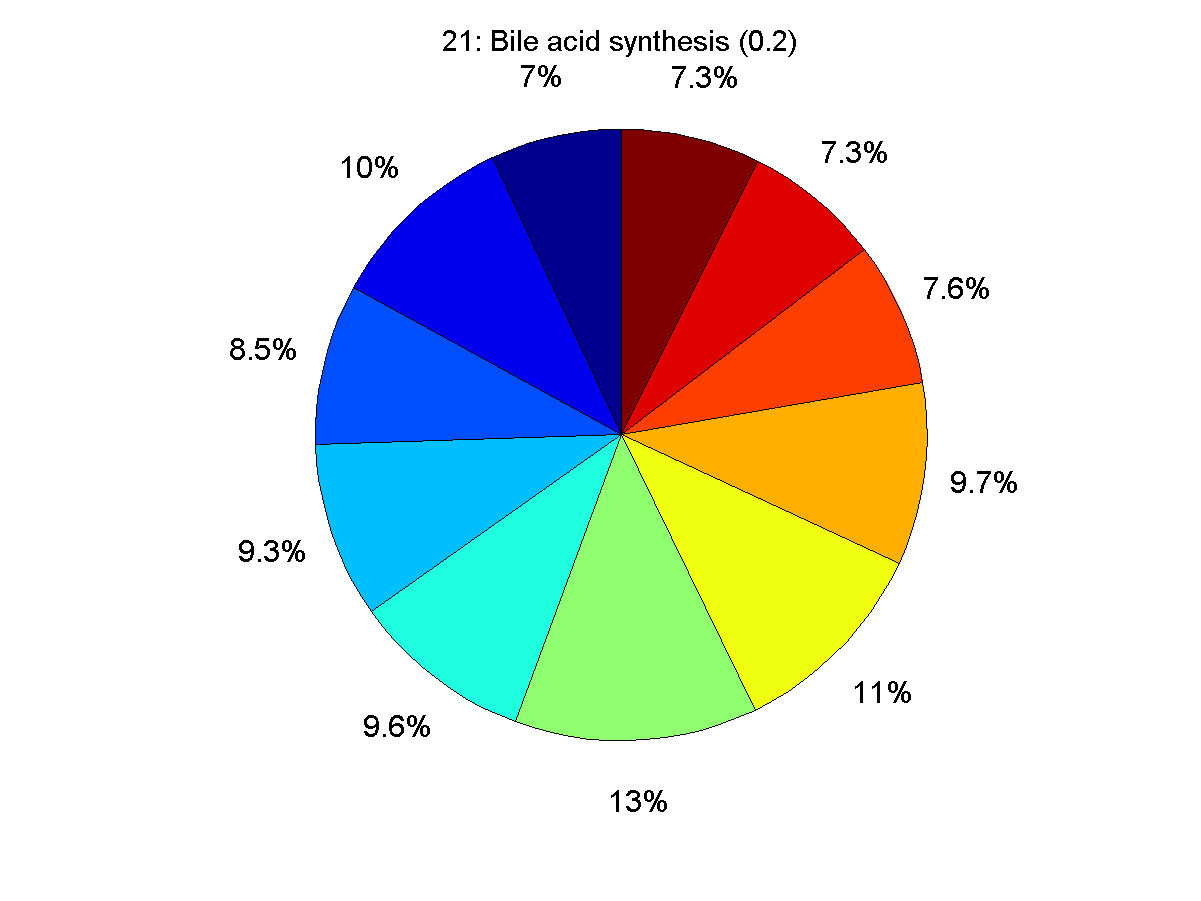

Supplement: S2 File — (ZIP) [file pone.0131875.s003.zip › MFC PieCharts/RegrEx2MFC/21Bileacidsynthesis.tif]

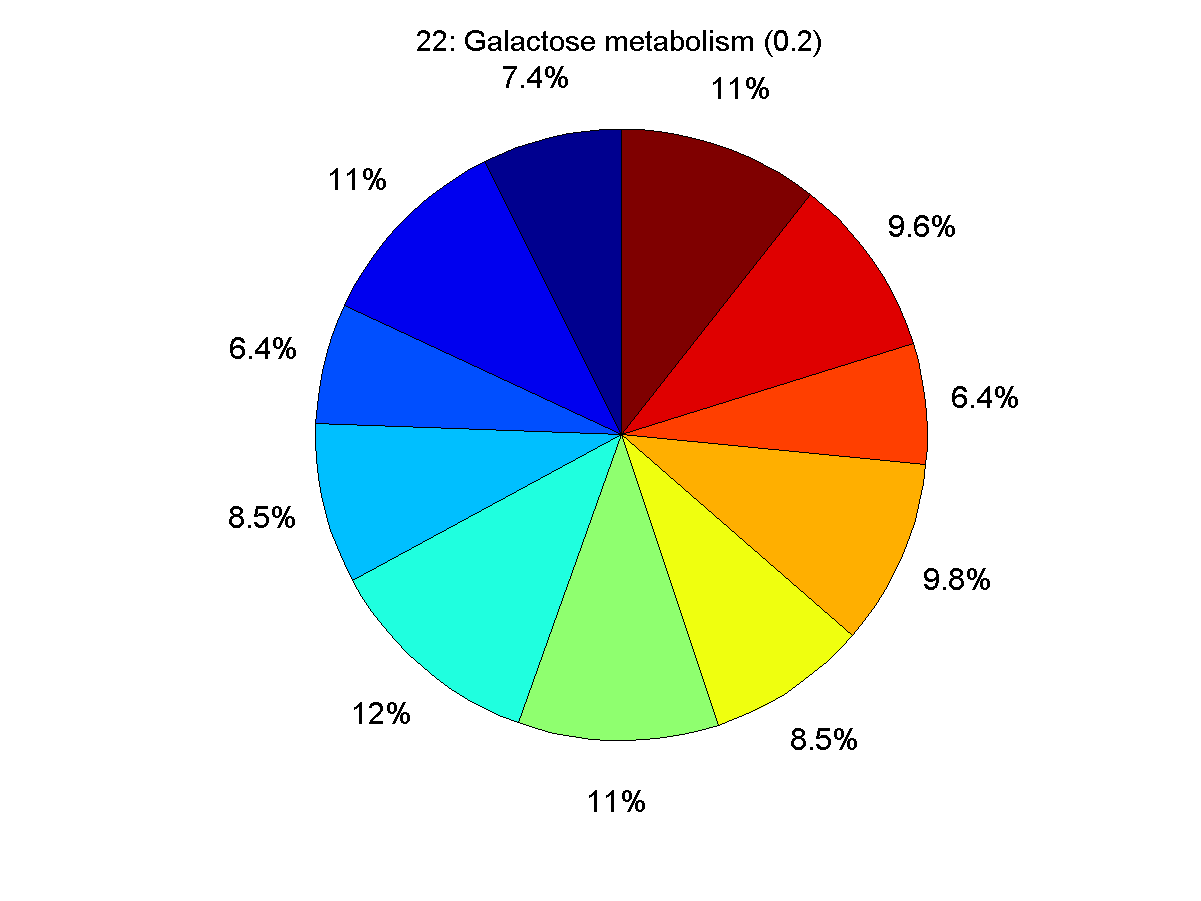

Supplement: S2 File — (ZIP) [file pone.0131875.s003.zip › MFC PieCharts/RegrEx2MFC/22Galactosemetabolism.tif]

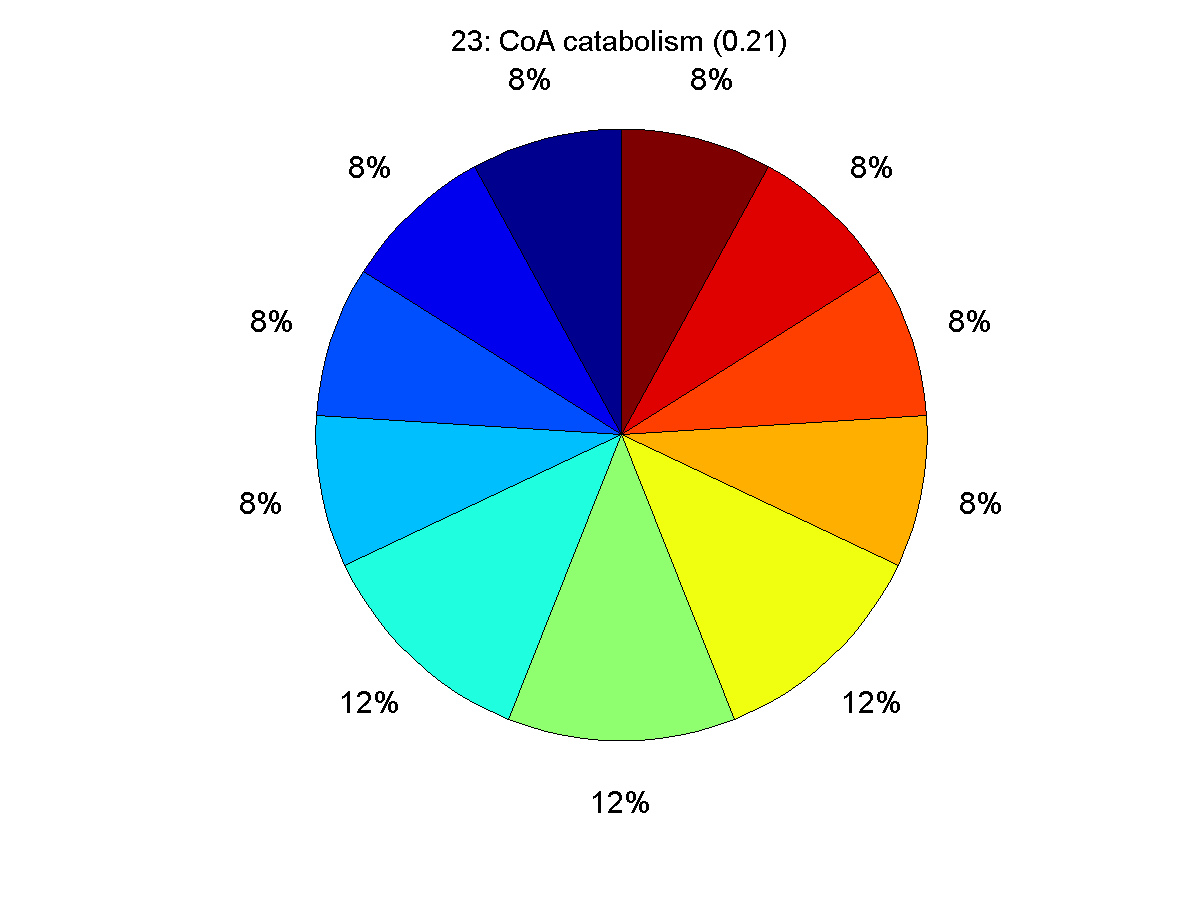

Supplement: S2 File — (ZIP) [file pone.0131875.s003.zip › MFC PieCharts/RegrEx2MFC/23CoAcatabolism.tif]

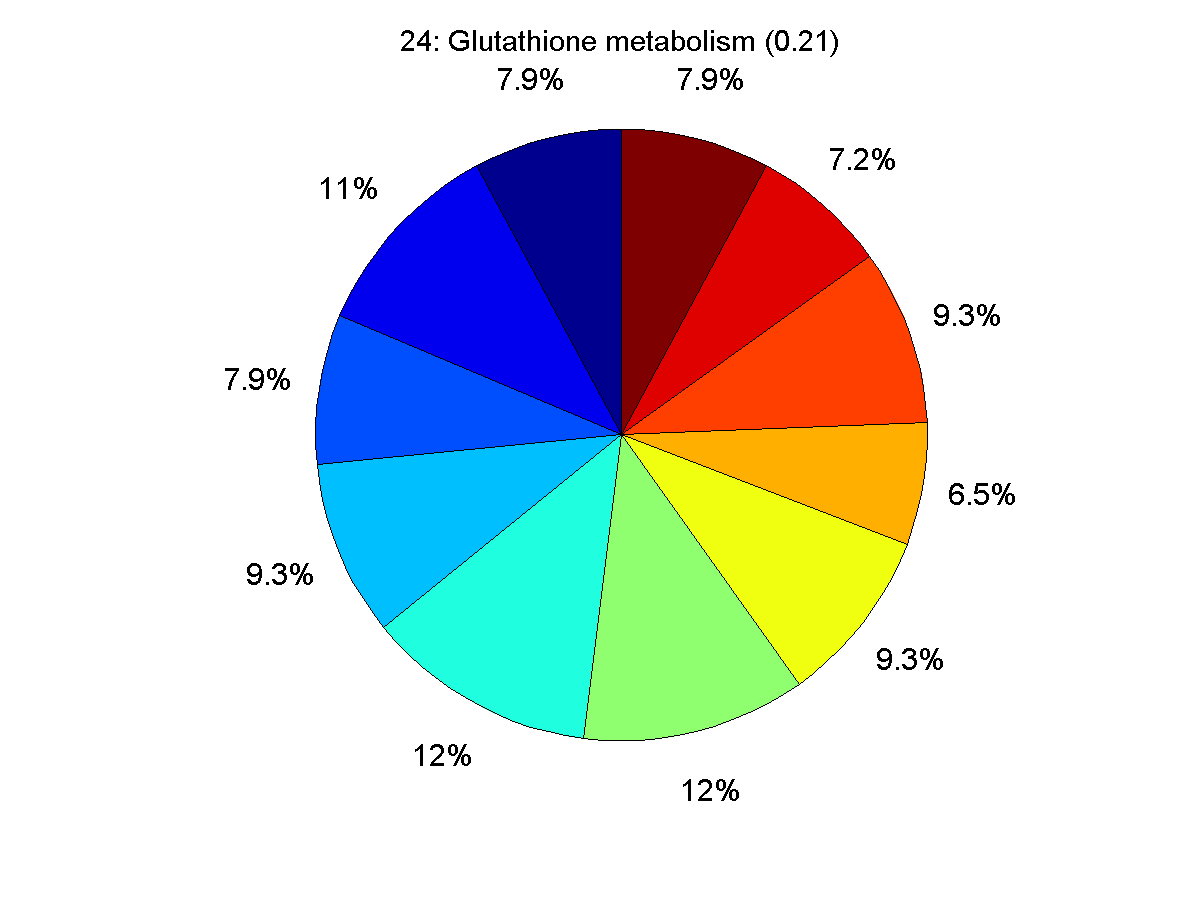

Supplement: S2 File — (ZIP) [file pone.0131875.s003.zip › MFC PieCharts/RegrEx2MFC/24Glutathionemetabolism.tif]

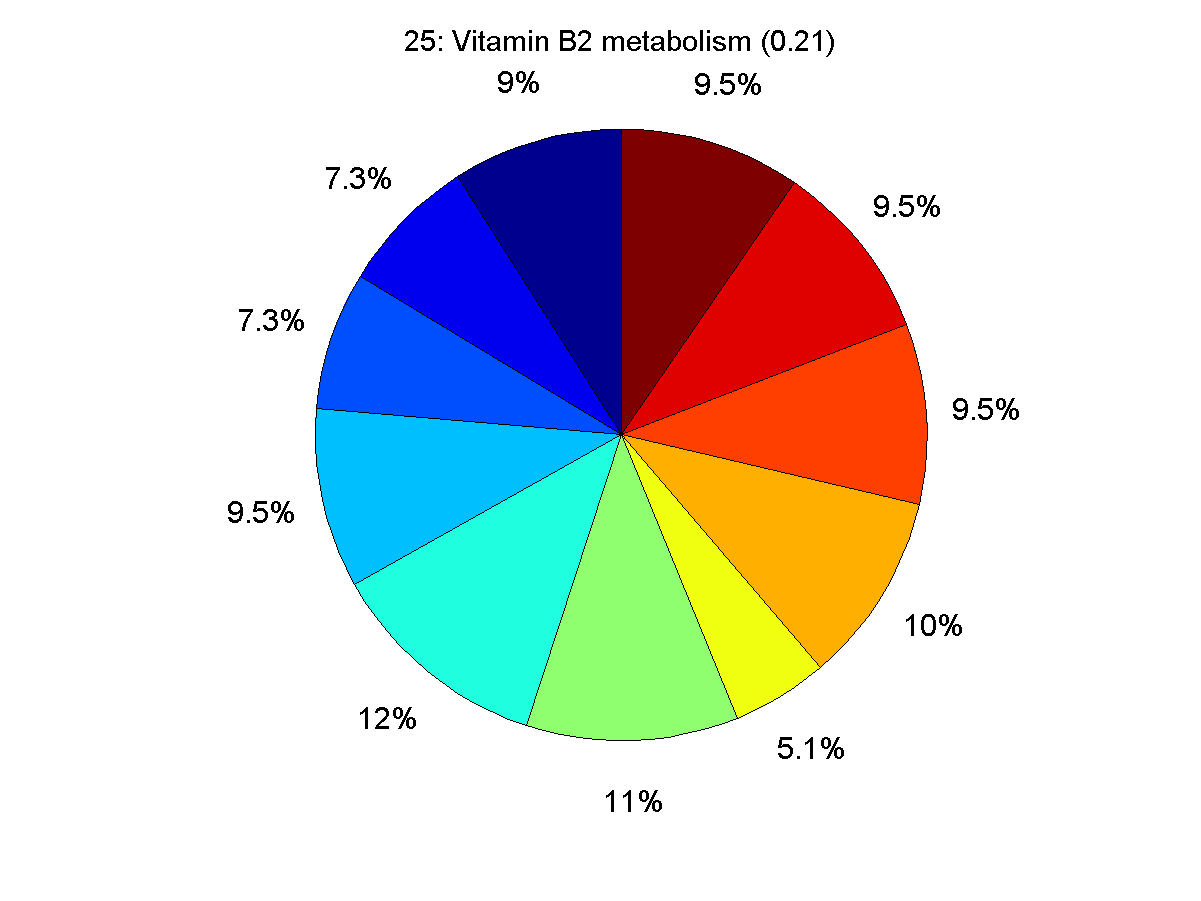

Supplement: S2 File — (ZIP) [file pone.0131875.s003.zip › MFC PieCharts/RegrEx2MFC/25VitaminB2metabolism.tif]

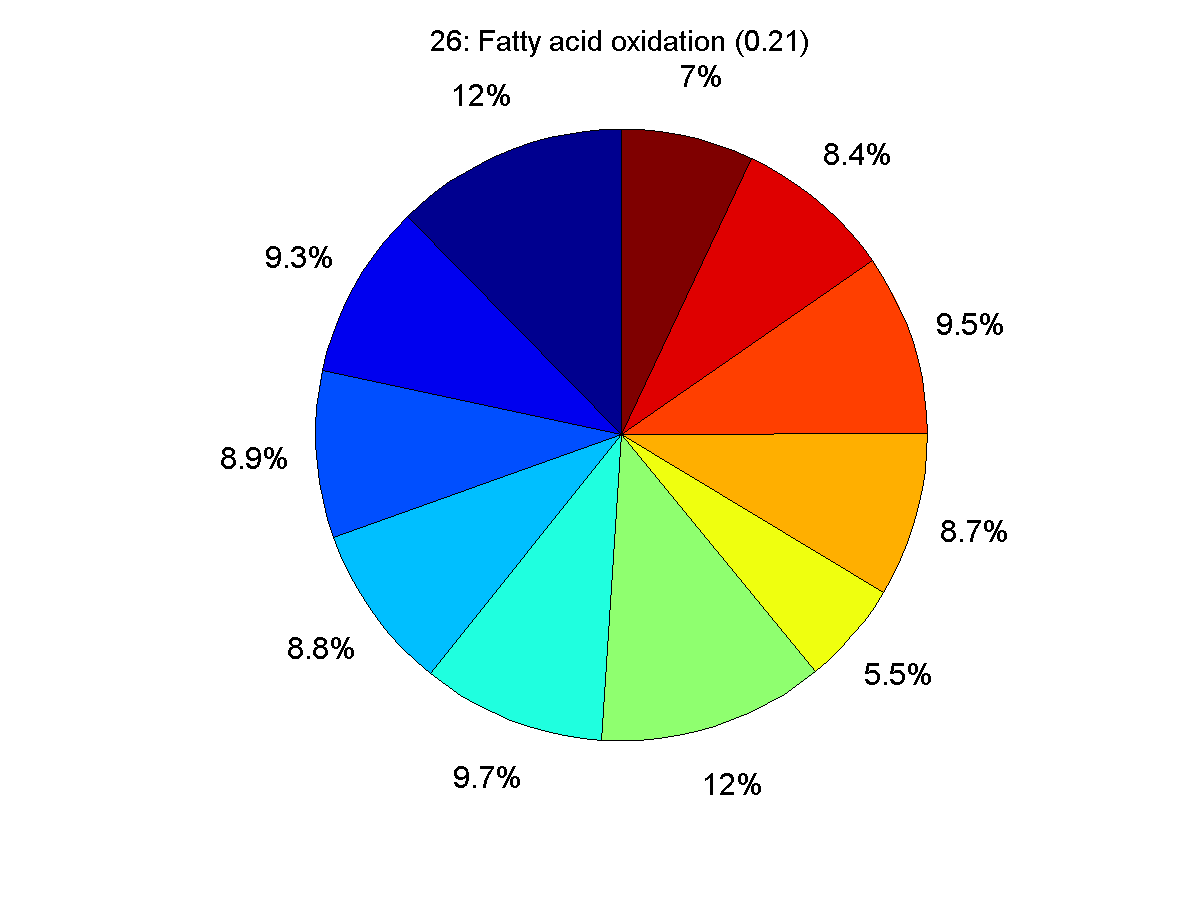

Supplement: S2 File — (ZIP) [file pone.0131875.s003.zip › MFC PieCharts/RegrEx2MFC/26Fattyacidoxidation.tif]

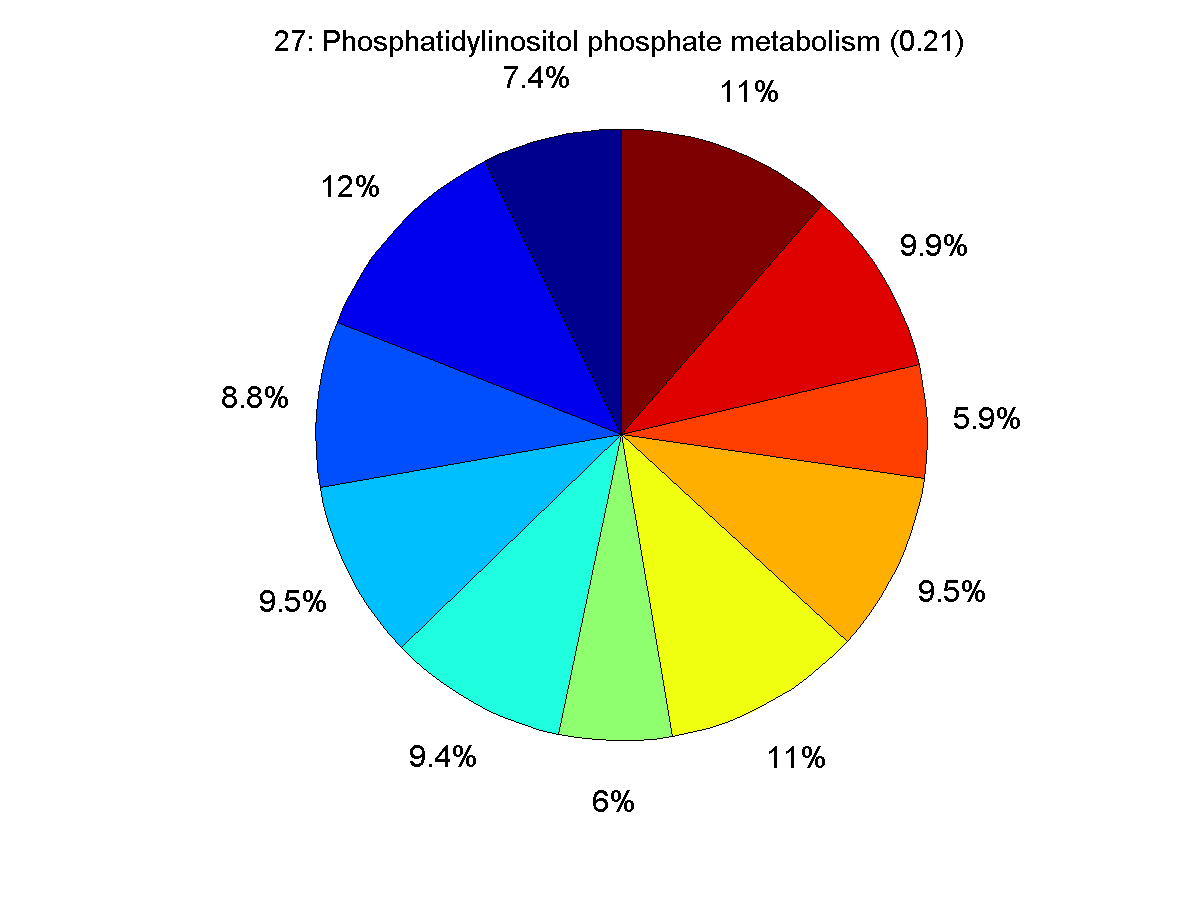

Supplement: S2 File — (ZIP) [file pone.0131875.s003.zip › MFC PieCharts/RegrEx2MFC/27Phosphatidylinositolphosphatemetabolism.tif]

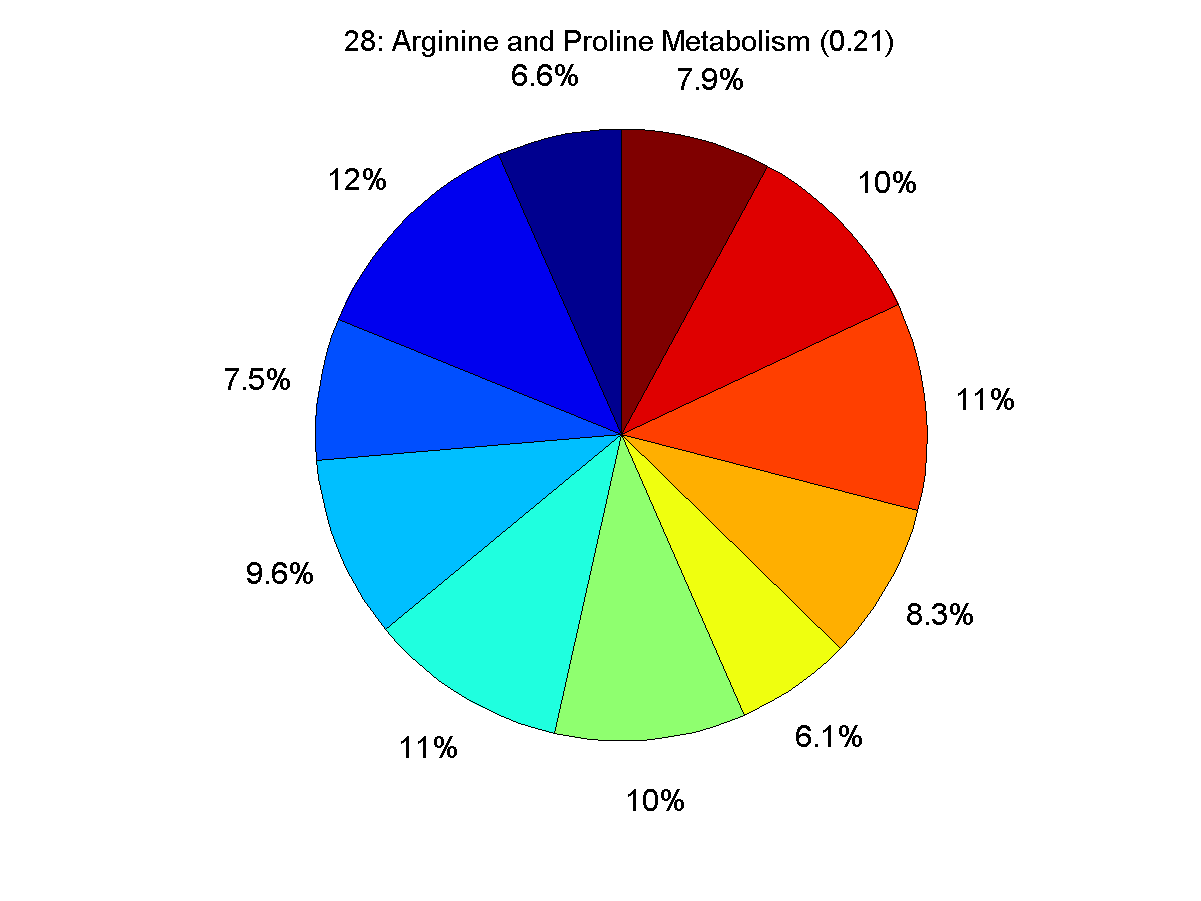

Supplement: S2 File — (ZIP) [file pone.0131875.s003.zip › MFC PieCharts/RegrEx2MFC/28ArginineandProlineMetabolism.tif]

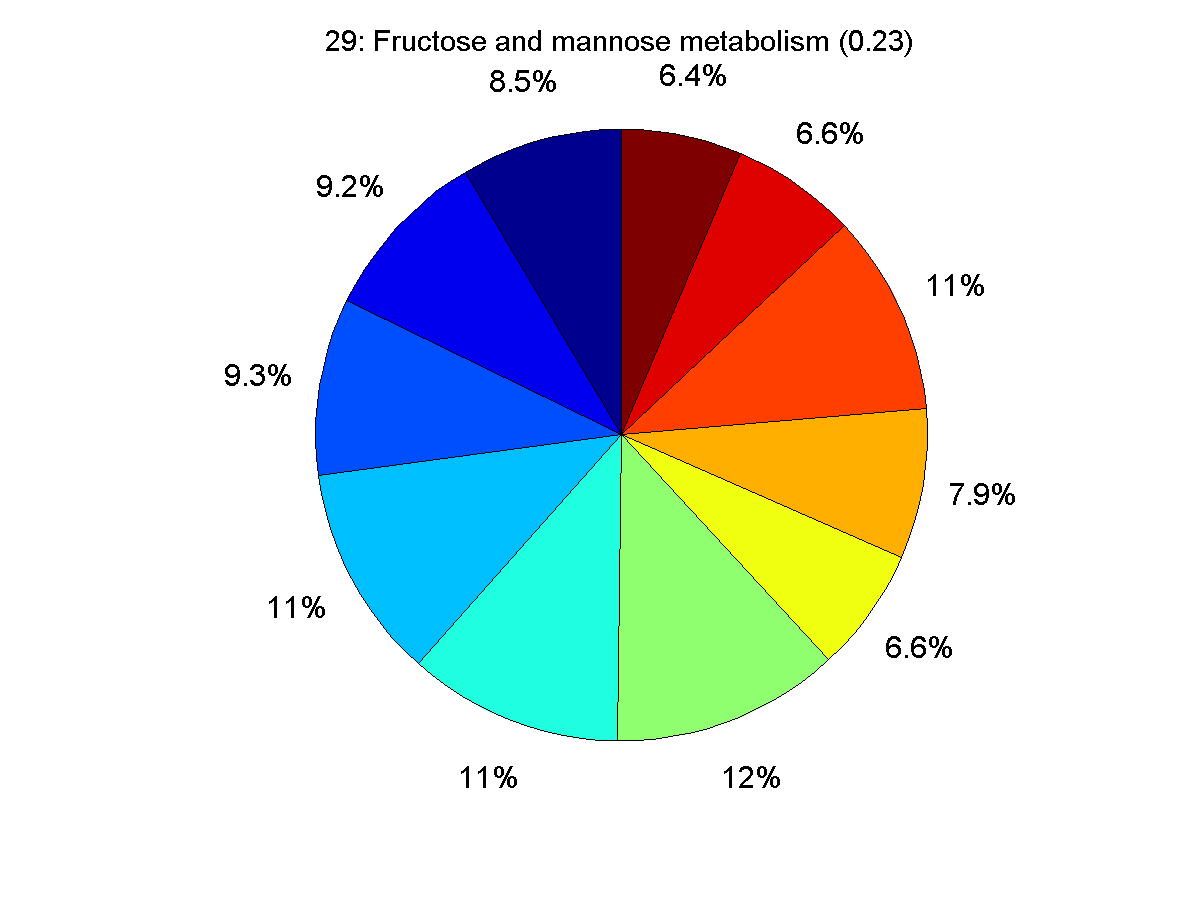

Supplement: S2 File — (ZIP) [file pone.0131875.s003.zip › MFC PieCharts/RegrEx2MFC/29Fructoseandmannosemetabolism.tif]

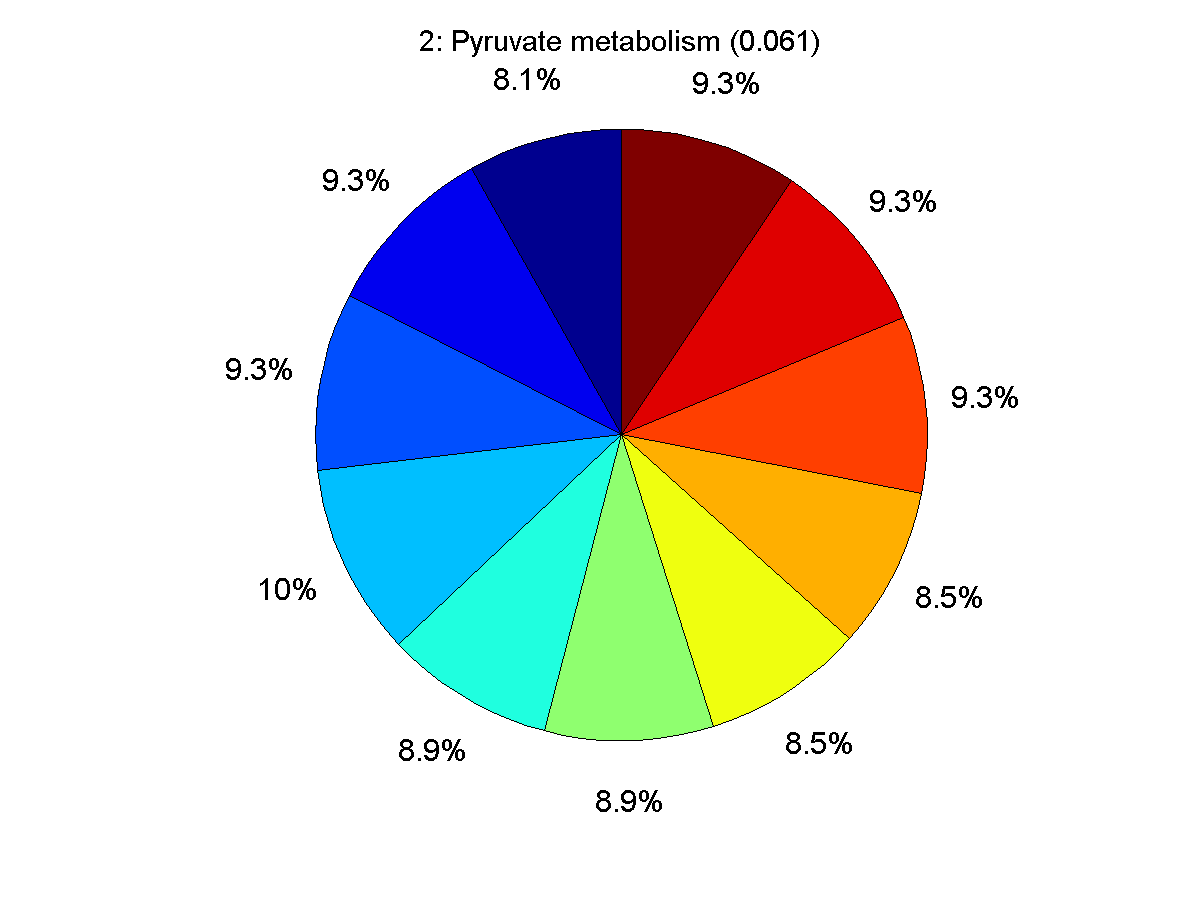

Supplement: S2 File — (ZIP) [file pone.0131875.s003.zip › MFC PieCharts/RegrEx2MFC/2Pyruvatemetabolism.tif]

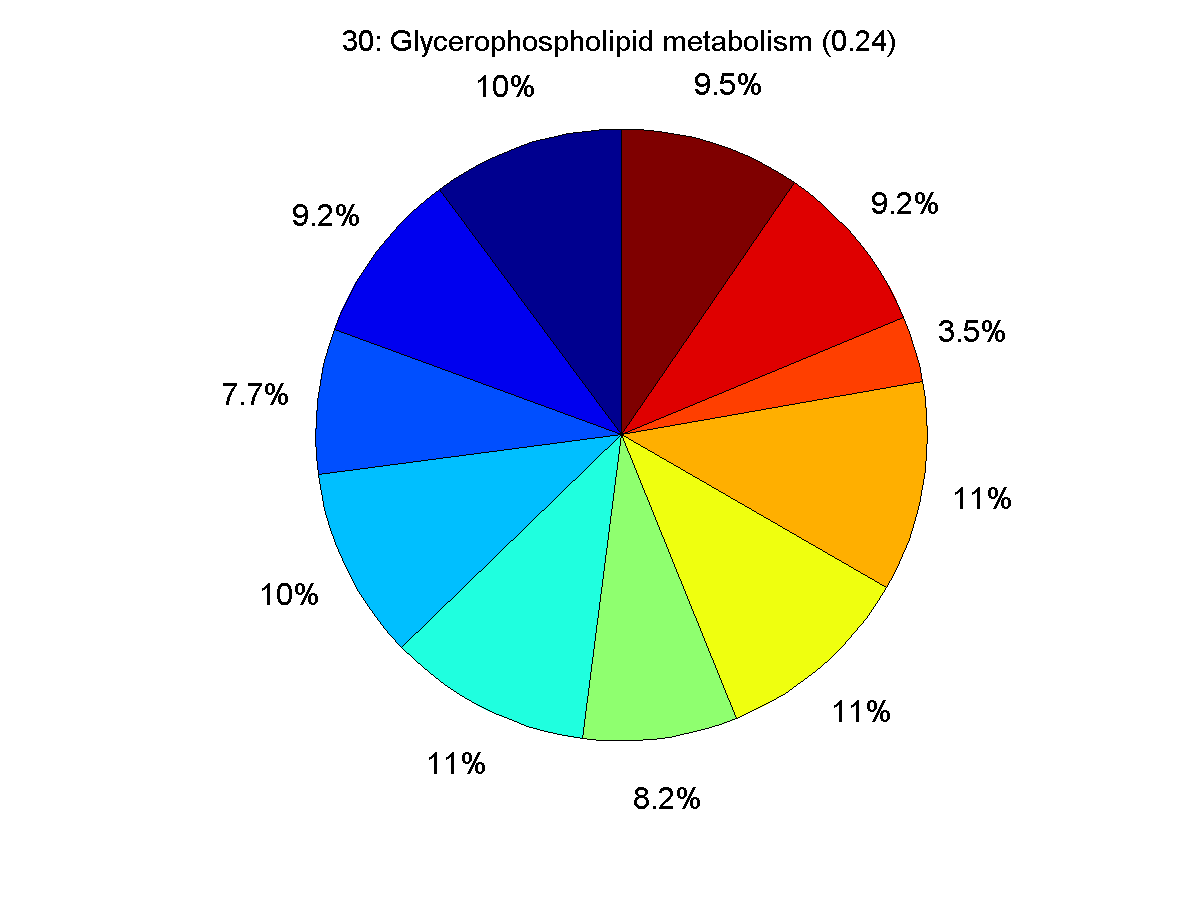

Supplement: S2 File — (ZIP) [file pone.0131875.s003.zip › MFC PieCharts/RegrEx2MFC/30Glycerophospholipidmetabolism.tif]
