# Supplementary figures and images for: Context-Specific Metabolic Model Extraction Based on Regularized Least Squares Optimization (part 2 of 2)
Source: PLoS One. 2015 Jul 9;10(7):e0131875. doi: 10.1371/journal.pone.0131875 (PMC4497637; doi:10.1371/journal.pone.0131875)

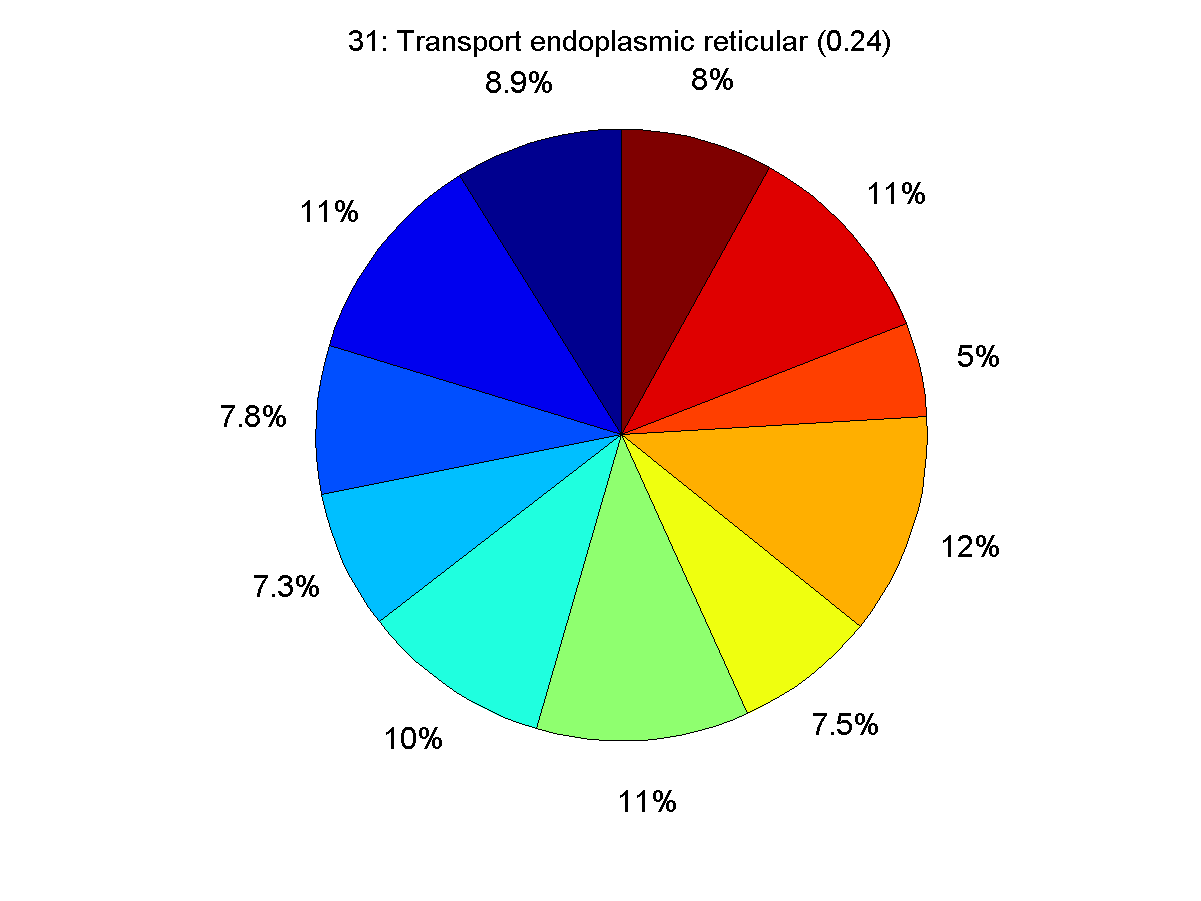

Supplement: S2 File — (ZIP) [file pone.0131875.s003.zip › MFC PieCharts/RegrEx2MFC/31Transportendoplasmicreticular.tif]

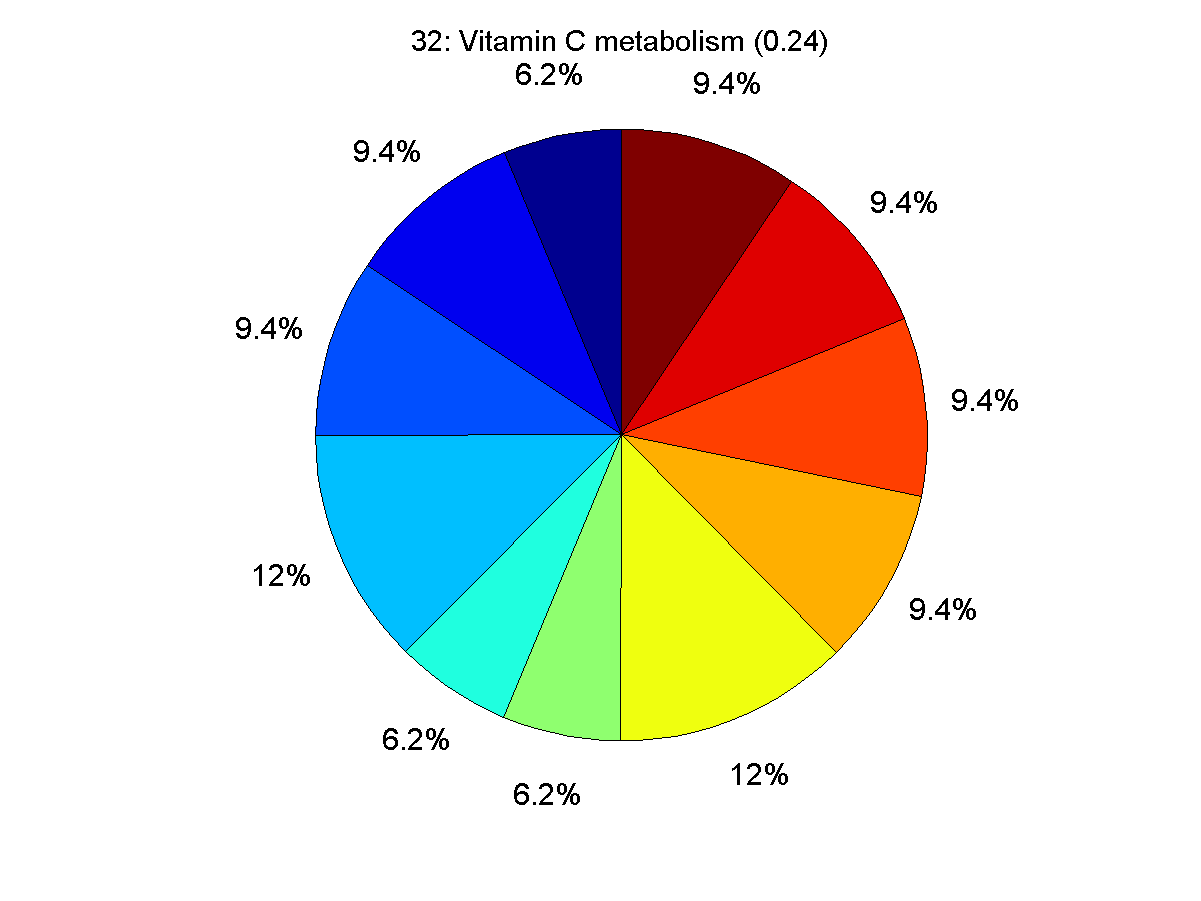

Supplement: S2 File — (ZIP) [file pone.0131875.s003.zip › MFC PieCharts/RegrEx2MFC/32VitaminCmetabolism.tif]

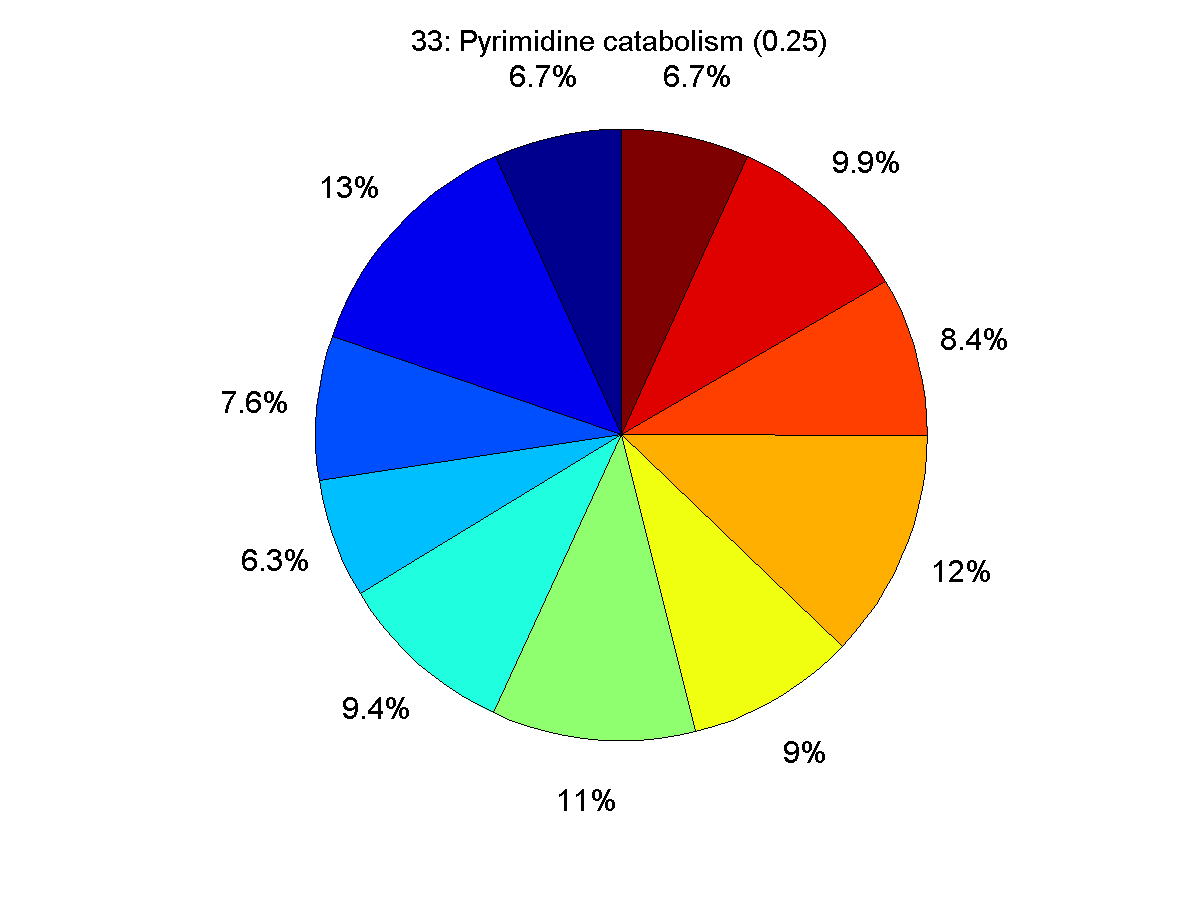

Supplement: S2 File — (ZIP) [file pone.0131875.s003.zip › MFC PieCharts/RegrEx2MFC/33Pyrimidinecatabolism.tif]

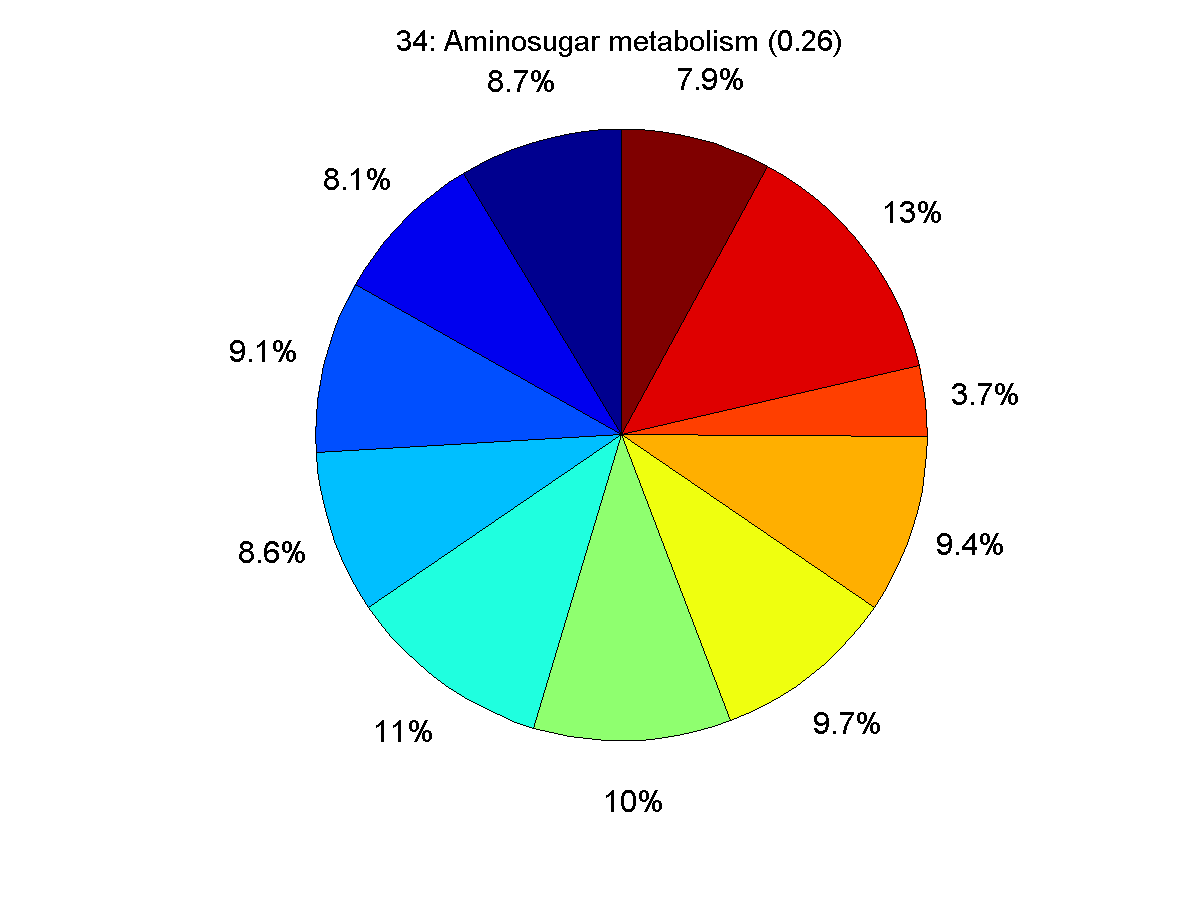

Supplement: S2 File — (ZIP) [file pone.0131875.s003.zip › MFC PieCharts/RegrEx2MFC/34Aminosugarmetabolism.tif]

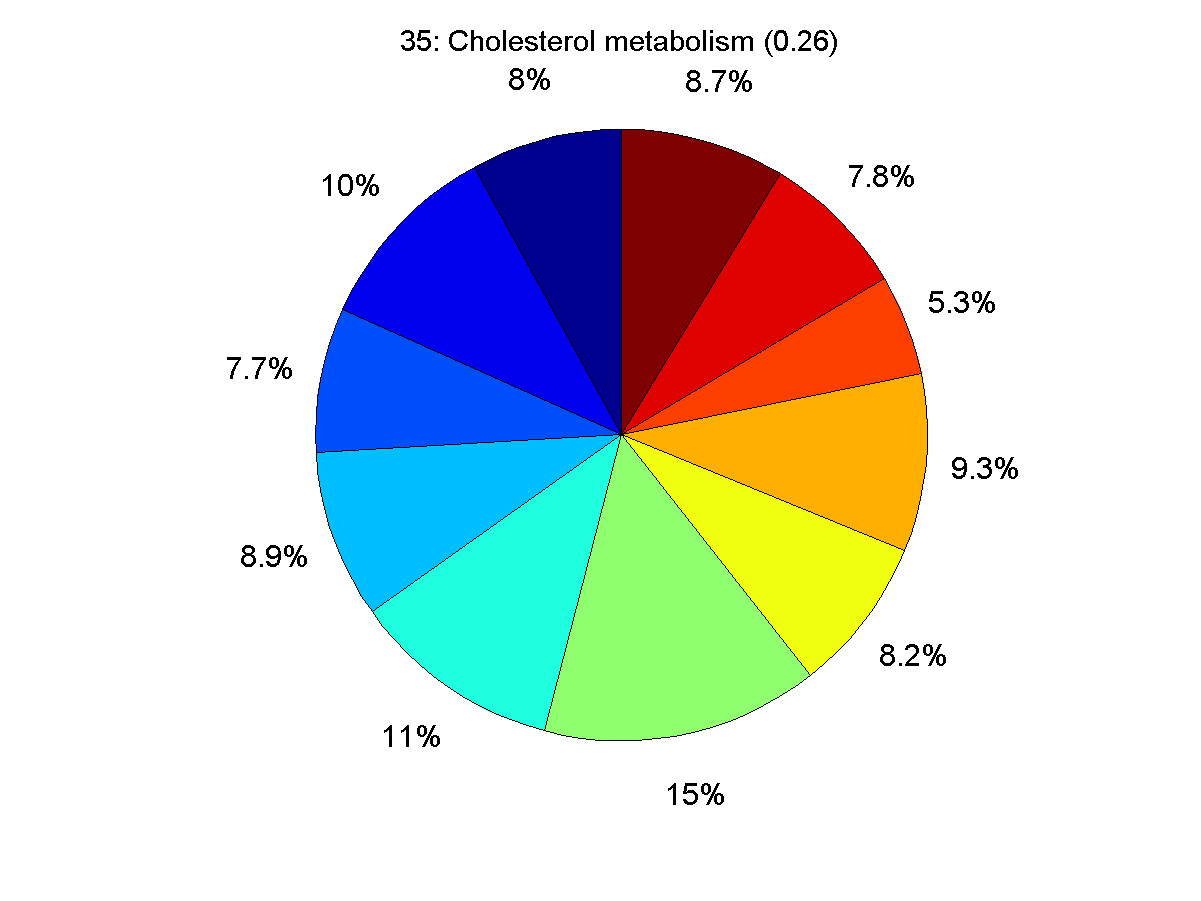

Supplement: S2 File — (ZIP) [file pone.0131875.s003.zip › MFC PieCharts/RegrEx2MFC/35Cholesterolmetabolism.tif]

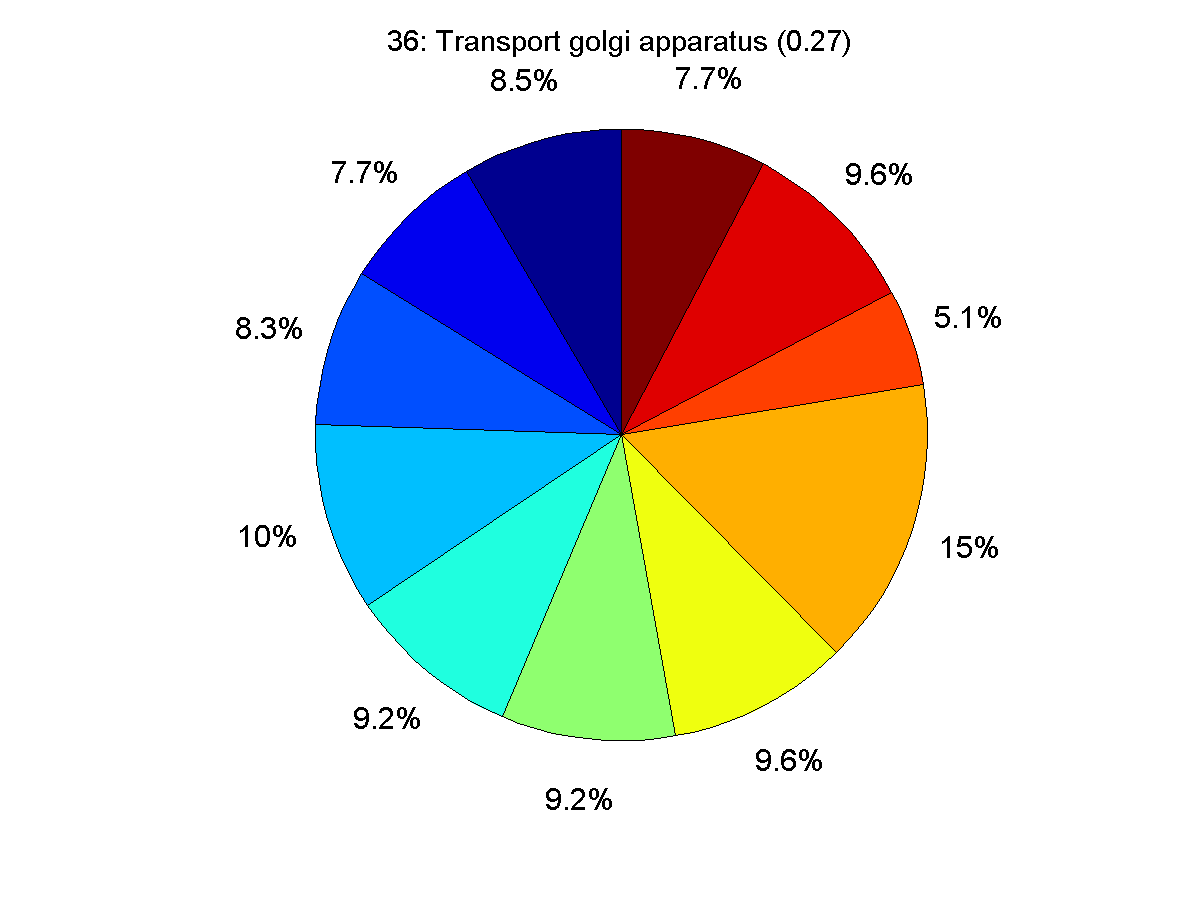

Supplement: S2 File — (ZIP) [file pone.0131875.s003.zip › MFC PieCharts/RegrEx2MFC/36Transportgolgiapparatus.tif]

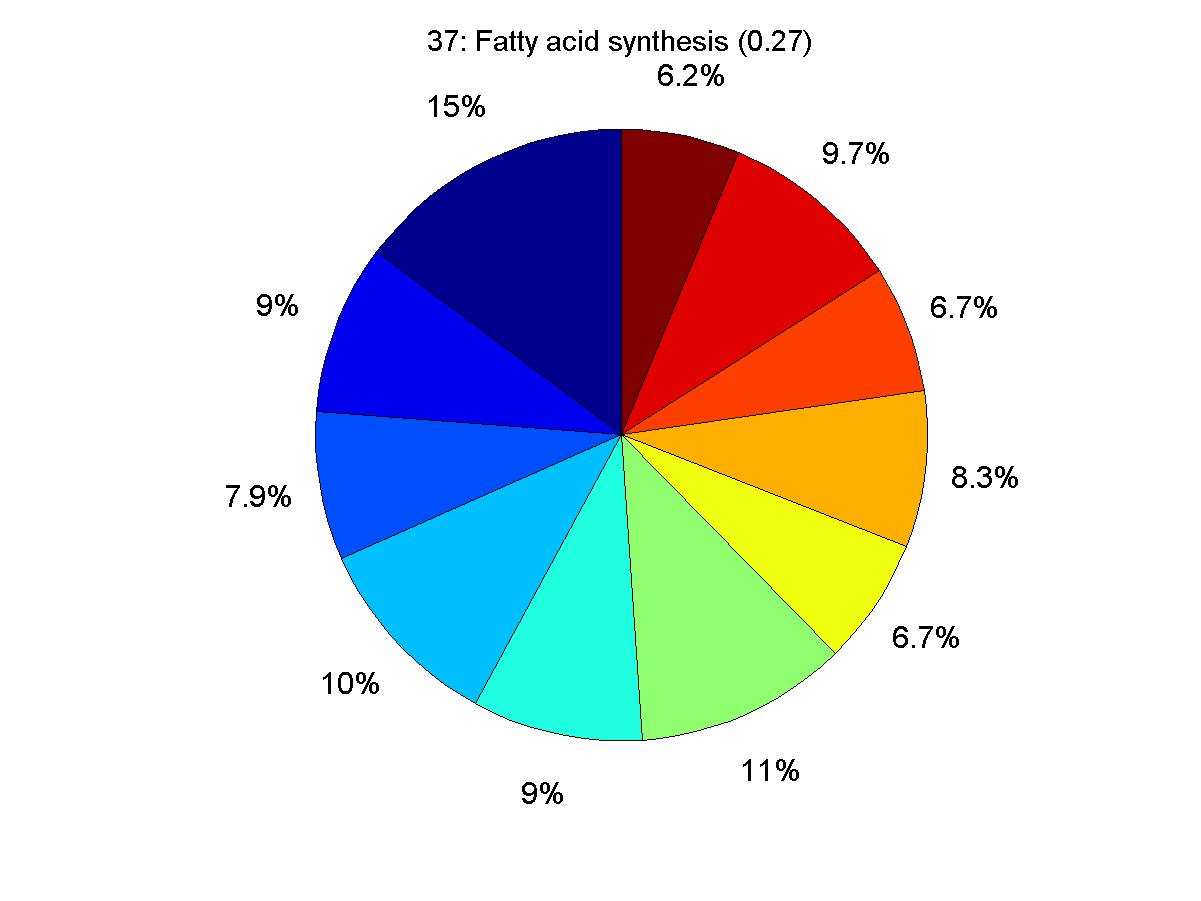

Supplement: S2 File — (ZIP) [file pone.0131875.s003.zip › MFC PieCharts/RegrEx2MFC/37Fattyacidsynthesis.tif]

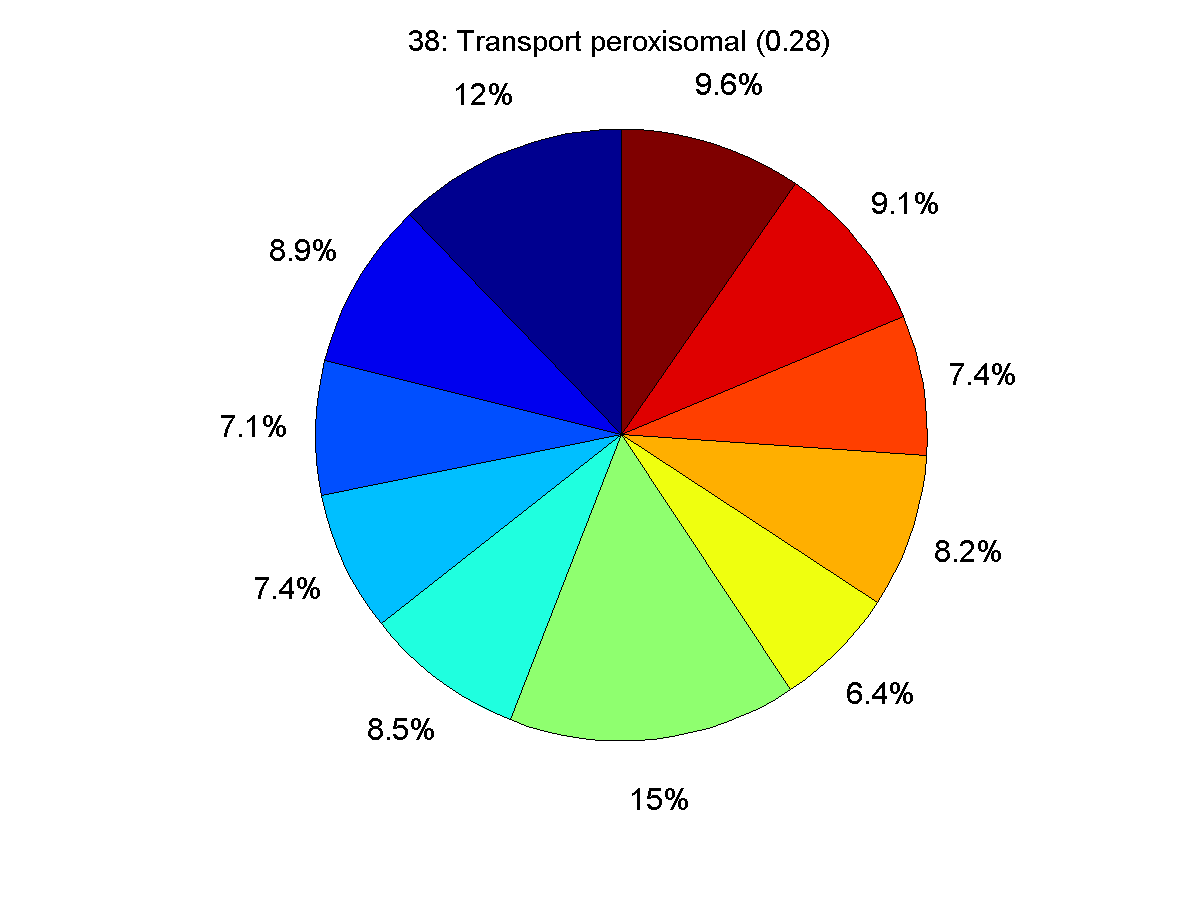

Supplement: S2 File — (ZIP) [file pone.0131875.s003.zip › MFC PieCharts/RegrEx2MFC/38Transportperoxisomal.tif]

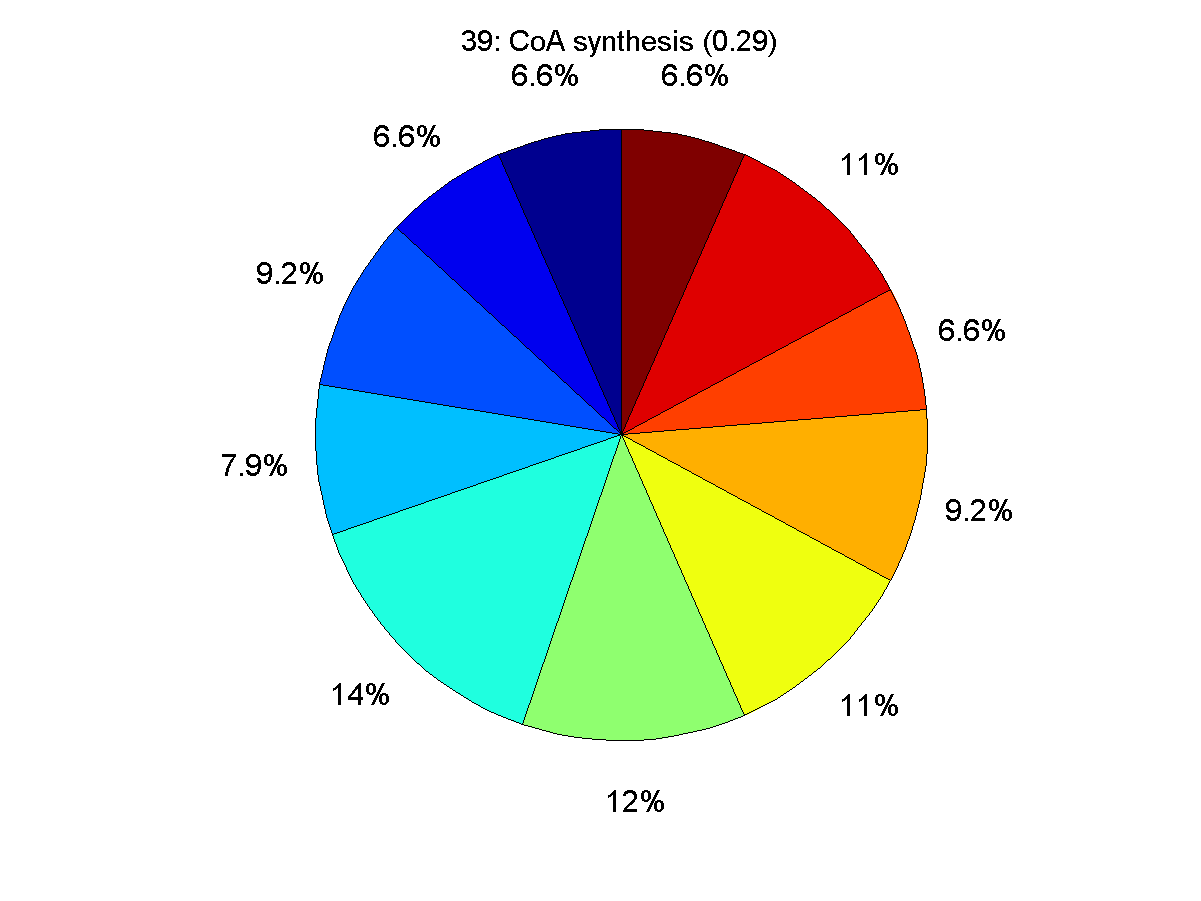

Supplement: S2 File — (ZIP) [file pone.0131875.s003.zip › MFC PieCharts/RegrEx2MFC/39CoAsynthesis.tif]

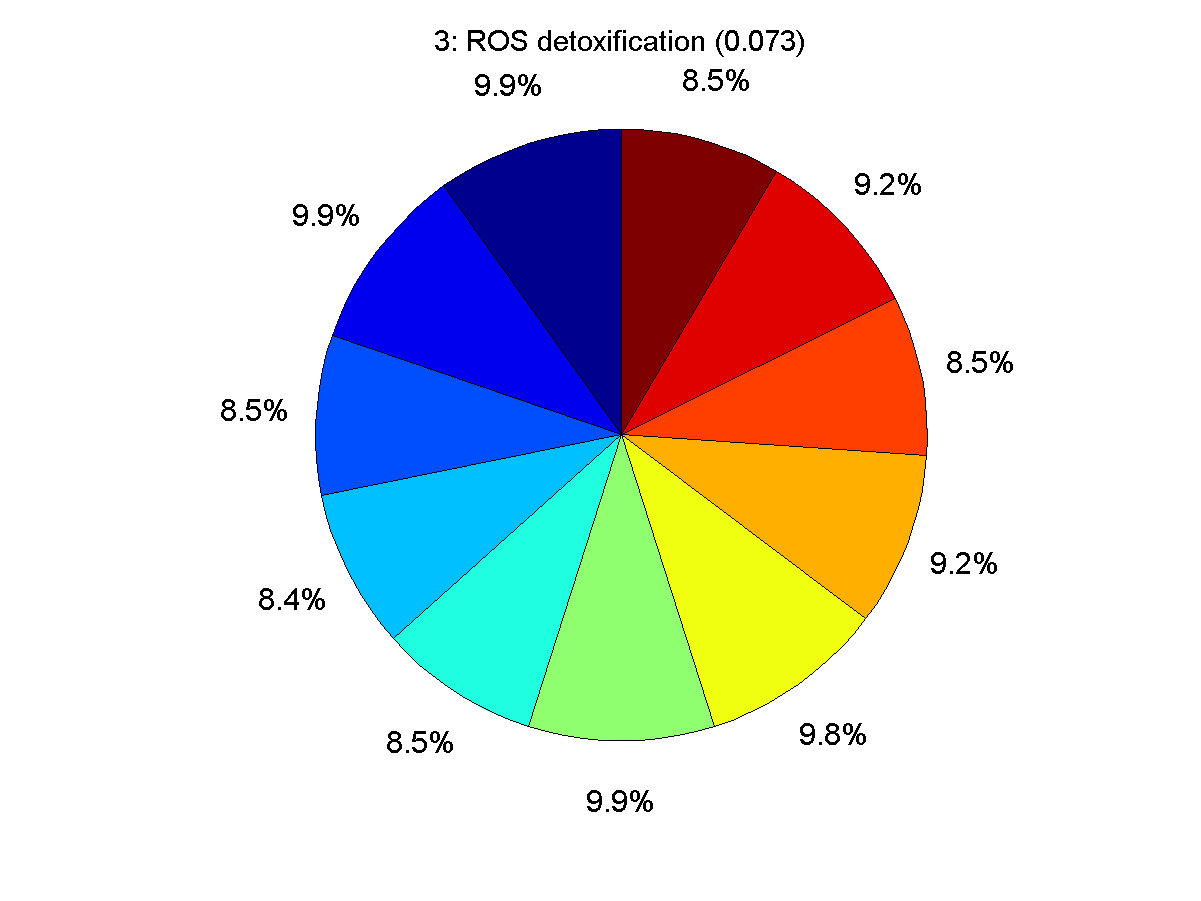

Supplement: S2 File — (ZIP) [file pone.0131875.s003.zip › MFC PieCharts/RegrEx2MFC/3ROSdetoxification.tif]

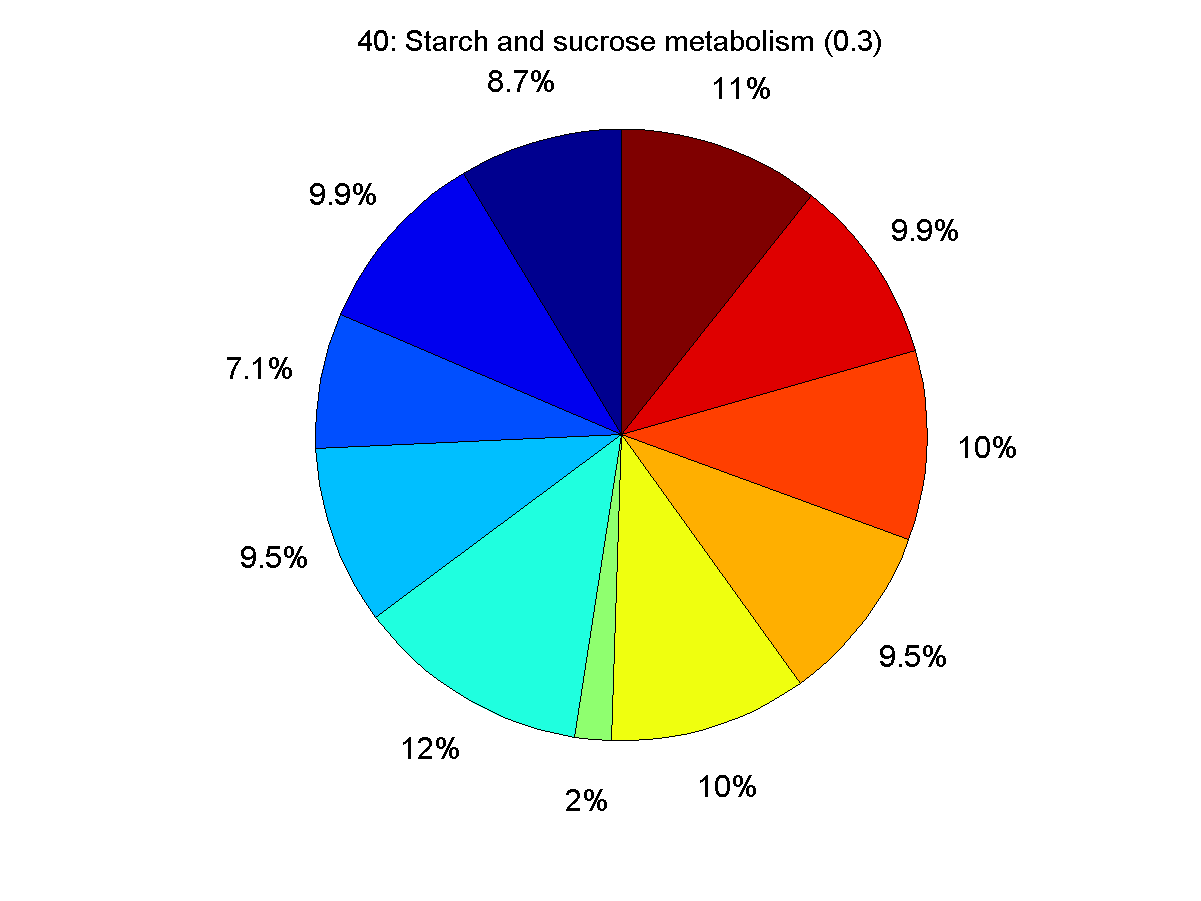

Supplement: S2 File — (ZIP) [file pone.0131875.s003.zip › MFC PieCharts/RegrEx2MFC/40Starchandsucrosemetabolism.tif]

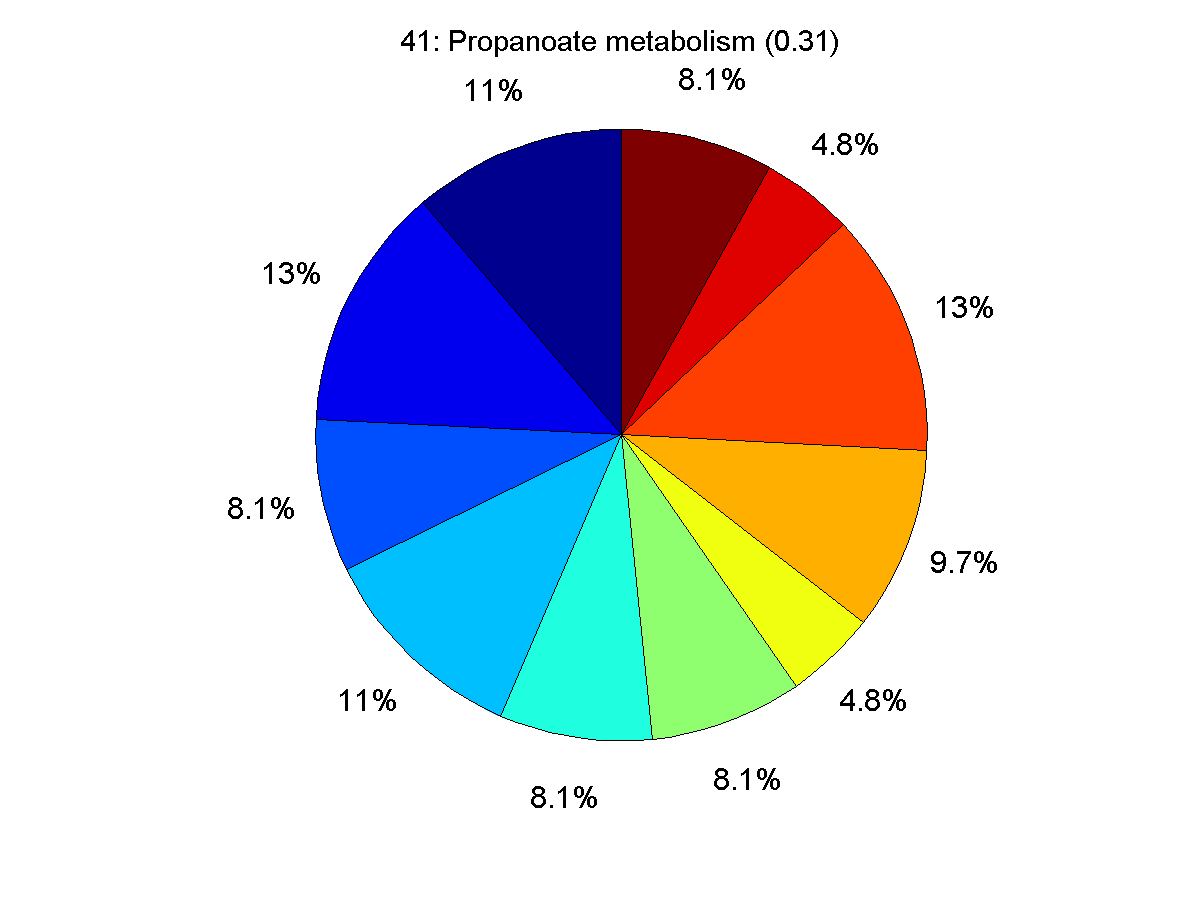

Supplement: S2 File — (ZIP) [file pone.0131875.s003.zip › MFC PieCharts/RegrEx2MFC/41Propanoatemetabolism.tif]

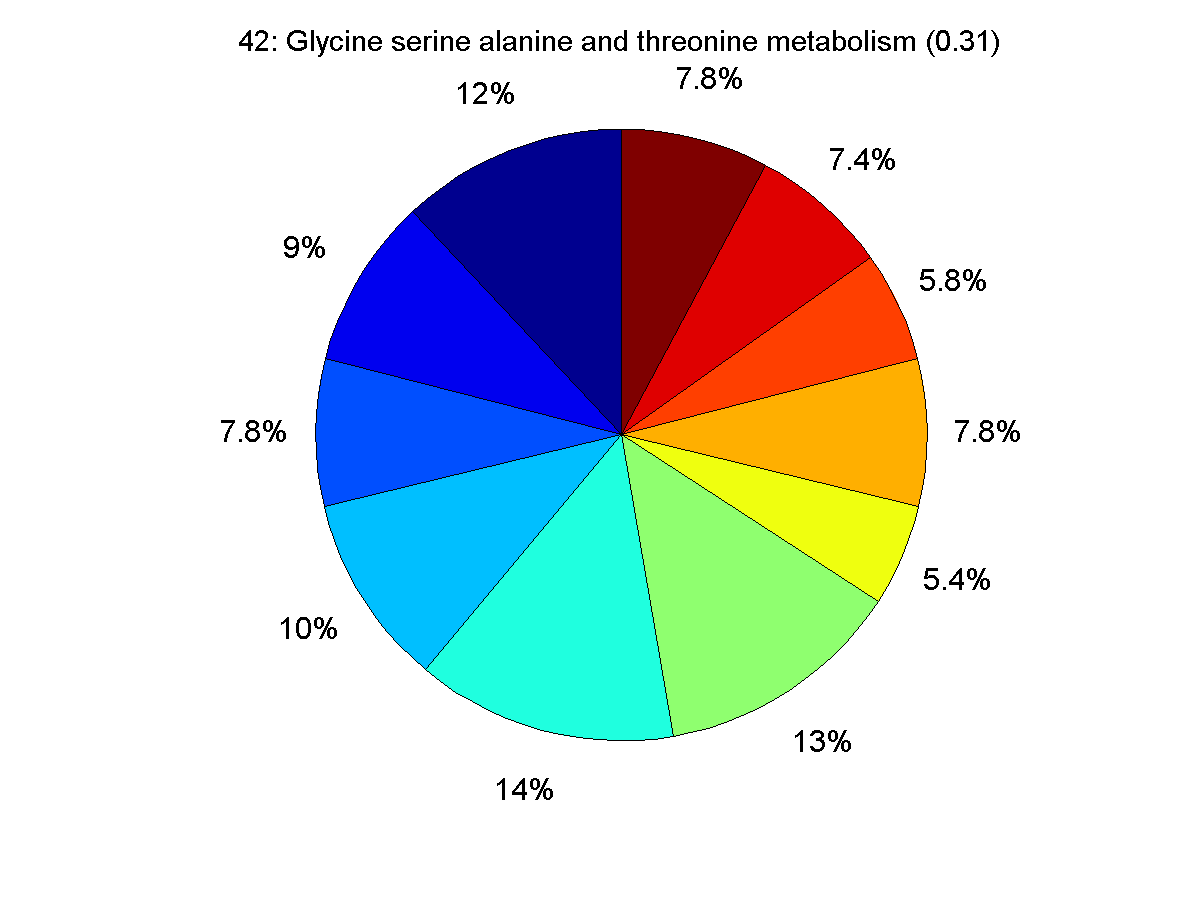

Supplement: S2 File — (ZIP) [file pone.0131875.s003.zip › MFC PieCharts/RegrEx2MFC/42Glycineserinealanineandthreoninemetabolism.tif]

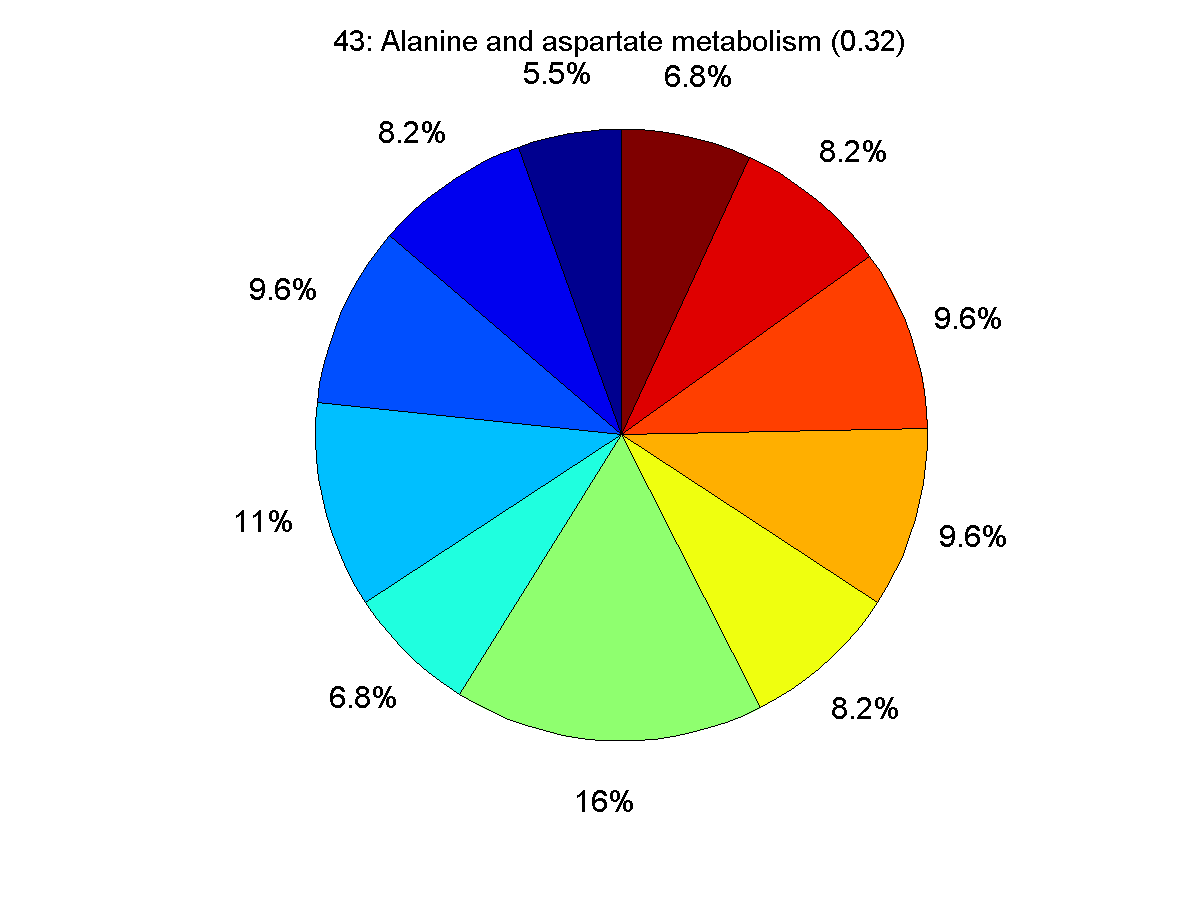

Supplement: S2 File — (ZIP) [file pone.0131875.s003.zip › MFC PieCharts/RegrEx2MFC/43Alanineandaspartatemetabolism.tif]

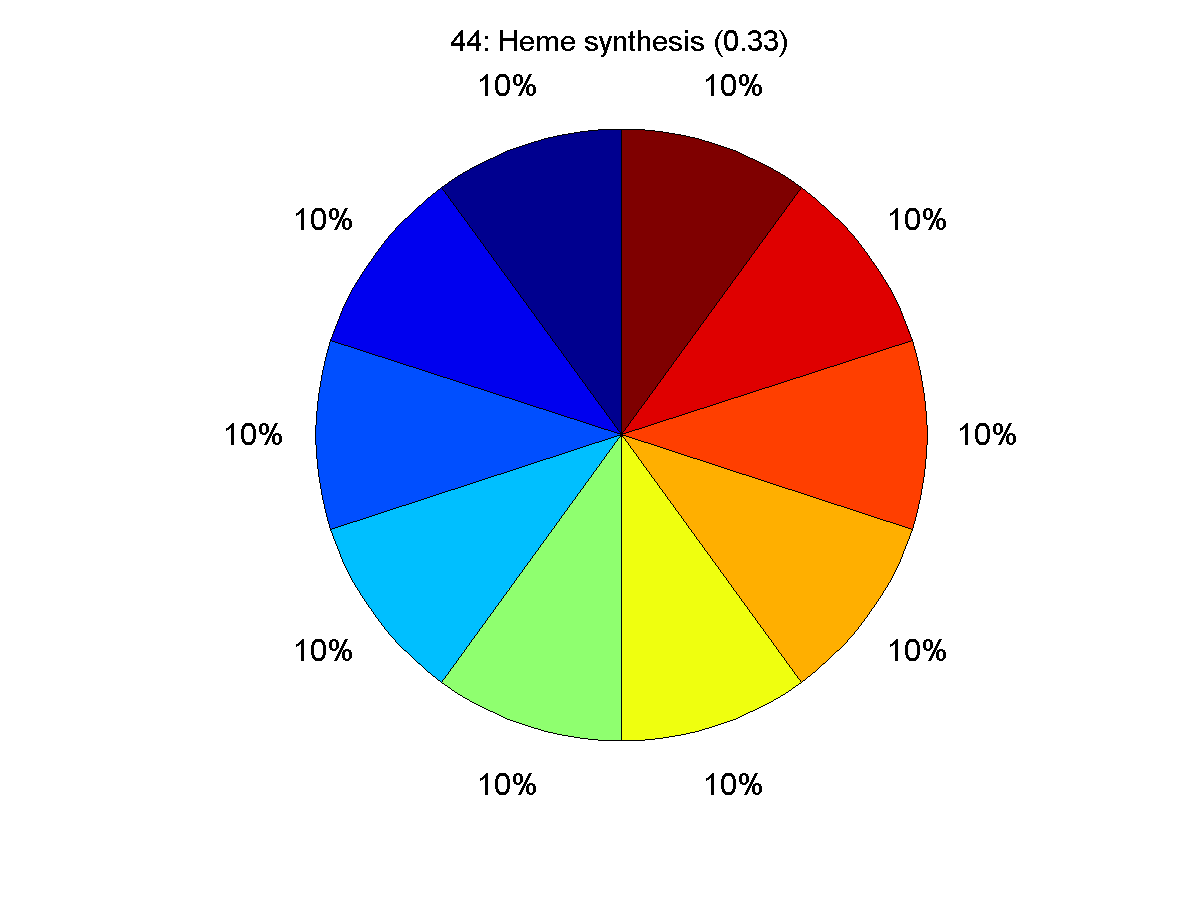

Supplement: S2 File — (ZIP) [file pone.0131875.s003.zip › MFC PieCharts/RegrEx2MFC/44Hemesynthesis.tif]

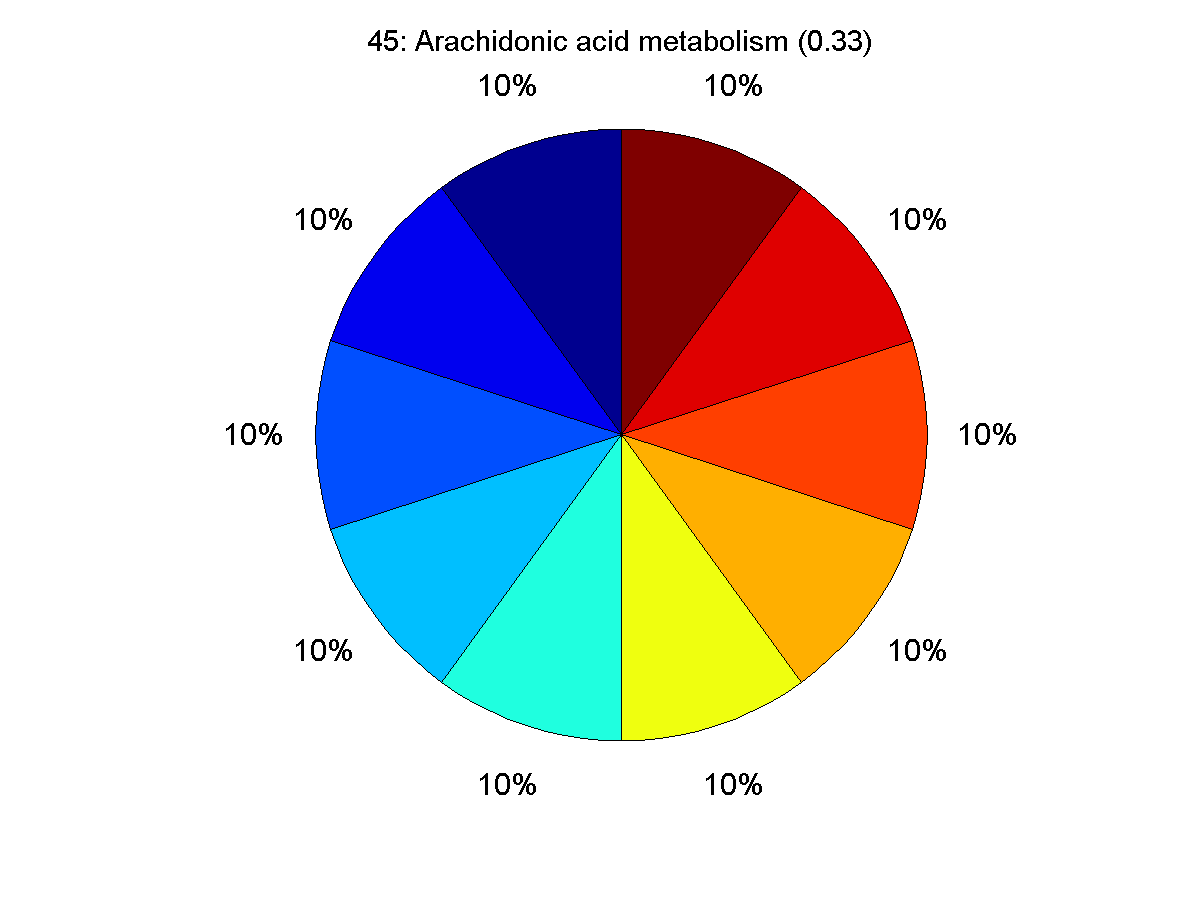

Supplement: S2 File — (ZIP) [file pone.0131875.s003.zip › MFC PieCharts/RegrEx2MFC/45Arachidonicacidmetabolism.tif]

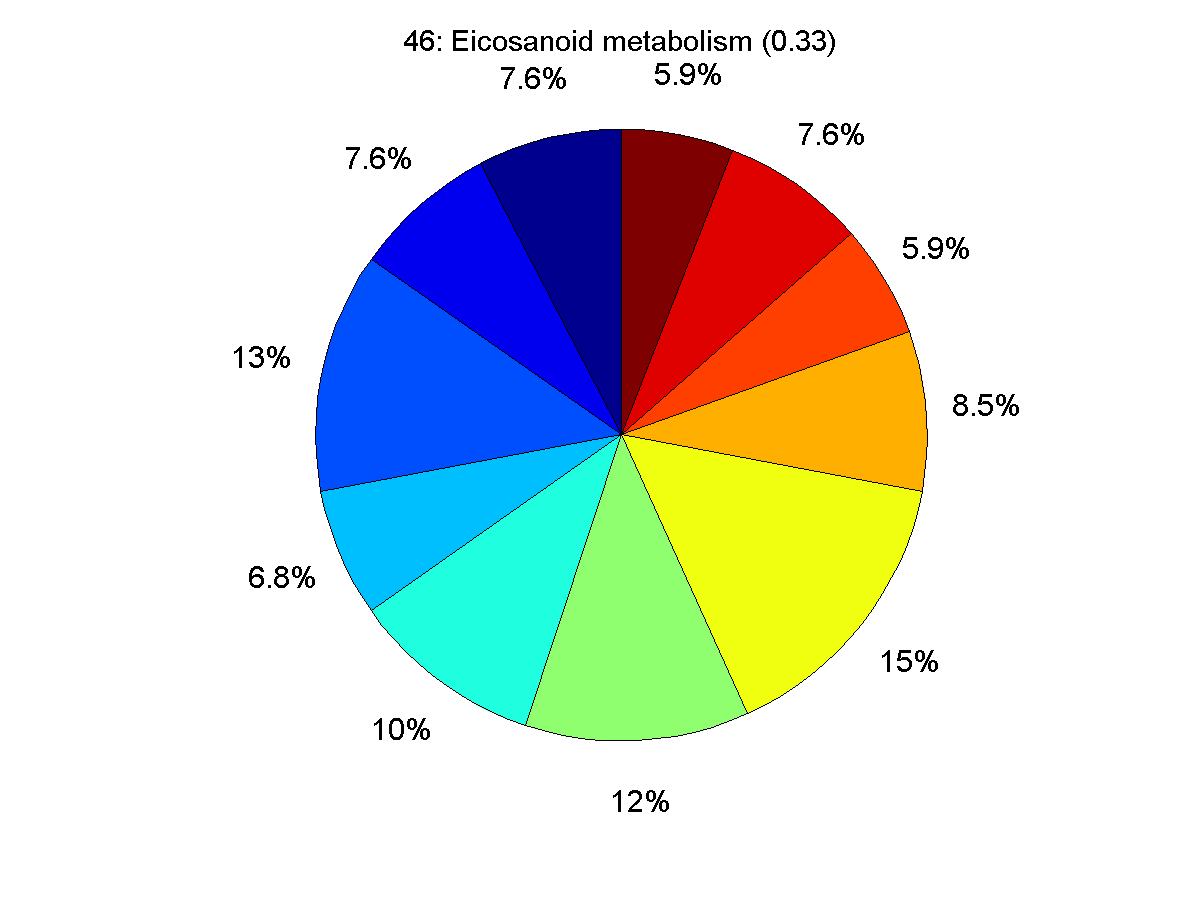

Supplement: S2 File — (ZIP) [file pone.0131875.s003.zip › MFC PieCharts/RegrEx2MFC/46Eicosanoidmetabolism.tif]

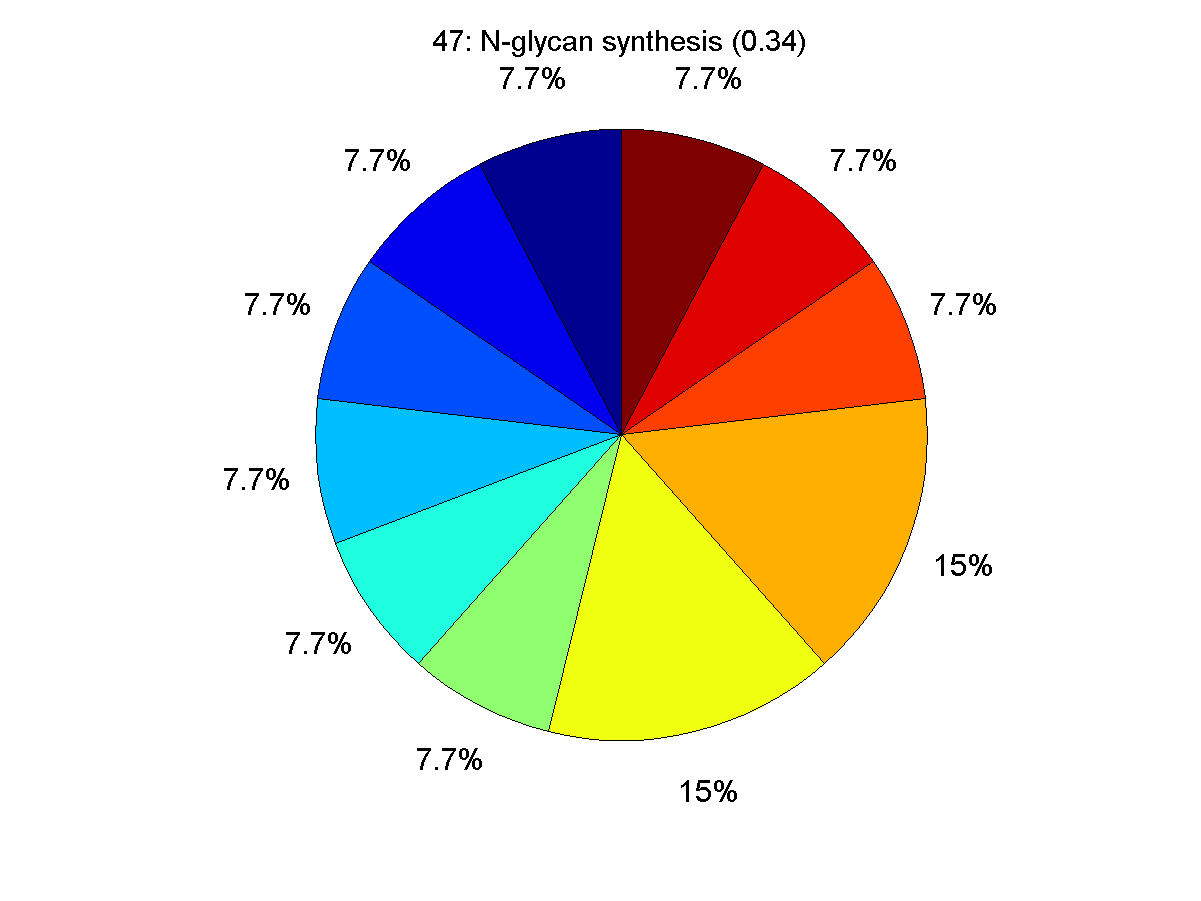

Supplement: S2 File — (ZIP) [file pone.0131875.s003.zip › MFC PieCharts/RegrEx2MFC/47N-glycansynthesis.tif]

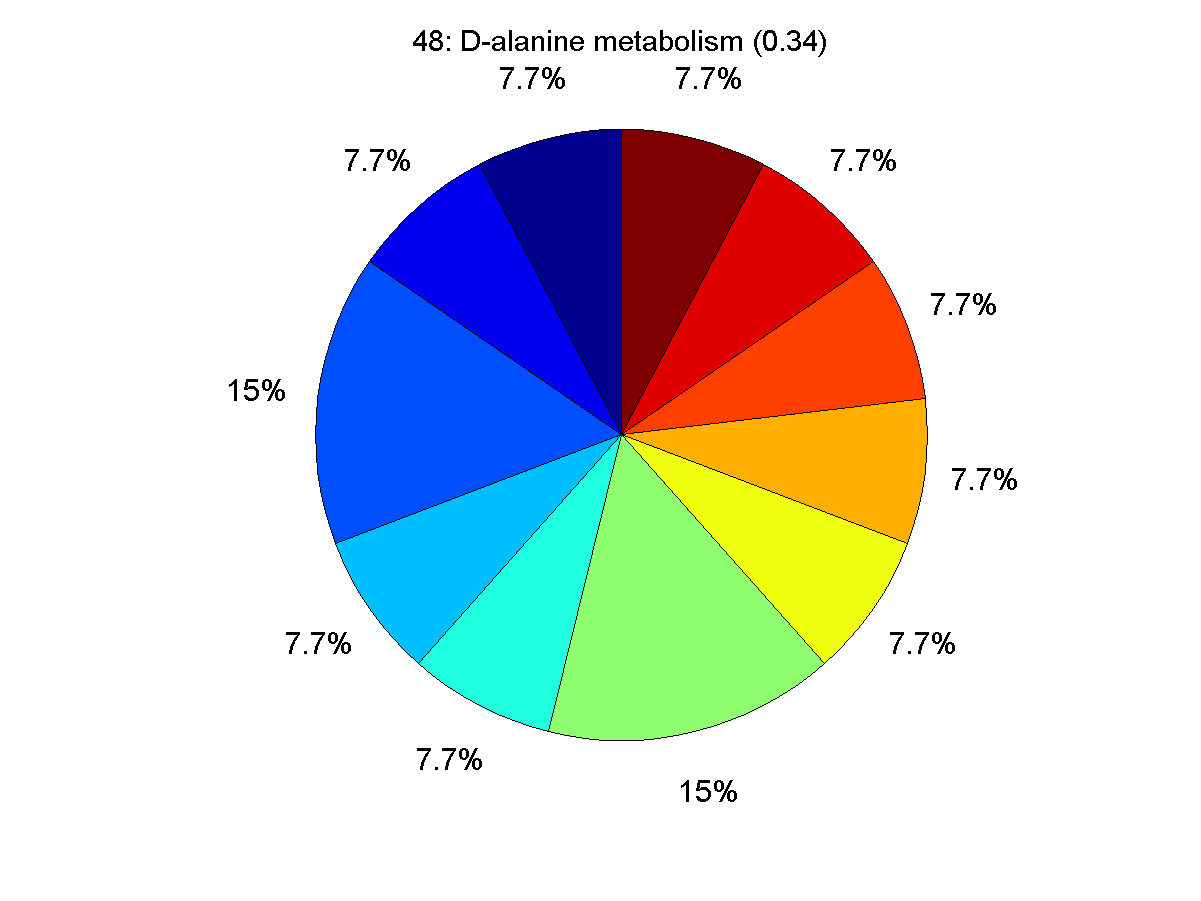

Supplement: S2 File — (ZIP) [file pone.0131875.s003.zip › MFC PieCharts/RegrEx2MFC/48D-alaninemetabolism.tif]

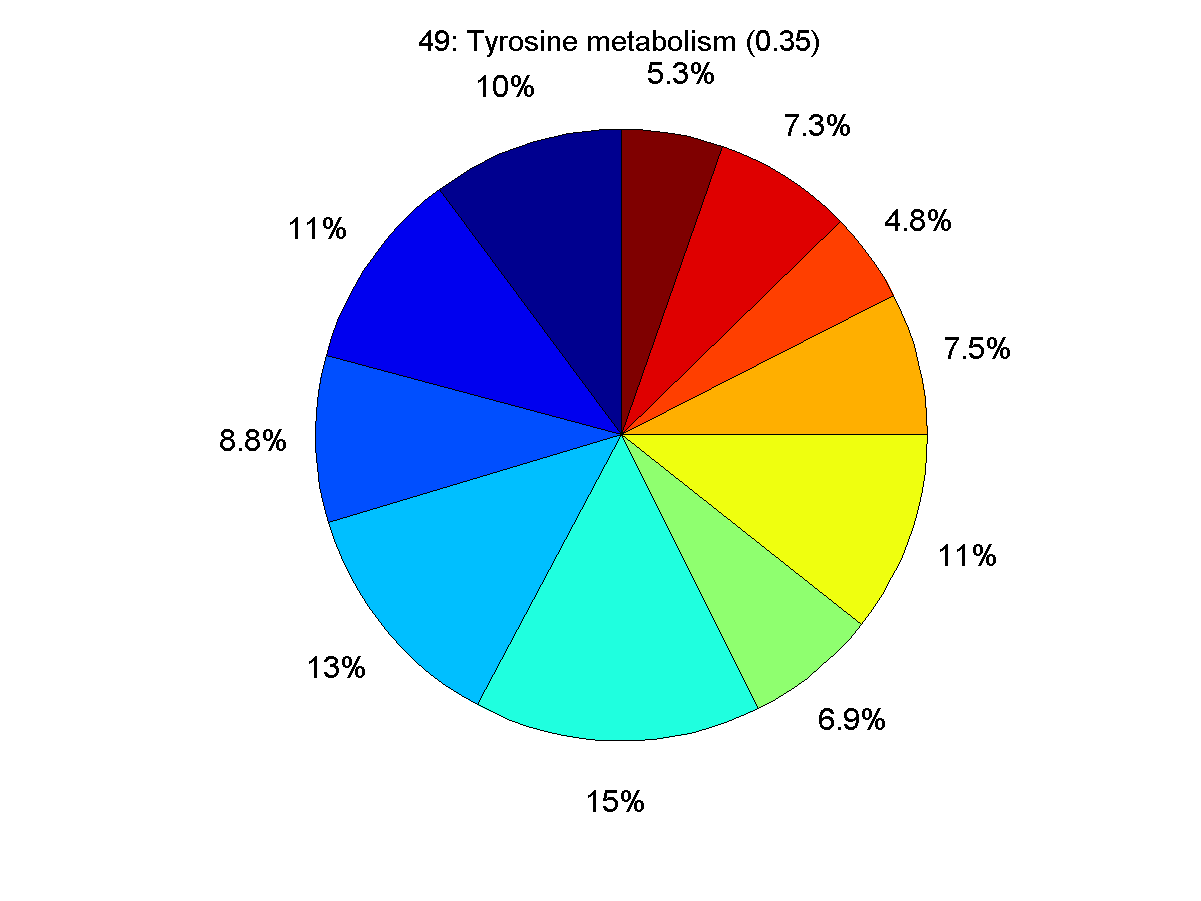

Supplement: S2 File — (ZIP) [file pone.0131875.s003.zip › MFC PieCharts/RegrEx2MFC/49Tyrosinemetabolism.tif]

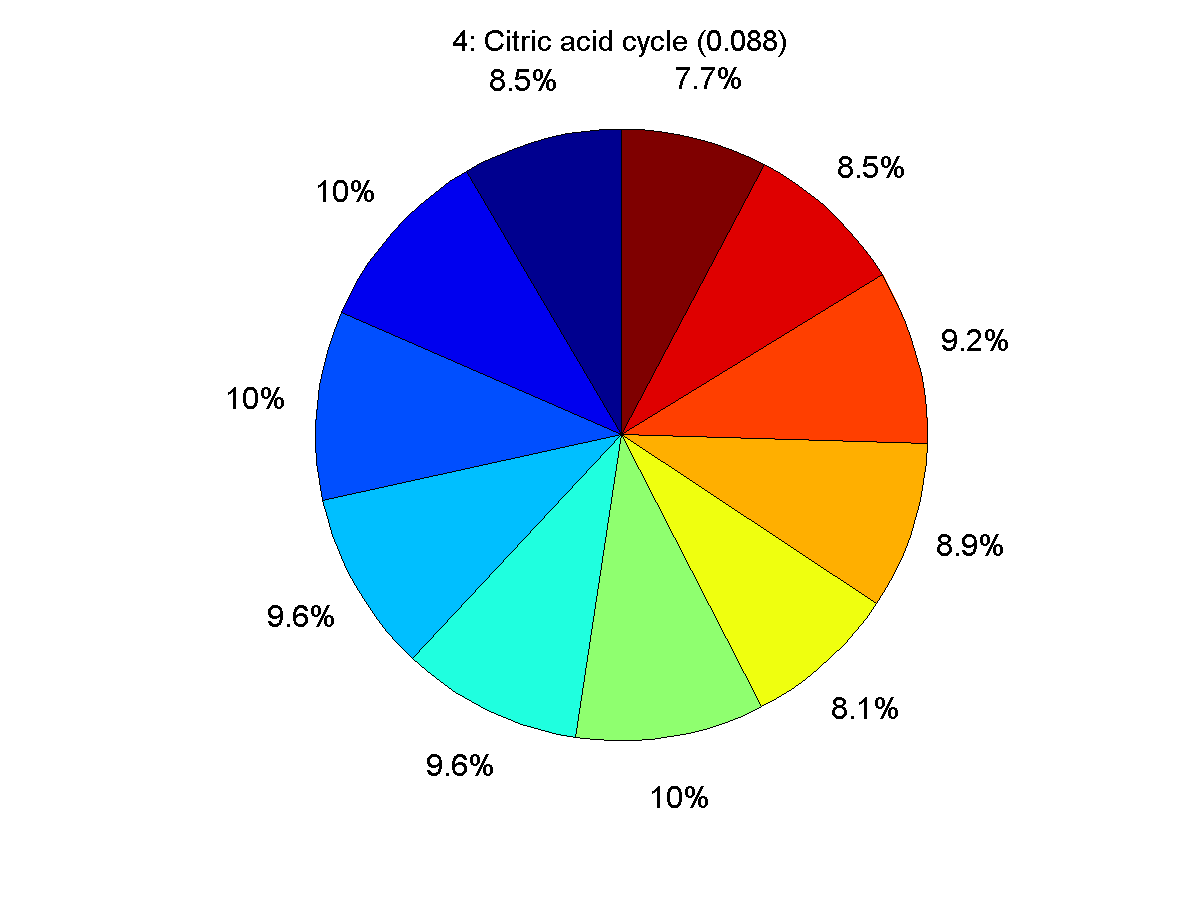

Supplement: S2 File — (ZIP) [file pone.0131875.s003.zip › MFC PieCharts/RegrEx2MFC/4Citricacidcycle.tif]

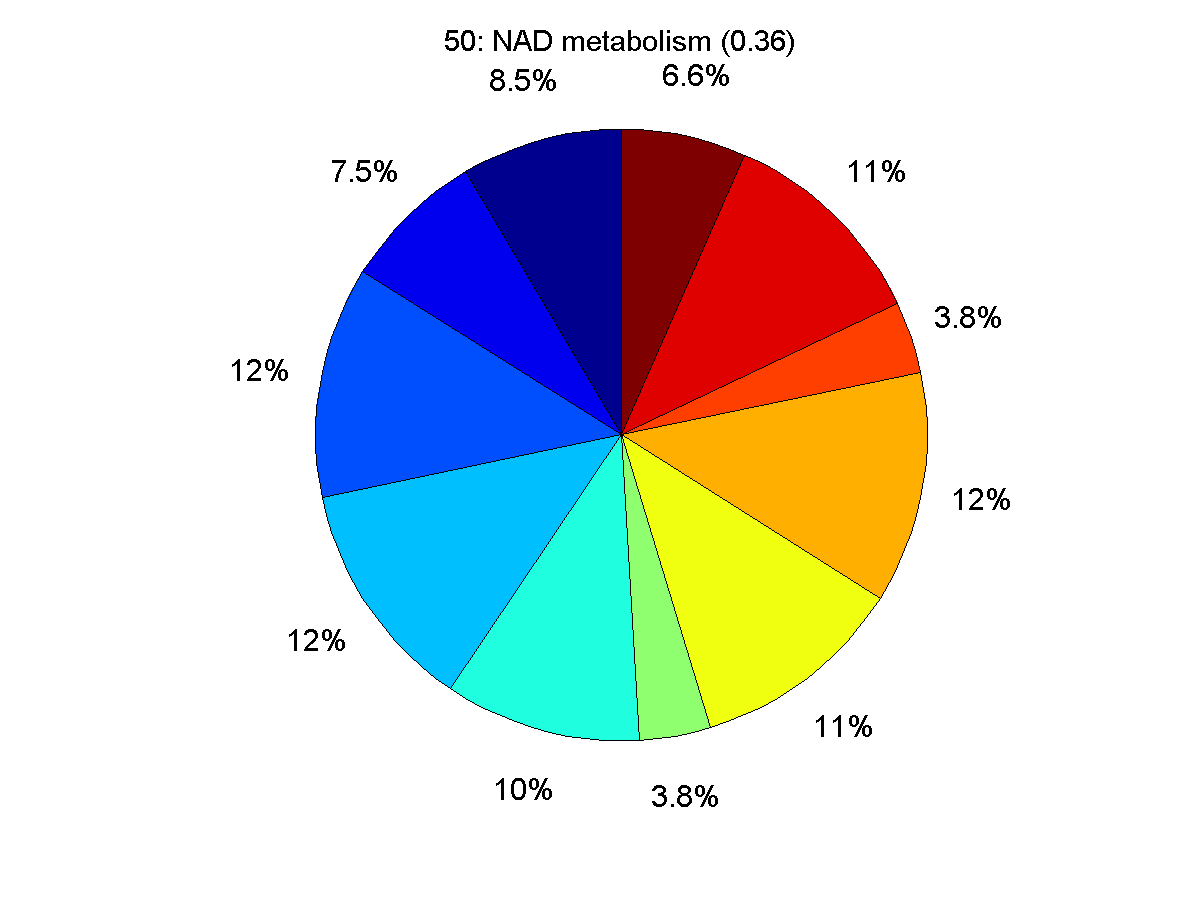

Supplement: S2 File — (ZIP) [file pone.0131875.s003.zip › MFC PieCharts/RegrEx2MFC/50NADmetabolism.tif]

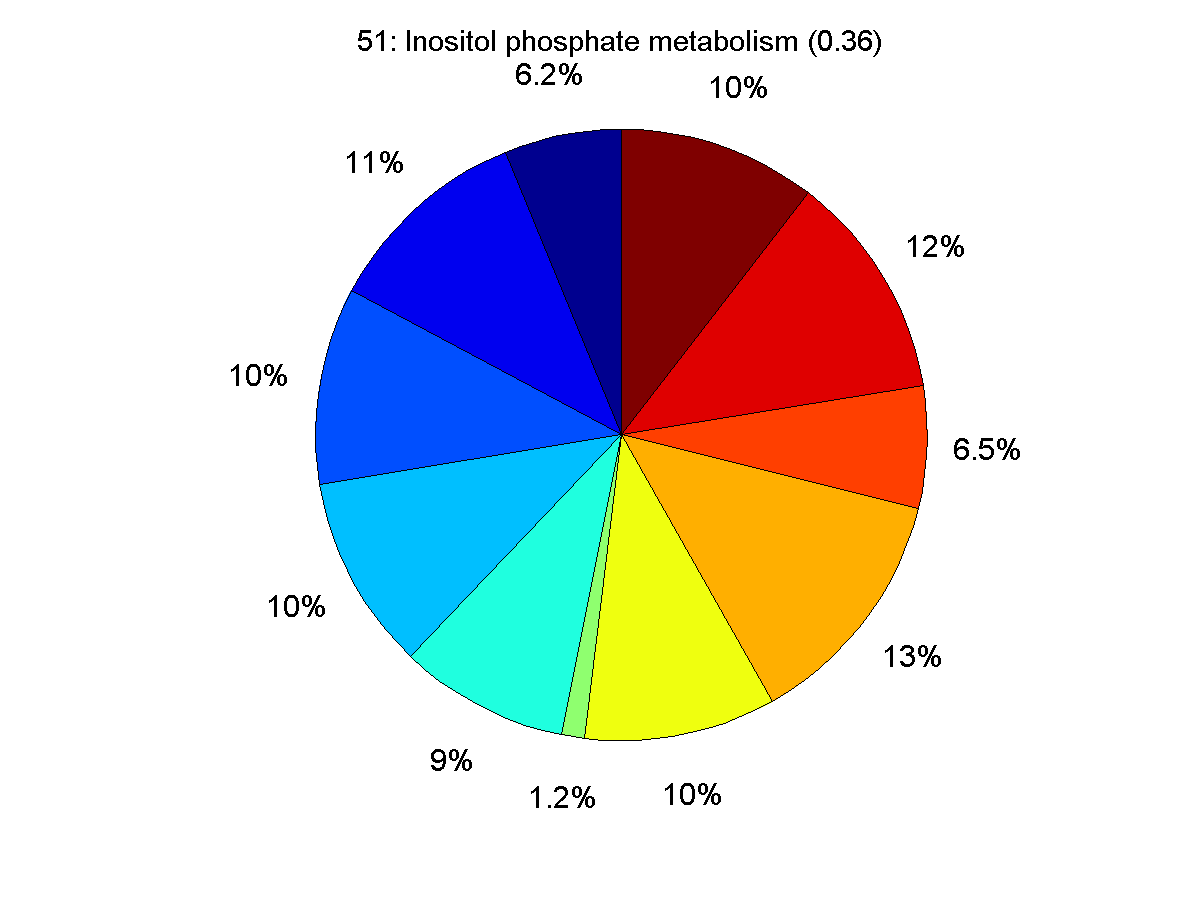

Supplement: S2 File — (ZIP) [file pone.0131875.s003.zip › MFC PieCharts/RegrEx2MFC/51Inositolphosphatemetabolism.tif]

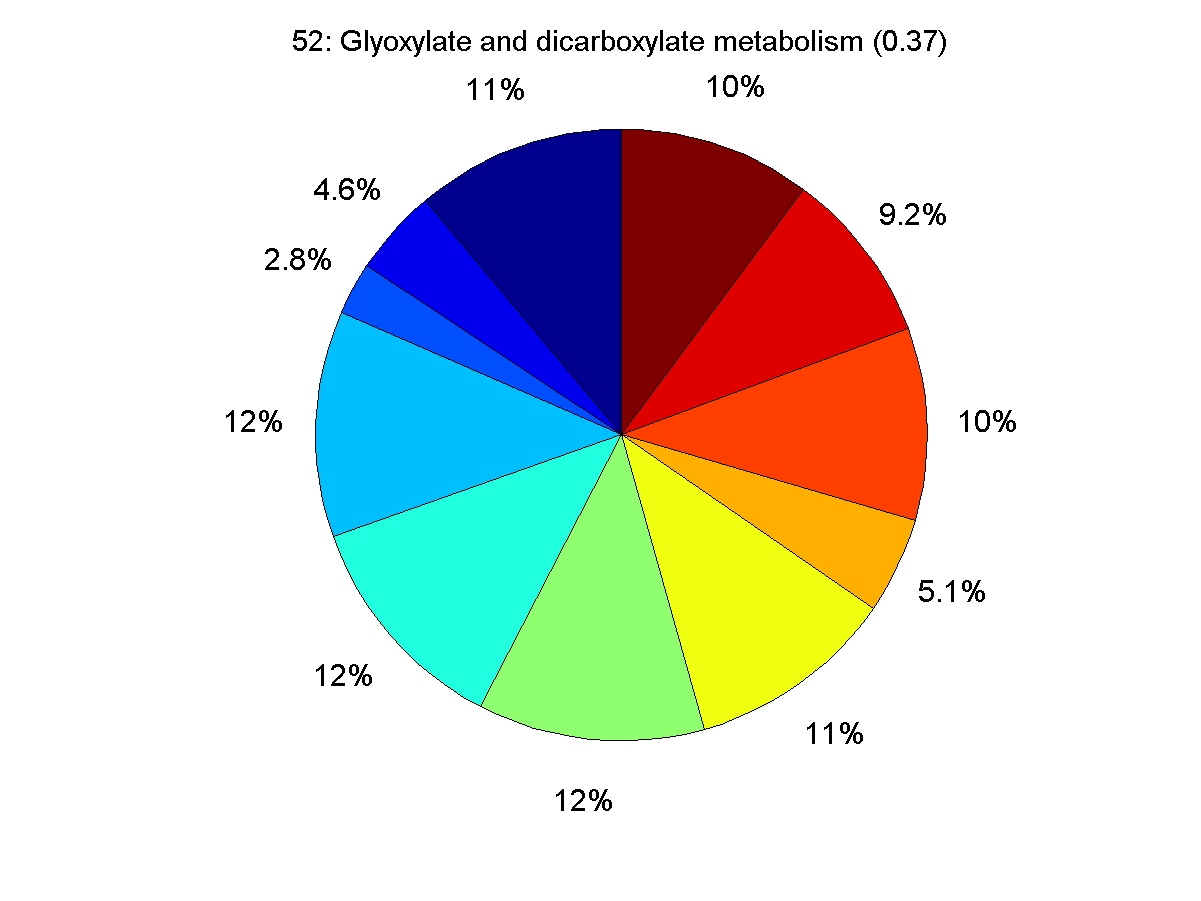

Supplement: S2 File — (ZIP) [file pone.0131875.s003.zip › MFC PieCharts/RegrEx2MFC/52Glyoxylateanddicarboxylatemetabolism.tif]

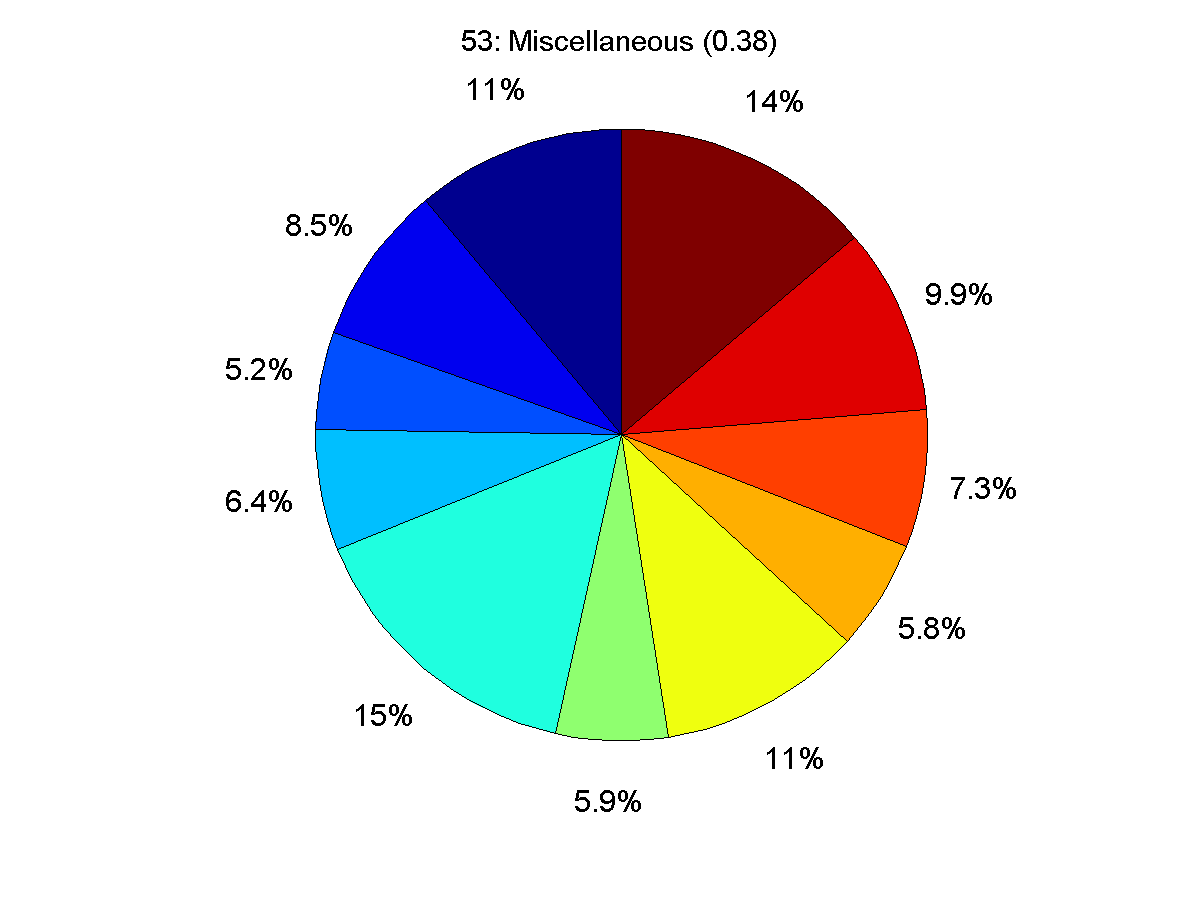

Supplement: S2 File — (ZIP) [file pone.0131875.s003.zip › MFC PieCharts/RegrEx2MFC/53Miscellaneous.tif]

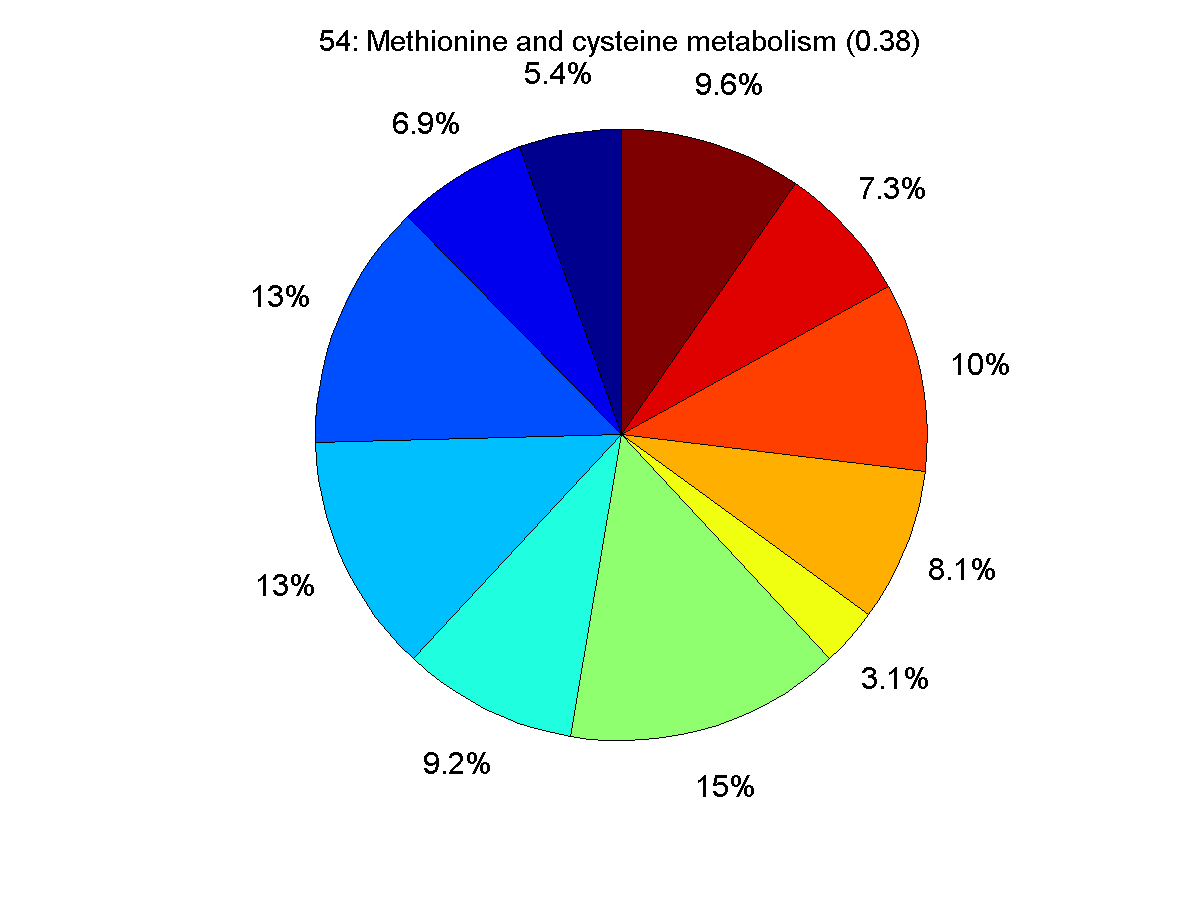

Supplement: S2 File — (ZIP) [file pone.0131875.s003.zip › MFC PieCharts/RegrEx2MFC/54Methionineandcysteinemetabolism.tif]

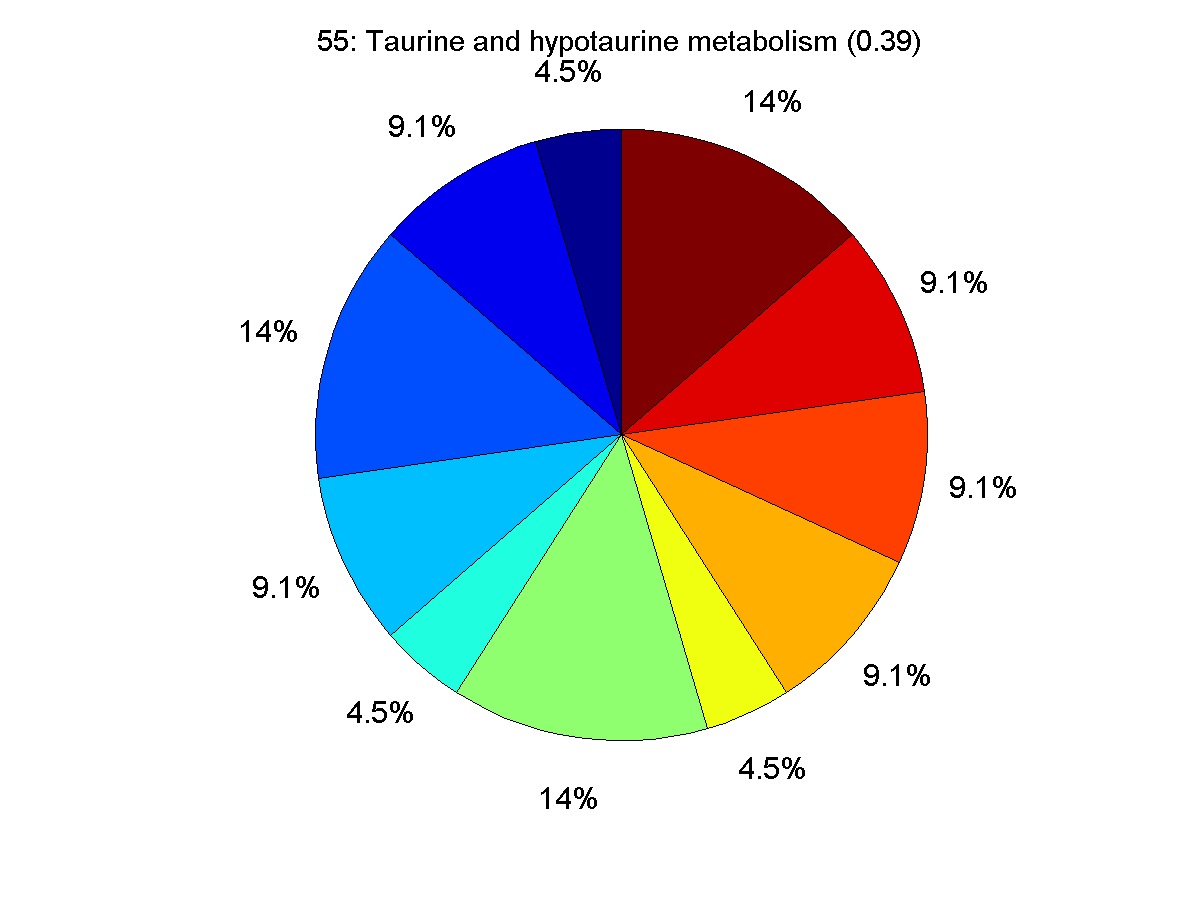

Supplement: S2 File — (ZIP) [file pone.0131875.s003.zip › MFC PieCharts/RegrEx2MFC/55Taurineandhypotaurinemetabolism.tif]

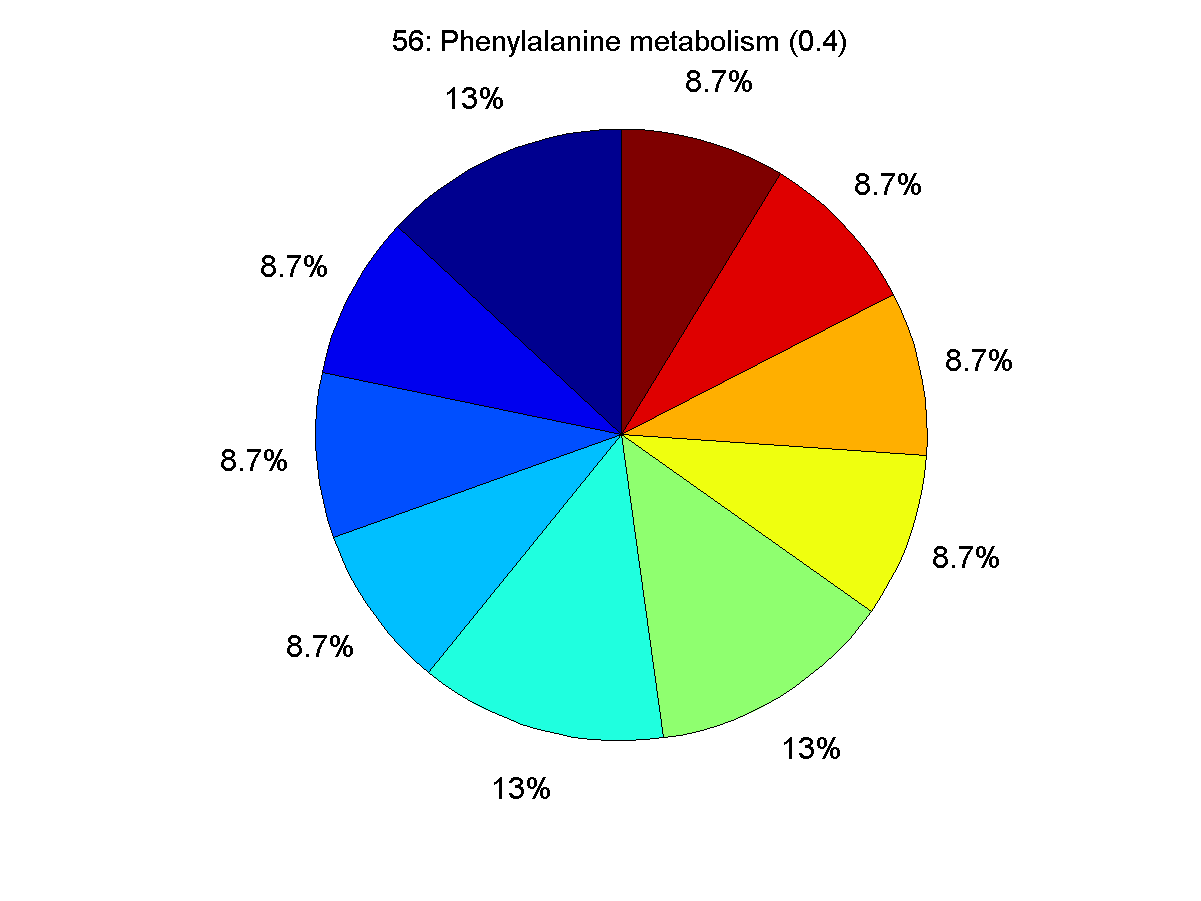

Supplement: S2 File — (ZIP) [file pone.0131875.s003.zip › MFC PieCharts/RegrEx2MFC/56Phenylalaninemetabolism.tif]

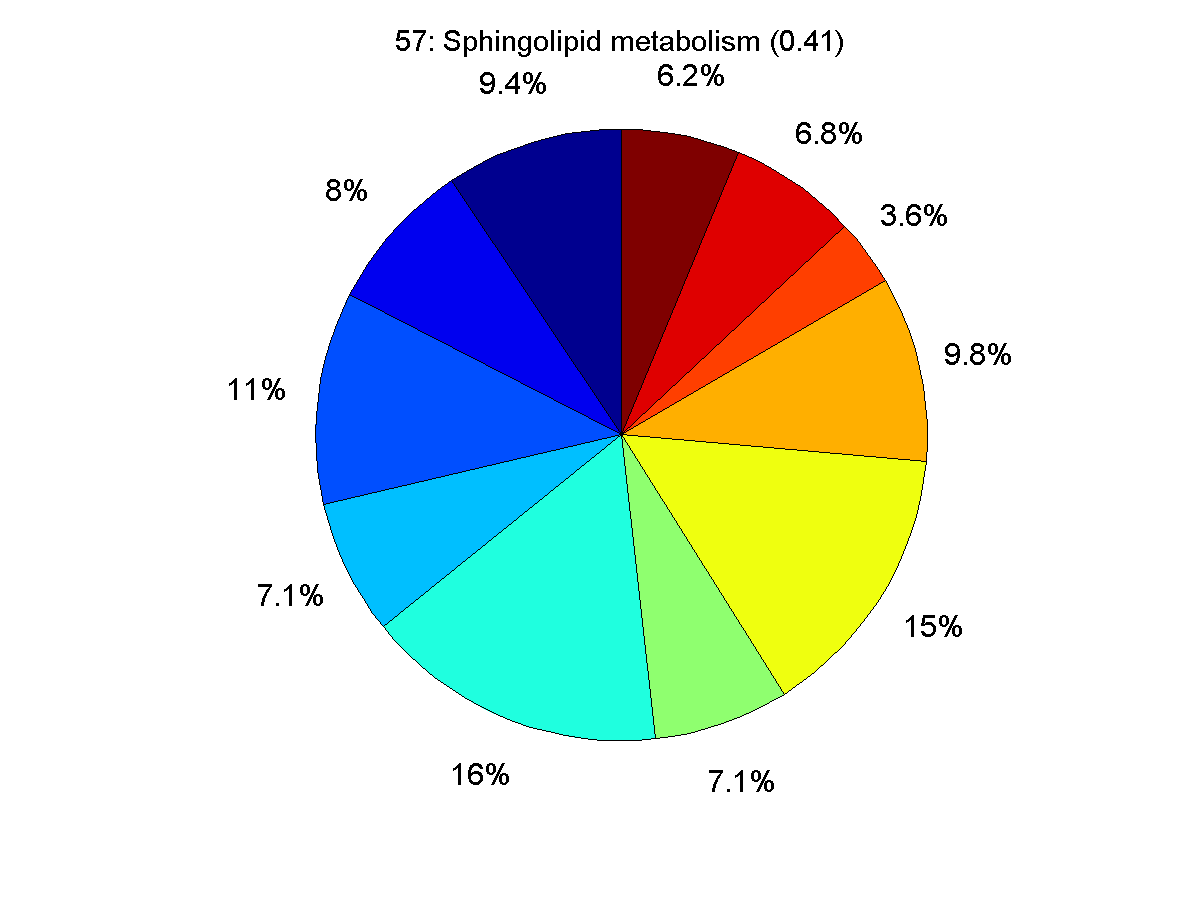

Supplement: S2 File — (ZIP) [file pone.0131875.s003.zip › MFC PieCharts/RegrEx2MFC/57Sphingolipidmetabolism.tif]

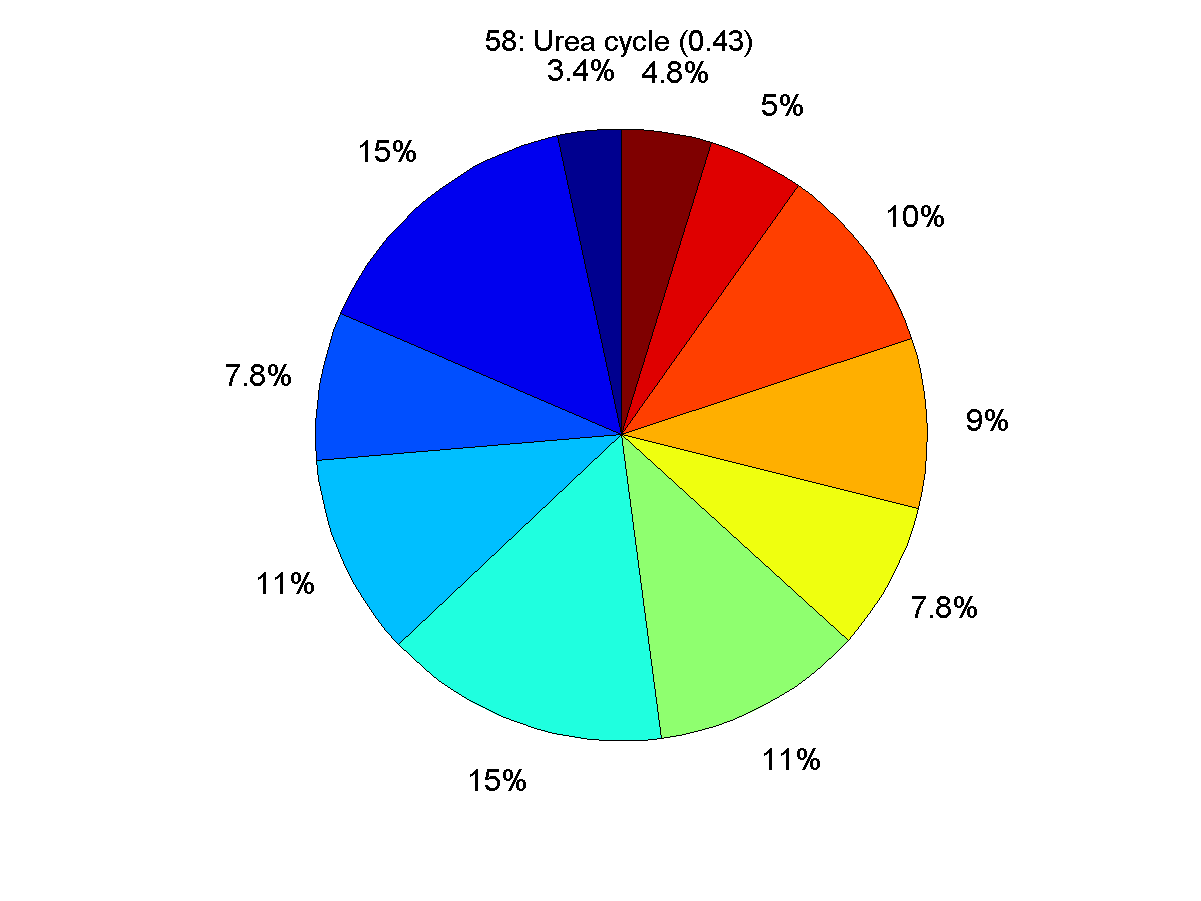

Supplement: S2 File — (ZIP) [file pone.0131875.s003.zip › MFC PieCharts/RegrEx2MFC/58Ureacycle.tif]

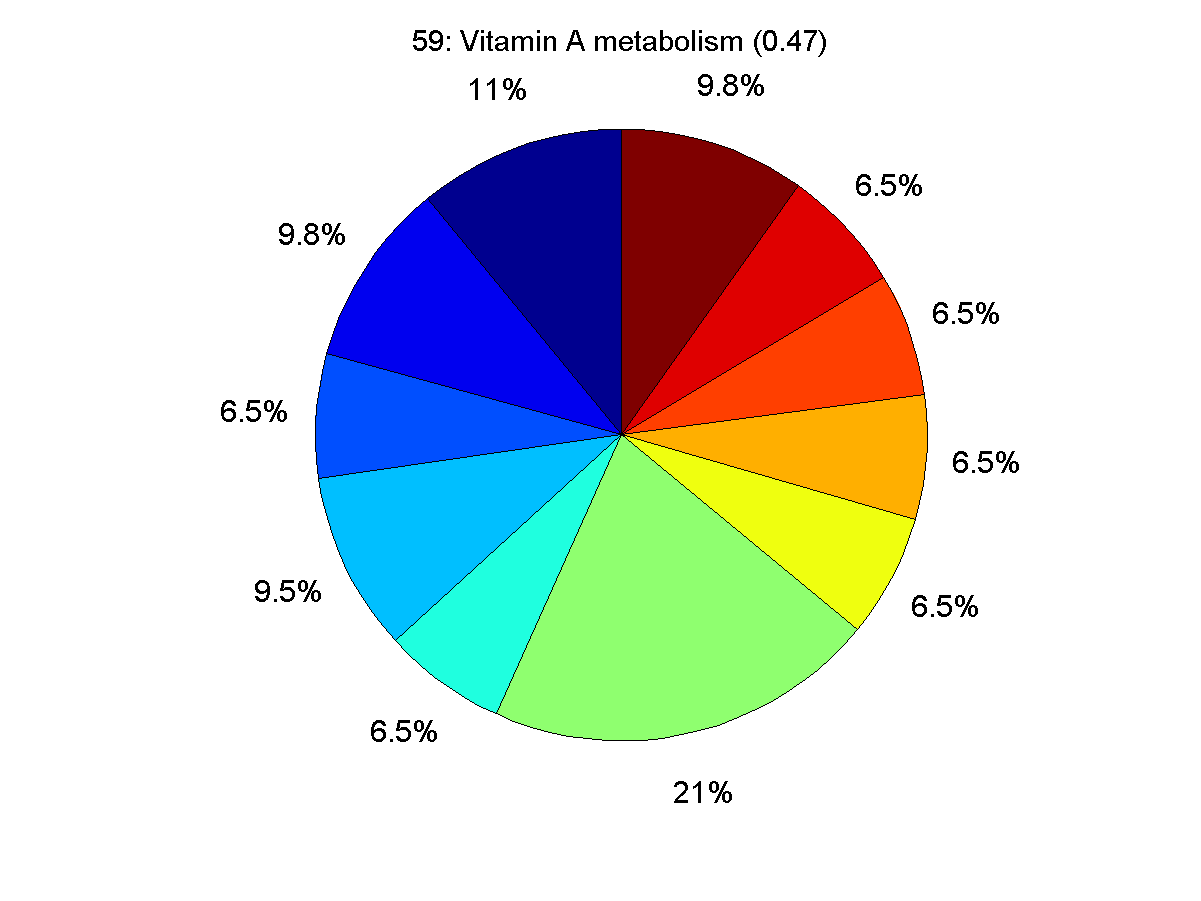

Supplement: S2 File — (ZIP) [file pone.0131875.s003.zip › MFC PieCharts/RegrEx2MFC/59VitaminAmetabolism.tif]

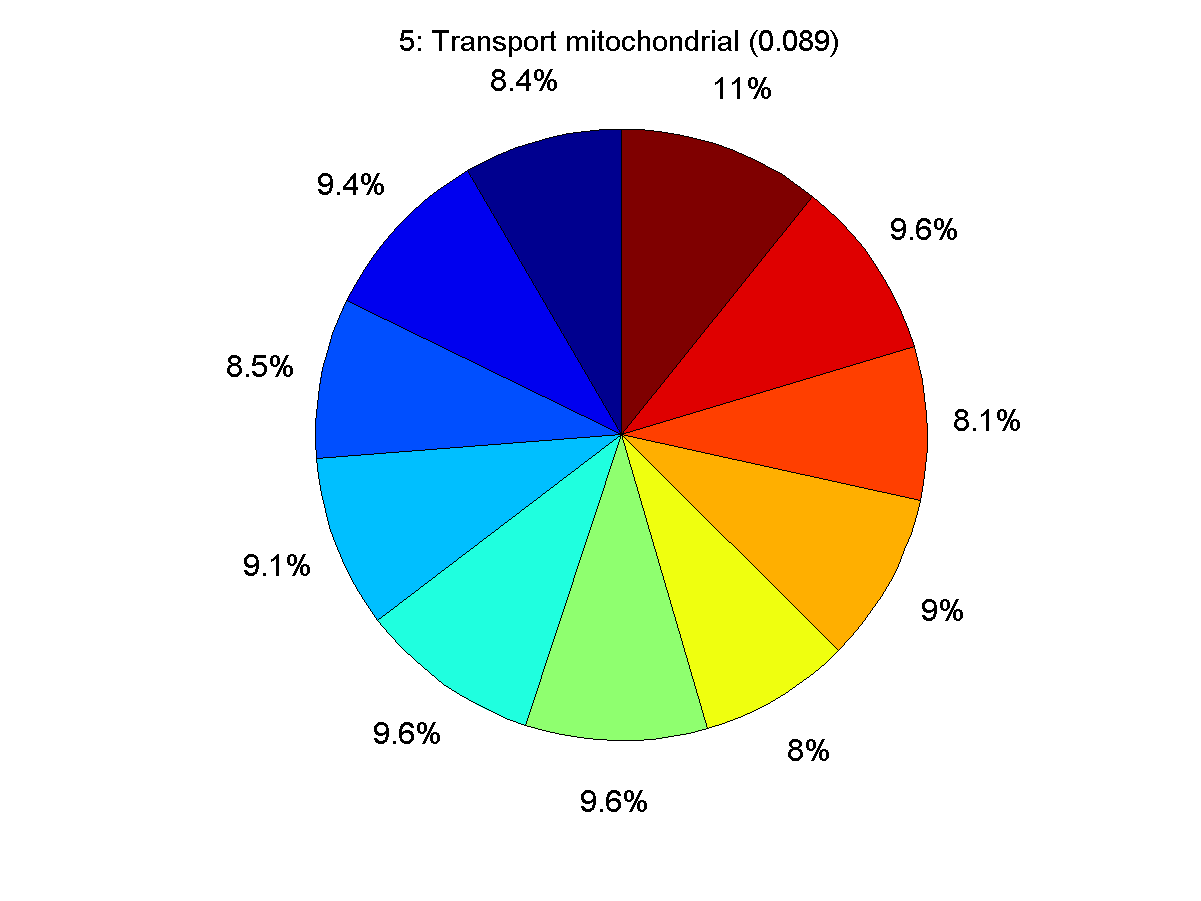

Supplement: S2 File — (ZIP) [file pone.0131875.s003.zip › MFC PieCharts/RegrEx2MFC/5Transportmitochondrial.tif]

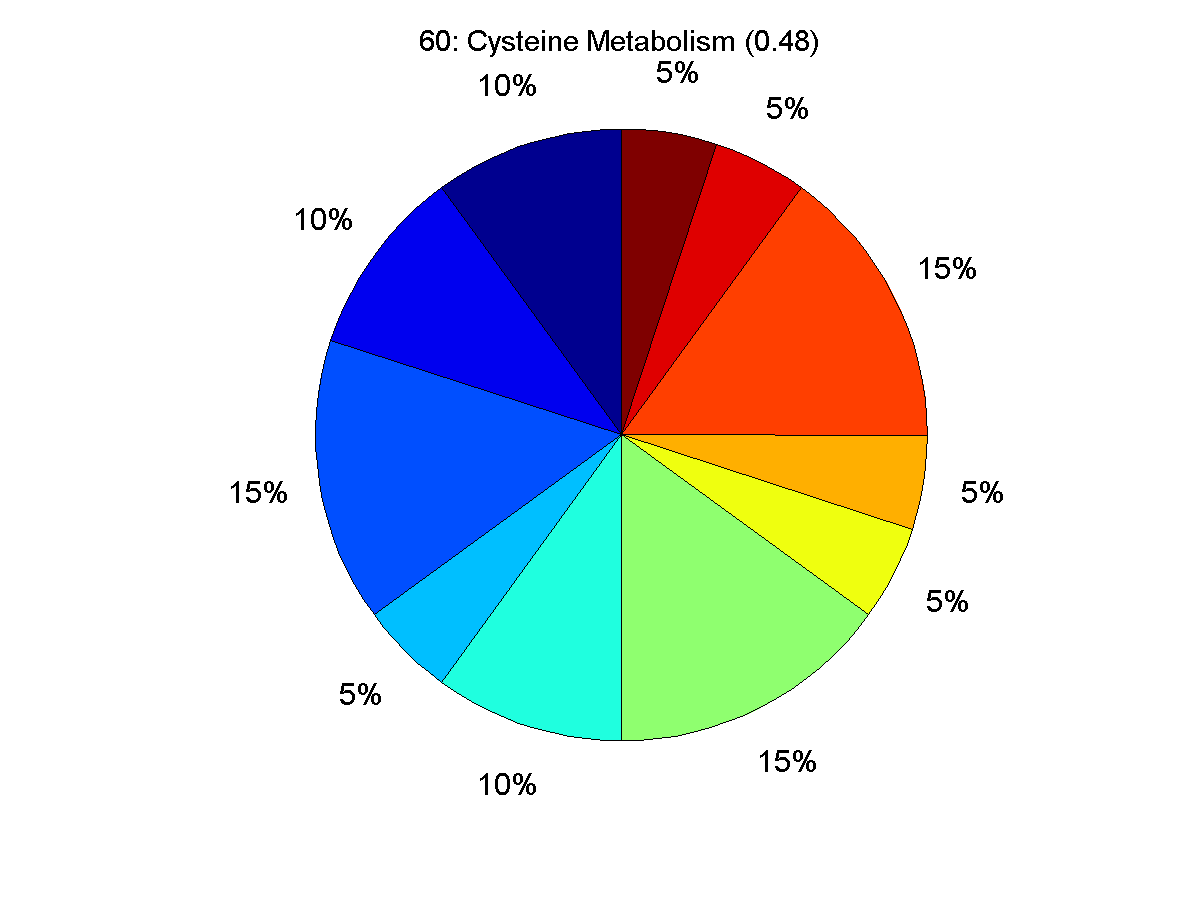

Supplement: S2 File — (ZIP) [file pone.0131875.s003.zip › MFC PieCharts/RegrEx2MFC/60CysteineMetabolism.tif]

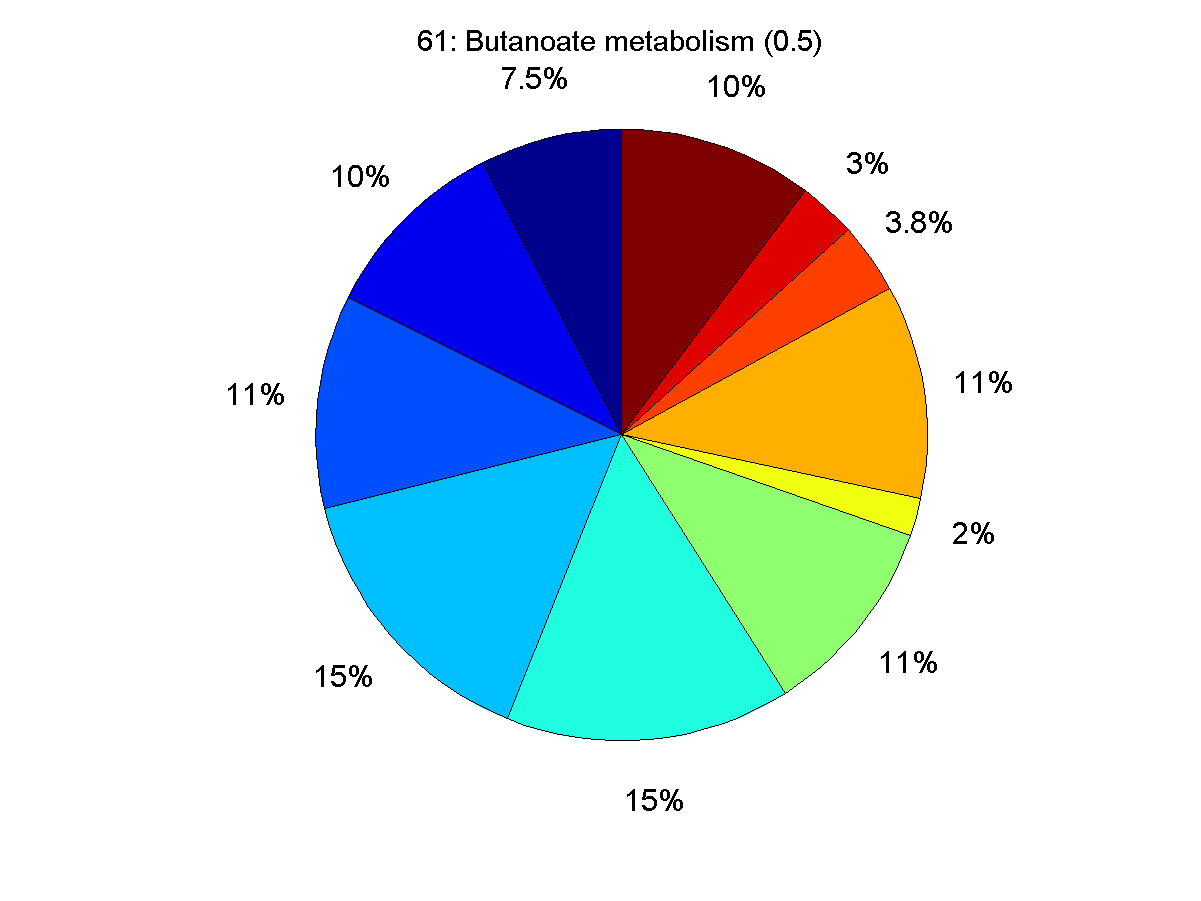

Supplement: S2 File — (ZIP) [file pone.0131875.s003.zip › MFC PieCharts/RegrEx2MFC/61Butanoatemetabolism.tif]

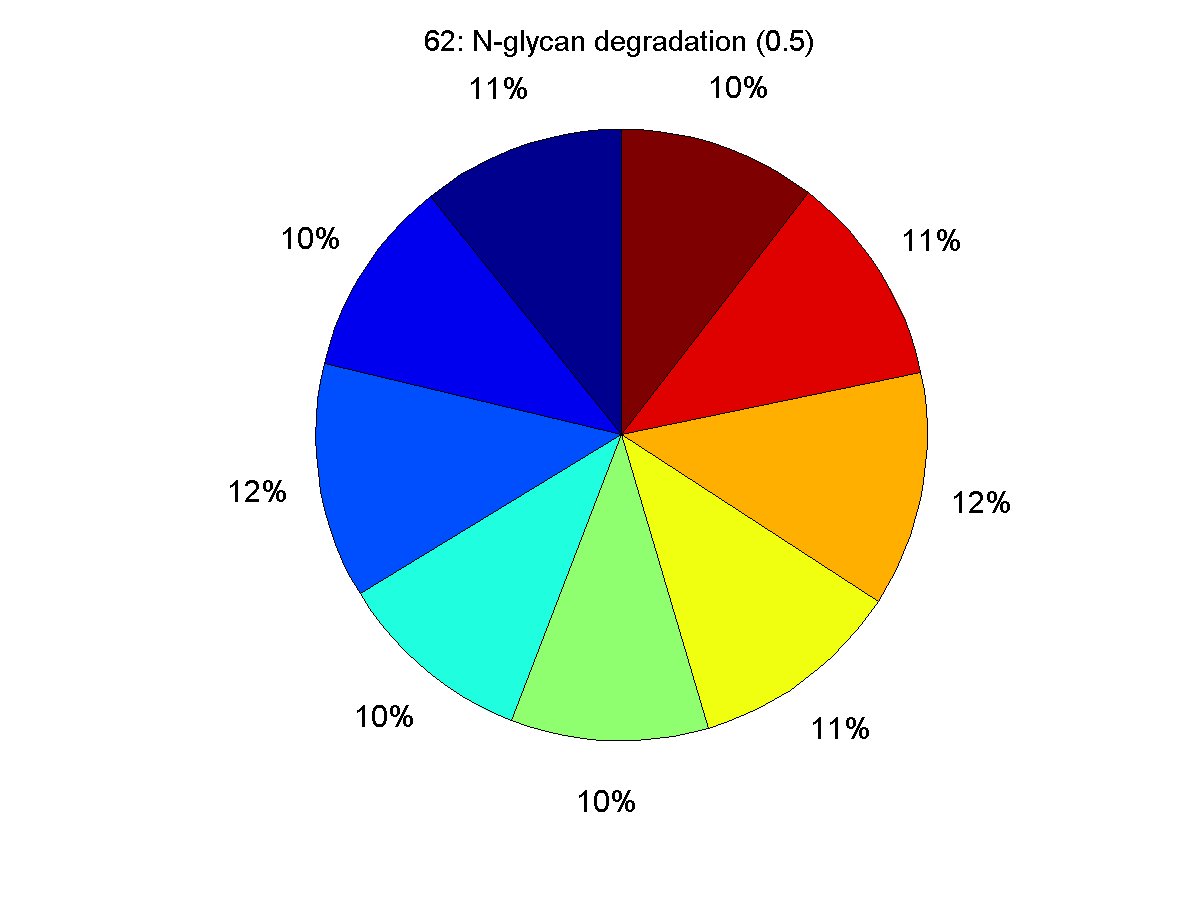

Supplement: S2 File — (ZIP) [file pone.0131875.s003.zip › MFC PieCharts/RegrEx2MFC/62N-glycandegradation.tif]

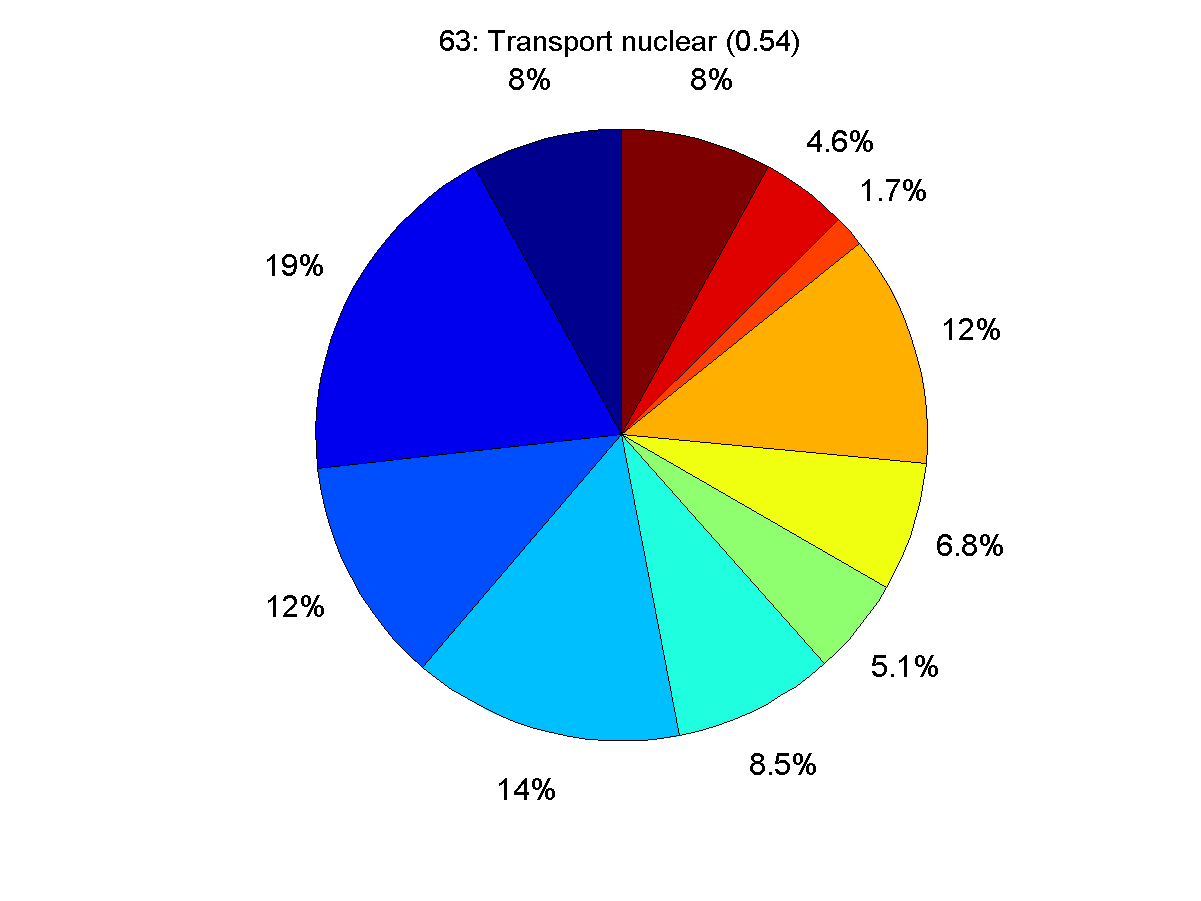

Supplement: S2 File — (ZIP) [file pone.0131875.s003.zip › MFC PieCharts/RegrEx2MFC/63Transportnuclear.tif]

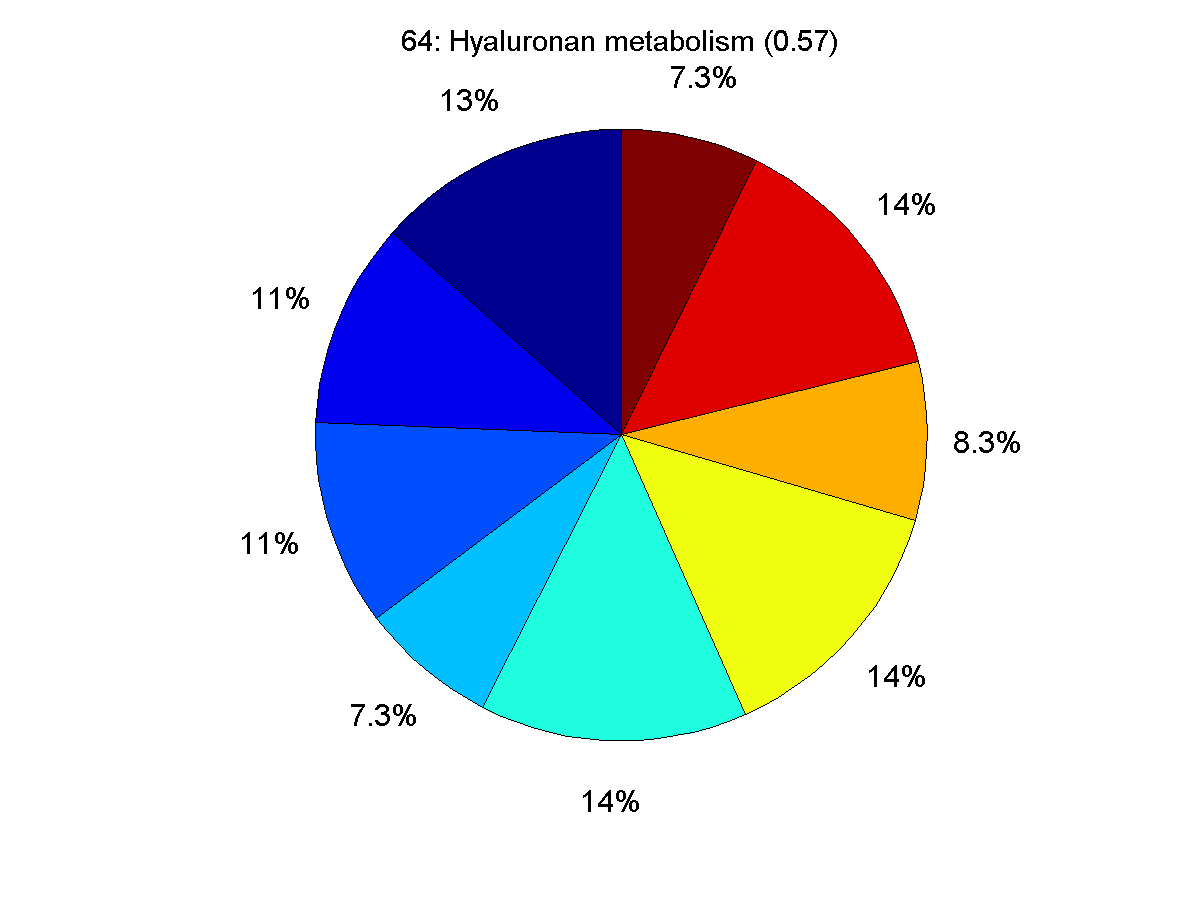

Supplement: S2 File — (ZIP) [file pone.0131875.s003.zip › MFC PieCharts/RegrEx2MFC/64Hyaluronanmetabolism.tif]

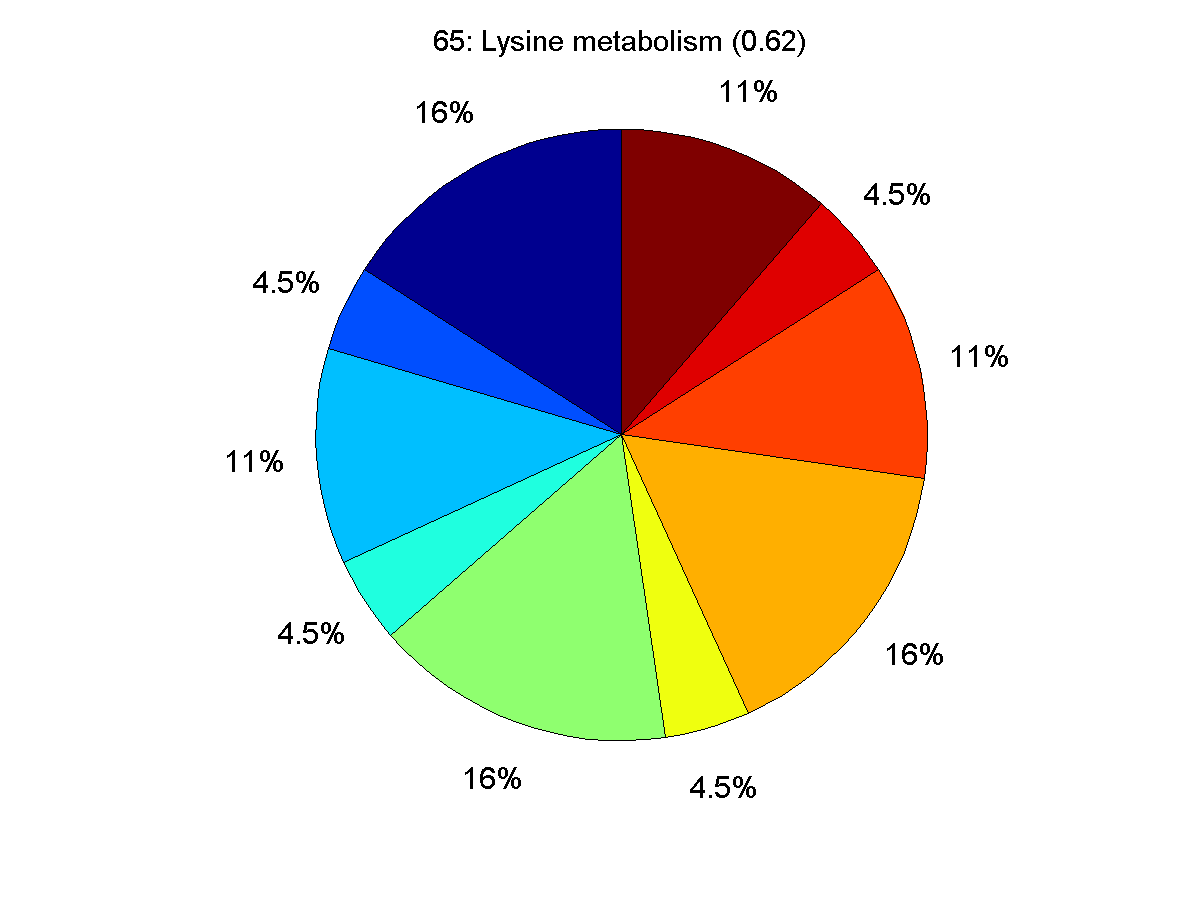

Supplement: S2 File — (ZIP) [file pone.0131875.s003.zip › MFC PieCharts/RegrEx2MFC/65Lysinemetabolism.tif]

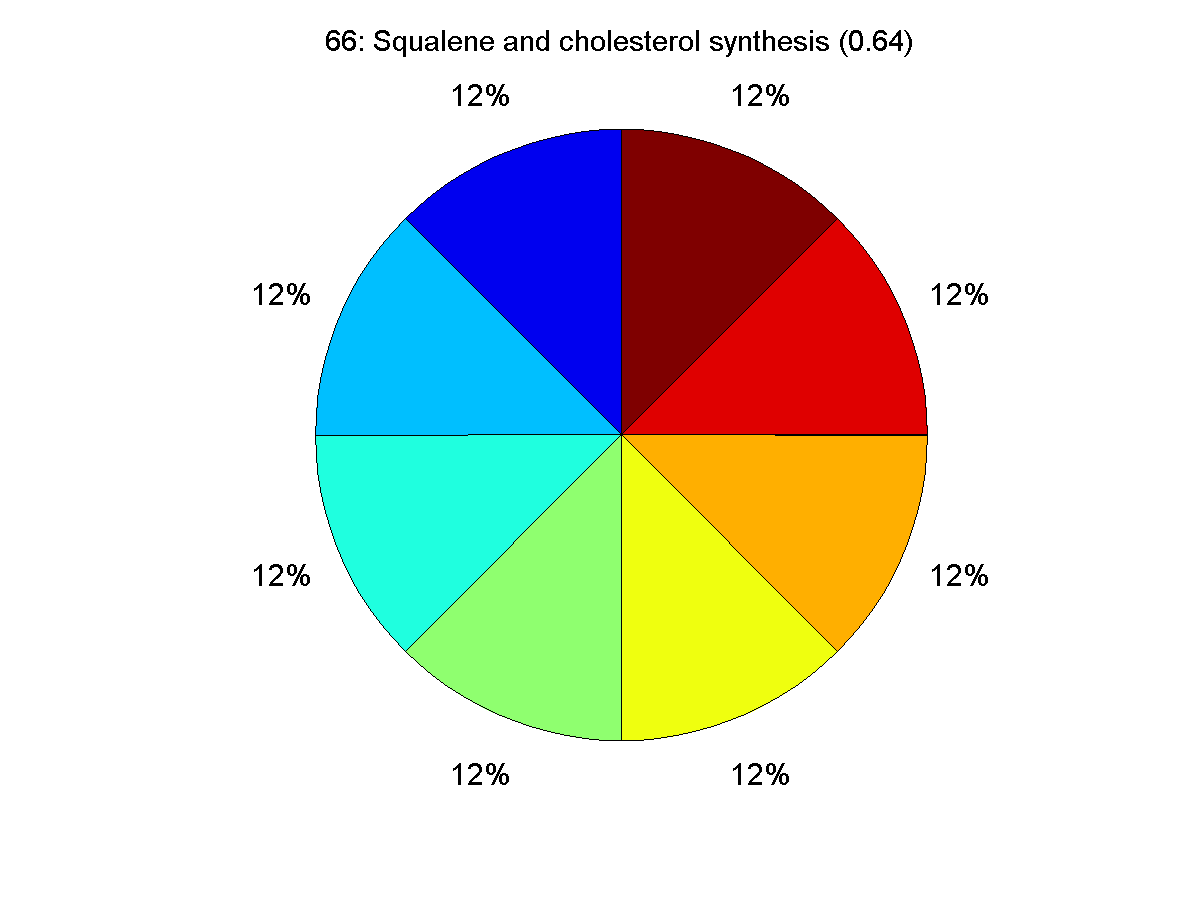

Supplement: S2 File — (ZIP) [file pone.0131875.s003.zip › MFC PieCharts/RegrEx2MFC/66Squaleneandcholesterolsynthesis.tif]

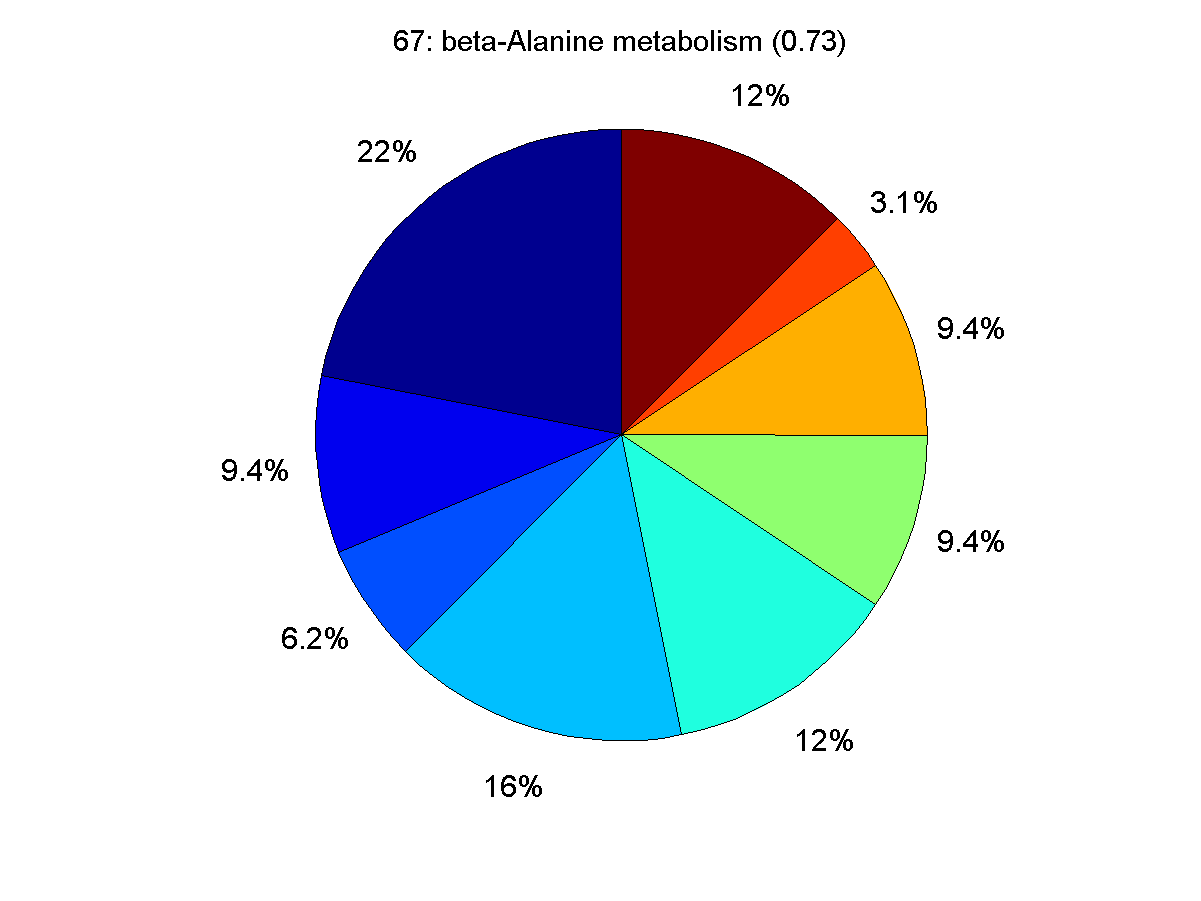

Supplement: S2 File — (ZIP) [file pone.0131875.s003.zip › MFC PieCharts/RegrEx2MFC/67beta-Alaninemetabolism.tif]

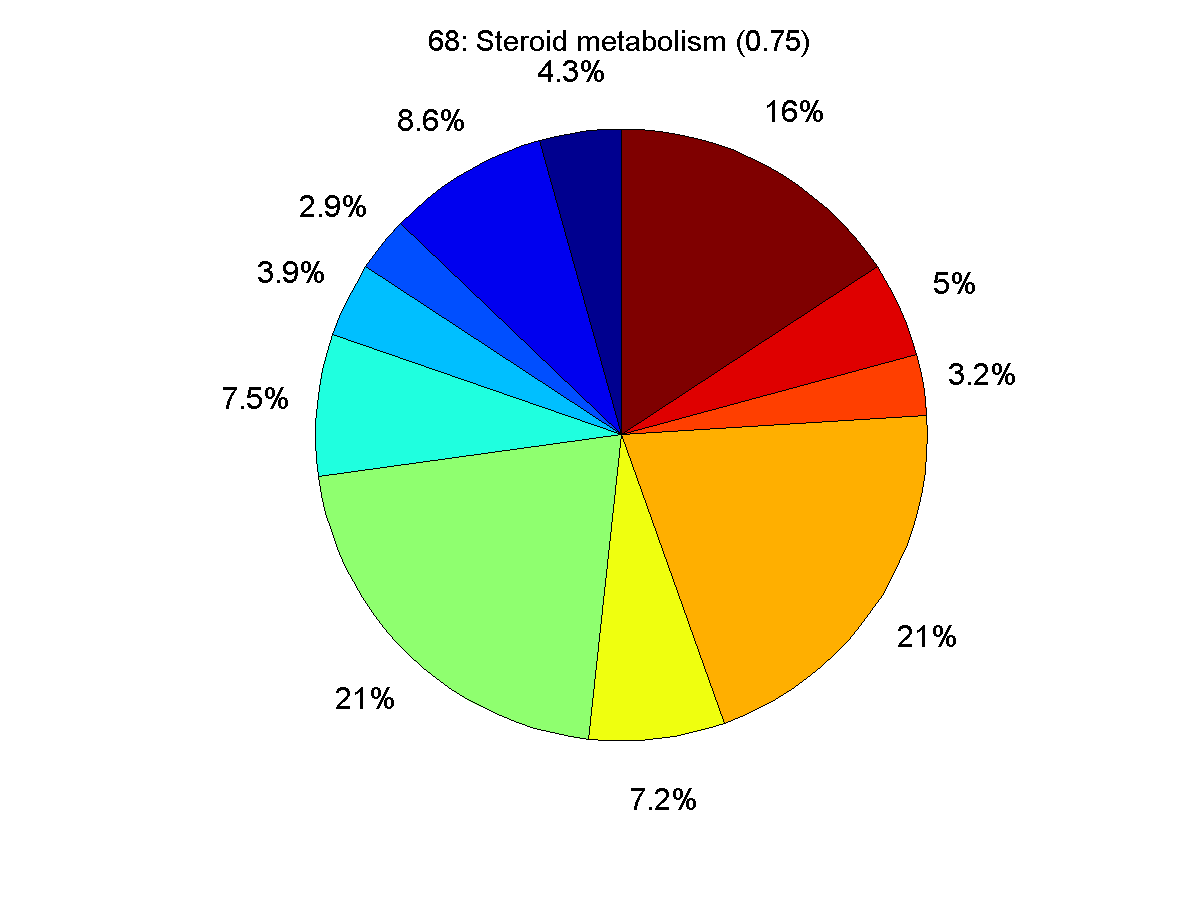

Supplement: S2 File — (ZIP) [file pone.0131875.s003.zip › MFC PieCharts/RegrEx2MFC/68Steroidmetabolism.tif]

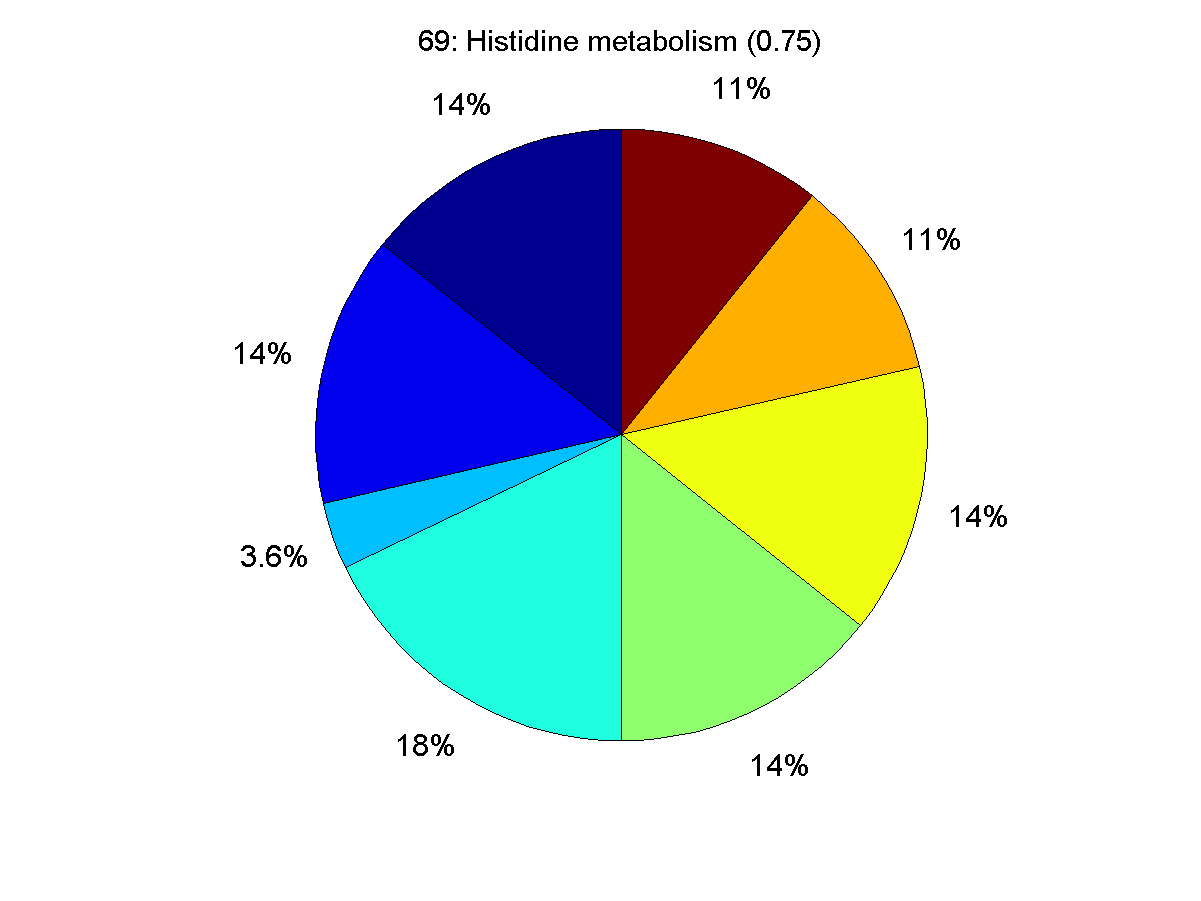

Supplement: S2 File — (ZIP) [file pone.0131875.s003.zip › MFC PieCharts/RegrEx2MFC/69Histidinemetabolism.tif]

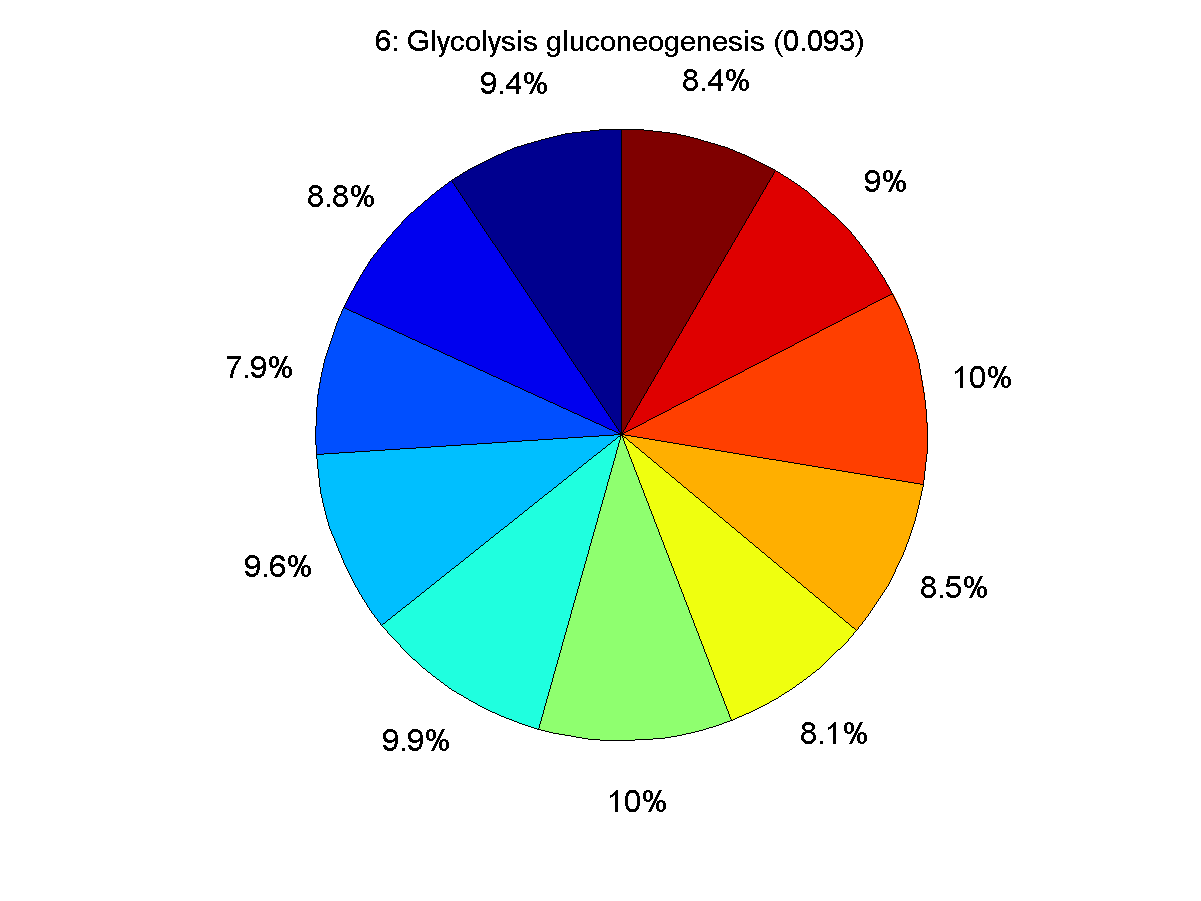

Supplement: S2 File — (ZIP) [file pone.0131875.s003.zip › MFC PieCharts/RegrEx2MFC/6Glycolysisgluconeogenesis.tif]

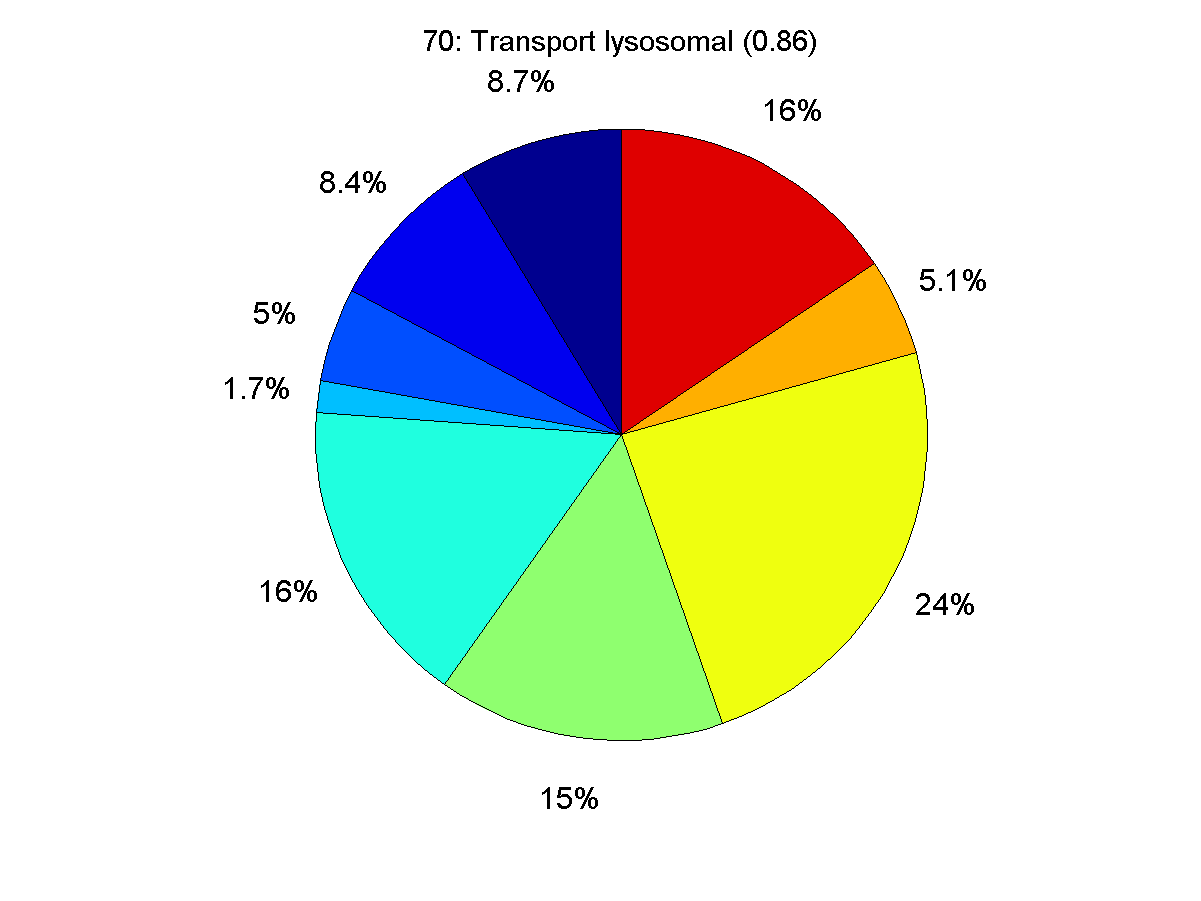

Supplement: S2 File — (ZIP) [file pone.0131875.s003.zip › MFC PieCharts/RegrEx2MFC/70Transportlysosomal.tif]

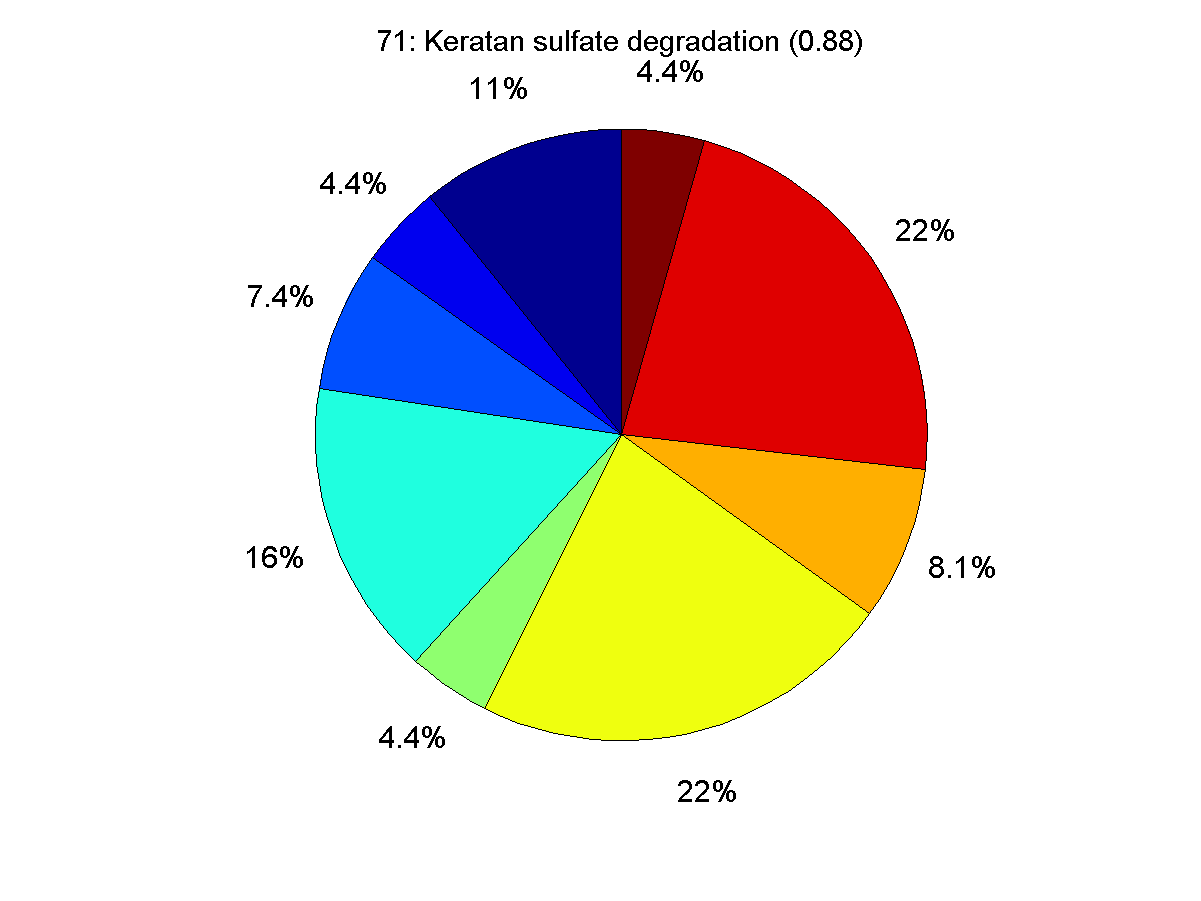

Supplement: S2 File — (ZIP) [file pone.0131875.s003.zip › MFC PieCharts/RegrEx2MFC/71Keratansulfatedegradation.tif]

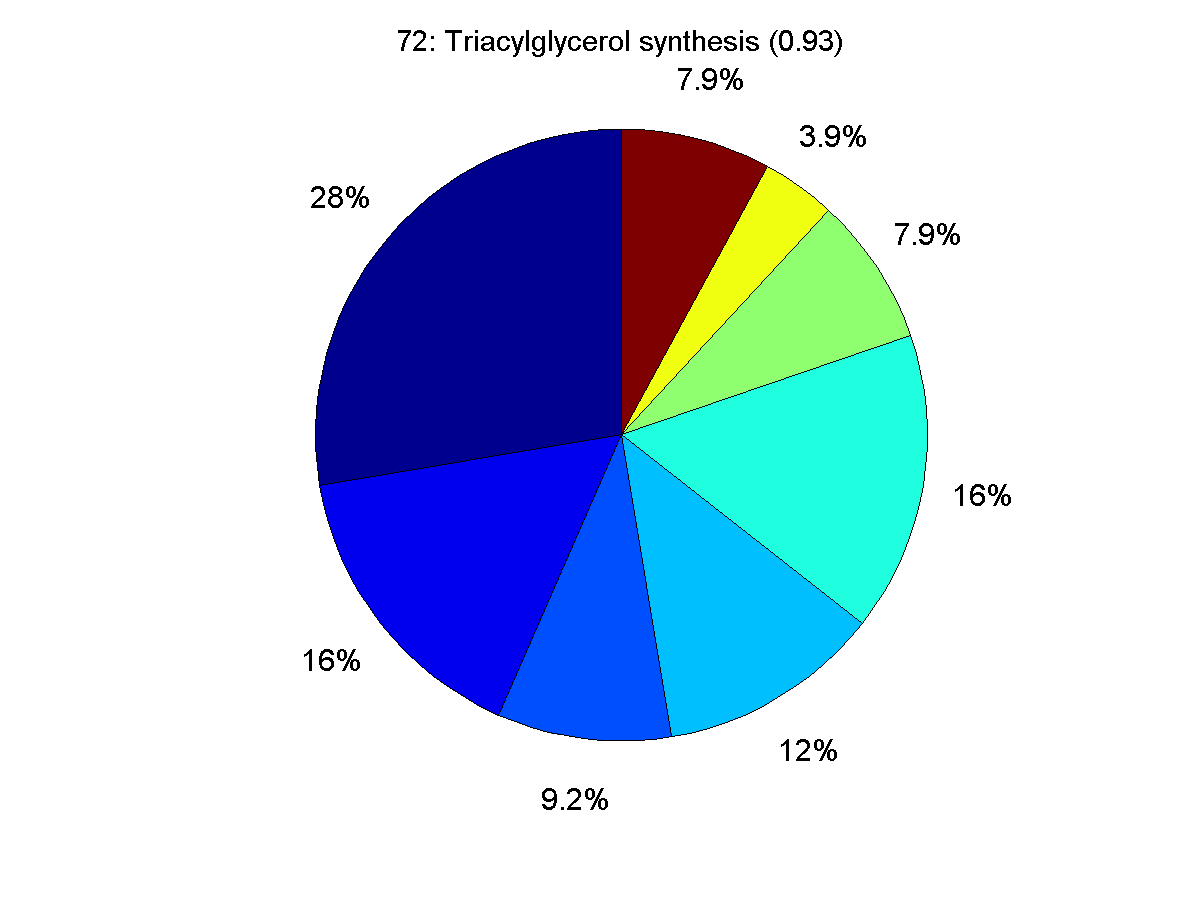

Supplement: S2 File — (ZIP) [file pone.0131875.s003.zip › MFC PieCharts/RegrEx2MFC/72Triacylglycerolsynthesis.tif]

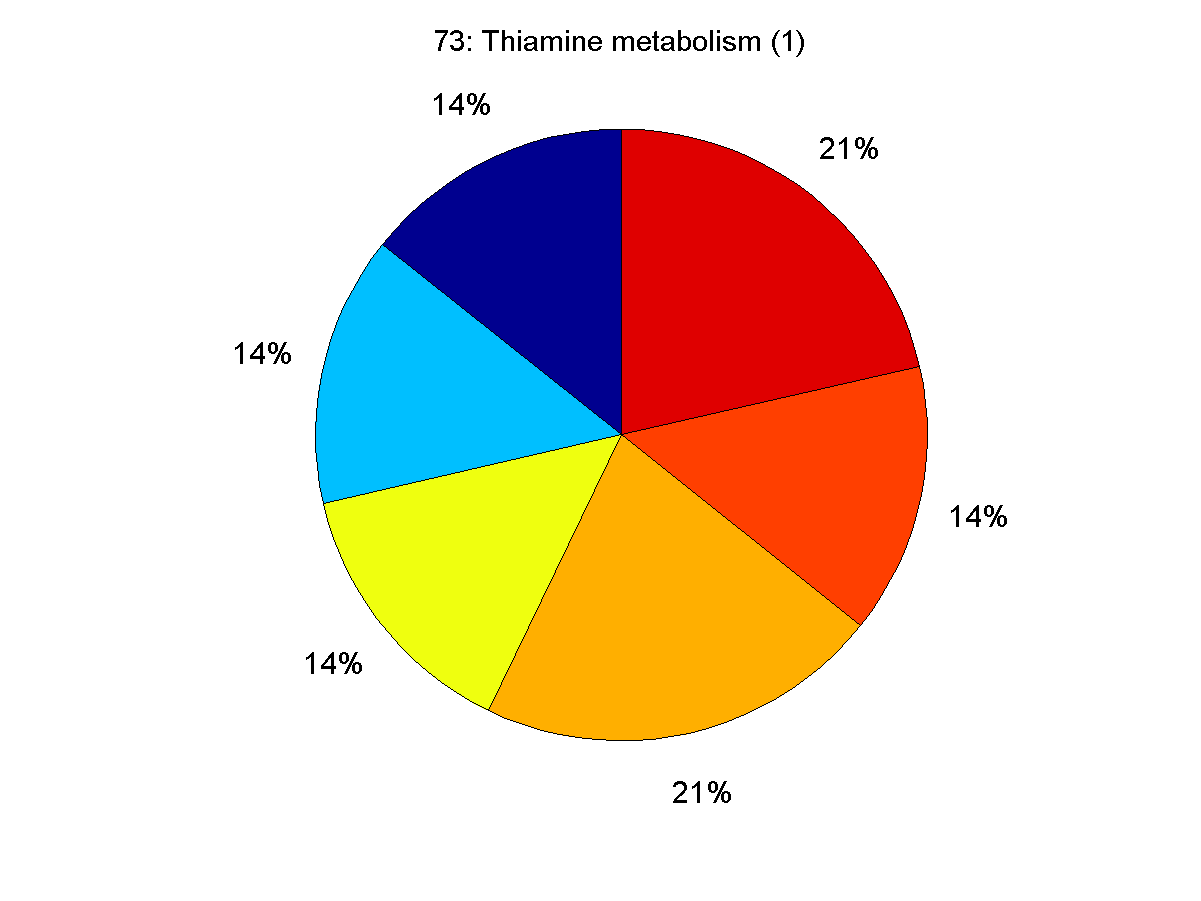

Supplement: S2 File — (ZIP) [file pone.0131875.s003.zip › MFC PieCharts/RegrEx2MFC/73Thiaminemetabolism.tif]

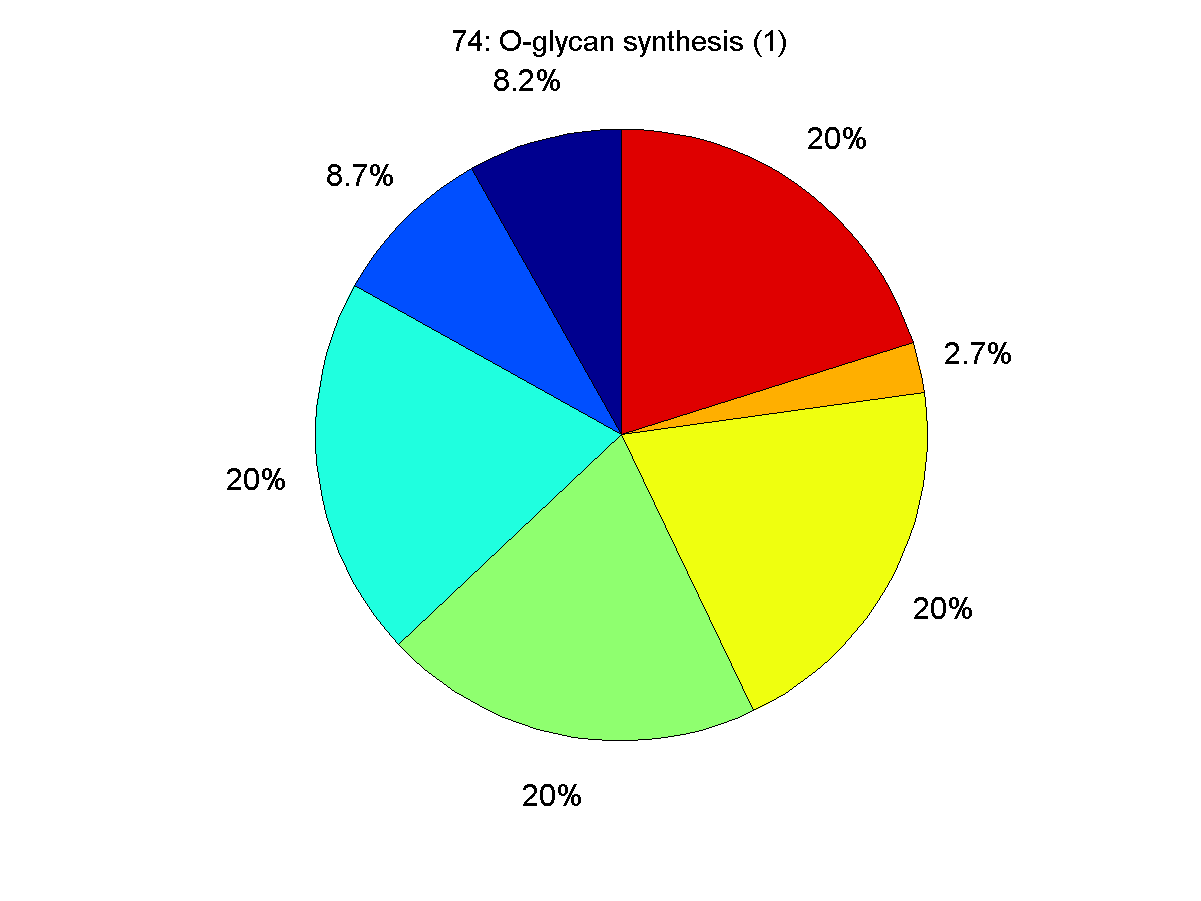

Supplement: S2 File — (ZIP) [file pone.0131875.s003.zip › MFC PieCharts/RegrEx2MFC/74O-glycansynthesis.tif]

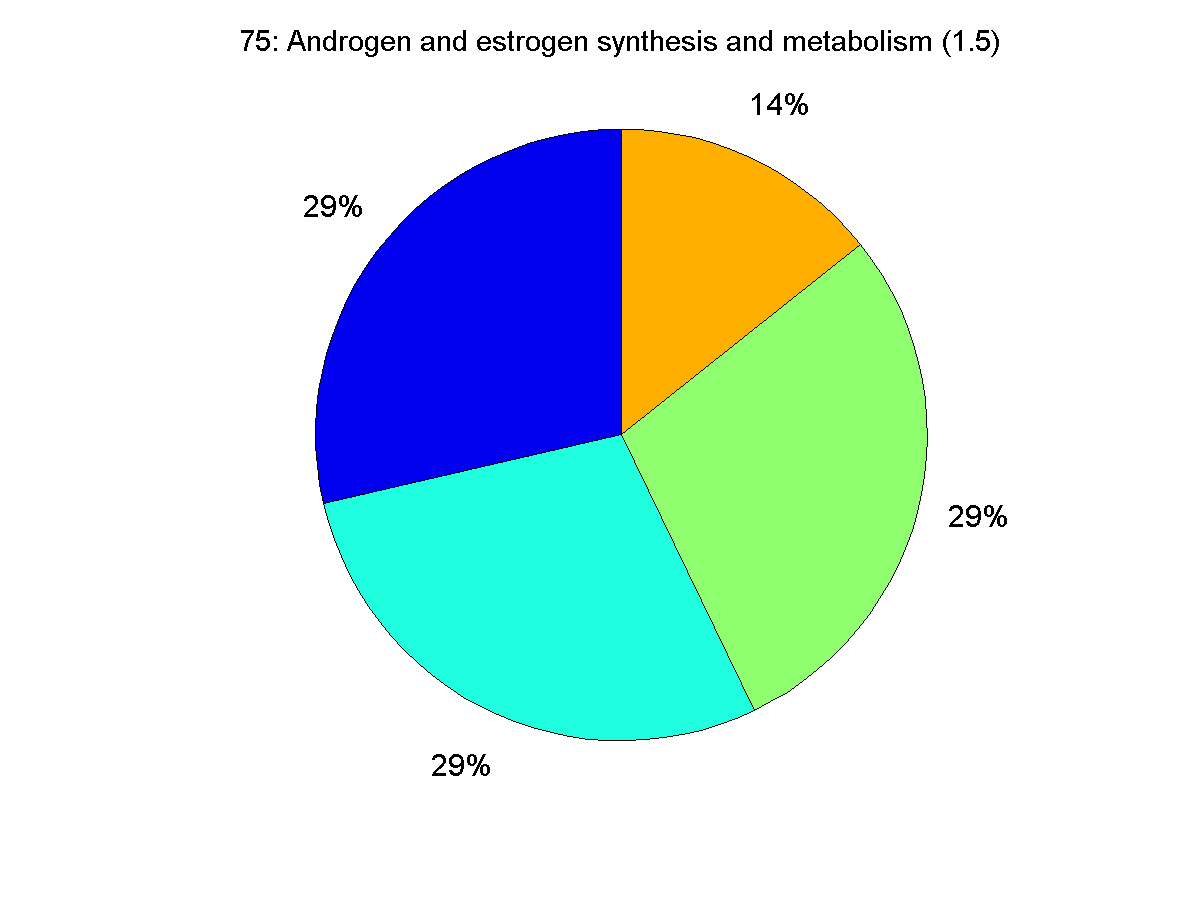

Supplement: S2 File — (ZIP) [file pone.0131875.s003.zip › MFC PieCharts/RegrEx2MFC/75Androgenandestrogensynthesisandmetabolism.tif]

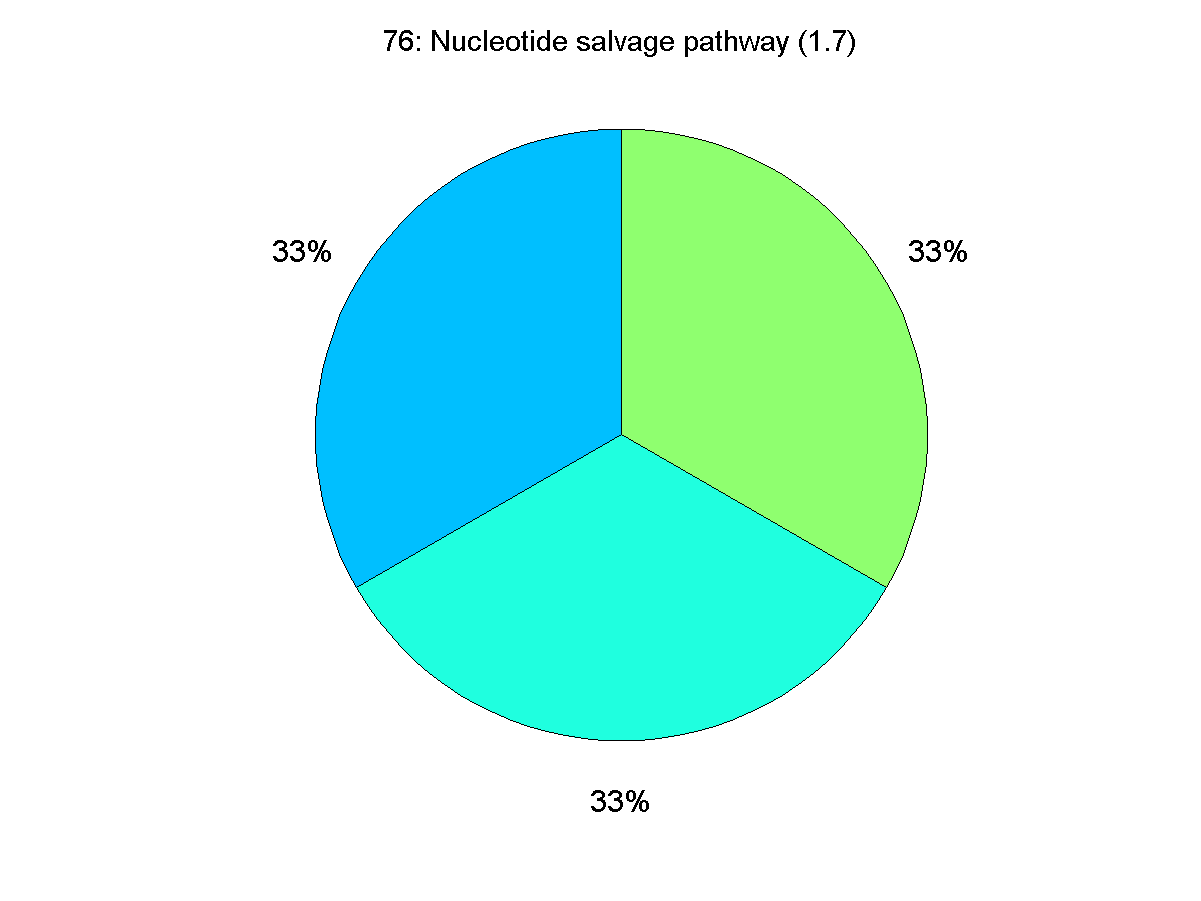

Supplement: S2 File — (ZIP) [file pone.0131875.s003.zip › MFC PieCharts/RegrEx2MFC/76Nucleotidesalvagepathway.tif]

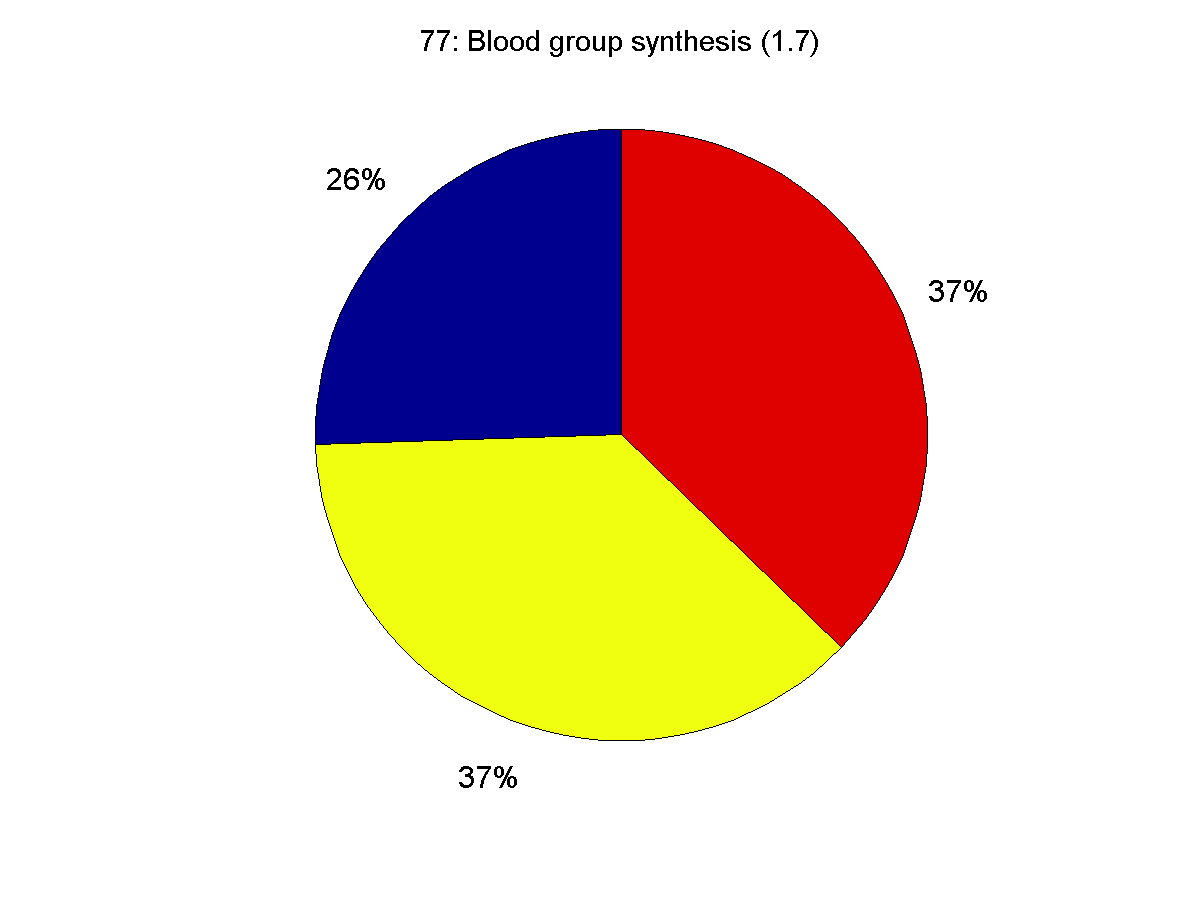

Supplement: S2 File — (ZIP) [file pone.0131875.s003.zip › MFC PieCharts/RegrEx2MFC/77Bloodgroupsynthesis.tif]

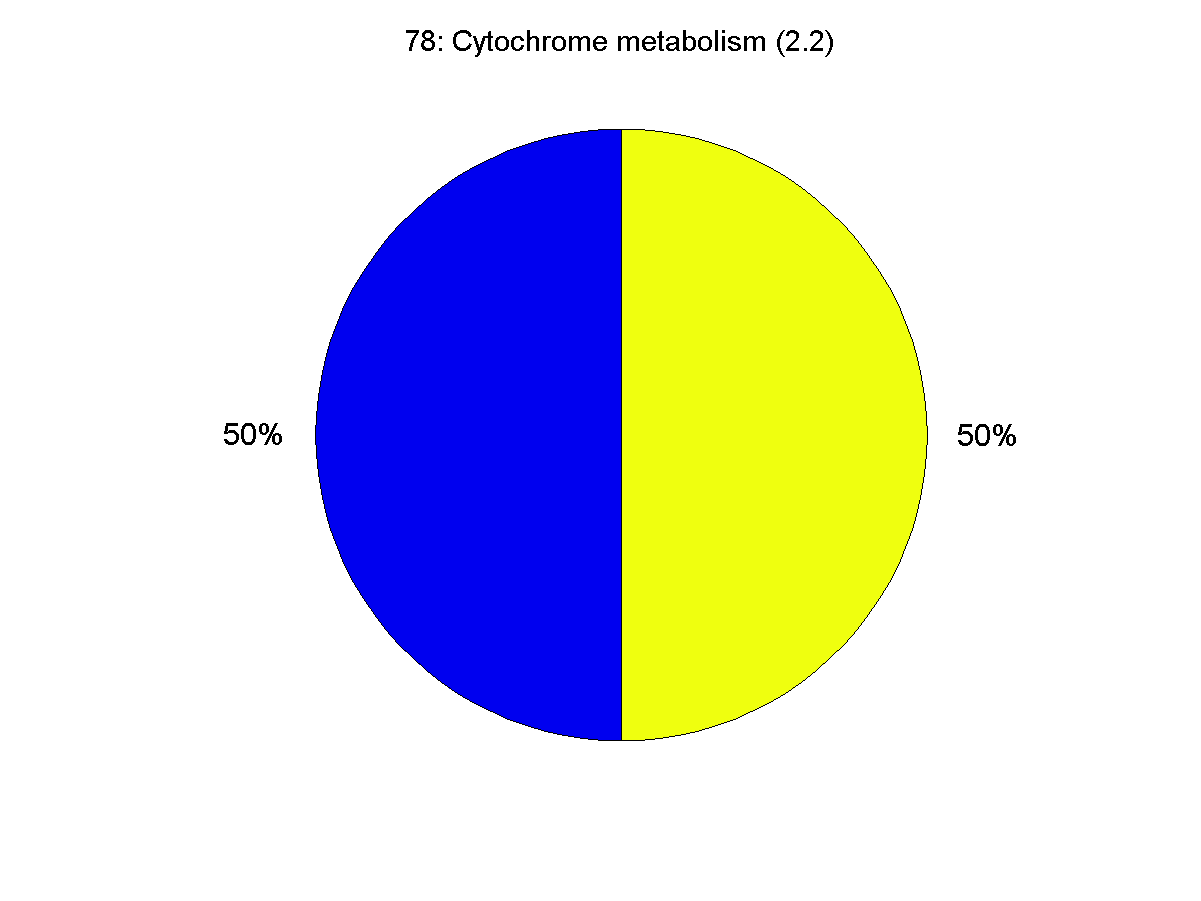

Supplement: S2 File — (ZIP) [file pone.0131875.s003.zip › MFC PieCharts/RegrEx2MFC/78Cytochromemetabolism.tif]

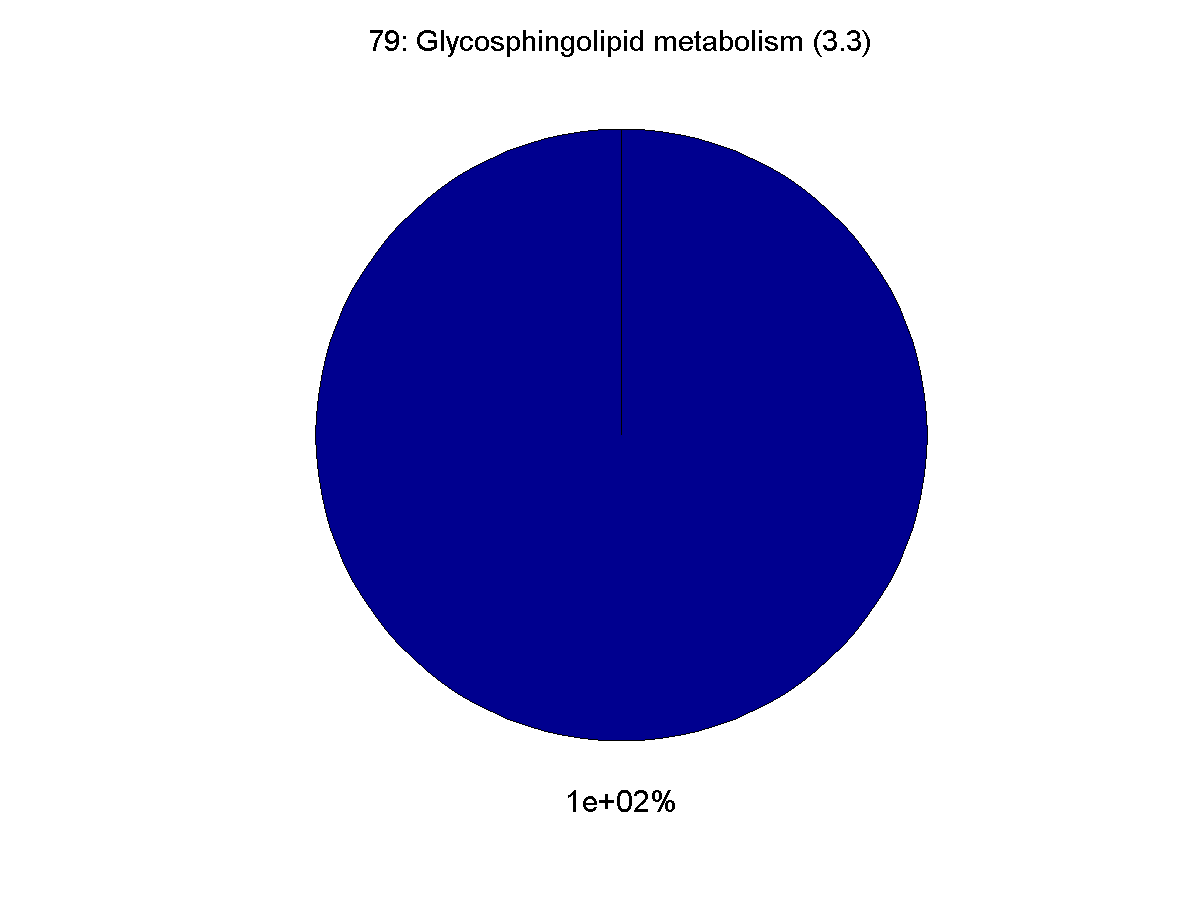

Supplement: S2 File — (ZIP) [file pone.0131875.s003.zip › MFC PieCharts/RegrEx2MFC/79Glycosphingolipidmetabolism.tif]

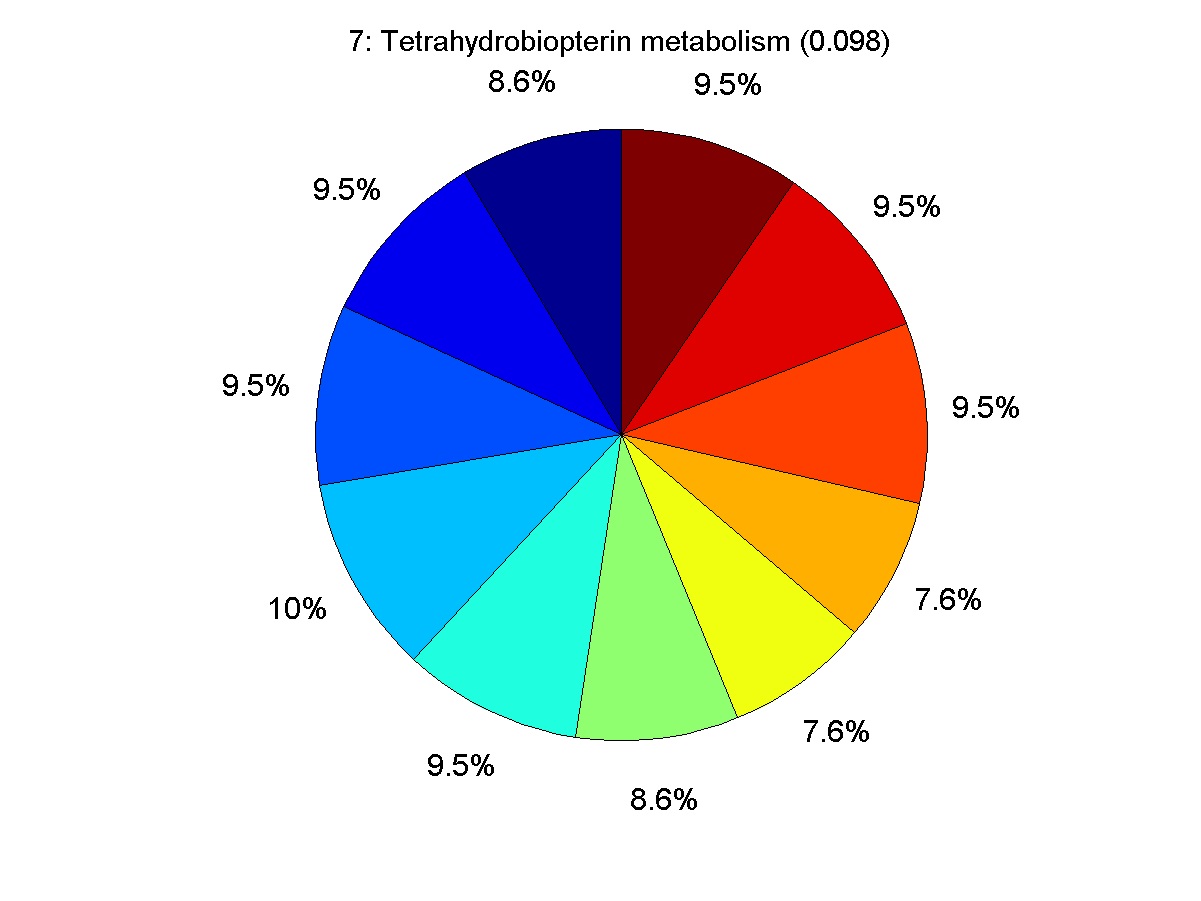

Supplement: S2 File — (ZIP) [file pone.0131875.s003.zip › MFC PieCharts/RegrEx2MFC/7Tetrahydrobiopterinmetabolism.tif]

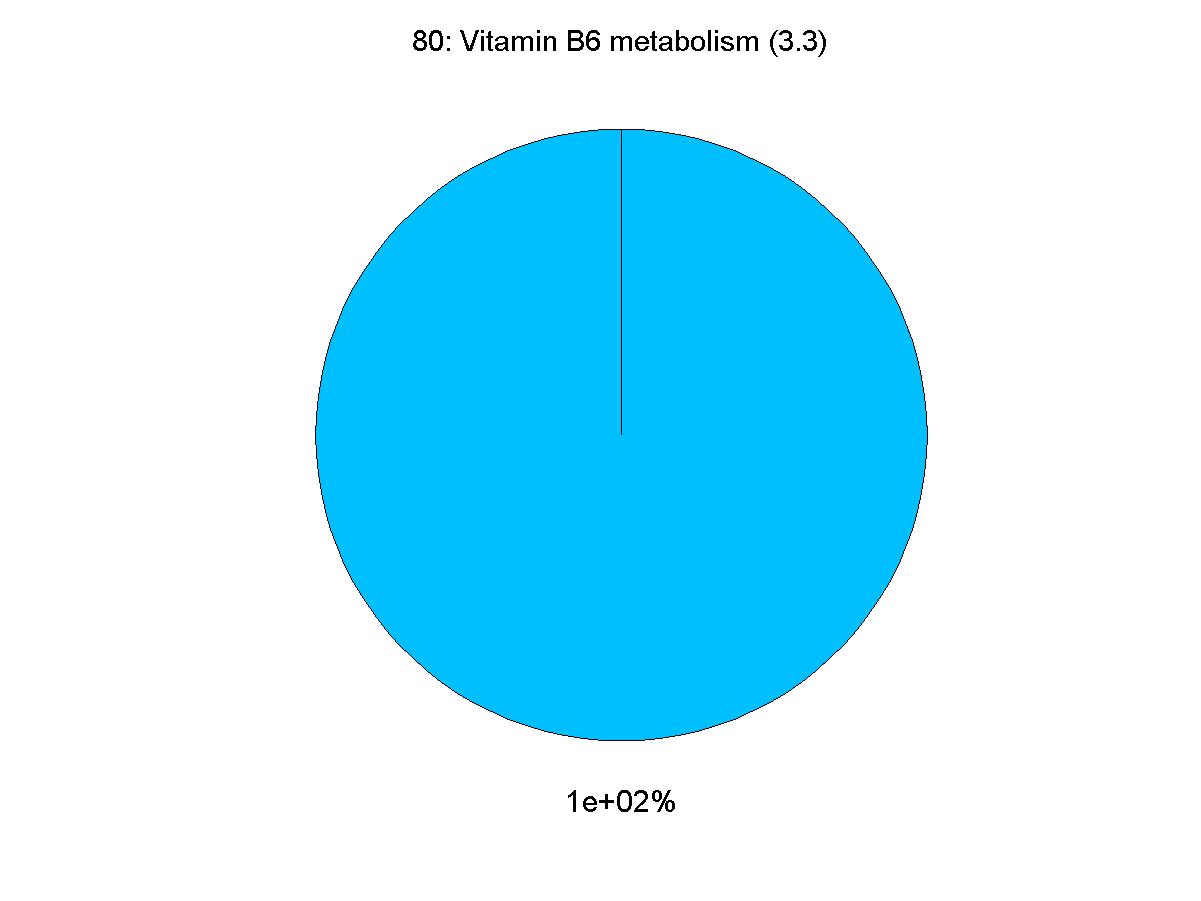

Supplement: S2 File — (ZIP) [file pone.0131875.s003.zip › MFC PieCharts/RegrEx2MFC/80VitaminB6metabolism.tif]

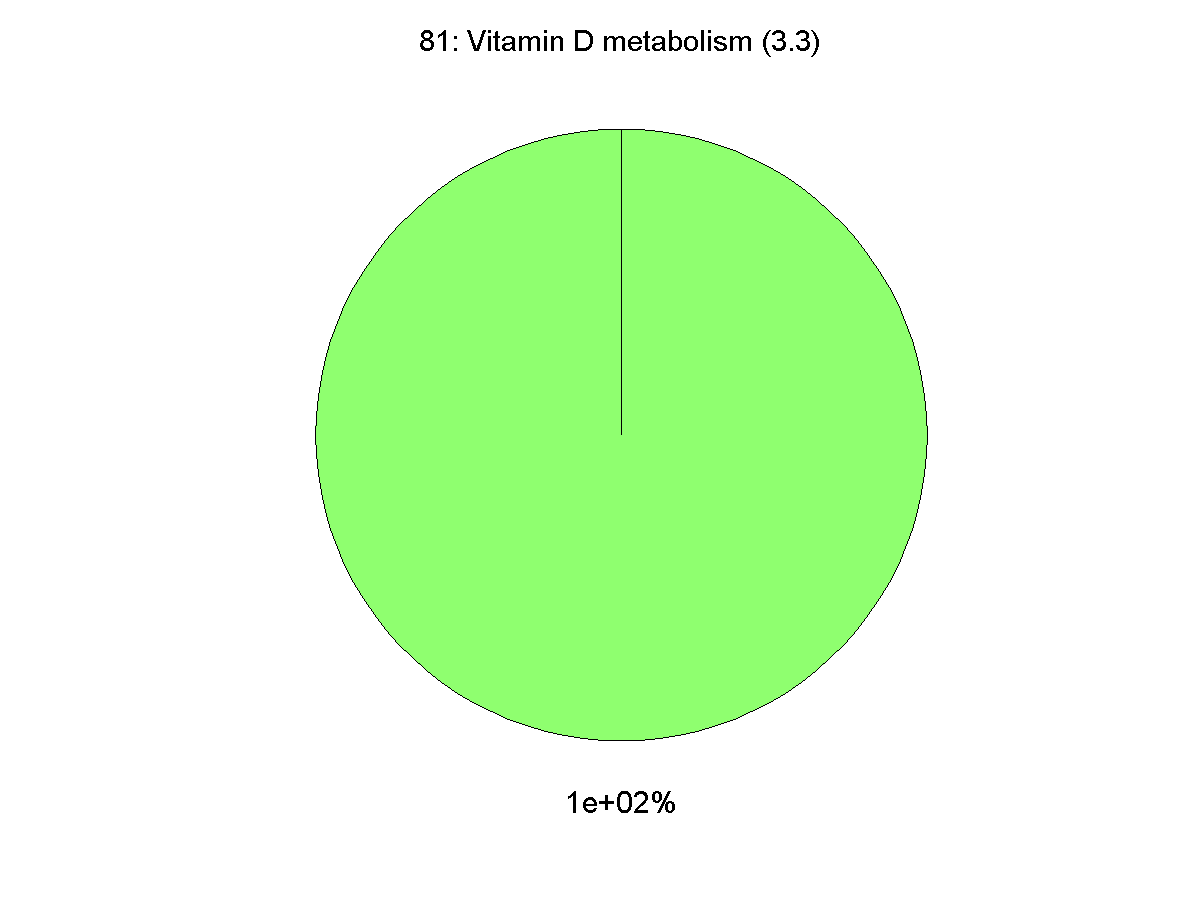

Supplement: S2 File — (ZIP) [file pone.0131875.s003.zip › MFC PieCharts/RegrEx2MFC/81VitaminDmetabolism.tif]

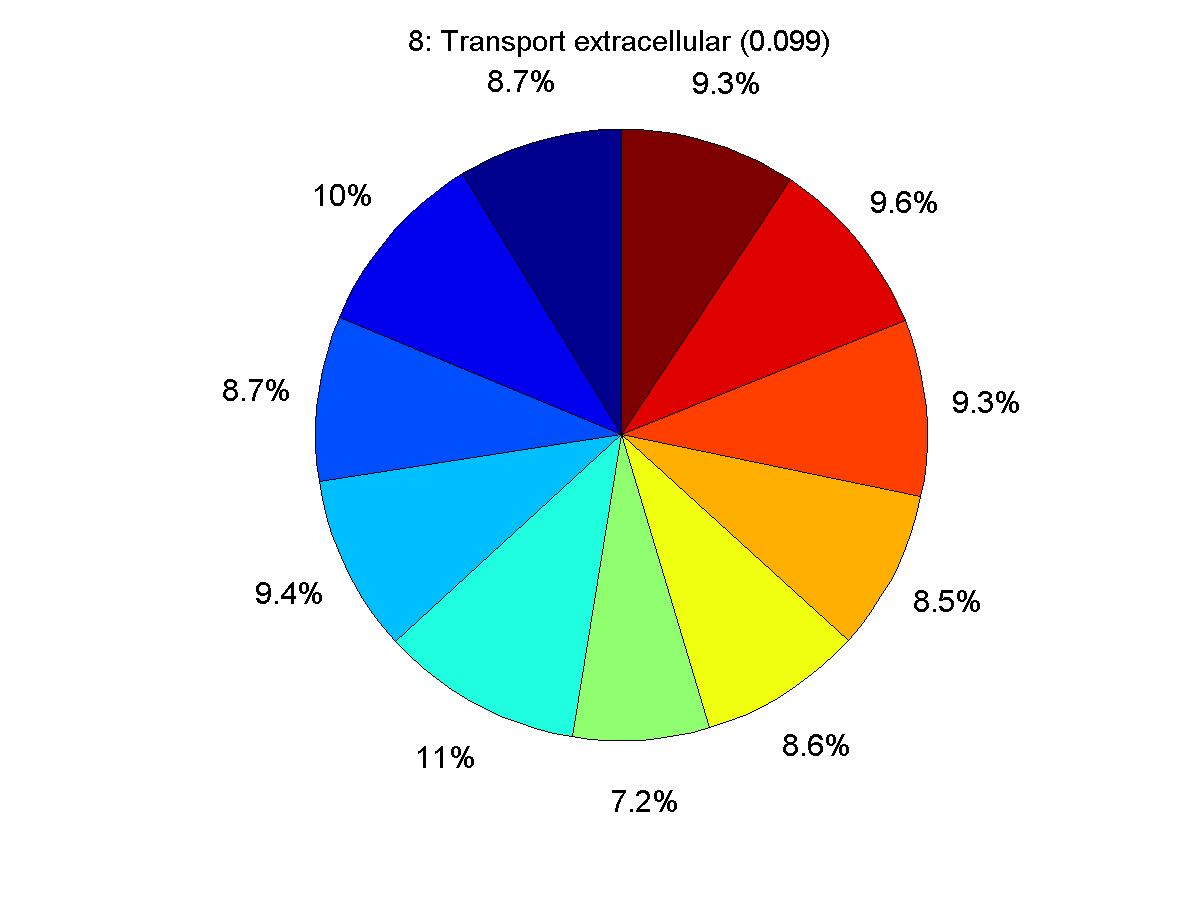

Supplement: S2 File — (ZIP) [file pone.0131875.s003.zip › MFC PieCharts/RegrEx2MFC/8Transportextracellular.tif]

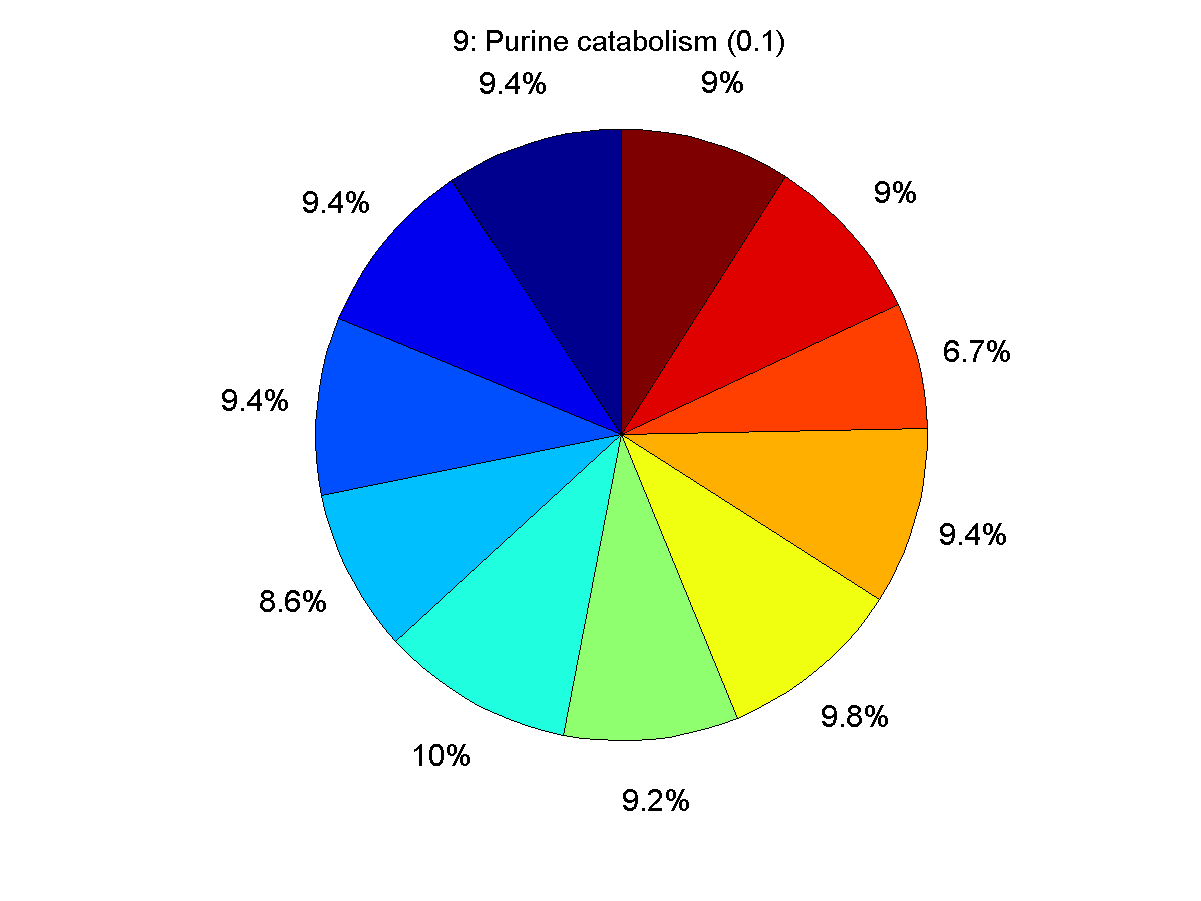

Supplement: S2 File — (ZIP) [file pone.0131875.s003.zip › MFC PieCharts/RegrEx2MFC/9Purinecatabolism.tif]

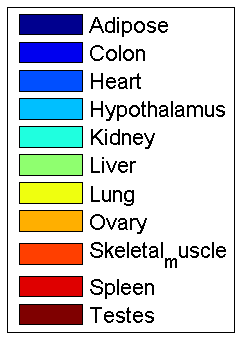

Supplement: S2 File — (ZIP) [file pone.0131875.s003.zip › MFC PieCharts/RegrEx2MFC/Legend.tif]
